# Open-Ended Metallodithiolene Complexes with the 1,2,4,5-Tetrakis(diphenylphosphino)benzene Ligand: Modular Building Elements for the Synthesis of Multimetal Complexes

**DOI:** 10.1021/acs.inorgchem.1c01573

**Published:** 2021-08-09

**Authors:** Satyendra Kumar, Malathy Selvachandran, Kuppuswamy Arumugam, Mohamed C. Shaw, Che Wu, Michael Maurer, Xiaodong Zhang, Stephen Sproules, Joel T. Mague, James P. Donahue

**Affiliations:** § Department of Chemistry, Tulane University, 6400 Freret Street, New Orleans, Louisiana 70118, United States; ⊥ Department of Chemistry, Wright State University, 3640 Colonel Glenn Highway, Dayton, Ohio 45435-0001, United States; ∥ WestCHEM, School of Chemistry, University of Glasgow, Glasgow G12 8QQ, United Kingdom

## Abstract

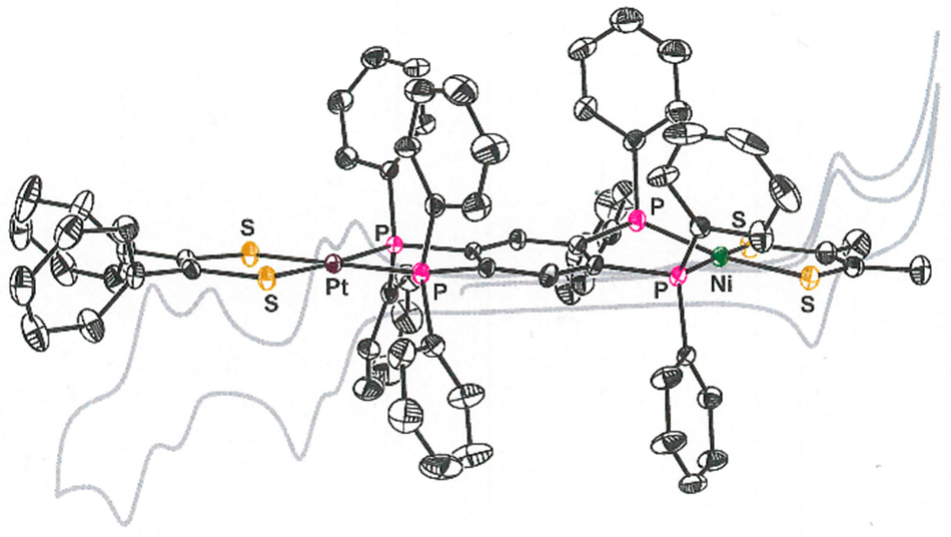

Open-ended, singly metalated dithiolene complexes with 1,2,4,5-tetrakis(diphenylphosphino)benzene (tpbz) are prepared either by ligand transfer to [Cl_2_M(tpbz)] from (R_2_C_2_S_2_)SnR′_2_ (R = CN, R′ = Me; R = Me, R′ = ^*n*^Bu) or by a direct reaction between tpbz and [M(S_2_C_2_R_2_)_2_] (M = Ni, Pd, Pt; R = Ph, *p*-anisyl) in a 1:1 ratio. The formation of dimetallic [(R_2_C_2_S_2_)M(tpbz)M(S_2_C_2_R_2_)] attends these syntheses in modest amounts, but the open-ended compounds are readily separated by silica chromatography. As affirmed by X-ray crystallographic characterization of numerous members of the set, the [(R_2_C_2_S_2_)M(tpbz)] compounds show dithiolene ligands in their fully reduced ene-1,2-dithiolate form conjoined with divalent Group 10 ions. Minor amounts of octahedral [(Ph_2_C_2_S_2_)_2_Pt^IV^(tpbz)], a presumed intermediate, are isolated from the preparation of [(Ph_2_C_2_S_2_)Pt^II^(tpbz)]. Heterodimetallic [(Ph_2_C_2_S_2_)Pt(tpbz)Ni(S_2_C_2_Me_2_)] is prepared from [(Ph_2_C_2_S_2_)Pt^II^(tpbz)]; its cyclic voltammogram, upon anodic scanning, shows two pairs of closely spaced, but resolved, 1e^–^ oxidations corresponding first to [R_2_C_2_S_2_^2^^–^] – 1e^–^ → [R_2_C_2_S^•^S^–^] and then to [R_2_C_2_S^•^S^–^] – 1e^–^ → [R_2_(C=S)_2_]. The open diphosphine of [(R_2_C_2_S_2_)M(tpbz)] can be oxidized to afford open-ended [(R_2_C_2_S_2_)M(tpbzE_2_)] (E = O, S). Synthesis of the octahedral [(dppbO_2_)_3_Ni][I_3_]_2_ [dppbO_2_ = 1,2-bis(diphenylphosphoryl)benzene] suggests that the steric profile of [(R_2_C_2_S_2_)M(tpbzE_2_)] is moderated enough that three could be accommodated as ligands around a metal ion.

## Abstract 2

Open-ended metallodithiolene complexes with 1,2,4,5-tetrakis(diphenylphosphino)benzene (tpbz) are prepared either by the reaction of [M(S_2_C_2_R_2_)_2_] with tpbz in a 1:1 ratio or by transmetalation from [(R_2_C_2_S_2_)SnR′_2_] to [Cl_2_M(tpbz)]. ^31^P NMR spectroscopy provides a clear diagnostic with the metalated (∼55 ppm) and open-phosphine (−14 ppm) signals. These compounds may be metalated at their open end to produce heterodimetallic compounds, or the open phosphines may be oxidized with chalcogen donors to the corresponding phosphine oxides or sulfides.

## Introduction

**Scheme 1:**
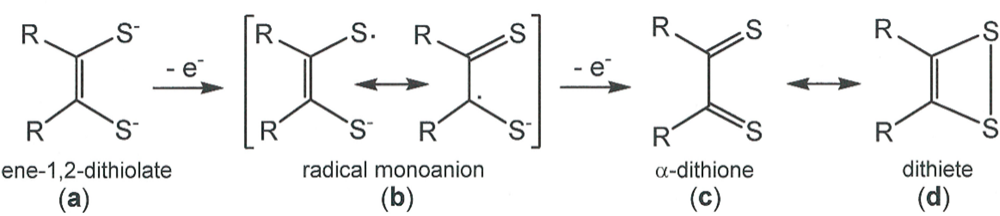
Redox Levels Available to a Dithiolene Ligand When Bound to a Transition Metal

In recent work,^1,2^ we have reported the synthesis, structures, and properties of a set of dimetallic compounds of the type [(R_2_C_2_S_2_)M(tpbz)M(S_2_C_2_R_2_)] [tpbz = 1,2,4,5-tetrakis(diphenylphosphino)benzene], where R may be CN, Me, Ph, or *p*-anisyl and, independent of R, M may be varied as Ni^2+^, Pd^2+^, or Pt^2+^. The dithiolene end groups can be concurrently oxidized to radical monoanions (**a** → **b**, Scheme 1), thus providing [(R_2_C_2_S^–^S^•^)M(tpbz)M(S^–^S^•^C_2_R_2_)]^2+^ dications, which weakly couple to provide nearly isoenergetic *S* = 1 and 0 states in equilibrium. Electron paramagnetic resonance (EPR) spectroscopy is effective in characterizing subtle differences among the [(R_2_C_2_S_2_)M(tpbz)M(S_2_C_2_R_2_)]^2+^ complexes as M and R are varied. For example, simulations of the EPR spectra of [((MeO-*p*-C_6_H_4_)_2_C_2_S_2_)M(tpbz)M(S_2_C_2_(C_6_H_4_-*p*-OMe)_2_)]^2+^, where M = Ni^2+^ or Pd^2+^, yield *D* = −18 × 10^–4^ cm^–1^ and −15 × 10^–4^ cm^–1^, respectively, with the former value indicative of a slightly shorter distance between the spin barycenters because of greater spin delocalization onto the metal from the dithiolene radicals.

**Figure 1:**
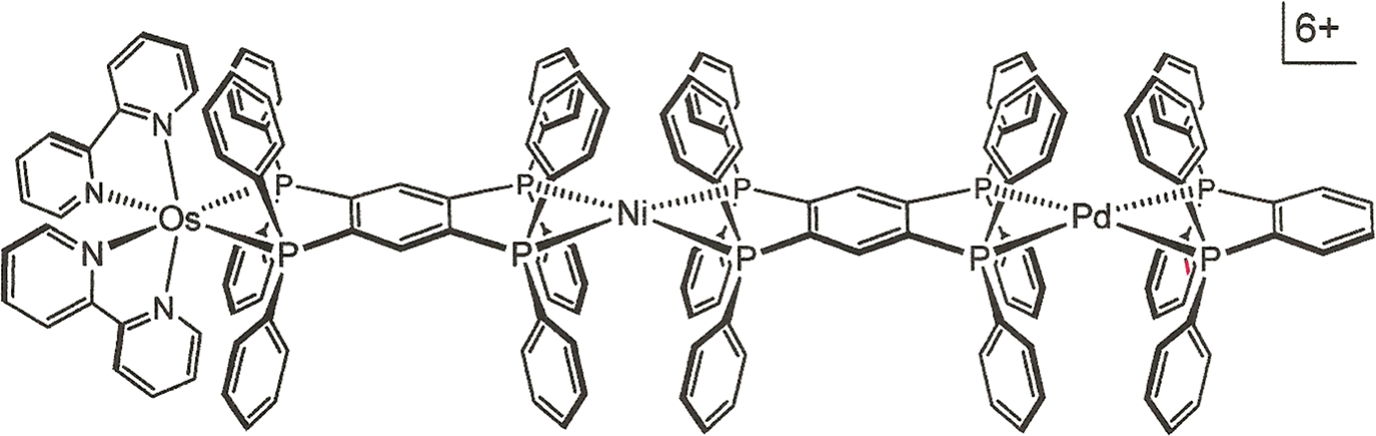
A previously reported^3^ tpbz-linked heterotrimetallic complex designed to support gated photoinduced electron transfer.

The conditions for the syntheses of [(R_2_C_2_S_2_)M(tpbz)M(S_2_C_2_R_2_)] that we reported were devised to promote formation of the dimetallic species. However, when the tpbz ligand is introduced to a source of the Group 10 metal bis(dithiolene) complex in an amount that is greater than the 1:2 ratio that is optimal for [(R_2_C_2_S_2_)M(tpbz)M(S_2_C_2_R_2_)], we have noted that “open-ended” tpbz compounds can be isolated in which only one of the two chelating sites is occupied. Although the centrosymmetic dimetal compounds still form, they are readily separated from the open-ended compounds because of their lowered solubility and slower movement on silica columns. These open-ended monometallic compounds still possess the air and moisture stability enjoyed by the free tpbz ligand itself, thus making them easily accessible in quantities that can be deployed for further synthetic ends. In every sense of the term, these open-ended complexes are themselves ligands, or “modules,” for the systematic creation of higher-order assemblies incorporating other metal ions with the capacity for a specific function. Zahavy and Fox, for example, described trimetallic [(bpy)_2_Os(tpbz)Ni(tpbz)Pd(dppb)]^6+^ (Figure 1), the synthesis of which proceeded through open-ended [(bpy)_2_Os(tpbz)]^2+^, as a photoinduced Os → Pd electron transfer device with gating controlled by the redox state at Ni.^3^

In general terms, modular chemical synthesis may be described as the preparation of materials or discrete compounds from two or more sets of parts such that (1) an array of products whose number is the product of the numbers of the members of the constituents sets can be obtained, (2) a common synthetic methodology can be executed for the synthesis of all products, and (3) desirable properties of the resulting materials, such as the redox potential or acidity, may be varied with a predictable effect. Arguably, the most prominent example of modular synthesis is the synthesis of metal–organic frameworks, wherein “nodes” defined by transition-metal ions with varying coordination environment constraints are joined by any of an array of chemical linkers to produce open spacings or channels.^4^ A rather different example from Lu and co-workers is the installment, via a two-step metalation protocol, of heterodimetallic units within a variety of tripodal ligands whose arms feature three-atom bridges.^5^ An obvious advantage of modular synthesis is the efficiency it can provide toward identifying an optimal candidate for a particular application.

**Scheme 2:**
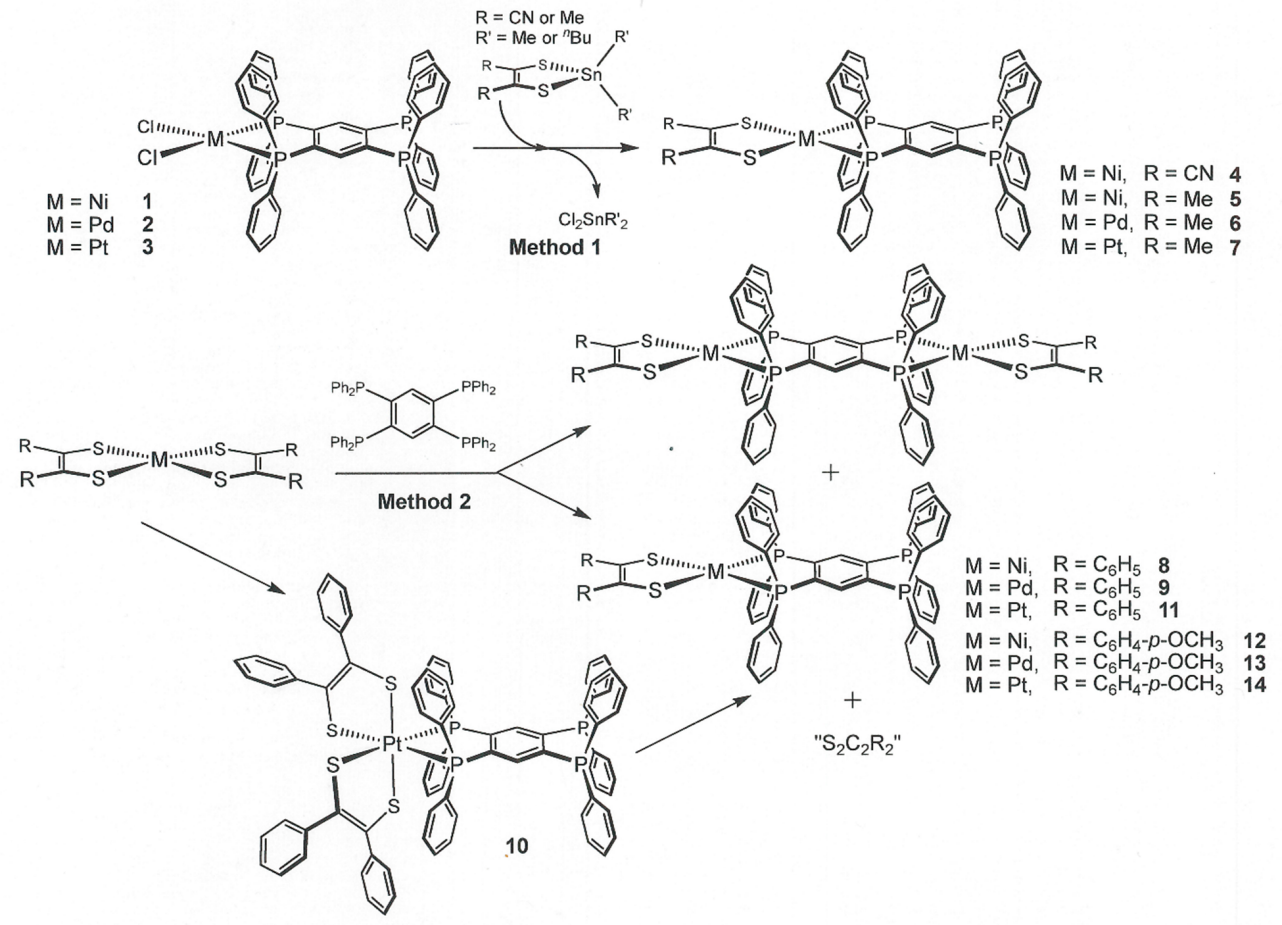
Syntheses of Open-Ended [(R_2_C_2_S_2_)M(tpbz)] (M = Ni^2+^, Pd^2+^, Pt^2+^; R = Me, Ph, CH_3_O-*p*-C_6_H_4_, CN) Compounds

**Scheme 3:**
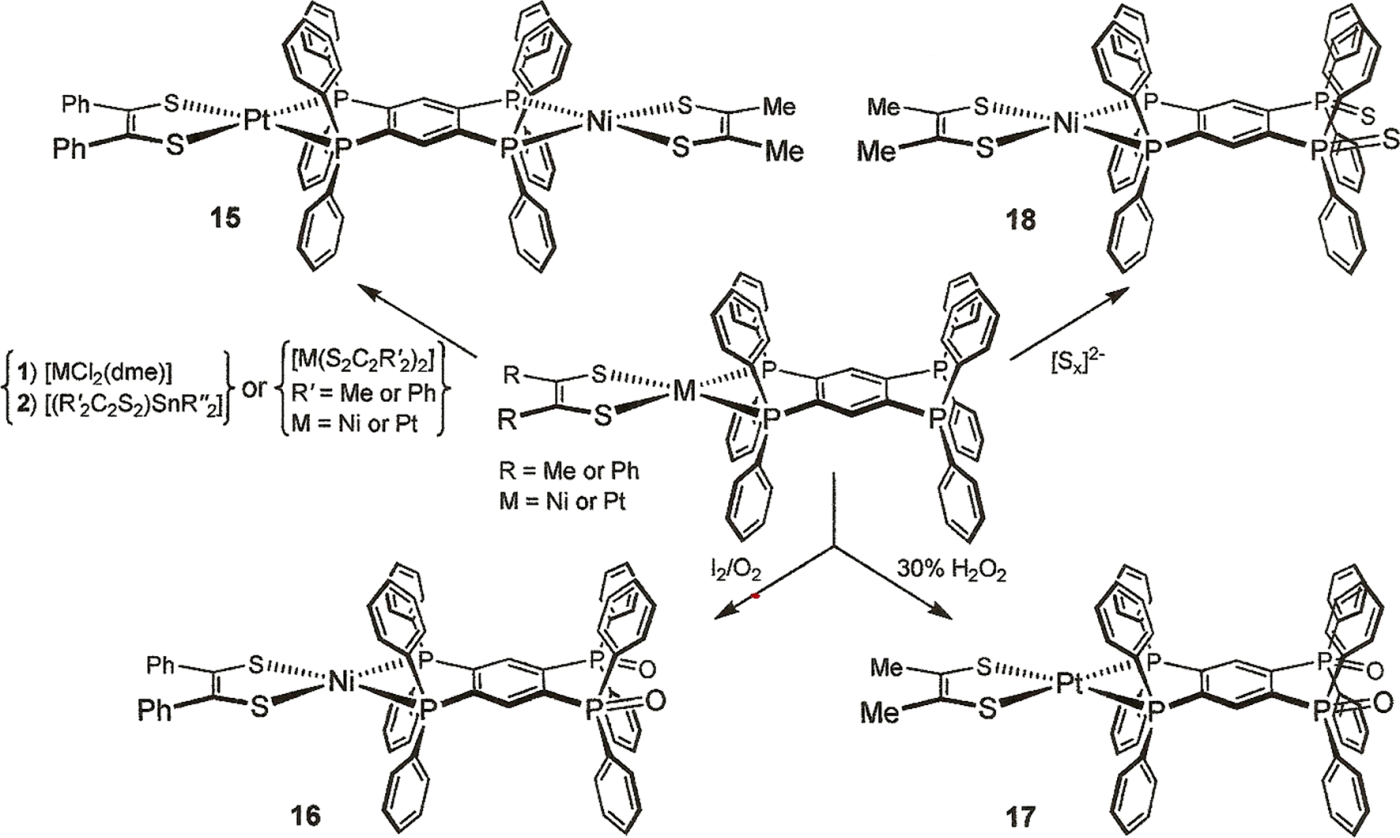
Syntheses of Pt–Ni Heterodimetallic **15** and Oxidized Open-Ended Compounds **16**–**18**

In this report, we provide an account of the synthesis and characterization of the open-ended [(R_2_C_2_S_2_)M(tpbz)] compounds that are summarized pictorially in Scheme 2. A particular emphasis is placed on the solid-state molecular structures and on ^31^P NMR spectroscopy as a convenient and definitive handle for characterization of these compounds. Following their isolation, postsynthetic modification of [(R_2_C_2_S_2_)M(tpbz)] by oxidation of the noncoordinating P sites to the corresponding phosphine oxide or sulfide is possible and provides a further dimension of variability for this metallodithiolene “module” (Scheme 3). Using isolated [(R_2_C_2_S_2_)M(tpbz)], asymmetric heterodimetallic complexes of the form [(R_2_C_2_S_2_)M(tpbz)M′(S_2_C_2_R′_2_)] (M′ ≠ M; R ≠ R′) are readily prepared that are not accessible by the method originally disclosed for [(R_2_C_2_S_2_)M(tpbz)M(S_2_C_2_R_2_)].

## Syntheses

### General Considerations

Literature methods were implemented for the syntheses of [Cl_2_Ni(dme)]^6^ (dme = 1,2-dimethoxyethane), [Cl_2_M(NCCH_3_)_2_] (M = Pd^2+^, Pt^2+^),^7^ [(R_2_C_2_S_2_)_2_M] (R = Me, Ph, *p*-anisyl; M = Ni^2+^, Pd^2+^, Pt^2+^),^8^ [((NC)_2_C_2_S_2_)SnMe_2_], [(Me_2_C_2_S_2_)Sn(^*n*^Bu)_2_], the tpbz ligand,^9^ and 1,2-bis(diphenylphosphino)benzene dioxide (dppbO_2_).^10^ All other reagents were purchased from commercial sources and used as received. Solvents were either dried with a system of drying columns from the Glass Contour Company [dichloromethane (CH_2_Cl_2_), *n*-pentane, hexanes, diethyl ether (Et_2_O), tetrahydrofuran (THF), benzene (C_6_H_6_), and toluene] or freshly distilled according to standard procedures [methanol (MeOH), acetonitrile (CH_3_CN), and 1,2-dichloroethane].^11^ All reactions described below were conducted under an atmosphere of N_2_, while silica columns were run in the open air using 60–230 μm silica (Dynamic Adsorbents). Dithiolene ligand abbreviations used throughout the text are as follows: mdt = [Me_2_C_2_S_2_]^2–^ = 1,2-dimethyl-1,2-dithiolate(2−); mnt = [(NC)_2_C_2_S_2_]^2–^ = maleonitriledithiolate(2−); pdt = [Ph_2_C_2_S_2_]^2–^ = 1,2-diphenyl-1,2-dithiolate(2−); adt = [(MeO-*p*-C_6_H_4_)_2_C_2_S_2_]^2–^ = 1,2-di-*p*-anisyl-1,2-dithiolate(2−).

### Physical Methods

All ^1^H and ^31^P NMR spectra were recorded at 25 °C with a Bruker Avance spectrometer operating at 300.13 and 121.49 MHz for the ^1^H and ^31^P nuclei, respectively. The ^1^H NMR spectra were referenced to the solvent signal, while an external aqueous phosphoric acid (H_3_PO_4_) solution was employed as the reference for all ^31^P NMR spectra. Mass spectra (positive-ion electrospray ionization, ESI^+^) were obtained with a Bruker micrOTOF II mass spectrometer. The X-band EPR spectrum was recorded on a Bruker ELEXSYS E500 spectrometer. Electrochemical measurements were made with a CHI620C electroanalyzer workstation using a Ag/AgCl reference electrode, a glassy carbon or Pt disk working electrode, a Pt wire auxiliary electrode, and a [^*n*^Bu_4_N][PF_6_] supporting electrolyte. Under these conditions, the Cp_2_Fe^+^/Cp_2_Fe (Fc^+^/Fc) couple consistently occurred at +0.436 mV in CH_2_Cl_2_. Elemental analyses were performed by Midwest Microlab, LLC (Indianapolis, IN), Galbraith Laboratories, Inc. (Knoxville, TN), or Kolbe Microanalytical Laboratory (Oberhausen, Germany). Procedural details regarding crystal growth, X-ray diffraction data collection, data processing, and structure solution and refinement are available in the Supporting Information. Unit cell data and selected refinement statistics are presented in Table 1.

**Table 1:** Unit Cell and Refinement Data for Compounds Characterized by X-ray Diffraction

compound	[Cl_2_Ni(tpbz)]	[(mnt)Ni(tpbz)]	[(mdt)Ni(tpbz)]	[(mdt)Pt(tpbz)]
compound no.	**1**	**4**	**5**	**7**
cocryst solvent	Et_2_O	2CHCl_3_	2CH_2_Cl_2_	2CH_2_Cl_2_
formula	C_58_H_52_Cl_2_NiOP_4_	C_60_H_44_Cl_6_N_2_NiP_4_S_2_	C_60_H_52_Cl_4_NiP_4_S_2_	C_60_H_52_Cl_4_P_4_PtS_2_
fw, g mol^–1^	1018.49	1252.38	1161.52	1297.91
temperature, K	100	100	100	100
wavelength, Å	0.71073	0.71073	0.71073	0.71073
2θ range, deg	2.34–55.70	4.22–56.74	3.24–60.44	3.22–56.56
cryst syst	orthorhombic	orthorhombic	monoclinic	monoclinic
space group	*Pna*2_1_	*P*2_1_2_1_2_1_	*C*2/*c*	*C*2/*c*
*a*, Å	34.703(3)	13.510(3)	21.223(2)	21.157(5)
*b*, Å	9.2939(9)	16.403(3)	15.7180(18)	15.836(4)
*c*, Å	15.5064(14)	25.380(5)	16.7123(18)	16.576(4)
α, deg	90	90	90	90
β, deg	90	90	100.039(2)	99.604(4)
γ, deg	90	90	90	90
volume (Å^3^), *Z*	5001.2(8), 4	5624.2(19), 4	5489.6(11), 4	5476(2), 4
density, g cm^–3^	1.353	1.479	1.405	1.574
μ, mm^–1^	0.664	0.860	0.780	2.991
color, habit	orange plate	orange block	brwn-grn column	yellow plate
limiting indices *h*	–44 ≤ *h* ≤ 45	–17 ≤ *h* ≤ 18	–29 ≤ *h* ≤ 29	–28 ≤ *h* ≤ 27
limiting indices *k*	–12 ≤ *k* ≤ 12	–21 ≤ *k* ≤ 21	–22 ≤ *k* ≤ 22	–20 ≤ *k* ≤ 20
limiting indices *l*	–20 ≤ *l* ≤ 19	–33 ≤ *l* ≤ 33	–23 ≤ *l* ≤ 23	–21 ≤ *l* ≤ 21
reflns collected	40918	100119	52873	24025
indep data, param^a^	11569, 597	14060, 676	7799, 322	6526, 322
GOF^b^	1.090	1.048	1.105	1.035
R1,^c^^,^^d^ wR2^d^^,^^e^	0.0574, 0.1259	0.0413, 0.1087	0.0450, 0.1434	0.0308, 0.0750
R1,^c^^,^^f^ wR2^e^^,^^f^	0.0725, 0.1327	0.0481, 0.1141	0.0590, 0.1468	0.0360, 0.0776

compound	[(pdt)Ni(tpbz)]	[(pdt)Pd(tpbz)]	[(pdt)Pt(tpbz)]	[(pdt)_2_Pt(tpbz)]
compound no.	**8**	**9**	**11**	**10**
cocryst solvent	none	none	none	2(ClCH_2_CH_2_Cl)
formula	C_68_H_52_NiP_4_S_2_	C_68_H_52_P_4_PdS_2_	C_68_H_52_P_4_PtS_2_	C_86_H_70_Cl_4_P_4_PtS_4_
fw, g mol^–1^	1115.80	1163.49	1252.18	1692.43
temperature, K	150	100	100	100
wavelength, Å	1.54178	0.71073	0.71073	0.71073
2θ range, deg	6.84–148.77	3.14–58.44	3.14–57.39	3.03–59.72
cryst syst	monoclinic	monoclinic	monoclinic	triclinic
space group	*C*2/*c*	*C*2/*c*	*C*2/*c*	*P*1̅
*a*, Å	21.9519(4)	21.7574(15)	21.7804(15)	13.9324(12)
*b*, Å	16.7831(3)	16.9716(12)	16.9661(12)	14.0989(12)
*c*, Å	16.4070(3)	16.2670(11)	16.2774(12)	21.2092(18)
α, deg	90	90	90	89.716(1)
β, deg	112.864(1)	112.666(1)	112.727(1)	84.452(1)
γ, deg	90	90	90	72.605(1)
volume (Å^3^), *Z*	5569.75(18), 4	5542.8(7), 4	5547.9(7), 4	3955.7(6), 2
density, g cm^–3^	1.331	1.394	1.499	1.421
μ, mm^–1^	2.612	0.569	2.763	2.139
color, habit	yellow block	pale-orange column	yellow column	dark-blue block
limiting indices *h*	–27 ≤ *h* ≤ 27	–29 ≤ *h* ≤ 28	–29 ≤ *h* ≤ 28	–19 ≤ *h* ≤ 19
limiting indices *k*	–20 ≤ *k* ≤ 20	–23 ≤ *k* ≤ 23	–22 ≤ *k* ≤ 22	–19 ≤ *k* ≤ 19
limiting indices *l*	–20 ≤ *l* ≤ 20	–22 ≤ *l* ≤ 22	–21 ≤ *l* ≤ 21	–29 ≤ *l* ≤ 29
reflns collected	35518	26293	26168	77174
indep data, param^a^	5650, 339	7084, 339	7003, 339	21625, 909
GOF^b^	1.063	1.074	1.006	1.071
R1,^c^^,^^d^ wR2^d^^,^^e^	0.0340, 0.0918	0.0408, 0.1122	0.0362, 0.0836	0.0379, 0.1019
R1,^c^^,^^f^ wR2^e^^,^^f^	0.0376, 0.0952	0.0558, 0.1179	0.0487, 0.0870	0.0433, 0.1044

a Independent data collected and parameters refined.

b GOF = {∑[*w*(*F*_o_^2^ – *F*_c_^2^)^2^]/(*n* – *p*)}^1/2^, where *n* = number of reflections and *p* is the total number of parameters refined.

c R1 = ∑||*F*_o_| – |*F*_c_||/∑|*F*_o_|.

d *R* indices for data cut off at *I* > 2σ(*I*).

e wR2 = {∑[*w*(*F*_o_^2^ – *F*_c_^2^)^2^]/∑[*w*(F_o_^2^)^2^]}^1/2^; *w* = 1/[σ^2^(*F*_o_^2^) + (*xP*)^2^ + *yP*], where *P* = [2*F*_c_^2^ + Max(*F*_o_^2^,0)]/3.

f *R* indices for all data.

### [Cl_2_Ni(tpbz)] (**1**)

A 50 mL Schlenk flask charged with [Cl_2_Ni(dme)] (0.022 g, 0.10 mmol), tpbz (0.081 g, 0.10 mmol), and CH_2_Cl_2_ (15 mL) was stirred for 1 h at ambient temperature, during which time a dark-red color developed. The reaction mixture was filtered to remove undissolved materials, and the filtrate was taken to dryness under reduced pressure to afford an orange-red solid residual that was suitable for use without further purification. Yield: 0.075 g, 0.079 mmol, 79%. ^1^H NMR (CD_2_Cl_2_): δ 9.63–9.51 (m, 11H, aromatic C–*H*), 9.34–9.15 (m, 14H, aromatic C–*H*), 8.98–8.78 (m, 17H, aromatic C–*H*). ^31^P{^1^H} NMR (CD_2_Cl_2_): δ 49.9 (s), −14.1 (s).

### [Cl_2_Pd(tpbz)] (**2**)

A 50 mL Schlenk flask was charged with [PdCl_2_(CH_3_CN)_2_] (0.026 g, 0.10 mmol), tpbz (0.081 g, 0.10 mmol), and a 1:1 mixture of CH_2_Cl_2_/CH_3_CN (20 mL). The resulting mixture was stirred at ambient temperature for 12 h in the dark, during which time a light-yellow solution developed. The solvent was removed under reduced pressure, and the residual light-yellow solid was washed with Et_2_O (2 × 5 mL) and dried in vacuo. This compound shows only limited solubility in common organic solvents. Yield: 0.084 g, 0.085 mmol, 85%. ^1^H NMR (DMSO-*d*_6_): δ 7.63–7.52 (m, 14H, aromatic C–*H*), 7.35–7.16 (m, 14H aromatic C–*H*), 6.94–6.79 (m, 14H, aromatic C–*H*). ^31^P{^1^H} NMR (DMSO-*d*_6_): δ 49.5 (s), −14.4 (s).

### [Cl_2_Pt(tpbz)] (**3**)

The same procedure and scale as those described for the synthesis of **2** were implemented using [PtCl_2_(CH_3_CN)_2_], which yielded **3** as a white solid. Yield: 0.083 g, 0.077 mmol, 77%. ^1^H NMR (DMSO-*d*_6_): δ 7.81–7.74 (m, 12H, aromatic C–*H*), 7.64–7.56 (m, 22H, aromatic C–*H*), 7.30–7.22 (m, 8H, aromatic C–*H*). ^31^P{^1^H} NMR (DMSO-*d*_6_): δ 39.1 (s), −14.1 (s).

### [(mnt)Ni(tpbz)] (**4**)

Under an atmosphere of N_2_, a 50 mL Schlenk flask with a stirbar was charged with **1** (0.088 g, 0.093 mmol) and 20 mL of CH_2_Cl_2_. Under an outward flow of N_2_, solid [(mnt)SnMe_2_] (0.029 g, 0.10 mmol) was added to the flask, and the resulting mixture was stirred at ambient temperature for 12 h, during which time a reddish-brown color developed. The solvent was removed under reduced pressure, and the reddish crude solid was then triturated with MeOH (2 × 5 mL), followed by Et_2_O (2 × 5 mL), and dried under vacuum. This crude material was further purified on a silica column eluted with CH_2_Cl_2_ and collected as a red-brown band. Recrystallization was accomplished by the diffusion of MeOH into a filtered CHCl_3_ solution. Yield: 0.070 g, 74%. *R*_*f*_ = 0.17 (9:1 CH_2_Cl_2_/hexanes). ^1^H NMR (CDCl_3_): δ 7.37–7.32 (m, 4H, aromatic C–*H*), 7.22–7.12 (m, 21H, aromatic C–*H*), 7.06–6.97 (m, 9H, aromatic C–*H*), 6.90–6.85 (m, 8H, aromatic C–*H*). ^31^P{^1^H} NMR (CDCl_3_): δ 57.2 (s), −14.5 (s). UV–vis [CH_2_Cl_2_; λ_max_, nm (ε, M^–1^ cm^–1^)]: 366 (4960). IR (KBr, cm^–1^): 2217 (ν_C=N_, symm), 2204 (ν_C≡N_, asymm). MS (ESI^+^). Calcd for monoisotopic [C_58_H_42_N_2_NiP_4_S_2_]^+^: *m*/*z* 1012.1093. Obsd: *m*/*z* 1012.1041. Error (δ): 5.2 ppm.

### [(Me_2_C_2_S_2_)Ni(tpbz)] (**5**)

A 50 mL Schlenk flask with a stirbar was charged with **1** (0.088 g, 0.093 mmol) and 20 mL of CH_2_Cl_2_. Under an outward flow of N_2_, solid [(Me_2_C_2_S_2_)Sn(^*n*^Bu)_2_] (0.0351 g, 0.10 mmol) was added to the flask, which immediately induced a dark-green-brown color. The resulting mixture was stirred at ambient temperature for 12 h. The solvent was removed under reduced pressure, and the solid residual was triturated with MeOH (2 × 5 mL), followed by Et_2_O (2 × 5 mL), and then dried under vacuum. This material was purified on a silica chromatography column eluted with CH_2_Cl_2_/hexanes (9:1) and collected as a green band. Recrystallization was accomplished by the diffusion of *n*-pentane or Et_2_O into a filtered CH_2_Cl_2_ solution. Yield: 0.044 g, 48%. *R*_*f*_ = 0.32 (9:1 CH_2_Cl_2_/hexanes). ^1^H NMR (CDCl_3_): δ 7.37–7.24 (overlapping m, 12H, aromatic C–*H*), 7.15–7.10 (m, 12H, aromatic C–*H*), 7.03–6.95 (m, 10H, aromatic C–*H*), 6.88–6.84 (m, 8H, aromatic C–*H*), 2.00 (s, 6H, −C*H*_3_). ^31^P{^1^H} NMR (CDCl_3_): δ 55.1 (s), −14.9 (s). UV–vis [CH_2_Cl_2_; λ_max_, nm (ε, M^–1^ cm^–1^)]: 444 (1440), 638 (160). MS (ESI^+^). Calcd for monoisotopic [C_58_H_48_NiP_4_S_2_]^+^: *m*/*z* 990.1501. Obsd: *m*/*z* 990.1509. Error (δ): 0.76 ppm.

### [(Me_2_C_2_S_2_)Pd(tpbz)] (**6**)

The same procedure and scale as those described for the synthesis of **4** were implemented but with **2** used in place of the corresponding Ni compound. Purification was accomplished by a chromatography column eluted with CH_2_Cl_2_/hexanes (3:1), with **5** collected as a yellow band. Yield: 0.032 g, 33%. *R*_*f*_ = 0.20 (9:1 CH_2_Cl_2_/hexanes). ^1^H NMR (CDCl_3_): δ 7.35–7.25 (m, 12H, aromatic C–*H*), 7.18–7.12 (m, 14H, aromatic C–*H*), 7.06–7.01 (m, 8H, aromatic C–*H*), 6.89–6.84 (m, 8H, aromatic C–H), 2.01 (s, 6H, −C*H*_3_). ^31^P{^1^H} NMR (CDCl_3_): δ 49.3 (s), −14.7 (s). UV–vis [CH_2_Cl_2_; λ_max_, nm (ε, M^–1^ cm^–1^)]: 427 (1450), 588 (360). MS (ESI^+^). Calcd for monoisotopic [C_58_H_48_P_4_PdS_2_]^+^: *m*/*z* 1038.1201. Obsd: *m*/*z* 1038.1159. Error (δ): 4.11 ppm.

### [(Me_2_C_2_S_2_)Pt(tpbz)] (**7**)

The same procedure and scale as those described for the synthesis of **4** were implemented but with **3** used in place of the corresponding Ni compound. Purification was accomplished using a silica column chromatography eluted with CH_2_Cl_2_/hexanes (2:1); **6** was collected as a yellow band. Yield: 0.041 g, 39%. *R*_*f*_ = 0.35 (9:1 CH_2_Cl_2_/hexanes). ^1^H NMR (CDCl_3_): δ 7.39–7.33 (m, 8H, aromatic C–*H*), 7.29–7.24 (m, 4H, aromatic C–*H*), 7.19–7.13 (m, 14H, aromatic C–*H*), 7.06–7.01 (m, 8H, aromatic C–*H*), 6.90–6.85 (m, 8H, aromatic C–*H*), 2.07 (s, 6H, −C*H*_3_). ^31^P{^1^H} NMR (CDCl_3_): δ 44.2 (s, *J*_Pt–P_= 2754 Hz), −14.8 (s). UV–vis [CH_2_Cl_2_; λ_max_, nm (ε, M^–1^ cm^–1^)]: 418 (3910). MS (ESI^+^). Calcd for monoisotopic [C_58_H_48_P_4_PtS_2_]^+^: *m*/*z* 1128.1811. Obsd: *m*/*z* 1128.1731. Error (δ): 7.11 ppm.

### [(Ph_2_C_2_S_2_)Ni(tpbz)] (**8**)

A 50 mL Schlenk flask with a stirbar was charged with tpbz (0.081 g, 0.099 mmol) and 20 mL of CH_2_Cl_2_. Under an outward flow of N_2_, [Ni(S_2_C_2_Ph_2_)_2_] (0.059 g, 0.11 mmol) was added, which quickly induced the formation of a dark-green color. The resulting mixture was stirred for 4 h, after which time the solvent was removed under reduced pressure. The solid residual was purified on a silica column eluted with 2:1 CH_2_Cl_2_/hexanes, and **8** was isolated as a green band. Following removal of the solvent under reduced pressure, recrystallization of **8** was accomplished by the diffusion of Et_2_O vapor into a filtered CH_2_Cl_2_ or ClCH_2_CH_2_Cl solution. Continued elution of the column with 10:1 CH_2_Cl_2_/THF led to a brown band of [(pdt)Ni(tpbz)Ni(pdt)], the properties of which were reported earlier.^2^ Yield: 0.059 g, 0.053 mmol, 53%. *R*_*f*_ = 0.82 (9:1 CH_2_Cl_2_/hexanes). ^1^H NMR (CDCl_3_): δ 7.48–7.42 (m, 8H, aromatic C–*H*), 7.38–7.33 (m, 4H, aromatic C–*H*), 7.24–7.20 (m, 16H, aromatic C–*H*), 7.13–7.08 (m, 10H, aromatic C–*H*), 7.04–7.00 (m, 5H, aromatic C–*H*), 6.97–6.92 (m, 9H, aromatic C–*H*). ^31^P{^1^H} NMR (CDCl_3_): δ 55.0 (s), −14.8 (s). UV–vis [CH_2_Cl_2_; λ_max_, nm (ε, M^–1^ cm^–1^)]: 416 (3680), 616 (560). MS (ESI^+^). Calcd for monoisotopic [C_68_H_52_NiP_4_S_2_]^+^: *m*/*z* 1114.1814. Obsd: *m*/*z* 1114.178. Error (δ): 3.08 ppm. Anal. Calcd for **8** (C_68_H_52_NiP_4_S_2_): C, 73.19; H, 4.70; P, 11.10. Found: C, 72.99; H, 4.78; P, 11.24.

### [(Ph_2_C_2_S_2_)Pd(tpbz)] (**9**)

The same procedure and scale as those described for **8** were implemented, with the only differences being the onset of a reddish-brown color during the reaction and elution (with 2:1 CH_2_Cl_2_/hexanes) of the product as a dark-red-purple band from the silica column. Recrystallization was accomplished by the diffusion of Et_2_O into a filtered CH_2_Cl_2_ solution. Continued elution of the column with 10:1 CH_2_Cl_2_.THF rapidly led to a dark-reddish-orange band of [(pdt)Pd(tpbz)Pd(pdt)], which has been previously described.^2^ Yield: 0.060 g, 0.052 mmol, 52%. *R*_*f*_ = 0.73 (9:1 CH_2_Cl_2_/hexanes). ^1^H NMR (CDCl_3_): δ 7.45–7.34 (m, 11H, aromatic C–*H*), 7.26–7.22 (m, 20H, aromatic C–*H*), 7.13–7.08 (m, 8H, aromatic C–*H*), 7.04–6.92 (m, 13H, aromatic C–*H*). ^31^P{^1^H} NMR (CDCl_3_): δ 49.4 (s), −14.7 (s). UV–vis [CH_2_Cl_2_; λ_max_, nm (ε, M^–1^ cm^–1^)]: 420 (2670), 544 (860). MS (ESI^+^). Calcd for monoisotopic [C_68_H_52_P_4_PdS_2_]^+^: *m*/*z* 1162.1517. Obsd: *m*/*z* 1162.1485. Error (δ): 2.77 ppm. Anal. Calcd for **9** (C_68_H_52_PdP_4_S_2_): C, 70.19; H, 4.50; P, 10.65. Found: C, 69.57; H, 4.17; P, 9.83.

### [(Ph_2_C_2_S_2_)Pt(tpbz)] (**11**)

Under an atmosphere of N_2_, a 50 mL Schlenk flask with a stirbar was charged with tpbz (0.100 g, 0.123 mmol) and CH_2_Cl_2_ (20 mL). Under an outward flow of N_2_, [Pt(S_2_C_2_Ph_2_)_2_] (0.0834 g, 0.123 mmol) was added, which immediately induced the formation of a blue color. Stirring was continued for 2 h, and the reaction mixture was then kept for 3 days without stirring. The solvent was removed under reduced pressure, and the crude solid residue was purified on a silica column that was flash-eluted with 1:1 CH_2_Cl_2_/hexanes. The title compound was collected as the leading yellow band. Crystallization of **11** as yellow columns was accomplished by the diffusion of Et_2_O vapor into a filtered CH_2_Cl_2_ solution. Yield: 0.057 g, 37%. *R*_*f*_ = 0.77 (9:1 CH_2_Cl_2_/hexanes). ^1^H NMR (CDCl_3_): δ 7.60–7.51 (m, 4H, aromatic C–*H*), 7.39–7.36 (m, 10H, aromatic C–*H*), 7.28–7.26 (m, 8H, aromatic C–*H*), 7.06–7.01 (m, 14H, aromatic C–*H*), 6.96–6.89 (m, 16H, aromatic C–*H*). ^31^P{^1^H} NMR (CDCl_3_): δ 42.7 (s, *J*_Pt–P_ = 2742 Hz), −14.9 (s). UV–vis [CH_2_Cl_2_; λ_max_, nm (ε, M^–1^ cm^–1^)]: 332 (8750), 399 (2750). MS (ESI^+^). Calcd for monoisotopic [C_68_H_52_P_4_PtS_2_]^+^: *m*/*z* 1252.2126. Obsd: *m*/*z* 1252.2067. Error (δ): 4.72 ppm. Anal. Calcd for **11** (C_68_H_52_PtP_4_S_2_): C, 65.22; H, 4.19. Found: C, 65.22; H, 4.13.

### [(Ph_2_C_2_S_2_)_2_Pt(tpbz)] (**10**)

Continued elution of the column used for the isolation of compound **11** with 2:1 CH_2_Cl_2_/hexanes moved **10** as a blue band, which was collected and reduced to dryness. Crystallization of **10** as blue blocks was achieved by the diffusion of Et_2_O vapor into a filtered CH_2_Cl_2_ solution. Yield: 0.017 g, 9%. *R*_*f*_ = 0.23 (9:1 CH_2_Cl_2_/hexanes), ^1^H NMR (CD_2_Cl_2_): δ 7.64–7.48 (m, 8H, aromatic C–*H*), 7.40–7.19 (m, 16H, aromatic C–*H*), 7.11–7.03 (m, 12H, aromatic C–*H*), 6.96–6.93 (m, 11H, aromatic C–*H*), 6.92–6.84 (m, 9H, aromatic C–*H*), 6.49–6.45 (m, 16H, aromatic C–*H*). ^31^P{^1^H} NMR (CD_2_Cl_2_): δ 19.0 (s, *J*_Pt–P_= 1787 Hz), −13.3 (s). UV–vis [CH_2_Cl_2_; λ_max_, nm (ε, M^–1^ cm^–1^)]: 583 (2140), 355 (11500). MS (ESI^+^). Calcd for monoisotopic [C_82_H_62_P_4_PtS_4_]^+^: *m*/*z* 1494.2349. Obsd: *m*/*z* 1494.229. Error (δ): 3.95 ppm.

### [((MeO-*p*-C_6_H_4_)_2_C_2_S_2_)Ni(tpbz)] (**12**)

The same procedure and scale as those described for the synthesis of **8** were implemented but with [((MeO-*p*-C_6_H_4_)_2_C_2_S_2_)_2_Ni] used in place of [(Ph_2_C_2_S_2_)_2_Ni]. Crystals were grown by the diffusion of hexanes vapor into a filtered chlorobenzene solution. Continued elution of the silica column used to purify **12** using 10:1 CH_2_Cl_2_/THF rapidly moved a brown band of [((MeO-*p*-C_6_H_4_)_2_C_2_S_2_)Ni(tpbz)Ni(S_2_C_2_(C_6_H_4_-*p*-OMe)_2_)], which has been previously described.^2^ Yield: 0.057 g of a dark-green solid, 49%. *R*_*f*_ = 0.42 (9:1 CH_2_Cl_2_/hexanes). ^1^H NMR (CDCl_3_): δ 7.50–7.35 (m, 14H, aromatic C–*H*), 7.26–7.19 (m, 14H, aromatic C–*H*), 7.16–7.10 (m, 11H, aromatic C–*H*), 6.99–6.94 (m, 7H, aromatic C–*H*), 6.63–6.60 (m, 4H, aromatic C–*H*), 3.71 (s, 6H, −O*Me*). ^31^P{^1^H} NMR (CDCl_3_): δ 55.2 (s), −14.2 (s). UV–vis [CH_2_Cl_2_; λ_max_, nm (ε, M^–1^ cm^–1^)]: 432 (2860), 620 (610). MS (ESI^+^). Calcd for monoisotopic [C_70_H_56_NiO_2_P_4_S_2_]^+^: *m*/*z* 1174.2026. Obsd: *m*/*z* 1174.1936. Error (δ): 7.67 ppm. Anal. Calcd for **12** ([C_70_H_56_NiO_2_P_4_S_2_]): C, 71.50; H, 4.80; P, 10.54; S, 5.45. Found: C, 71.29; H, 4.86; P, 10.41; S, 5.51.

### [((MeO-*p*-C_6_H_4_)_2_C_2_S_2_)Pd(tpbz)] (**13**)

The same procedure and scale as those described for the synthesis of **8** were implemented but with [((MeO-*p*-C_6_H_4_)_2_C_2_S_2_)_2_Pd] used in place of [(Ph_2_C_2_S_2_)_2_Ni]. The crude solid residue was purified on a silica column that was flash-eluted with 2:1 CH_2_Cl_2_/hexanes; **13** was collected as the leading red-purple band. Continued elution with 10:1 CH_2_Cl_2_/THF rapidly led to a brown-purple band of [((MeO-*p*-C_6_H_4_)_2_C_2_S_2_)Pd(tpbz)Pd(S_2_C_2_(C_6_H_4_-*p*-OMe)_2_)], which has been previously described.^2^ Yield: 0.063 g of a brown-red solid, 52%. *R*_*f*_ = 0.23 (9:1 CH_2_Cl_2_/hexanes). ^1^H NMR (CDCl_3_): δ 7.47–7.35 (m, 13H, aromatic C–*H*), 7.28–7.21 (m, 16H, aromatic C–*H*), 7.17–7.10 (m, 10H, aromatic C–*H*), 6.99–6.94 (m, 7H, aromatic C–*H*), 6.60–6.30 (m, 4H, aromatic C–*H*), 3.70 (s, 6H, −O*Me*). ^31^P{^1^H} NMR (CDCl_3_): δ 49.3 (s), −14.7 (s). UV–vis [CH_2_Cl_2_; λ_max_, nm (ε, M^–1^ cm^–1^)]: 432 (2180), 551 (930). MS (ESI^+^). Calcd for monoisotopic [C_70_H_56_O_2_P_4_PdS_2_]^+^: *m*/*z* 1221.1729. Obsd: *m*/*z* 1221.1652. Error (δ): 6.23 ppm.

### [((MeO-*p*-C_6_H_4_)_2_C_2_S_2_)Pt(tpbz)] (**14**)

The same procedure and scale as those described for the synthesis of **8** were implemented but with [((MeO-*p*-C_6_H_4_)_2_C_2_S_2_)_2_Pd] used in place of [(Ph_2_C_2_S_2_)_2_Ni]. The crude solid residue was purified on a silica column eluted with 2:1 CH_2_Cl_2_/hexanes, which led to the title compound as the leading yellow band. Continued elution with 10:1 CH_2_Cl_2_/THF rapidly led to a red band of [((MeO-*p*-C_6_H_4_)_2_C_2_S_2_)Pt(tpbz)Pt(S_2_C_2_(C_6_H_4_-*p*-OMe)_2_)]. Yield: 0.046 g of a bright-yellow solid, 35%. *R*_*f*_ = 0.34 (9:1 CH_2_Cl_2_/hexanes). ^1^H NMR (CDCl_3_): δ 7.41–7.35 (m, 8H, aromatic C–*H*), 7.29–7.25 (m, 6H, aromatic C–*H*), 7.19–7.10 (m, 16H, aromatic C–*H*), 7.09–7.01 (m, 10H, aromatic C–*H*), 6.90–6.85 (m, 8H, aromatic C–*H*), 3.61 (s, 6H, – O*Me*). ^31^P{^1^H} NMR (CDCl_3_): δ 43.8 (s, *J*_Pt–P_ = 2742 Hz), −14.8 (s). UV–vis [CH_2_Cl_2_; λ_max_, nm (ε, M^–1^ cm^–1^)]: 414 (4140). MS (ESI^+^). Calcd for monoisotopic [C_70_H_56_O_2_P_4_PtS_2_]^+^: *m*/*z* 1312.2338. Obsd: *m*/*z* 1312.2294. Error (δ): 3.33 ppm.

### [(Ph_2_C_2_S_2_)Pt(tpbz)Ni(S_2_C_2_Me_2_)] (**15**)

A 50 mL Schlenk flask was charged with [Cl_2_Ni(dme)] (0.022 g, 0.1 mmol) and **11** (0.125 g, 0.1 mmol) under a N_2_ atmosphere. To this mixture of solids was added CH_2_Cl_2_ (20 mL) via a syringe, and the resulting solution was stirred at room temperature for 1 h. During this time, a red color appeared. To the same reaction mixture was added [(Me_2_C_2_S_2_)Sn^*n*^Bu_2_] (0.035 g, 0.1 mmol), and a deep-red color was generated. The reaction mixture was stirred at ambient temperature overnight (12 h), after which time the solvent was removed under reduced pressure. The resulting dark-red solid residue was triturated with stirring under MeOH (8 mL). This MeOH washing was removed by filter cannulation, and the residue was washed again with MeOH (5 mL), followed by Et_2_O (2 × 5 mL). Crude **15** was purified on a silica column eluted with CH_2_Cl_2_. The red band of **15**, following reduction to dryness, was crystallized from ClCH_2_CH_2_Cl/^*t*^BuOMe. Yield: 0.051 g of a red solid, 36%. *R*_*f*_ = 0.18 (CH_2_Cl_2_). ^1^H NMR (CDCl_3_): δ 7.47–7.41 (m, 18H, aromatic C–*H*), 7.37–7.30 (m, 9H, aromatic C–*H*), 7.24–7.21 (m, 9H, aromatic C–*H*), 7.17–7.13 (m, 11H, aromatic C–*H*), 6.99–6.96 (m, 5H, 2H, aromatic C–*H*), 2.01 (s, 6H, −C*H*_3_). ^31^P{^1^H} NMR (CDCl_3_): δ 53.1 (s), 42.3 (s, *J*_Pt–P_ = 2734 Hz). UV–vis [CH_2_Cl_2_; λ_max_, nm (ε, M^–1^ cm^–1^)]: 466 (3940), 733 (1030). MS (ESI^+^). Calcd for monoisotopic [C_72_H_58_NiP_4_PtS_4_]^+^: *m*/*z* 1428.1375. Obsd: *m*/*z* 1428.1367. Error (δ): 0.56 ppm.

### [(Ph_2_C_2_S_2_)Ni(tpbzO_2_)] (**16**)

In the open air, a solution of **8** (0.111 g, 0.1 mmol) in 15 mL of CHCl_3_ was treated with a solution of I_2_ (0.051 g, 0.2 mmol) in CHCl_3_ (5 mL). The reaction mixture was stirred overnight (14 h) at ambient temperature. The brownish solution was transferred to a separatory funnel and washed one time with aqueous 1 M NaOH (20 mL). The organic phase was separated as a green solution from the aqueous phase and taken to dryness under reduced pressure to afford crude **16** as a dark-green solid. Further purification was performed by eluting a slurry of the crude mixture with 20:80 THF/CHCl_3_ from a silica column; **16** led to a green band and was collected and recrystallized from CH_2_Cl_2_/Et_2_O. Yield: 0.044 g of a green solid, 39%. *R*_*f*_ = 0.18 (8:2 CH_2_Cl_2_/THF). ^1^H NMR (CDCl_3_): δ 7.42–7.36 (m, 10H, aromatic C–*H*), 7.33–7.28 (m, 10H, aromatic C–*H*), 7.26–7.21 (m, 14H, aromatic C–*H*), 7.16–7.12 (m, 13H, aromatic C–*H*), 6.98–6.94 (m, 5H, aromatic C–*H*). ^31^P{^1^H} NMR (CDCl_3_): δ 55.8 (s), 29.1 (s). UV–vis [CH_2_Cl_2_; λ_max_, nm (ε, M^–1^ cm^–1^)]: 432 (2340), 630 (660). IR (CH_2_Cl_2_, cm^–1^): 1245 (vs, ν_P=O_). MS (ESI^+^). Calcd for monoisotopic [C_68_H_52_NiO_2_P_4_S_2_]^+^: *m*/*z* 1146.1713. Obsd: *m*/*z* 1146.1743. Error (δ): 2.62 ppm.

### [(Me_2_C_2_S_2_)Pt(tpbzO_2_)] (**17**)

In a 25 mL Schlenk tube, [((CH_3_)_2_C_2_S_2_)Pt(tpbz)] (0.0601 g, 0.0533 mmol) was dissolved in CH_2_Cl_2_ (3 mL) under N_2_. To this solution was added H_2_O_2_ (0.02 mL, 30% in H_2_O, 0.2 mmol), and the mixture was stirred at room temperature overnight. The solvent and all volatiles were removed under reduced pressure, and the solid residual was redissolved in a minimal volume of CH_2_Cl_2_. To this CH_2_Cl_2_ solution were added hexanes (15 mL) to induce precipitation of an orange solid, which was then isolated by filtration and washed with hexanes (3 × 5 mL). Recrystallization of **17** as orange needles was accomplished by the diffusion of Et_2_O vapor into a CH_2_Cl_2_ solution. Yield: 0.0245 g, 40%. ^1^H NMR (CDCl_3_): δ 7.47–7.21 (m, 42H, aromatic C–*H*), 2.15 (s, 6H, C*H*_3_). ^31^P{^1^H} NMR (CDCl_3_): δ 45.1 (s, *P*Ph_2_Pt, *J*_P–Pt_ = 2755 Hz), 29.0 (s, *P*Ph_2_O). MS (ESI^+^). Calcd for monoisotopic [C_58_H_48_O_2_P_4_PtS_2_]^1+^: *m*/*z* 1160.1604. Obsd: *m*/*z* 1160.1709. Error (δ): 9.05 ppm.

### [(Me_2_C_2_S_2_)Ni(tpbzS_2_)] (**18**)

A solution of [((CH_3_)_2_C_2_S_2_)Ni(tpbz)] (0.0477 g, 0.048 mmol) in THF (3.6 mL) was treated with [NH_4_]_2_S (0.60 mL, 20% in H_2_O) and then allowed to stir at ∼50 °C for 3 days. The mixture was reduced to dryness under reduced pressure, redissolved in a minimal volume of CHCl_3_, and eluted from a silica column packed as a slurry in CHCl_3_. The leading green-brown band was collected and reduced to dryness, yielding **18** as a brown solid. Yield: 0.0223 g, 38%. ^1^H NMR (CDCl_3_): δ 7.51–7.30 (m, 26H, aromatic C–*H*), 7.30–7.16 (m, 16H, aromatic C–*H*), 2.06 (s, 6H, −C*H*_3_). ^31^P NMR (CDCl_3_): δ 55.9 (s, −Ph_2_*P*Ni), 46.9 (s, −Ph_2_*P*S). UV–vis [CH_2_Cl_2_; λ_max_, nm (ε, M^–1^ cm^–1^)]: 448 (2270). MS (ESI^+^). Calcd for monoisotopic [C_58_H_48_NiP_4_S_4_]^+^: *m*/*z* 1054.0943. Obsd: *m*/*z* 1054.0951. Error (δ): 0.82 ppm. Anal. Calcd for **18** ([C_58_H_48_NiP_4_S_4_]): C, 65.98; H, 4.58; P, 11.73. Found: C, 65.46; H, 4.44; P, 10.7.

### [Ni(dppbO_2_)_3_][I_3_]_2_ ([**19**][I_3_]_2_)

Under an atmosphere of N_2_, a 50 mL Schlenk flask with a stirbar was charged with Ni(NO_3_)_2_ (0.013 g, 0.039 mmol) and THF (20 mL). Under an outward flow of N_2_, solid dppbO_2_ (0.056 mg, 0.117 mmol) was added in a single portion, which induced the development of a very light-green solution. The reaction mixture was stirred overnight (14 h) at ambient temperature. Cesium triiodide (0.040 g, 0.078 mmol) was added under an outward flow of N_2_, followed by the addition of MeOH (2 mL). The resulting red-brown solution was stirred for 5 h at ambient temperature. Under reduced pressure, the solution was concentrated to a volume of ∼3 mL, whereupon Et_2_O (10 mL) was added to precipitate the crude product as a red-brown powder. The solvent was removed by cannula filtration, and the dark red-brown solid residue was washed with Et_2_O (3 × 5 mL) and dried under vacuum. Diffraction-quality red-orange plate crystals were grown by diffusion of Et_2_O into a filtered concentrate of the complex in CH_2_Cl_2_. Yield: 0.076 g, 87%. UV–vis [CH_2_Cl_2_; λ_max_, nm (ε, M^–1^ cm^–1^)]: 426 (8920). Solution IR (CH_2_Cl_2_, cm^–1^): 1264 (vs, ν_P=O_). MS (ESI^+^). Calcd for monoisotopic [C_90_H_72_NiP_6_S_6_]^+^: *m*/*z* 1492.3108. Calcd for [C_90_H_72_NiP_6_S_6_]^2+^: *m*/*z* 746.1554. Obsd: *m*/*z* 746.1566. Error (δ): 1.65 ppm.

## Discussion

### Syntheses and Structures

Charge-neutral homoleptic bis(dithiolene) complexes of the Group 10 metals [M(S_2_C_2_R_2_)_2_] (M = Ni^2+^, Pd^2+^, Pt^2+^) are subject to direct displacement of one ligand by soft σ donors such as phosphines or isocyanides. When the tpbz ligand is introduced to [M(S_2_C_2_R_2_)_2_] in a 1:1 ratio, the open-ended [[(R_2_C_2_S_2_)M(tpbz)] compounds are efficiently generated in yields ranging from ∼30 to 50% (Scheme 2, Method 2). Purification by column chromatography and crystallization from dry solvents by vapor diffusion methods are straightforward. The Pt complexes proceed through an octahedral bis(dithiolene)diphosphine intermediate that is isolable in minor amounts (cf. **10**, Scheme 2). Because the cyano-substituted dithiolene ligand does not support stable [M(S_2_C_2_(CN)_2_)_2_]^0^ complexes that can extrude a dithiolene ligand, the [((NC)_2_C_2_S_2_)M(tpbz)] complexes must be prepared by the alternate route of transmetalation using a dialkyltin dithiolene complex (Scheme 2, Method 1). Method 1 has demonstrated usefulness in chloride-for-dithiolene ligand exchange for a broad variety of dithiolene ligand types.^2,12−18^ The open-ended [Cl_2_M(tpbz)] compounds (**1**–**3**) with which these transmetalation reactions occur are themselves new compounds and are readily obtained in ∼80–90% yields by the introduction of tpbz to [Cl_2_Ni(dme)] or [Cl_2_M(N≡CCH_3_)_2_] (M = Pd, Pt). All open-ended tpbz compounds are spectroscopically distinct by ^31^P NMR from the symmetric, dimetallic compounds because they display a signal upfield of the H_3_PO_4_ reference, which is ascribed to the open end, and a signal ∼50 ppm downfield of the reference, which arises from the P atoms chelated to M^2+^.

**Figure 2:**
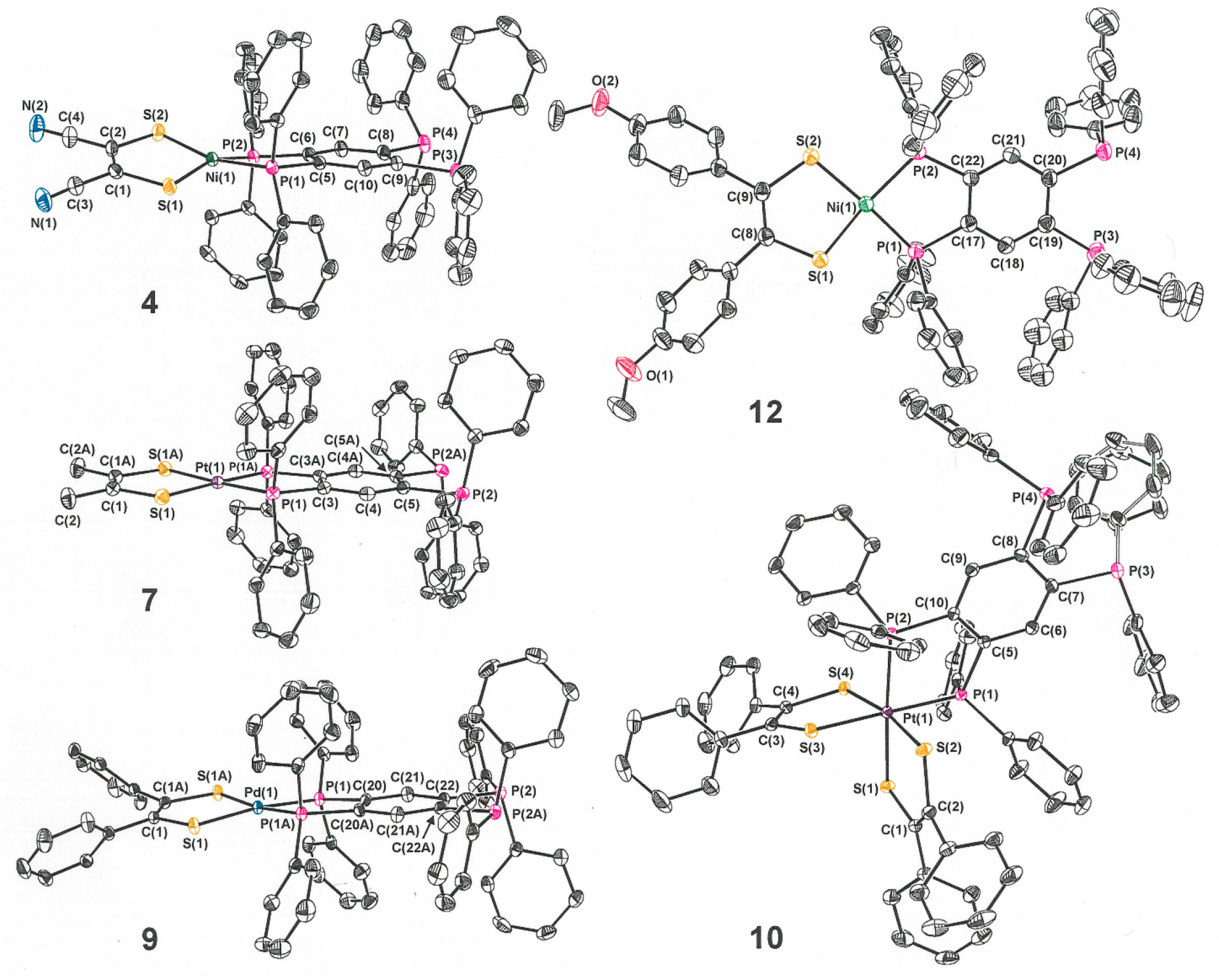
Thermal ellipsoid plots (50% probability level) of selected open-ended tpbz compounds that have been characterized by X-ray crystallography. All H atoms have been omitted for clarity.

Representative members of the set of new compounds summarized in Scheme 2 have been characterized by X-ray crystallography (Figure 2 and Tables 1 and 2). In contrast to the dimetallic compounds [(R_2_C_2_S_2_)M(tpbz)M(S_2_C_2_R_2_)], which show a proclivity to crystallize upon an inversion center in monoclinic *P*2_1_/*c* (No. 14), the structures of the compounds in Scheme 2 reveal a tendency to coincide with *C*_2_ axes in monoclinic *C*2/*c* (No. 15). As is typical for the d^8^ configuration, compounds **1**–**5**, **7**–**10**, and **11** display structures that are best described as square-planar, albeit with minor tetrahedral distortions. Quantified by the angle θ between the S–M–S and P–M–P planes, these departures from ideal planarity are more pronounced for the Ni complexes than for the heavier metal complexes (Table 3). A longer M−P then M−S bond length is observed in all Ni compounds, while the inverse is true is for the Pd and Pt complexes. This same pattern is clearly evident in the [(R_2_C_2_S_2_)M(tpbz)M(S_2_C_2_R_2_)] compounds and has been attributed to more effective metal d – sulfur p σ overlap in the HOMO–3 and HOMO–4 for Ni versus Pd and Pt.^2^ Undoubtedly, this explanation is equally pertinent to the open-ended compounds. The P–C bond lengths to the central arene ring of the tpbz ligand differ notably between the bound and open ends of the ligand. A consistently longer P–C bond, by ∼0.02 Å, is found at the open end.

**Table 2:** Unit Cell and Refinement Data for Compounds Characterized by X-ray Diffraction

compound	[(adt)Ni(tpbz)]	[(pdt)Pt(tpbz)Ni(mdt)]	[(pdt)Ni(tpbzO_2_)]	[(mdt)Pt(tpbzO_2_)]
compound no.	**12**	**15**	**16**	**17**
cocryst solvent	none	2^1^/_2_(ClCH_2_CH_2_Cl)	CH_2_Cl_2_	none
formula	C_70_H_56_NiO_2_P_4_S_2_	C_77_H_68_Cl_5_NiP_4_PtS_4_	C_69_H_54_Cl_2_NiO_2_P_4_S_2_	C_58_H_48_O_2_P_4_PtS_2_
fw, g mol^–1^	1175.85	1676.48	1232.73	1160.05
temperature, K	150	150	150	150
wavelength, Å	1.54178	0.71073	0.71073	0.71073
2θ range, deg	4.59–144.21	3.87–66.84	3.57–61.23	4.18–66.45
cryst syst	monoclinic	triclinic	triclinic	monoclinic
space group	*P*2_1_/*n*	*P*1̅	*P*1̅	*P*2_1_/*c*
*a*, Å	12.0422(4)	12.5374(6)	13.4505(18)	17.0751(11)
*b*, Å	38.5014(13)	12.5692(6)	16.597(2)	16.0625(10)
*c*, Å	14.0003(6)	24.7673(13)	16.653(2)	20.5241(13)
α, deg	90	92.953(2)	64.068(4)	90
β, deg	112.798(2)	91.560(2)	70.817(4)	108.283(2)
γ, deg	90	108.166(2)	72.092(4)	90
volume (Å^3^), *Z*	5984.0(4), 4	3699.6(3), 2	3098.4(7), 2	5345.0(6), 4
density, g cm^–3^	1.305	1.505	1.321	1.442
μ, mm^–1^	2.484	2.565	0.615	2.864
color, habit	yellow-green plate	orange plate	orange plate	dark-orange column
limiting indices *h*	–14 ≤ *h* ≤ 14	–19 < *h* < 19	–19 < *h* < 19	–26 ≤ *h* ≤ 26
limiting indices *k*	–44 ≤ *k* ≤ 46	–19 < *k* < 19	–23 < *k* < 23	–24 ≤ *k* ≤ 24
limiting indices *l*	–16 ≤ *l* ≤ 14	0 < *l* < 38	–23 < *l* < 23	–31 ≤ *l* ≤ 31
reflns collected	51648	32993	144627	403235
indep data, param^a^	11399, 703	32993, 856	19027, 728	20472, 606
GOF^b^	1.039	1.277	1.044	1.081
R1,^c^^,^^d^ wR2^d^^,^^e^	0.0479, 0.1008	0.0751, 0.1691	0.0488, 0.1165	0.0239, 0.0594
R1,^c^^,^^f^ wR2^e^^,^^f^	0.0699, 0.1106	0.0827, 0.1717	0.0878, 0.1350	0.0314, 0.0641

compound	[(mdt)Ni(tpbzS_2_)]	[(dppbO_2_)_3_Ni][I_3_]_2_	[(dppbO_2_)_3_Ni][I_3_]_2_
compound no.	**18**	[**19**][I_3_]_2_	[**19**][I_3_]_2_
cocryst solvent	none	none	none
formula	C_58_H_48_NiP_4_S_4_	C_90_H_72_I_6_NiO_6_P_6_	C_90_H_72_I_6_NiO_6_P_6_
fw, g mol^–1^	1055.79	2255.41	2255.40
temperature, K	100	100	150
wavelength, Å	0.71073	0.71073	0.71073
2θ range, deg	2.46–49.52	3.12–54.00	3.628–46.660
cryst syst	monoclinic	triclinic	monoclinic
space group	*P*2_1_/*c*	*P*1̅	*P*2_1_/*c*
*a*, Å	9.3284(12)	13.0484(12)	15.0552(10)
*b*, Å	30.386(4)	13.3478(12)	26.0258(18)
*c*, Å	19.858(3)	26.880(3)	22.6547(15)
α, deg	90	102.137(1)	90
β, deg	98.487(2)	92.504(1)	101.217(2)
γ, deg	90	106.961(1)	90
volume (Å^3^), *Z*	5567.1(13), 4	4350.1(7), 2	8707.1(10), 4
density, g cm^–3^	1.260	1.722	1.721
μ, mm^–1^	0.649	2.514	2.512
color, habit	yellow needle	orange plate	orange-red plate
limiting indices *h*	–10 ≤ *h* ≤ 10	–16 ≤ *h* ≤ 16	–15 ≤ *h* ≤ 16
limiting indices *k*	–35 ≤ *k* ≤ 35	–17 ≤ *k* ≤ 17	–28 ≤ *k* ≤ 28
limiting indices *l*	–23 ≤ *l* ≤ 23	–34 ≤ *l* ≤ 34	–25 ≤ *l* ≤ 25
reflns collected	39326	37066	153572
indep data, param^a^	9510, 610	18761, 985	12505, 989
GOF^b^	0.964	0.945	1.023
R1,^c^^,^^d^ wR2^d^^,^^e^	0.0898, 0.1686	0.0427, 0.0931	0.0505, 0.1241
R1,^c^^,^^f^ wR2^e^^,^^f^	0.1753, 0.2007	0.0707, 0.1017	0.0794, 0.1403

a Independent data collected and parameters refined.

b GOF = {∑[*w*(*F*_o_^2^ – *F*_c_^2^)^2^]/(*n* – *p*)}^1/2^, where *n* = number of reflections and *p* is the total number of parameters refined.

c R1 = ∑||*F*_o_| – |*F*_c_||/∑|*F*_o_|.

d *R* indices for data cut off at *I* > 2σ(*I*).

e wR2 = {∑[*w*(*F*_o_^2^ – *F*_c_^2^)^2^]/∑[*w*(F_o_^2^)^2^]}^1/2^; *w* = 1/[σ^2^(*F*_o_^2^) + (*xP*)^2^ + *yP*], where *P* = [2*F*_c_^2^ + Max(*F*_o_^2^,0)]/3.

f *R* indices for all data.

**Table 3:** Selected Interatomic Distances, Bond Angles, And Other Structural Parameters for Compounds **1**, **4**, **5**, **7**, [(pdt)M(η^2^-tpbz)] (M = Ni, **8**; Pd, **9**; Pt, **11**), and **12**^a^

	**1**	**4**	**5**	**7**	**8**	**9**	**11**	**12**
M–X^b^	2.1814[8]	2.1489[6]	2.1494(6)	2.2933(8)	2.1518(5)	2.2935(6)	2.2993(8)	2.1457[6]
M–P^c^	2.1320[8]	2.1542[6]	2.1620(6)	2.2431(8)	2.1712(8)	2.2713(2)	2.2521(9)	2.1503[6]
Δ,^d^ Å		0.0053	0.0126	–0.0502	0.0194	–0.0222	–0.0472	0.0046
S–C		1.738[2]	1.765(2)	1.760(3)	1.7568(16)	1.767(2)	1.767(3)	1.757[2]
_S–_C=C_–S_		1.353(5)	1.333(5)	1.327(6)	1.346(3)	1.340(5)	1.348(7)	1.353(4)
P–C_bound_^e^	1.814[3]	1.813[2]	1.827(2)	1.822(3)	1.8242(16)	1.825(2)	1.820(3)	1.827[2]
P–C_open_^f^	1.852[3]	1.848[2]	1.848(2)	1.849(3)	1.8449(16)	1.849(2)	1.840(3)	1.843[2]
md,^g^ Å	0.144	0.267	0.070	0.035	0.125	0.112	0.112	0.243
δ,^h^ Å	0.019	0.028	0.000	0.000	0.000	0.000	0.000	0.056
X–M–X^b^	94.73(5)	93.62(3)	90.83(3)	88.16(4)	90.89(2)	88.35(3)	88.04(4)	91.71(3)
P–M–P^c^	88.68(2)	87.65(3)	89.38(3)	87.74(4)	88.64(4)	86.50(3)	86.80(4)	89.57(3)
X–M–P_cis_^b^	89.04[4]	92.00[2]	90.08(2)	92.09(3)	90.827(14)	93.01(2)	93.01(3)	89.68[2]
X–M–P_trans_^b^	169.96[4]	162.19[3]	175.30(2)	177.75(3)	171.658(16)	172.88(8)	172.92(3)	163.90[3]
θ,^i^ deg	13.1	24.9	6.6	3.2	11.7	10.2	10.2	22.5
φ,^j^ deg	3.5	7.2	1.2	1.2	2.6	2.4	2.5	1.9

a Averaged values are given where two or more chemically identical interatomic distances or angles are present. Uncertainties are propagated according to Taylor, J. R. *An Introduction to Error Analysis*, 2nd ed.; University Science Books: Sausalito, CA, 1997; pp 73–77; propagated uncertainties are designated with [ ].

b M = Ni, X = Cl; M = Ni, Pd, or Pt and X = S.

c M = Ni, Pd, or Pt.

d Δ = M–P – M–S bond length difference.

e C atom of the central arene ring, metalated side.

f C atom of the central arene ring, open side.

g md = mean atom deviation from the X_2_MP_2_ plane.

h δ = deviation (Å) of M from the X_2_MP_2_ plane.

i Angle between the S_2_M and P_2_M planes.

j Angle between the MP_2_ and P_2_C_6_P_2_ mean planes.

Bis(dithiolene)diphosphine compound **10** is an intermediate on the pathway toward **11** and is isolable in varying amounts depending the temperature and length of the reaction time. Compound **10** occurs as a result of an atypical oxidative addition reaction, wherein the two electrons from Pt^II^ in [(Ph_2_C_2_S_2_)_2_Pt] are distributed to the dithiolene radical monoanions (**b** → **a**, Scheme 1), thereby reducing them to ene-1,2-dithiolates rather than to the incoming ligand (diphosphine), as is usually the case. The subsequent transformation of **10** to **11** is a reductive elimination that extrudes one dithiolene ligand in a highly reactive fully oxidized form (**c** or possibly **d**, Scheme 1) and restores the Pt^II^ redox state. In related systems, trapped forms of this expelled dithiolene ligand, e.g., as a 1,2,5,6-tetrathiocin^19^ or as a 1,3-dithiol-2-alkylimine,^20^ affirm this general accounting of the redox transaction. The crystal structure of **10** (Figure 2) establishes the octahedral geometry that is common for Pt^IV^, with both dithiolene ligands in the fully reduced ene-1,2-dithiolate redox level, as gauged by S–C and C–C_chelate_ bond lengths (Table 4). These S–C and C–C_chelate_ bond lengths are markedly longer and shorter, respectively, than those in the initial [(Ph_2_C_2_S_2_)_2_Pt] complex. Furthermore, UV–vis and X-ray absorption spectroscopic measurements of very similar platinum dithiolenebis(phosphine) complexes^21^ are consistent with the foregoing descriptions of the redox changes and molecular electronic structure.

**Table 4:** Selected Bond Lengths (Å) and Angles (deg) for **10**^a^

C=C_dithiolene,chelate_	1.348[3]
C–S	1.771[2]
Pt–S_cis to P_	2.3688[5]
Pt–S_trans to P_	2.3607[5]
Pt–P	2.3373[5]
S(2)–Pt(1)–S(4)	170.34(2)
S–Pt–S_intraligand_	88.63[1]
S(1)–Pt(1)–S(3)	91.23(3)
S–Pt–S^b^	84.63[1]
P–Pt–P	86.09(2)
φ^c^	15.9

a Averaged values are given where two or more chemically identical interatomic distances or angles are present. Uncertainties are propagated according to Taylor, J. R. *An Introduction to Error Analysis*, 2nd ed.; University Science Books: Sausalito, CA, 1997; pp 73–77; propagated uncertainties are designated with [ ].

b Interligand S–Pt–S, with one S atom in the PtP_2_ plane and one orthogonal to it.

c φ = angle between the PtP_2_ plane and P_2_C_6_P_2_ mean plane.

**Figure 3:**
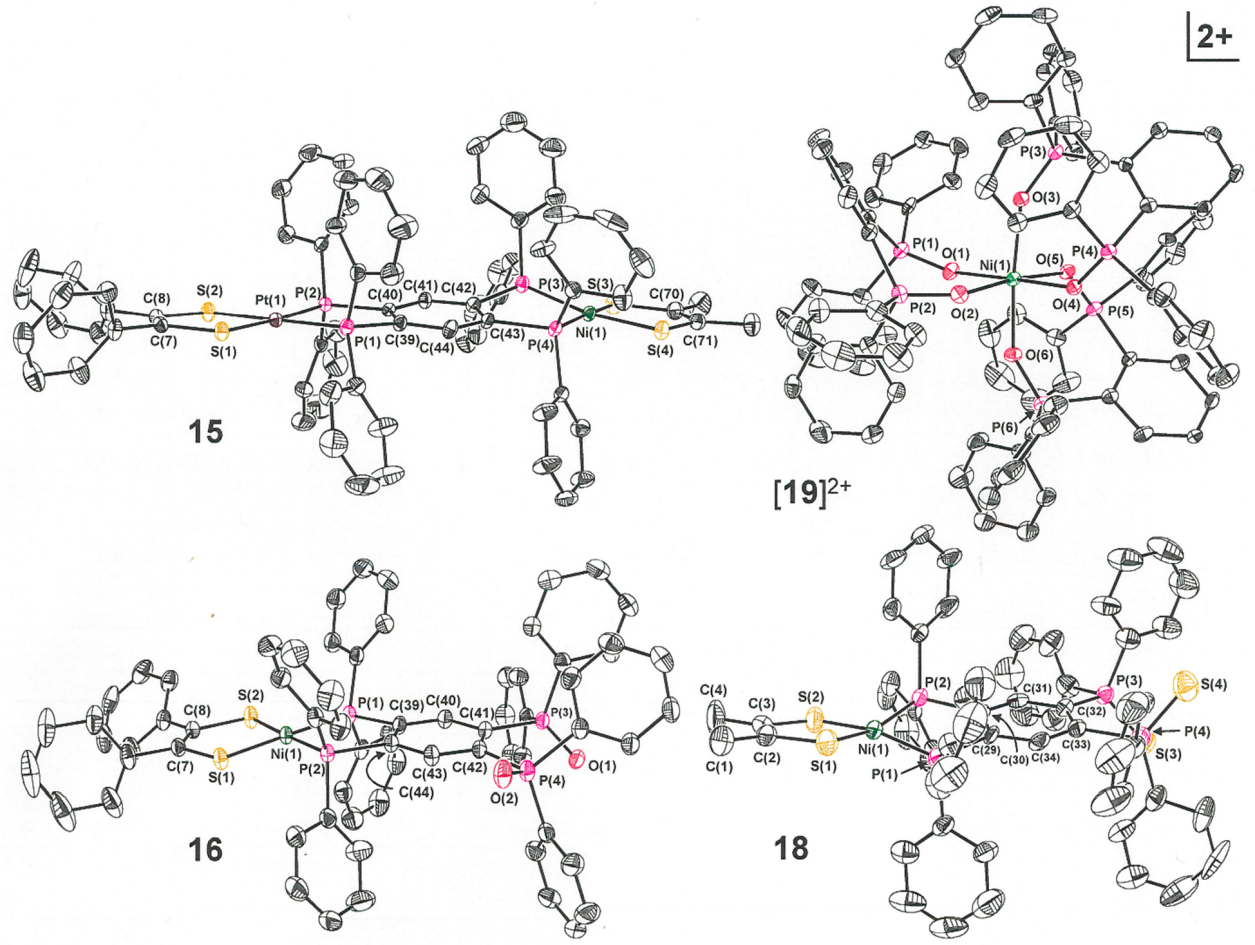
Thermal ellipsoid plots (50% probability level) of dimetallic **15**, open-ended oxidized **16** and **18**, and mononuclear [**19**]^2+^ from X-ray crystallographic characterization. All H atoms have been omitted for clarity.

Heterodimetallic compound **15** is readily prepared either by the treatment of **11** with [(Me_2_C_2_S_2_)_2_Ni] or by the reaction between **5** and [(Ph_2_C_2_S_2_)_2_Pt] (Scheme 3). This flexibility in the approach to the synthesis is enabled by the robust character of the open-ended tpbz compounds and offers a glimmer of future possibilities for “modular” synthesis. The structure of **15** (Figure 3 and Table 5) reveals bond lengths and other parameters that are consistent with independent, noninteracting metal centers. For example, the Δ_M_ values (the difference between the M–P and M–S average bond lengths) are negative and positive, respectively, for Pt and Ni, as found for the analogous homodimetallic compounds.^2^ The Ni center reveals a slight tetrahedralization (φ = 11.4°) compared to the more planar environment around Pt (φ = 3.4°).

**Table 5:** Selected Interatomic Distances (Å) and Angles (deg) for **15**^a^

Pt–S	2.2921[12]	S–Pt–S	88.81(6)
Pt–P	2.2461[12]	P–Pt–P	87.43(6)
Δ_Pt_^b^	–0.046	S–Pt–P_cis_	91.88[4]
Ni–S	2.142[1]	S–Pt–P_trans_	177.68[5]
Ni–P	2.1535[13]	θ_1_,^c^ deg	3.4
Δ_Ni_^b^	+0.012	S–Ni–S	91.21(8)
S–C_pdt_	1.758[4]	P–Ni–P	88.07(7)
S–C_mdt_	1.751[5]	S–Ni–P_cis_	90.82[5]
C=C_pdt_	1.350(9)	S–Ni–P_trans_	171.68[6]
C=C_mdt_	1.352(11)	θ_2_,^c^ deg	11.4
Pt···Ni, Å	8.811		

a Averaged values are given where two or more chemically identical interatomic distances or angles are present. Uncertainties are propagated according to Taylor, J. R. *An Introduction to Error Analysis*, 2nd ed.; University Science Books: Sausalito, CA, 1997; pp 73–77; propagated uncertainties are designated with [ ].

b Δ_M_ = (M−P) − (M−S) bond length difference.

c θ = angle between the S_2_M and P_2_M planes.

As suggested by the synthesis of **15**, the open-ended compounds of Scheme 2 are themselves phosphine ligands that should broadly manifest the reactivity associated with this ligand type. As one example, without detriment to the metalated end of tpbz, the open phosphine groups can undergo oxidative addition of the chalcogen atom to form the corresponding phosphine oxides or sulfides. Several representative compounds—[(Ph_2_C_2_S_2_)Ni(tpbzO_2_)], [(Me_2_C_2_S_2_)Pt(tpbzO_2_)], and [(Me_2_C_2_S_2_)Ni(tpbzS_2_)]—have been prepared in a straightforward fashion using I_2_/air, H_2_O_2_, [NH_4_]_2_[S], respectively. For reasons not immediately obvious, the known I_2_/air protocol^11^ that works to produce **16** in modest yield appears to be ineffective in generating the Pt compound **17**. However, the more vigorous H_2_O_2_ effectively oxidizes **7** to afford **17** in a yield comparable to the production of **16** from **8** by I_2_/air. Considering the reported susceptibility of [(bdt)Pt(bipy)] to complete oxidation of the thiolate S atoms to the disulfinate form in the presence of light and O_2_,^22^ it is somewhat surprising that the often indiscriminate H_2_O_2_ can produce **17** without significant attending decomposition. These several oxidized compounds are readily distinguished by ^31^P NMR spectroscopy from the open-ended [(R_2_C_2_S_2_)M(tpbz)] compounds because the signal arising from the trivalent P is moved 40−60 ppm downfield and nearer to the signal arising from the P nuclei bound to M^2+^. Structurally, **16**–**18** are similar to **5**–**9** and **11** in having closer adherence to planarity for the heavier metal, positive and negative Δ (Table 6) for the Ni and Pt compounds, respectively, and modestly longer P–C_central arene_ bond lengths at the open end versus the metalated end. The bond lengths from P to terminal chalogenide are typically at ∼1.49 Å for the oxide^23,24^ and ∼1.93 Å for the sulfide.^25,26^

**Table 6:** Selected Interatomic Distances (Å), Bond Angles (deg), and Other Structural Parameters for **16**–**18**^a^

	[(Ph_2_C_2_S_2_)Ni(tpbzO_2_)]	[(Me_2_C_2_S_2_)Pt(tpbzO_2_)]	[(Me_2_C_2_S_2_)Ni(tpbzS_2_)]
M–S	2.1477[5]	2.2934[4]	2.137[1]
M–P	2.1656[5]	2.2588[3]	2.141[1]
Δ^b^	+0.0179	–0.0346	+0.004
S–C	1.759[1]	1.759[1]	1.755[6]
P–C^c^	1.833[1]	1.8247[12]	1.836[5]
P–C^d^	1.840[1]	1.8323[12]	1.848[5]
P=E	1.4931[12]	1.4936[11]	1.929[2]
S–M–S	90.86(2)	88.006(18)	92.13(9)
P–M–P	89.42(2)	86.717(16)	89.95(8)
S–M–P_cis_	89.83[1]	93.265[12]	91.06[6]
S–M–P_trans_	177.76[2]	171.307[12]	164.34[6]
θ,^e^ deg	2.3	12.3	22.1
φ,^f^ deg	5.5	19.1	7.9

a Averaged values are given where two or more chemically identical interatomic distances or angles are present. Uncertainties are propagated according to Taylor, J. R. *An Introduction to Error Analysis*, 2nd ed.; University Science Books: Sausalito, CA, 1997; pp 73–77; propagated uncertainties are designated with [ ].

b Δ = (M–P) – (M–S) bond length difference.

c C atom of the central arene ring, metalated side.

d C atom of the central arene ring, open side.

e Angle between the S_2_M and P_2_M planes.

f Angle between the MP_2_ plane and P_2_C_6_P_2_ mean plane.

**Scheme 4:**
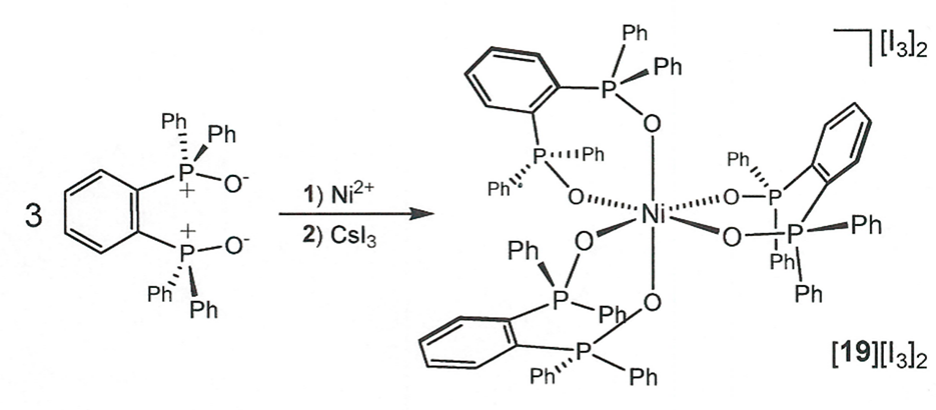
Preparation of [Ni(dppbO_2_)_3_][I_3_]_2_

The potential utility of compounds such as **17** and **18** is: (1) Their capacity to select different ions at their open end versus [(R_2_C_2_S_2_)M(tpbz)] by virtue of the ylide character to the phosphine sulfide or oxide; (2) their ability to form homoleptic tris(chelate) complexes because the otherwise prohibitive congestion that would be occasioned by six Ph_2_P groups is further removed from the coordination sphere of M^*n*+^. Such possibilities are intimated by the finding that a test reaction between the related dppbO_2_ and Ni^2+^ leads to octahedral [(dppbO_2_)_3_Ni]^2+^, which has been isolated as its I_3_^–^ salt (Scheme 4 and Figure 3) in both triclinic and monoclinic forms. Selected structural parameters, which are highly similar for both polymorphs, are presented in Table S5. The phosphoryl P–O bond lengths in [**19**]^2+^ (1.491[1] Å) are slightly longer than those in the free ligand (1.485[1] Å).^24^ Each ligand shows a substantial folding around the intraligand O···O axis such that the NiO_2_ plane meets the P_2_C_2 chelate_ mean plane at an angle of ∼57° (average of six values). For both structures of [**19**]^2+^, the directionality of this folding is different for one dppbO_2_ ligand than it is for the other two, thus lowering the symmetry from *C*_3_ to *C*_1_. These structures for [**19**]^2+^ appear to be the first for a homoleptic tris(chelate) coordination complex with dppbO_2_, although a preparation of the [(dppbO_2_)_3_M]^2+^ (M = Mg^2+^, Ca^2+^, Sr^2+^, Ba^2+^) series has been reported.^27^ A related tris(chelate) of Ni^2+^, [(dppeO_2_)_3_Ni]^2+^ (dppeO_2_ = 1,2-bis(diphenylphosphino)ethane dioxide), has been structurally authenticated^28^ but notably contrasts with [**19**]^2+^ in having a much more modest average angle of 9.2° between the NiO_2_ and P_2_C_2 chelate_ planes for its ligands.

### Electrochemistry

**Figure 4:**
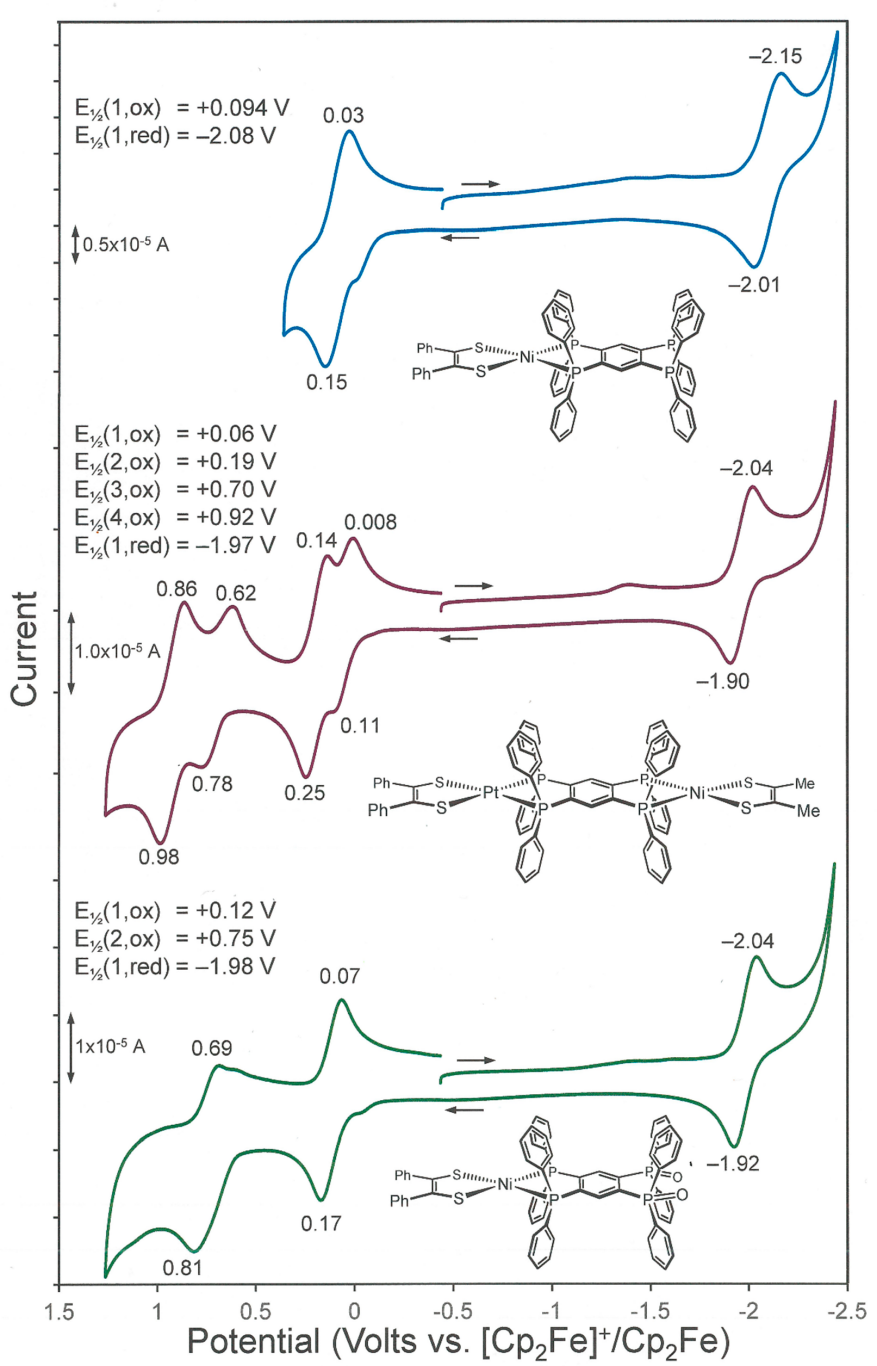
Cyclic voltammetry of selected compounds from Schemes 2 and 3.

The open-ended compounds [(R_2_C_2_S_2_)M(tpbz)] generally support a reversible 1e^–^ oxidation that is attributed to transformation of the ene-1,2-dithiolate dianion into its radical monoanionic form (Scheme 1, **a** → **b**). The potential at which this oxidation occurs is shifted cathodically by ∼0.050 V compared to the corresponding feature in the symmetric, dimetallic complex. This modest shift appears to be due to an inherent electron-donating character of the tetraphosphino ligand platform, which is tempered somewhat when the ligand’s open end chelates a second M(S_2_C_2_R_2_) group. However, the second reversible oxidation that occurs in [(R_2_C_2_S_2_)M(tpbz)M(S_2_C_2_R_2_)] (M = Ni, Pd, Pt; R = Ph, *p*-anisyl) is typically irreversible in their open-ended counterparts (Table 7). In [(R_2_C_2_S_2_)M(tpbz)M(S_2_C_2_R_2_)], this second wave was assigned to radical monoanion to α-dithione oxidation (Scheme 1, **b** → **c**). Assuming a similar description pertains to the open-ended compounds, it is unclear why reversible behavior is not sustained under the same conditions. The cathodic direction reveals a single 1e^–^ reduction for [(R_2_C_2_S_2_)M(tpbz)] that is due to a reduction of the tpbz ligand. The more comparable scaling of the current amplitude for this reduction relative to the oxidation waves is consistent with the assignment of all processes as one-electron events (Figure 4 (top)). In contrast, the oxidation waves in [(R_2_C_2_S_2_)M(tpbz)M(S_2_C_2_R_2_)] are 2e^–^ processes and display substantially greater current compared to the reduction wave, which is also due to the reduction of tpbz. In open-ended [(R_2_C_2_S_2_)M(tpbz)], the reduction wave is shifted to a more negative potential than that in the corresponding dimetallic compound. Here again, the effect is accounted for by the greater electron richness of the tpbz ligand when metalated at one end instead of both. The description of these redox events is corroborated by structure optimizations that reveal the HOMO and LUMO for **8** to be dithiolene-based and predominantly tpbz-based, respectively (Figure S116).

**Table 7:** Electrochemical Data (V) for the Open-Ended Compounds **4**–**6** and **8**–**15** for Dimetallic tpbz-Bridged **15** and for Open-Ended Oxidized **16** versus Cp_2_Fe^+^/Cp_2_Fe with a 0.10 M [Bu_4_N][PF_6_] Supporting Electrolyte, a Glassy Carbon or Pt Disk Working Electrode, and CH_2_Cl_2_ as the Solvent^a^

	*E*_4_(ox)	*E*_3_(ox)	*E*_2_(ox)	*E*_1_(ox)	*E*_1_(red)
[(mnt)Ni(tpbz)]^b^, **4**				+0.62 (ir, 1e)^d^	–1.53 (r, 1e)^e^
[(mdt)Ni(tpbz)]^c^, **5**			+0.71 (ir, 1e)^d^	0.00 (r, 1e)^e^	–2.11 (qr, 1e)^f^
[(mdt)Pt(tpbz)]^c^, **6**		+0.85 (ir)^g^	+0.66 (ir)^g^	–0.01 (r, 1e)^e^	
[(pdt)Ni(tpbz)]^c^, **8**			+0.71 (ir, 1e)^e^	+0.09 (r, 1e)^e^	–2.10 (r, 1e)^e^
[(pdt)Pd(tpbz)]^c^, **9**		+0.85 (ir, 1e)^d^	∼+0.66 (ir, 1e)^d^	+0.05 (r, 1e)^e^	
[(pdt)Pt(tpbz)]^c^, **11**		+0.88 (qr, 1e)^e^	+0.54 (ir, 1e)^d^	+0.13 (r, 1e)^e^	–1.97 (qr, 1e)^f^
[(pdt)_2_Pt(tpbz)]^b^, **10**	+0.66 (ir)^g^	+0.48 (r, 1e)^e^	+0.16 (qr, 1e)^f^	–0.02 (r, 1e)^e^	–1.22 (ir)^g^
[(adt)Ni(tpbz)]^c^, **12**			+0.59 (qr, 1e)^f^	+0.02 (r, 1e)^e^	–2.11 (qr, 1e)^f^
[(adt)Pd(tpbz)]^c^, **13**			+0.67 (ir, 1e)^d^	+0.09 (r, 1e)^e^	
[(adt)Pt(tpbz)]^c^, **14**			+0.60 (qr, 1e)^f^	+0.01 (r, 1e)^e^	
[(pdt)Pt(tpbz)Ni(mdt)]^c^, **15**	+0.92 (r, 1e)^e^	+0.70 (r, 1e)^e^	+0.19 (r, 1e)^e^	+0.06 (r, 1e)^e^	–1.97 (r, 1e)^e^
[(pdt)Ni(tpbzO_2_)]^c^, **16**			+0.75 (qr, 1e)^f^	0.12 (r, 1e)^e^	–1.98 (r, 1e)^e^

a Ligand abbreviations: mnt = [(NC)_2_C_2_S_2_]^2–^, mdt = [Me_2_C_2_S_2_]^2–^, pdt = [Ph_2_C_2_S_2_]^2–^, and adt = [(MeO-*p*-C_6_H_4_)_2_C_2_S_2_]^2–^.

b A Pt disk working electrode was used.

c A glassy carbon working electrode was used.

d ir = irreversible; value obtained by differential pulse voltammetry.

e r = reversible; value obtained by cyclic voltammetry.

f qr = quasireversible; value obtained by cyclic voltammetry.

g ir = irreversible; value estimated from the anodic maximum, *E*_a_, in the cyclic voltammogram.

**Figure 5:**
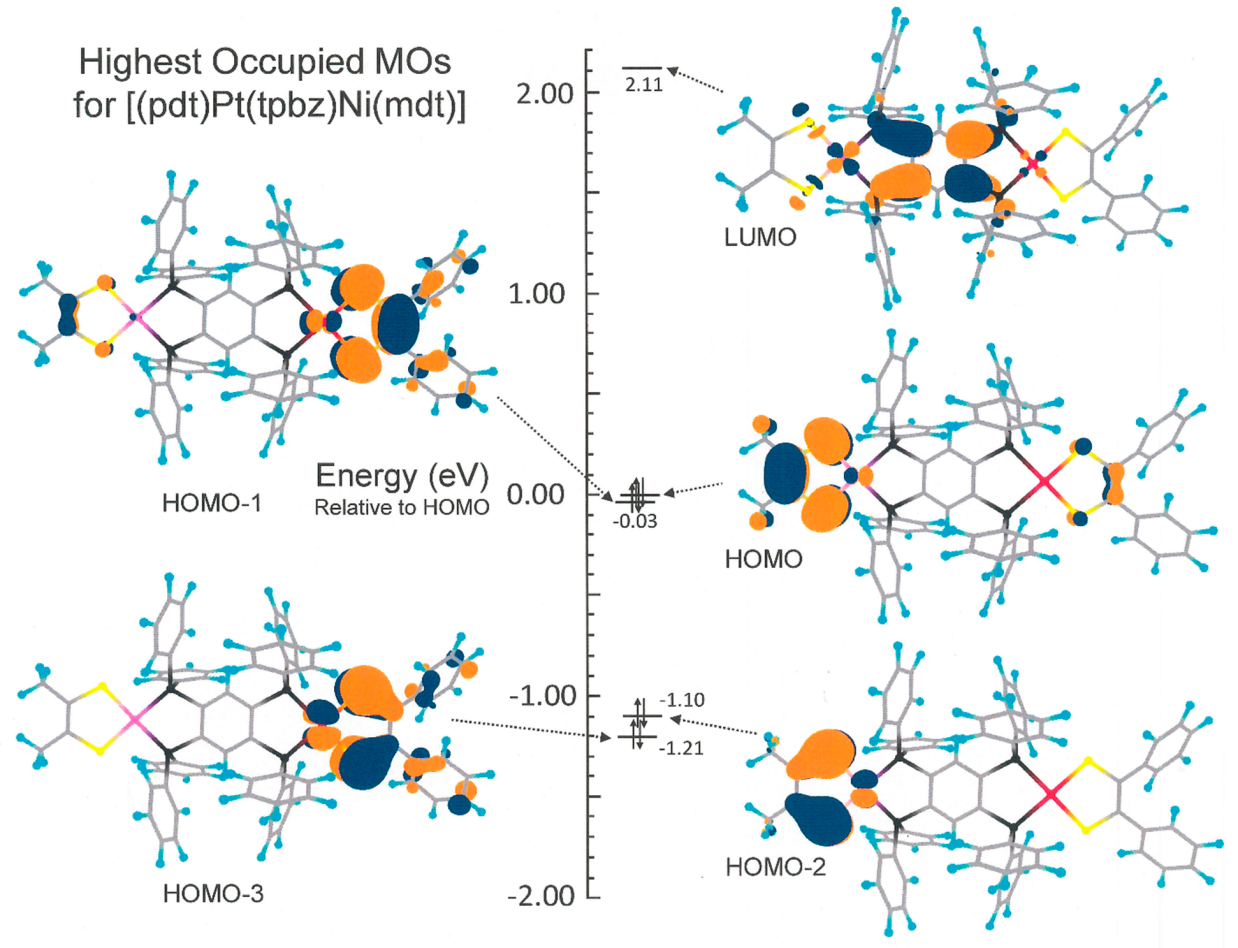
Frontier molecular orbitals for [(Ph_2_C_2_S_2_)Pt(tpbz)Ni(S_2_C_2_Me_2_)], showing the HOMO and HOMO–1 to be largely localized at the Ni(S_2_C_2_Me_2_) and Pt(S_2_C_2_Ph_2_) ends, respectively. Orbital images are shown at the 0.03 contour level.

Because of the asymmetry in **15**, the successive oxidation waves that arise from ene-1,2-dithiolate to radical monoanion (Scheme 1, **a** → **b**) and radical monoanion to α-dithione (Scheme 1, **b** → **c**) oxidation are partially resolved [Figure 4 (middle)]. In our earlier study involving the centrosymmetric homodimetallic compounds [(R_2_C_2_S_2_)M(tpbz)M(S_2_C_2_R_2_)],^2^ a consistent anodic shift of +0.04–0.05 V was observed upon moving from the methyl- to phenyl-substituted dithiolene ligand, while the metal ion was constant as either Ni^2+^, Pd^2+^, or Pt^2+^. This difference was attributed to a modestly greater electron-donating effect for Me over Ph, which enables the ligand-based oxidation to occur at a less positive potential. Similarly, the change in [(R_2_C_2_S_2_)M(tpbz)M(S_2_C_2_R_2_)] from Ni^2+^ to Pt^2+^ with a constant dithiolene ligand occasioned a +0.13–0.14 V anodic shift, which was ascribed to a greater dipositive character of the third-row metal compared to the first-row metal. Assuming the effects of the dithiolene substituent and of the metal-ion identity to be simply additive leads to the prediction that [(Ph_2_C_2_S_2_)Pt(tpbz)Ni(S_2_C_2_Me_2_)] would undergo oxidation at its Ni(S_2_C_2_Me_2_) end first, with ∼0.18 V separating that process from an oxidation of the same nature at the Pt(S_2_C_2_Ph_2_) end. As gauged by the anodic and cathodic maxima in the voltammogram [Figure 4 (middle)], the Δ*E*_1/2_ separating the first and second oxidation processes is ∼0.13 V, while the Δ*E*_1/2_ between the third and fourth waves is ∼0.22 V. The assignment of the first anodic wave as Ni(^−^S_2_C_2_Me_2_) – 1e^–^ → Ni(^−^S^•^SC_2_Me_2_)^+^ and the second as (Ph_2_C_2_S^–^_2_)Pt – 1e^–^ → (Ph_2_C_2_S^–^S^•^)Pt^+^ is affirmed by a geometry optimization of **15**. The calculated electronic structure shows the HOMO to be predominantly constituted of the Ni(S_2_C_2_Me_2_) end, while the HOMO–1, which likely becomes the HOMO in [**15**]^+^, is largely localized at the opposite (Ph_2_C_2_S_2_)Pt terminus (Figure 5). The 1e^–^ cathodic wave at −1.97 V, which arises from a reduction of the tpbz ligand, occurs at a potential very similar to its place in the homodimetallic compounds.

Although they have been known for some time,^8,21,30−32^ platinum dithiolene compounds of the type [(R_2_C_2_S_2_)_2_Pt(phosphine)_2_] have been little studied electrochemically. In the oxidizing direction, the cyclic voltammogram of **10** reveals multiple reversible and partially reversible waves (Figure S75). Given the reduced state of the dithiolene ligand, as inferred from the structural data, these processes are likely successive 1e^–^ oxidations of the dithiolene ligands, of which there are four in principle. Cathodic scanning reveals irreversible behavior beginning at ∼−1.22 V versus the [Cp_2_Fe]^+^/[Cp_2_Fe] couple, which is likely metal-based and possibly leads to the extrusion of one of the dithiolene ligands.

### EPR Spectroscopy

**Figure 6:**
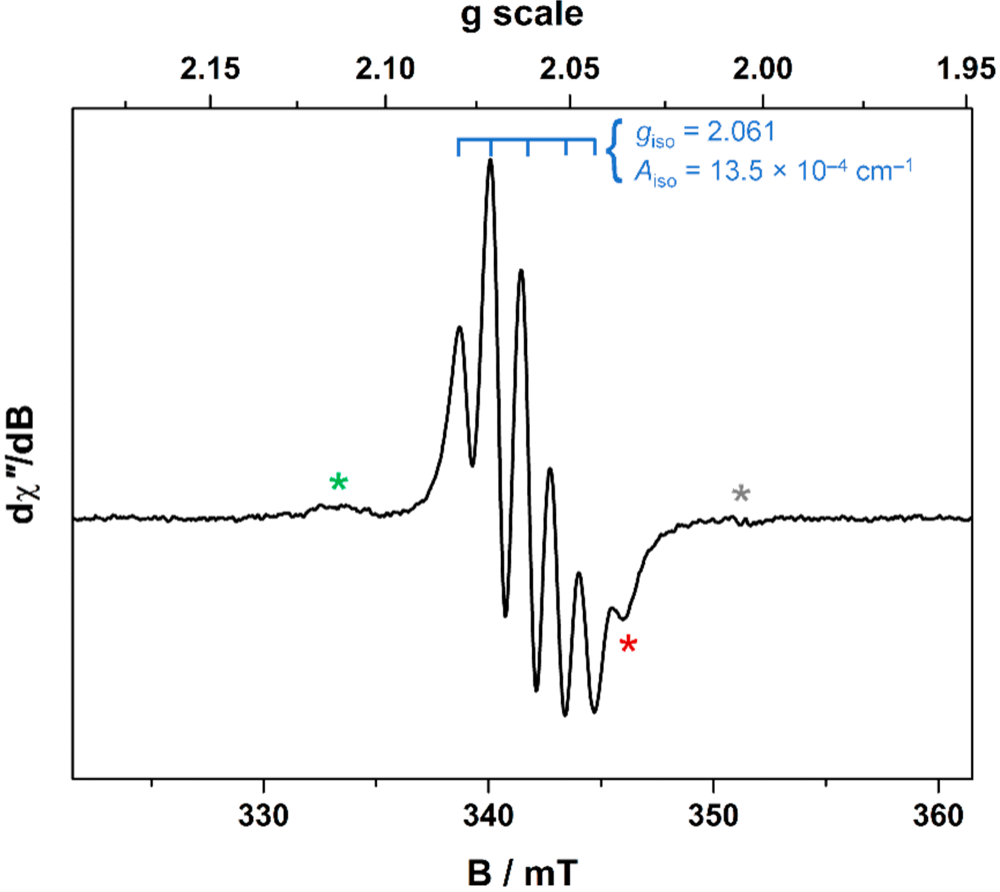
X-band EPR spectrum of **8** treated with 2 equiv of tris(4-bromophenyl)ammoniumyl hexachloroantimonate in CH_2_Cl_2_ recorded at room temperature (experimental conditions: frequency, 9.8587 GHz; power, 6.3 mW; modulation, 0.4 mT). The main signal indicated in blue comprises the five lines with *g*_iso_ = 2.061 and *A*_iso_ = 13.5 × 10^–4^ cm^–1^. Minor signals are denoted with the red and green asterisks. The gray asterisk references the position of the oxidant (*g* ∼ 2.0036).

An aliquot of **8** was treated with successive equivalents of tris(4-bromophenyl)ammoniumyl hexachloroantimonate and monitored by EPR spectroscopy. The addition of 0.5 and 1 equiv of oxidant yielded no signal, although the reduction potential of the oxidant 0.7 V (vs Fc^+/0^) is sufficient to oxidize this compound.^33^ At >1 equiv, the main signal shown in the spectrum in Figure 6 appeared, which is comprised of fives lines in a binomial intensity pattern brought about by the coupling of four ^31^P nuclei. The signal is short-lived, diminishing over the course of 2–3 h even in a Teflon-stoppered EPR tube. On the basis of its relative intensity and position, the feature denoted by the red asterisk is a separate signal, presumably the high-field hyperfine component of a multiline signal that lies beneath the main signal. A second minor signal is identified at *g* ∼ 2.011, as indicated by the green asterisk, and comprises a weakly resolved three-line binomial pattern.

At this stage, no definitive assignment can be made of the EPR signal. The *g* and *A* values are distinct from other paramagnetic nickel diphosphine species. For example, the spin doublet [(adt^•^)Ni^II^(dppb)]^+^ has a three-line spectrum with *g*_iso_ = 2.0106 and *A*_iso_ = 3.3 × 10^–4^ cm^–1^, while the spin triplet [(adt^•^)Ni^II^(tpbz)Ni^II^(adt^•^)]^+^ has a five-line signal with *g*_iso_ = 2.011 and *A*_iso_ = 1.7 × 10^–4^ cm^–1^ [where adt^2–^ = bis(*p*-anisyl)-1,2-ethenedithiolate].^34^ The hyperfine coupling constant is 25% of that in the spectrum in Figure 6. The *g* values are also noticeably smaller, where the higher value here is better matched by [Ni(adt)(adt^•^)]^−^ with *g*_iso_ = 2.0059 (but with no hyperfine splitting). In contrast, Ni-centered *S* = ^1^/_2_ complexes with phosphine ligands have even larger hyperfine coupling constants, e.g., [Ni^I^(dppe)_2_]^+^ with *g*_iso_ = 2.090 and *A*_iso_ = 83 × 10^–4^ cm^–1^ and [(Et_2_dtc)Ni^I^(dppe)]^0^ with *g*_iso_ = 2.089 and *A*_iso_ = 81 × 10^–4^ cm^–1^ [dppe = 1,2-bis(diphenylphosphino)ethane; Et_2_dtc = *N*,*N*-diethyldithiocarbamate].^31^ The main signal here is consistent with four P donor atoms at a Ni^II^ center with a coordinated dithiolene radical and results from subsequent oxidation of the first oxidation product of the monometallic [(pdt)Ni(tpbz)]. The exact composition and construct of this paramagnetic species are not known at this time.

## Summary and Conclusions

The principal findings of this work are the follows:

(1) Open-ended compounds of the type [(R_2_C_2_S_2_)M(tpbz)] can be prepared in good yields either by the direct reaction between charge-neutral [(R_2_C_2_S_2_)_2_M] (M = Ni^2+^, Pd^2+^, Pt^2+^; R = Me, Ph, *p*-anisyl) and tpbz or by transmetalation between [Cl_2_M(tpbz)] and [(R_2_C_2_S_2_)SnR′_2_] (R = Me, R′ = ^*n*^Bu; R = CN, R′ = Me).

(2) The open-ended [(R_2_C_2_S_2_)M(tpbz)] compounds reveal ^31^P NMR signals in the 55–40 ppm range and at ∼−15.0 ppm, corresponding respectively to the metalated and open ends, in clear distinction from their symmetric homodimetallic counterparts.

(3) The open-ended compounds are subject to the reaction types typical of chelating diphosphines. They may be oxidized to diphosphine dicalcogenides at their open end, and they may be metalated asymmetrically with an altogether different ML_*n*_ fragment.

(4) The cyclic voltammogram of the asymmetric heterodimetallic compound [(Ph_2_C_2_S_2_)Pt(tpbz)Ni(S_2_C_2_Me_2_)] shows two pairs of closely spaced, but resolved, 1e^–^ oxidations that correspond to the successive oxidation of each metallodithiolene end group, first to the radical monoanion state and then to the α-dithione. In contrast, the centrosymmetric homodimetallic [(R_2_C_2_S_2_)M(tpbz)M(S_2_C_2_R_2_)] compounds show two 2e^–^ oxidation waves.

In forthcoming reports, we detail the syntheses, structures, and properties of heterotrimetallic compounds of the form [[(R_2_C_2_S_2_)M(μ-tpbz)]_2_M′L_*x*_]^*n*^, where M′L_*x*_ is a either a charge-neutral fragment or a cation, using the monometallic [(R_2_C_2_S_2_)M(tpbz)] complexes described here as fungible building units.

## Supporting Information Available

Procedures for crystal growth, X-ray diffraction data collection, and structure solution and refinement, summaries of the unit cell and refinement data (Tables S1–S4), thermal ellipsoid plots with complete atom labeling (Figures S1–S23), spectroscopic, electrochemical, and analytical data for the compounds reported (Figures S24–S115), a description of the computational procedures, an MO energy level diagram for **8** (Figure S116), and coordinates for geometry-optimized **15** and **8** (Tables S6 and S7) (PDF)

## Accession Codes

CCDC 2024446–2024456 and 2076132–2076135 contain the supplementary crystallographic data for this paper. These data can be obtained free of charge via www.ccdc.cam.ac.uk/data_request/cif, or by emailing data_request@ccdc.cam.ac.uk, or by contacting The Cambridge Crystallographic Data Centre, 12 Union Road, Cambridge CB2 1EZ, UK; fax: +44 1223 336033.
