# Supplementary material for: Open-Ended Metallodithiolene Complexes with the 1,2,4,5-Tetrakis(diphenylphosphino)benzene Ligand: Modular Building Elements for the Synthesis of Multimetal Complexes
Source: Inorg Chem. 2021 Aug 9;60(17):13177–92. doi: 10.1021/acs.inorgchem.1c01573 (PMC8424627; doi:10.1021/acs.inorgchem.1c01573)
Supplement: Supplementary file 1 — ic1c01573_si_001.pdf [file ic1c01573_si_001.pdf]

Supporting Information

Open-Ended Metallodithiolene Complexes with the 1,2,4,5-Tetrakis(diphenylphosphino)benzene (tpbz) Ligand: Modular Building Elements for the Synthesis of Multimetal Complexes

by

Satyendra Kumar,[†,£,*] Malathy Selvachandran,[†,‡] Kuppuswamy Arumugam,[§]
Mohamed C. Shaw,[†] Che Wu,[†] Michael Maurer,[†] Xiaodong Zhang,[†]
Stephen Sproules,[¥] Joel T. Mague,[†] and James P. Donahue[†,*]

[†]Department of Chemistry, Tulane University, 6400 Freret Street, New Orleans, Louisiana, 70118, U.S.A.
[£]Current Address: Xavier University of Louisiana, 1 Drexel Dr., New Orleans, Louisiana, 70125, U.S.A.
[‡]Current Address: University of Jaffna, Vavuniya Campus, Sri Lanka.
[§]Department of Chemistry, Wright State University, 3640 Colonel Glenn Hwy, Dayton, Ohio, 45435-0001, U.S.A.
[¥]WestCHEM, School of Chemistry, University of Glasgow, Glasgow G12 8QQ, United Kingdom.

Table of Contents

Procedures for Crystal Growth, Collection and Processing of Diffraction Data, and Solving and Refining of Structures.		S6-S8
Computational Details		S8-S9
References		S9-S10
**Table S1**.	Unit cell and refinement data for compounds **1**, **4**, **5** and **7**	S11
**Table S2**.	Unit cell and refinement data for compounds **8**, **9**, **11**, and **10**	S12
**Table S3**.	Unit cell and refinement data for compounds **12**, **15**, and **16**	S13
**Table S4**.	Unit cell and refinement data for compounds **17**, **18**, and $[\mathbf{19}]_2[I_3]_2$	S14
**Table S5**.	Selected bond distances and angles for $[(dppbO_2)Ni][I_3]_2$	S14
**Figure S1**.	Thermal ellipsoid plot (50%) of $[(tpbz)NiCl_2]$.	S15
**Figure S2**.	Thermal ellipsoid plot (50%) of $[((NC)_2C_2S_2)Ni(\eta^2\text{-tpbz})]$.	S16
**Figure S3**.	Thermal ellipsoid plot (50%) of interstitial $CHCl_3$ in $[((NC)_2C_2S_2)Ni(\eta^2\text{-tpbz})]\cdot 2(CHCl_3)$.	S16
**Figure S4**.	Thermal ellipsoid plot (50%) of $[(Me_2C_2S_2)Ni(\eta^2\text{-tpbz})]$.	S17
**Figure S5**.	Thermal ellipsoid plot (50%) of $[(Me_2C_2S_2)Ni(\eta^2\text{-tpbz})]$ with interstitial CH_2Cl_2.	S17
**Figure S6**.	Thermal ellipsoid plot (50%) of $[(Me_2C_2S_2)Pt(\eta^2\text{-tpbz})]$.	S18
**Figure S7**.	Thermal ellipsoid plot (50%) of $[(Me_2C_2S_2)Pt(\eta^2\text{-tpbz})]$ with interstitial CH_2Cl_2.	S19
**Figure S8**.	Thermal ellipsoid plot (50%) of $[(Ph_2C_2S_2)Ni(\eta^2\text{-tpbz})]$.	S20
**Figure S9**.	Thermal ellipsoid plot (50%) of $[(Ph_2C_2S_2)Pd(\eta^2\text{-tpbz})]$.	S20
**Figure S10**.	Thermal ellipsoid plot (50%) of $[(Ph_2C_2S_2)Pt(\eta^2\text{-tpbz})]$.	S21
**Figure S11**.	Thermal ellipsoid plot (50%) of $[(Ph_2C_2S_2)_2Pt(\eta^2\text{-tpbz})]$.	S22
**Figure S12**.	Thermal ellipsoid plot (50%) for interstitial solvent in $[(Ph_2C_2S_2)_2Pt(\eta^2\text{-tpbz})]\cdot 2(ClCH_2CH_2Cl)$.	S22
**Figure S13**.	Thermal ellipsoid plot (50%) of $[(CH_3O\text{-}p\text{-}C_6H_4)_2C_2S_2)Ni(\eta^2\text{-tpbz})]$ with one positional variant of a disordered phenyl group shown.	S23
**Figure S14**.	Thermal ellipsoid plot (50%) of $[(CH_3O\text{-}p\text{-}C_6H_4)_2C_2S_2)Ni(\eta^2\text{-tpbz})]$ with the second of two positional variants of a disordered phenyl group shown.	S23
**Figure S15**.	Thermal ellipsoid plot (50%) of $[(Ph_2C_2S_2)Pt(tpbz)Ni(S_2C_2Me_2)]$.	S24
**Figure S16**.	Thermal ellipsoid plot (50%) and atom labeling for interstitial solvent in $[(Ph_2C_2S_2)Pt(tpbz)Ni(S_2C_2Me_2)]\cdot 2½(ClCH_2CH_2Cl)$.	S24
**Figure S17**.	Thermal ellipsoid plot (50%) of $[(Me_2C_2S_2)Pt(\eta^2\text{-tpbz}O_2)]$.	S25
**Figure S18**.	Thermal ellipsoid plot (50%) of $[(Ph_2C_2S_2)Ni(\eta^2\text{-tpbz}O_2)]$.	S26
**Figure S19**.	Thermal ellipsoid plot showing interstitial CH_2Cl_2 in $[(Ph_2C_2S_2)Ni(\eta^2\text{-tpbz}O_2)]\cdot CH_2Cl_2$.	S26
**Figure S20**.	Thermal ellipsoid plot (50%) of $[(Me_2C_2S_2)Ni(\eta^2\text{-tpbz}S_2)]$.	S27
**Figure S21**.	Thermal ellipsoid plot (50%) with full atom labelling for $[Ni(dppbO_2)_3]^{2+}$ (triclinic polymorph). All H atoms are omitted for clarity.	S28
**Figure S22**.	Thermal ellipsoid plot (50%) with partial atom labelling for $[Ni(dppbO_2)_3][I_3]_2$ (triclinic polymorph). All H atoms are omitted for clarity.	S29
**Figure S23**.	Thermal ellipsoid plot (50%) with full atom labelling for $[Ni(dppbO_2)_3][I_3]_2$ (monoclinic polymorph). All H atoms are omitted for clarity.	S30

Table of Contents, Continued

**Figure S24**.	^{1}H NMR spectrum (CD_2Cl_2) of [Cl_2Ni(tpbz)].	S31
**Figure S25**.	^{31}P NMR spectrum (CD_2Cl_2) of [Cl_2Ni(tpbz)].	S31
**Figure S26**.	^{1}H NMR spectrum (DMSO-d_6) of [Cl_2Pd(tpbz)].	S32
**Figure S27**.	^{31}P NMR spectrum (DMSO-d_6) of [Cl_2Pd(tpbz)].	S32
**Figure S28**.	^{1}H NMR spectrum (DMSO-d_6) of [Cl_2Pt(tpbz)].	S33
**Figure S29**.	^{31}P NMR spectrum (DMSO-d_6) of [Cl_2Pt(tpbz)].	S33
**Figure S30**.	^{1}H NMR spectrum ($CDCl_3$) of [(mnt)Ni(tpbz)].	S34
**Figure S31**.	^{1}H NMR spectrum ($CDCl_3$) of [(mnt)Ni(tpbz)] – close-up of aromatic region.	S34
**Figure S32**.	^{31}P NMR spectrum ($CDCl_3$) of [(mnt)Ni(tpbz)].	S35
**Figure S33**.	IR spectrum (KBr disk) of [(mnt)Ni(tpbz)].	S35
**Figure S34**.	MALDI mass spectrum (positive ion mode) of [(mnt)Ni(tpbz)].	S36
**Figure S35**.	Cyclic voltammogram of [(mnt)Ni(tpbz)] in CH_2Cl_2.	S37
**Figure S36**.	Differential pulse voltammogram of [(mnt)Ni(tpbz)] in CH_2Cl_2.	S37
**Figure S37**.	^{1}H NMR spectrum ($CDCl_3$) of [($Me_2C_2S_2$)Ni(tpbz)].	S38
**Figure S38**.	^{1}H NMR spectrum ($CDCl_3$) of [($Me_2C_2S_2$)Ni(tpbz)] – close-up of aromatic region.	S38
**Figure S39**.	^{31}P NMR spectrum ($CDCl_3$) of [($Me_2C_2S_2$)Ni(tpbz)].	S39
**Figure S40**.	UV-vis spectrum (CH_2Cl_2) of [($Me_2C_2S_2$)Ni(tpbz)].	S39
**Figure S41**.	ESI mass spectrum (positive ion mode) of [(mdt)Ni(tpbz)].	S40
**Figure S42**.	CV (100 mV/sec) of [(mdt)Ni(tpbz)] in CH_2Cl_2 with [$^{n}Bu_4$N][PF_6] supporting electrolyte.	S40
**Figure S43**.	DPV (oxidizing direction, pulse amplitude 50 mV) of [(mdt)Ni(tpbz)] in CH_2Cl_2 with [$^{n}Bu_4$N][PF_6] supporting electrolyte.	S41
**Figure S44**.	DPV (reducing direction, pulse amplitude 50 mV) of [(mdt)Ni(tpbz)] in CH_2Cl_2 with [$^{n}Bu_4$N][PF_6] supporting electrolyte.	S41
**Figure S45**.	^{1}H NMR spectrum ($CDCl_3$) of [($Me_2C_2S_2$)Pd(tpbz)].	S42
**Figure S46**.	^{31}P NMR spectrum ($CDCl_3$) of [($Me_2C_2S_2$)Pd(tpbz)].	S42
**Figure S47**.	UV-vis spectrum (CH_2Cl_2) of [($Me_2C_2S_2$)Pd(tpbz)].	S43
**Figure S48**.	ESI mass spectrum (positive ion mode) of [($Me_2C_2S_2$)Pd(tpbz)].	S43
**Figure S49**.	^{1}H NMR spectrum ($CDCl_3$) of [($Me_2C_2S_2$)Pt(tpbz)].	S44
**Figure S50**.	^{31}P NMR spectrum ($CDCl_3$) of [($Me_2C_2S_2$)Pt(tpbz)].	S44
**Figure S51**.	UV-vis spectrum (CH_2Cl_2) of [($Me_2C_2S_2$)Pt(tpbz)].	S45
**Figure S52**.	ESI mass spectrum (positive ion mode) of [($Me_2C_2S_2$)Pt(tpbz)].	S45
**Figure S53**.	^{1}H NMR spectrum ($CDCl_3$) of [($Ph_2C_2S_2$)Ni(tpbz)].	S46
**Figure S54**.	^{1}H NMR spectrum ($CDCl_3$) of [($Ph_2C_2S_2$)Ni(tpbz)] – close-up of aromatic region.	S46
**Figure S55**.	^{31}P NMR spectrum ($CDCl_3$) of [($Ph_2C_2S_2$)Ni(tpbz)].	S47
**Figure S56**.	ESI mass spectrum (positive ion mode) of [($Ph_2C_2S_2$)Ni(tpbz)].	S48
**Figure S57**.	CV (100 mV/sec) of [($Ph_2C_2S_2$)Ni(tpbz)] in CH_2Cl_2 with [$^{n}Bu_4$N][PF_6] supporting electrolyte, +0.80 - –2.00 V window.	S49
**Figure S58**.	CV (100 mV/sec) of [($Ph_2C_2S_2$)Ni(tpbz)] in CH_2Cl_2 with [$^{n}Bu_4$N][PF_6] supporting electrolyte, +1.70 - –2.00 V window.	S49
**Figure S59**.	DPV (oxidizing direction, pulse amplitude 50 mV) of [($Ph_2C_2S_2$)Ni(tpbz)] in CH_2Cl_2 with [$^{n}Bu_4$N][PF_6] supporting electrolyte.	S50

Table of Contents, Continued

**Figure S60**.	Elemental analysis of $[(Ph_2C_2S_2)Ni(tpbz)]$ from Midwest Microlab, LLC.	S51
**Figure S61**.	^{1}H NMR spectrum ($CDCl_3$) of $[(Ph_2C_2S_2)Pd(tpbz)]$.	S52
**Figure S62**.	^{31}P NMR spectrum ($CDCl_3$) of $[(Ph_2C_2S_2)Pd(tpbz)]$.	S52
**Figure S63**.	ESI mass spectrum (positive ion mode) of $[(Ph_2C_2S_2)Pd(tpbz)]$.	S53
**Figure S64**.	CV (100 mV/sec) of $[(Ph_2C_2S_2)Pd(tpbz)]$ in CH_2Cl_2 with $[^{n}Bu_4N][PF_6]$ supporting electrolyte.	S54
**Figure S65**.	DPV (oxidizing direction, pulse amplitude 50 mV) of $[(Ph_2C_2S_2)Pd(tpbz)]$ in CH_2Cl_2 with $[^{n}Bu_4N][PF_6]$ supporting electrolyte.	S54
**Figure S66**.	Elemental analysis of $[(Ph_2C_2S_2)Pd(tpbz)]$ from Galbraith, Laboratories, Inc.	S55
**Figure S67**.	^{31}P NMR spectrum ($CDCl_3$) of $[(Ph_2C_2S_2)Pt(tpbz)]$.	S56
**Figure S68**.	ESI mass spectrum (positive ion mode) of $[(Ph_2C_2S_2)Pt(tpbz)]$.	S56
**Figure S69**.	CV (100 mV/sec) of $[(Ph_2C_2S_2)Pt(tpbz)]$ in CH_2Cl_2 with $[^{n}Bu_4N][PF_6]$ supporting electrolyte.	S57
**Figure S70**.	DPV (oxidizing direction, pulse amplitude 50 mV) of $[(Ph_2C_2S_2)Pt(tpbz)]$ in CH_2Cl_2 with $[^{n}Bu_4N][PF_6]$ supporting electrolyte.	S57
**Figure S71**.	Elemental analysis of $[(Ph_2C_2S_2)Pt(tpbz)]$ from Galbraith, Laboratories, Inc.	S58
**Figure S72**.	^{1}H NMR spectrum (CD_2Cl_2) of $[(Ph_2C_2S_2)_2Pt(tpbz)]$.	S59
**Figure S73**.	^{31}P NMR spectrum (CD_2Cl_2) of $[(Ph_2C_2S_2)_2Pt(tpbz)]$.	S59
**Figure S74**.	ESI mass spectrum (positive ion mode) of $[(Ph_2C_2S_2)_2Pt(tpbz)]$.	S60
**Figure S75**.	CV (100 mV/sec) of $[(Ph_2C_2S_2)_2Pt(tpbz)]$ in CH_2Cl_2 with $[^{n}Bu_4N][PF_6]$ supporting electrolyte.	S60
**Figure S76**.	^{1}H NMR spectrum ($CDCl_3$) of $[((MeO\text{-}p\text{-}C_6H_4)_2C_2S_2)Ni(tpbz)]$.	S61
**Figure S77**.	^{31}P NMR spectrum ($CDCl_3$) of $[((MeO\text{-}p\text{-}C_6H_4)_2C_2S_2)Ni(tpbz)]$.	S61
**Figure S78**.	ESI mass spectrum (positive ion mode) of $[((MeO\text{-}p\text{-}C_6H_4)_2C_2S_2)Ni(tpbz)]$.	S62
**Figure S79**.	CV (100 mV/sec) of $[((MeO\text{-}p\text{-}C_6H_4)_2C_2S_2)Ni(tpbz)]$ in CH_2Cl_2 with $[^{n}Bu_4N][PF_6]$ supporting electrolyte.	S62
**Figure S80**.	DPV of $[((MeO\text{-}p\text{-}C_6H_4)_2C_2S_2)Ni(tpbz)]$ (oxidizing direction, pulse amplitude 50 mV) of [(mdt)Ni(tpbz)] in CH_2Cl_2 with $[^{n}Bu_4N][PF_6]$ supporting electrolyte.	S63
**Figure S81**.	DPV of $[((MeO\text{-}p\text{-}C_6H_4)_2C_2S_2)Ni(tpbz)]$ (reducing direction, pulse amplitude 50 mV) of [(mdt)Ni(tpbz)] in CH_2Cl_2 with $[^{n}Bu_4N][PF_6]$ supporting electrolyte.	S63
**Figure S82**.	Elemental analysis of $[((MeO\text{-}p\text{-}C_6H_4)_2C_2S_2)Ni(tpbz)]$ from Kolbe.	S64
**Figure S83**.	^{1}H NMR spectrum ($CDCl_3$) of $[((MeO\text{-}p\text{-}C_6H_4)_2C_2S_2)Pd(tpbz)]$.	S65
**Figure S84**.	^{31}P NMR spectrum ($CDCl_3$) of $[((MeO\text{-}p\text{-}C_6H_4)_2C_2S_2)Pd(tpbz)]$.	S65
**Figure S85**.	UV-vis spectrum (CH_2Cl_2) of $[((MeO\text{-}p\text{-}C_6H_4)_2C_2S_2)Pd(tpbz)]$.	S66
**Figure S86**.	ESI mass spectrum (positive ion mode) of $[((MeO\text{-}p\text{-}C_6H_4)_2C_2S_2)Pd(tpbz)]$.	S66
**Figure S87**.	CV (100 mV/sec) of $[((MeO\text{-}p\text{-}C_6H_4)_2C_2S_2)Pd(tpbz)]$ in CH_2Cl_2 with $[^{n}Bu_4N][PF_6]$ supporting electrolyte.	S67
**Figure S88**.	DPV of $[((MeO\text{-}p\text{-}C_6H_4)_2C_2S_2)Pd(tpbz)]$ (oxidizing direction, pulse amplitude 50 mV) of [(mdt)Ni(tpbz)] in CH_2Cl_2 with $[^{n}Bu_4N][PF_6]$ supporting electrolyte.	S68
**Figure S89**.	DPV of $[((MeO\text{-}p\text{-}C_6H_4)_2C_2S_2)Pd(tpbz)]$ (reducing direction, pulse amplitude 50 mV) of [(mdt)Ni(tpbz)] in CH_2Cl_2 with $[^{n}Bu_4N][PF_6]$ supporting electrolyte.	S68

Table of Contents, Continued

Figure S90. ^{1}H NMR spectrum ($CDCl_3$) of [((MeO-*p*-C_6H_4)$_2C_2S_2$)Pt(tpbz)]. S69
Figure S91. ^{31}P NMR spectrum ($CDCl_3$) of [((MeO-*p*-C_6H_4)$_2C_2S_2$)Pt(tpbz)]. S69
Figure S92. UV-vis spectrum (CH_2Cl_2) of [((MeO-*p*-C_6H_4)$_2C_2S_2$)Pd(tpbz)]. S70
Figure S93. ESI mass spectrum (positive ion mode) of [((MeO-*p*-C_6H_4)$_2C_2S_2$)Pt(tpbz)]. S71
Figure S94. CV (100 mV/sec) of [((MeO-*p*-C_6H_4)$_2C_2S_2$)Pt(tpbz)] in CH_2Cl_2 with [$^{n}Bu_4N$][PF_6] supporting electrolyte. S71
Figure S95. DPV of [((MeO-*p*-C_6H_4)$_2C_2S_2$)Pt(tpbz)] (oxidizing direction, pulse amplitude 50 mV) of [(mdt)Ni(tpbz)] in CH_2Cl_2 with [$^{n}Bu_4N$][PF_6] supporting electrolyte. S71
Figure S96. ^{1}H NMR spectrum of [(pdt)Pt(tpbz)Ni(mdt)] in $CDCl_3$. S72
Figure S97. ^{31}P NMR spectrum of [(pdt)Pt(tpbz)Ni(mdt)] in $CDCl_3$. S72
Figure S98. UV-vis spectrum (CH_2Cl_2) of [(pdt)Pt(tpbz)Ni(mdt)]. S73
Figure S99. Mass spectrum (ESI, positive ion mode) of [($Ph_2C_2S_2$)Pt(tpbz)Ni($S_2C_2Me_2$)]. S73
Figure S100. Cyclic voltammogram of [($Ph_2C_2S_2$)Pt(tpbz)Ni($S_2C_2Me_2$)] in CH_2Cl_2 with [$^{n}Bu_4N$][PF_6]. S74
Figure S101. ^{1}H NMR spectrum ($CDCl_3$) of [($Ph_2C_2S_2$)Ni(tpbzO_2)]. S75
Figure S102. ^{31}P NMR spectrum ($CDCl_3$) of [($Ph_2C_2S_2$)Ni(tpbzO_2)]. S75
Figure S103. UV-vis spectrum (CH_2Cl_2) of [($Ph_2C_2S_2$)Ni(tpbzO_2)]. S76
Figure S104. Mass spectrum (ESI, positive ion mode) of [($Ph_2C_2S_2$)Ni(tpbzO_2)]. S76
Figure S105. Cyclic voltammogram of [($Ph_2C_2S_2$)Ni(tpbzO_2)] in CH_2Cl_2 with [$^{n}Bu_4N$][PF_6]. S77
Figure S106. ^{1}H NMR spectrum ($CDCl_3$) of [($Me_2C_2S_2$)Pt(tpbzO_2)]. S78
Figure S107. ^{31}P NMR spectrum ($CDCl_3$) of [($Me_2C_2S_2$)Pt(tpbzO_2)]. S78
Figure S108. Mass spectrum (ESI, positive ion mode) of [($Me_2C_2S_2$)Pt(tpbzO_2)]. S79
Figure S109. ^{1}H NMR spectrum ($CDCl_3$) of [($Me_2C_2S_2$)Ni(tpbzS_2)]. S80
Figure S110. ^{31}P NMR spectrum ($CDCl_3$) of [($Me_2C_2S_2$)Ni(tpbzS_2)]. S80
Figure S111. UV-vis spectrum (CH_2Cl_2) of [($Me_2C_2S_2$)Ni(tpbzS_2)]. S81
Figure S112. Mass spectrum (ESI, positive ion mode) of [($Me_2C_2S_2$)Ni(tpbzS_2)]. S81
Figure S113. Elemental analysis of [($Me_2C_2S_2$)Ni(tpbzS2)] from Galbraith. S82
Figure S114. UV-vis spectrum (CH_2Cl_2) of [Ni(dppbO_2)$_3$][I_3]$_2$. S83
Figure S115. Mass spectrum (ESI, positive ion mode) of [Ni(dppbO_2)$_3$]$^{2+}$. S83
Figure S116. MO energy level diagram showing frontier MOs for [(pdt)Ni(tpbz)]. S84
Table S6. Atomic coordinates for optimized geometry of [(pdt)Pt(tpbz)Ni(mdt)]. S85-S88
Table S7. Atomic coordinates for optimized geometry of [(pdt)Ni(tpbz)]. S89-S91

Procedures for Crystal Growth, Collection and Processing of Diffraction Data, and Solving and Refining of Structures.

All crystals used in X-ray diffraction data collections were obtained by the vial-in-a-vial vapor diffusion technique. The following solvent pairs identify specific combinations successfully employed as solvent/diffusing vapor for crystal growth: $[Cl_2Ni(tpbz)]\cdot Et_2O$ (orange plates): CH_2Cl_2/Et_2O; $[((NC)_2C_2S_2)Ni(tpbz)]\cdot 2(CHCl_3)$ (orange blocks): $CHCl_3/MeOH$; $[(Me_2C_2S_2)Ni(tpbz)]\cdot 2(CH_2Cl_2)$ (light brown-green columns): CH_2Cl_2/Et_2O; $[(Me_2C_2S_2)Pt(tpbz)]\cdot 2(CH_2Cl_2)$ (yellow plates): CH_2Cl_2/Et_2O; $[(Ph_2C_2S_2)Ni(tpbz)]$ (clear yellow blocks): CH_2Cl_2/Et_2O; $[(Ph_2C_2S_2)Pd(tpbz)]$ (pale orange columns): CH_2Cl_2/Et_2O or $ClCH_2CH_2Cl/Et_2O$; $[(Ph_2C_2S_2)Pt(tpbz)]$ (yellow columns): CH_2Cl_2/*n*-pentane; $[(Ph_2C_2S_2)_2Pt^{IV}(\eta^2\text{-}tpbz)]\cdot 2ClCH_2CH_2Cl$ (dark blue blocks): $ClCH_2CH_2Cl/Et_2O$ or $ClCH_2CH_2Cl$/*n*-pentane; $[((MeO\text{-}p\text{-}C_6H_4)_2C_2S_2)Ni(tpbz)]$ (clear yellow blocks): chlorobenzene/hexanes or chlorobenzene/^{t}BuOMe; $[(Ph_2C_2S_2)Pt(tpbz)Ni(S_2C_2Me_2)]\cdot 2½(ClCH_2CH_2Cl)$: (orange plates): $ClCH_2CH_2Cl/^tBuOMe$ or $ClCH_2CH_2Cl$/hexanes; $[(Ph_2C_2S_2)Ni(tpbzO_2)\cdot CH_2Cl_2$ (orange plates): CH_2Cl_2/Et_2O; $[(Me_2C_2S_2)Pt(tpbzO_2)\cdot 2CH_2Cl_2$ (orange plates): CH_2Cl_2/Et_2O; $[(Me_2C_2S_2)Ni(tpbzS_2)]$ (yellow needles): CH_2Cl_2/Et_2O or CH_2Cl_2/*n*-pentane; $[Ni(dppbO_2)_3][I_3]_2$ (orange-red plates): CH_2Cl_2/Et_2O for both triclinic and monoclinic polymorphs.

All crystals were coated with paratone oil and mounted on the end of a nylon loop attached to the end of the goniometer. Data were obtained at a temperature of 100, 150 or 158 K that was maintained by a cold nitrogen stream supplied under the control of an Oxford Cryostream 800 attachment. The data collection instrument was either a Bruker D8 Venture Photon 100 instrument, the radiation source for which was a Cu Incoatec I microfocus source generating X-rays with λ = 1.54178 nm, a Bruker Smart APEX II CCD diffractometer equipped with a Mo fine-focus sealed tube providing radiation at λ = 0.71073 nm, or a Bruker D8 Quest Photon 3 diffractometer that similarly operated with the Mo Kα 0.71073 nm light source.

The data sets obtained with the D8 Venture were hemispheres of data comprised from the following assemblies of ω-scan frames and frame times: $[(Ph_2C_2S_2)Ni(tpbz)]$: 10 sets of 326 or 330 frames at 10 or 20 sec/frame; $[((MeO\text{-}p\text{-}C_6H_4)_2C_2S_2)Ni(tpbz)]$: 7 sets of 368 or 371 frames at 40 or 20 sec/frame. The data sets that were collected with the APEX II diffractometer implemented one of the following programmed routines: (1) Three sets of 363 frames in ω (0.5°/scan) with φ held constant at 0, 120, and then 240°; (2) Three sets of 606 frames in ω (0.3°/scan) with φ held

constant at 0, 120, and then 240°; (3) Three sets of 400 frames in ω (0.5°/scan), collected at φ = 0.00, 90.00 and 180.00° and two sets of 800 frames in φ (0.45°/scan) collected with ω constant at -30.00 and 210.00°. The data collection program and frame time used for these data sets were as follows: [($Me_2C_2S_2$)Ni(tpbz)]·2(CH_2Cl_2): Routine 3, 60 sec; [($Me_2C_2S_2$)Pt(tpbz)]·2(CH_2Cl_2): Routine 2, 30 sec; [($Ph_2C_2S_2$)Pd(tpbz)]: Routine 1, 40 sec; [($Ph_2C_2S_2$)Pt(tpbz)]: Routine 1, 80 sec; [($Ph_2C_2S_2$)$_2$Pt(tpbz)]·2($ClCH_2CH_2Cl$): Routine 3, 60 sec; [($Me_2C_2S_2$)Ni(tpbzS_2)]: Routine 2, 90 sec; triclinic [Ni(dppbO_2)$_3$][I_3]$_2$: Routine 2, 60 sec; [Cl_2Ni(tpbz)]·Et_2O: Routine 2, 50 sec; [((NC)$_2C_2S_2$)Ni(tpbz)]·2($CHCl_3$): Routine 3: 25 sec. The data sets gathered with the D8 Quest Photon 3 ([($Ph_2C_2S_2$)Pt(tpbz)Ni($S_2C_2Me_2$)]·2½($ClCH_2CH_2Cl$), [($Ph_2C_2S_2$)Ni(tpbzO_2)·CH_2Cl_2, [($Me_2C_2S_2$)Pt(tpbzO_2)], and monoclinic [Ni(dppbO_2)$_3$][I_3]$_2$) were comprised of sets of frames, each of 0.5° width in either ω or φ, whose number and scan parameters were determined by the "Strategy" routine in APEX3.

All data were collected under control of either the Bruker SMART,[1] *APEX2*[2a-2f] or *APEX3*[2g] software packages. Raw data were reduced to F^2 values using the *SAINT+*[3] or *SAINT*[4] software, and a global refinement of unit cell parameters was performed using ~3,660–9,990 selected reflections from the full data set. For [($Ph_2C_2S_2$)Pt(tpbz)Ni($S_2C_2Me_2$)]·2.5($ClCH_2CH_2Cl$), analysis of 1675 reflections having I/σ(I) > 20 and chosen from the full data set with *CELL_NOW* showed the crystal to belong to the triclinic system and to be twinned by a 180° rotation about c*. The raw data were processed using the multi-component version of *SAINT* under control of the two-component orientation file generated by *CELL_NOW*,[5] and an absorption correction was applied using the *TWINABS* routine.[6] All other data sets were corrected for absorption on the basis of multiple measurements of symmetry equivalent reflections or by numerical methods with the use of *SADABS*,[7] as described by Krause *et al*.[8] All structure solutions were obtained by direct methods using *SHELXM*,[9] *SHELXS*[10] or *SHELXT*,[11] while refinements were accomplished by full-matrix least-squares procedures using *SHELXL*.[12] Both the *SHELXS* and *SHELXL* programs are incorporated into the *SHELXTL*[13] and *APEX2*/*APEX3*[2] software suites.

All structure refinements were routine except as noted: (**1**) In the structure of [($Me_2C_2S_2$)Ni(tpbzS_2)] (JPD184), one terminal sulfido ligand was disordered between two positions and was modeled as an 88:12 distribution between the two sites; (**2**) For [((MeO-*p*-C_6H_4)$_2C_2S_2$)Ni(tpbz)] (JPD950), one phenyl group was disordered between two positions and refined with isotropic thermal parameters as a best-fit distribution between the two sites; (**3**) For

$[(Ph_2C_2S_2)_2Pt^{IV}(\eta^2\text{-tpbz})]\cdot 2ClCH_2CH_2Cl$ (JPD736), minor disorder for one phenyl group of the tpbz ligand and one Cl atom of the interstitial solvent was observed and similarly treated with split atom models. (4) In the structure of $[(Ph_2C_2S_2)Ni(tpbzO_2)]\cdot(CH_2Cl_2)$, residual density attributed to partially occupied/disordered solvent CH_2Cl_2 sites was removed with *PLATON SQUEEZE*[14] (Spek, 2015). Furthermore, five reflections determined to be partially or wholly obscured by the beamstop were omitted from the final refinement. (**5**) The structure of $[(Ph_2C_2S_2)Pt(tpbz)Ni(S_2C_2Me_2)]\cdot 2.5(ClCH_2CH_2Cl)$ was refined as a two-component twin, and the disordered interstitial $ClCH_2CH_2Cl$ molecules were refined with constraints approximating ideal geometries. (**6**) The monoclinic polymorph of $[Ni(dppbO_2)_3][I_3]_2$ showed one triiodide ion (I1···I3) to be disordered over two closely neighboring sites in a 0.703(4)/0.297(4) ratio. The two components of the disorder were refined as rigid rods. Additionally, a small peak appearing to be a partially occupied lattice water site was removed with PLATON SQUEEZE.[14] Seven reflections partially or totally obscured by the beamstop were omitted from the final refinement. In all the structures, hydrogen atoms were added in calculated positions and included as riding contributions with isotropic displacement parameters 1.2-1.5 times those of the carbon atoms to which they were attached. Thermal ellipsoid images have been created with the use of *XP*, which also is part of the *SHELXTL* package. All structures were checked for overlooked symmetry and other errors by the checkCIF service provided by the International Union of Crystallography.[15] Final unit cell data and refinement statistics are collected in **Tables S1-S4**.

Computational Details

The density functional theory (DFT) calculations were carried out at the supercomputing facility at Tulane University, using the GAUSSIAN-09 package.[16] Geometry optimizations of structures were carried out with no symmetry restrictions beginning with the coordinates of the molecules from X-ray crystallographic data. The geometries were optimized at the Becke, 3-Parameter, Lee-Yang-Parr (B3LYP) level of theory[17] with typical basis sets. Frequency calculations were done to confirm the validity of optimized structures. For the transition metals (nickel, palladium and platinum), a double-ζ (DZ) basis set with an effective electron core potential (LANL2DZ ECP) was implemented.[18] The 6-31G (d,p) basis set was chosen for the light main group elements (C and N), whereas the triple-ζ (TZVP) was used for the heavier elements (S and P), and a Gaussian split valence (SV) basis set[19] was used for the hydrogen atoms. The molecular

orbital (MOs) plots were created using the Chemcraft[20] program package (http://www.chemcraftprog.com).

References

(**1**) *SMART*, Version 5.625, Bruker-AXS, Madison, Wisconsin, 2000.

(**2**) (**a**) *APEX2*, Version 2008.6-1, Bruker-AXS, Madison, Wisconsin, 2008. (**b**) *APEX2*, Version 2009.1-0, Bruker-AXS, Madison, Wisconsin, 2009. (**c**) *APEX2*, Version 2009.9-0, Bruker-AXS, Madison, Wisconsin, 2009. (**d**) *APEX2*, Version 2009.11-0, Bruker-AXS, Madison, Wisconsin, 2009. (**e**) *APEX2*, Bruker-AXS, Inc., Madison, Wisconsin, USA, 2014. (**f**) *APEX2*, Bruker-AXS, Inc., Madison, Wisconsin, USA, 2015. (**g**) *APEX3*, Bruker-AXS, Inc., Madison, Wisconsin, USA, 2016. (**h**) *APEX3*, Bruker-AXS, LLC, Madison, Wisconsin, USA, 2020.

(**3**) *SAINT+*, Bruker-AXS, Version 7.03, Madison, Wisconsin, 2004.

(**4**) (**a**) *SAINT*, Version 7.60A, Bruker AXS, Inc., Madison, Wisconsin, 2008. (**b**) *SAINT*, Version 7.68A, Bruker AXS, Inc., Madison, Wisconsin, 2009. (**c**) *SAINT*, Bruker AXS, Inc., Madison, Wisconsin, 2014. (**d**) *SAINT*, Bruker AXS, Inc., Madison, Wisconsin, 2015. (**e**) *SAINT*, Bruker AXS, LLC, Madison, Wisconsin, 2020.

(**5**) Sheldrick, G, M. *CELL_NOW*, University of Göttingen, Göttingen, Germany, 2008.

(**6**) Sheldrick, G, M. *TWINABS*, University of Göttingen, Göttingen, Germany, 2009.

(**7**) (**a**) Sheldrick, G. M. *SADABS*, Version 2.05, Universität Göttingen, Göttingen, Germany, 2002. (**b**) Sheldrick, G. M. *SADABS*, Version 2007/4, Universität Göttingen, Göttingen, Germany, 2007. (**c**) Sheldrick, G. M. *SADABS*, Version 2008/2, Universität Göttingen, Göttingen, Germany, 2008. (**d**) *SADABS*, Bruker AXS, Inc., Madison, Wisconsin, 2014. (**e**) *SADABS*, Bruker AXS, Inc., Madison, Wisconsin, 2015. (**f**) *SADABS*, Bruker AXS, Inc., Madison, Wisconsin, 2016.

(**8**) Krause, L.; Herbst-Irmer, R.; Sheldrick, G.M.; Stalke; D. Comparison of Silver and Molybdenum Microfocus X-ray Sources for Single-Crystal Structure Determination. *J. Appl. Cryst.* **2015**, *48*, 3-10.

(**9**) Sheldrick, G. M. *SHELXM*, Version 2004/1, University of Göttingen, Göttingen, Germany, 2004.

(**10**) (**a**) Sheldrick, G. M. *SHELXS-97*, University of Göttingen, Göttingen, Germany, 1997. (**b**) Sheldrick, G. M. *SHELXS-97*, University of Göttingen, Göttingen, Germany, 2008. (**c**) Sheldrick, G. M. A Short History of SHELX. *Acta Crystallogr., Sect. A: Foundations Adv.* **2008**, *64*, 112-122.

(**11**) (**a**) *SHELXT*, Bruker AXS, Inc., Madison, Wisconsin, 2014. (**b**) Sheldrick, G. M. *SHELXT* – Integrated Space-Group and Crystal-Structure Determination. *Acta Crystallogr., Sect. A: Foundations Adv.* **2015**, *71*, 3-8.

(**12**) (**a**) Sheldrick, G. M. *SHELXL-97*, University of Göttingen, Göttingen, Germany, 1997. (**b**) Sheldrick, G. M. *SHELXL-97*, University of Göttingen, Göttingen, Germany, 2008. (**c**) Sheldrick, G. M. A Short History of SHELX. *Acta Crystallogr., Sect. A* **2008**, *64*, 112-122. (**d**) Sheldrick, G. M. *SHELXL-2014*. University of Göttingen, Göttingen, Germany, 2014. (**e**) Sheldrick, G. M. *SHELXL-2014/7*. University of Göttingen, Göttingen, Germany, 2015. (**f**) Sheldrick, G. M.

SHELXL. University of Göttingen, Göttingen, Germany, 2015. (**g**) Sheldrick, G. M. *SHELXL-2018/1* University of Göttingen, Göttingen, Germany, 2018.

(**13**) (**a**) *SHELXTL*, Version 6.10, Bruker-AXS, Madison, Wisconsin, 2000. (**b**) *SHELXTL*, Version 2008/4, Bruker-AXS, Madison, Wisconsin, 2008. (**c**) *SHELXTL*, Bruker-AXS, Madison, Wisconsin, 2014. (**d**) *SHELXTL*, Bruker-AXS, Madison, Wisconsin, 2015. (**e**) *SHELXTL*, Bruker-AXS, Madison, Wisconsin, 2016.

(**14**) Spek, A.L. *PLATON SQUEEZE*: A Tool for the Calculation of the Disordered Solvent Contribution to the Calculated Structure Factors. *Acta Crystallogr., Sect. C: Struct. Chem.* **2015**, *71*, 9-18.

(**15**) See http://checkcif.iucr.org/

(16) Gaussian 09, Revision A. 02, Frisch, M. J.; Trucks, G. W.; Schlegel, H. B.; Scuseria, G. E.; Robb, M. A.; Cheeseman, J. R.; Scalmani, G.; Barone, V.; Mennucci, B.; Petersson, G. A.; Nakatsuji, H.; Caricato, M.; Li, X.; Hratchian, H. P.; Izmaylov, A. F.; Bloino, J.; Zheng, G.; Sonnenberg, J. L.; Hada, M.; Ehara, M.; Toyota, K.; Fukuda, R.; Hasegawa, J.; Ishida, M.; Nakajima, T.; Honda, Y.; Kitao, O.; Nakai, H.; Vreven, T.; Montgomery, J. A., Jr.; Peralta, J. E.; Ogliaro, F.; Bearpark, M.; Heyd, J. J.; Brothers, E.; Kudin, K. N.; Staroverov, V. N.; Kobayashi, R.; Normand, J.; Raghavachari, K.; Rendell, A.; Burant, J. C.; Iyengar, S. S.; Tomasi, J.; Cossi, M.; Rega, N.; Millam, J. M.; Klene, M.; Knox, J. E.; Cross, J. B.; Bakken, V.; Adamo, C.; Jaramillo, J.; Gomperts, R.; Stratmann, R. E.; Yazyev, O.; Austin, A. J.; Cammi, R.; Pomelli, C.; Ochterski, J. W.; Martin, R. L.; Morokuma, K.; Zakrzewski, V. G.; Voth, G. A.; Salvador, P.; Dannenberg, J. J.; Dapprich, S.; Daniels, A. D.; Farkas, Ö.; Foresman, J. B.; Ortiz, J. V.; Cioslowski, J.; Fox, D. J. Gaussian, Inc., Wallingford CT, 2009.

(17) (a) Becke, A. D. Density-Functional Thermochemistry. III. The Role of Exact Exchange. *J. Chem. Phys.* **1993**, *98*, 5648-5652. (b) Lee, C. T.; Yang, W. T.; Parr, R. G. Development of the Colle-Salvetti Correlation-Energy Formula into a Functional of the Electron Density *Phys. Rev. B* **1988**, *37*, 785-789.

(**18**) https://bse.pnl.gov/bse/portal. (Accessed Mach 4, 2021).

(**19**) Schäfer, A.; Horn, H.; Ahlrichs, R. Fully Optimized Contracted Gaussian Basis Sets for Atoms Li to Kr. *J. Chem. Phys.* **1992**, *97*, 2571-2577.

(**20**) Chemcraft, Version 1.8 (build 445); http://chemcraftprog.com (accessed March 4, 2021).

Table S1. Unit Cell and Refinement Data for Compounds **1**, **4**, **5** and **7**.

compound	[Cl_2Ni(tpbz)]	[(mnt)Ni(tpbz)]	[(mdt)Ni(tpbz)]	[(mdt)Pt(tpbz)]
compound #	**1**	**4**	**5**	**7**
structure #	JPD412	JPD267	JPD751	JPD150
solvent/cocrystallite	Et_2O	$2CHCl_3$	$2CH_2Cl_2$	$2CH_2Cl_2$
formula	$C_{58}H_{52}Cl_2NiOP_4$	$C_{60}H_{44}Cl_6N_2NiP_4S_2$	$C_{60}H_{52}Cl_4NiP_4S_2$	$C_{60}H_{52}Cl_4P_4PtS_2$
fw, g/mol	1018.49	1252.38	1161.52	1297.91
temperature, K	100	100	100	100
wavelength, Å	0.71073	0.71073	0.71073	0.71073
2θ range, deg.	2.34 – 55.70	4.22 – 56.74	3.24 – 60.44	3.22 – 56.56
crystal system	orthorhombic	orthorhombic	monoclinic	monoclinic
space group	$Pna2_1$	$P2_12_12_1$	$C2/c$	$C2/c$
a, Å	34.703(3)	13.510(3)	21.223(2)	21.157(5)
b, Å	9.2939(9)	16.403(3)	15.7180(18)	15.836(4)
c, Å	15.5064(14)	25.380(5)	16.7123(18)	16.576(4)
α, deg.	90	90	90	90
β, deg.	90	90	100.039(2)	99.604(4)
γ, deg.	90	90	90	90
volume, $Å^3$	5001.2(8)	5624.2(19)	5489.6(11)	5476(2)
Z	4	4	4	4
density, g/cm^3	1.353	1.479	1.405	1.574
μ, mm^{-1}	0.664	0.860	0.780	2.991
crystal size	0.02 x 0.12 x 0.32	0.09 x 0.14 x 0.17	0.04 x 0.09 x 0.47	0.05 x 0.15 x 0.24
color, habit	orange plate	orange block	brwn-grn column	yellow plate
limiting indices, *h*	$-44 < h < 45$	$-17 < h < 18$	$-29 \leq h \leq 29$	$-28 < h < 27$
limiting indices, *k*	$-12 < k < 12$	$-21 < k < 21$	$-22 \leq k \leq 22$	$-20 < k < 20$
limiting indices, *l*	$-20 < l < 19$	$-33 < l < 33$	$-23 \leq l \leq 23$	$-21 < l < 21$
reflections collected	40918	100119	52873	24025
independent data	11569	14060	7799	6526
restraints	1	0	0	0
parameters refined	597	676	322	322
GooF[a]	1.090	1.048	1.105	1.035
R1,[b,c] wR2[d,c]	0.0574, 0.1259	0.0413, 0.1087	0.0450, 0.1434	0.0308, 0.0750
R1,[b,e] wR2[d,e]	0.0725, 0.1327	0.0481, 0.1141	0.0590, 0.1468	0.0360, 0.0776
largest diff. peak, e·$Å^{-3}$	0.734	0.650	2.930	1.601
largest diff. hole, e·$Å^{-3}$	–0.755	–1.153	–0.572	–0.790
abs structure parameter	0.013(15)	–0.012(11)	-	-

[a]GooF = $\{\Sigma[w(F_o^2 - F_c^2)^2]/(n - p)\}^{½}$, where n = number of reflections and p is the total number of parameters refined; [b]R1 = $\Sigma||F_o| - |F_c||/\Sigma|F_o|$; [c]R indices for data cut off at I > 2σ(I); [d]wR2 = $\{\Sigma[w(F_o^2 - F_c^2)^2]/\Sigma[w(F_o^2)^2]\}^{1/2}$; $w = 1/[\sigma^2(F_o^2) + (xP)^2 + yP]$, where $P = [2F_c^2 + \mathrm{Max}(F_o^2,0)]/3$; [e]R indices for all data.

Table S2. Unit Cell and Refinement Data for Compounds **8**, **9**, **11**, and **10**.

compound	[(pdt)Ni(tpbz)]	[(pdt)Pd(tpbz)]	[(pdt)Pt(tpbz)]	[$(pdt)_2$Pt(tpbz)]
compound #	**8**	**9**	**11**	**10**
structure #	JPD659	JPD757	JPD798	JPD736
solvent/cocrystallite	none	none	none	2($ClCH_2CH_2Cl$)
formula	$C_{68}H_{52}NiP_4S_2$	$C_{68}H_{52}P_4PdS_2$	$C_{68}H_{52}P_4PtS_2$	$C_{86}H_{70}Cl_4P_4PtS_4$
fw, g/mol	1115.80	1163.49	1252.18	1692.43
temperature, K	150	100	100	100
wavelength, Å	1.54178	0.71073	0.71073	0.71073
2θ range, deg.	6.84 – 148.77	3.14 – 58.44	3.14 – 57.39	3.03 – 59.72
crystal system	monoclinic	monoclinic	monoclinic	triclinic
space group	*C*2/*c*	*C*2/*c*	*C*2/*c*	*P*-1
a, Å	21.9519(4)	21.7574(15)	21.7804(15)	13.9324(12)
b, Å	16.7831(3)	16.9716(12)	16.9661(12)	14.0989(12)
c, Å	16.4070(3)	16.2670(11)	16.2774(12)	21.2092(18)
α, deg.	90	90	90	89.716(1)
β, deg.	112.864(1)	112.666(1)	112.727(1)	84.452(1)
γ, deg.	90	90	90	72.605(1)
volume, $Å^3$	5569.75(18)	5542.8(7)	5547.9(7)	3955.7(6)
Z	4	4	4	2
density, g/cm^3	1.331	1.394	1.499	1.421
μ, mm^{-1}	2.612	0.569	2.763	2.139
crystal size	0.11 x 0.14 x 0.15	0.07 x 0.08 x 0.28	0.04 x 0.05 x 0.14	0.10 x 0.20 x 0.37
color, habit	yellow block	pale orange column	yellow column	dark blue block
limiting indices, *h*	$-27 \le h \le 27$	$-29 \le h \le 28$	$-29 \le h \le 28$	$-19 \le h \le 19$
limiting indices, *k*	$-20 \le k \le 20$	$-23 \le k \le 23$	$-22 \le k \le 22$	$-19 \le k \le 19$
limiting indices, *l*	$-20 \le l \le 20$	$-22 \le l \le 22$	$-21 \le l \le 21$	$-29 \le l \le 29$
reflections collected	35518	26293	26168	77174
independent data	5650	7084	7003	21625
restraints	0	0	0	2
parameters refined	339	339	339	909
GooF[a]	1.063	1.074	1.006	1.071
R1,[b,c] wR2[d,c]	0.0340, 0.0918	0.0408, 0.1122	0.0362, 0.0836	0.0379, 0.1019
R1,[b,e] wR2[d,e]	0.0376, 0.0952	0.0558, 0.1179	0.0487, 0.0870	0.0433, 0.1044
largest diff. peak, e·$Å^{-3}$	0.492	1.247	1.883	3.844
largest diff. hole, e·$Å^{-3}$	–0.199	–0.457	–0.995	–2.215
abs structure parameter	-	-	-	-

[a]GooF = $\{\Sigma[w(F_o^2 - F_c^2)^2]/(n - p)\}^{½}$, where *n* = number of reflections and *p* is the total number of parameters refined; [b]R1 = $\Sigma||F_o| - |F_c||/\Sigma|F_o|$; [c]R indices for data cut off at I > 2σ(I); [d]wR2 = $\{\Sigma[w(F_o^2 - F_c^2)^2]/\Sigma[w(F_o^2)^2]\}^{1/2}$; $w = 1/[\sigma^2(F_o^2) + (xP)^2 + yP]$, where $P = [2F_c^2 + \mathrm{Max}(F_o^2,0)]/3$; [e]R indices for all data.

Table S3. Unit Cell and Refinement Data for Compounds **12**, **15**, and **16**.

compound	[(adt)Ni(tpbz)]	[(pdt)Pt(tpbz)Ni(mdt)]	[(pdt)Ni(tpbzO$_2$)]
compound #	**12**	**15**	**16**
structure #	JPD950	JPD1139	JPD1132
solvent	none	2½($ClCH_2CH_2Cl$)	CH_2Cl_2
formula	$C_{70}H_{56}NiO_2P_4S_2$	$C_{77}H_{68}Cl_5NiP_4PtS_4$	$C_{69}H_{54}Cl_2NiO_2P_4S_2$
fw, g/mol	1175.85	1676.48	1232.73
temperature, K	150	150	150
wavelength, Å	1.54178	0.71073	0.71073
2θ range, deg.	4.59 – 144.21	3.87 – 66.84	3.57 – 61.226
crystal system	monoclinic	triclinic	triclinic
space group	$P2_1/n$	P-1	P-1
a, Å	12.0422(4)	12.5374(6)	13.4505(18)
b, Å	38.5014(13)	12.5692(6)	16.597(2)
c, Å	14.0003(6)	24.7673(13)	16.653(2)
α, deg.	90	92.953(2)	64.068(4)
β, deg.	112.798(2)	91.560(2)	70.817(4)
γ, deg.	90	108.166(2)	72.092(4)
volume, Å^3	5984.0(4)	3699.6(3)	3098.4(7)
Z	4	2	2
density, g/cm^3	1.305	1.505	1.321
μ, mm^{-1}	2.484	2.565	0.615
crystal size	0.02 x 0.12 x 0.27	0.08 x 0.23 x 0.33	0.05 x 0.17 x 0.29
color, habit	yellow-grn plate	orange plate	orange plate
limiting indices, h	$-14 < h < 14$	$-19 < h < 19$	$-19 < h < 19$
limiting indices, k	$-44 < k < 46$	$-19 < k < 19$	$-23 < k < 23$
limiting indices, l	$-16 < l < 14$	$0 < l < 38$	$-23 < l < 23$
reflections collected	51648	32993	144627
independent data	11399	32993	19027
restraints	0	51	14
parameters refined	703	856	728
GooF^a	1.039	1.277	1.044
R1,^{b,c} wR2^{d,c}	0.0479, 0.1008	0.0751, 0.1691	0.0488, 0.1165
R1,^{b,e} wR2^{d,e}	0.0699, 0.1106	0.0827, 0.1717	0.0878, 0.1350
largest diff. peak, e·Å^{-3}	0.830	2.720	0.802
largest diff. hole, e·Å^{-3}	–0.388	–6.777	–0.915
abs structure parameter	-	-	-

^{a}GooF = $\{\Sigma[w(F_o^2 - F_c^2)^2]/(n-p)\}^{½}$, where n = number of reflections and p is the total number of parameters refined; ^{b}R1 = $\Sigma||F_o| - |F_c||/\Sigma|F_o|$; ^{c}R indices for data cut off at I > 2σ(I); ^{d}wR2 = $\{\Sigma[w(F_o^2 - F_c^2)^2]/\Sigma[w(F_o^2)^2]\}^{1/2}$; $w = 1/[\sigma^2(F_o^2) + (xP)^2 + yP]$, where $P = [2F_c^2 + \mathrm{Max}(F_o^2,0)]/3$; ^{e}R indices for all data.

Table S4. Unit Cell and Refinement Data for Compounds **17**, **18** and [**19**][I_3]$_2$.

compound	[(mdt)Pt(tpbzO$_2$)]	[(mdt)Ni(tpbzS$_2$)]	[(dppbO$_2$)$_3$Ni][I_3]$_2$	[(dppbO$_2$)$_3$Ni][I_3]$_2$
compound #	**17**	**18**	[**19**][I_3]$_2$	[**19**][I_3]$_2$
structure #	JPD1146	JPD184	JPD334	JPD1118
solvent	none	none	none	none
formula	$C_{58}H_{48}O_2P_4PtS_2$	$C_{58}H_{48}NiP_4S_4$	$C_{90}H_{72}I_6NiO_6P_6$	$C_{90}H_{72}I_6NiO_6P_6$
fw, g/mol	1160.05	1055.79	2255.41	2255.40
temperature, K	150	100	100	150
wavelength, Å	0.71073	0.71073	0.71073	0.71073
2θ range, deg.	4.18 – 66.45	2.46 – 49.52	3.12 – 54.00	3.628 – 46.660
crystal system	monoclinic	monoclinic	triclinic	monoclinic
space group	$P2_1/c$	$P2_1/c$	P-1	$P2_1/c$
a, Å	17.0751(11)	9.3284(12)	13.0484(12)	15.0552(10)
b, Å	16.0625(10)	30.386(4)	13.3478(12)	26.0258(18)
c, Å	20.5241(13)	19.858(3)	26.880(3)	22.6547(15)
α, deg.	90	90	102.137(1)	90
β, deg.	108.283(2)	98.487(2)	92.504(1)	101.217(2)
γ, deg.	90	90	106.961(1)	90
volume, Å^3	5345.0(6)	5567.1(13)	4350.1(7)	8707.1(10)
Z	4	4	2	4
density, g/cm^3	1.442	1.260	1.722	1.721
μ, mm^{-1}	2.864	0.649	2.514	2.512
crystal size	0.21 x 0.21 x 0.26	0.03 x 0.03 x 0.18	0.02 x 0.13 x 0.21	0.05 x 0.06 x 0.28
color, habit	dark orange column	yellow needle	orange plate	orange-red plate
limiting indices, h	$-26 \le h \le 26$	$-10 \le h \le 10$	$-16 \le h \le 16$	$-15 \le h \le 16$
limiting indices, k	$-24 \le k \le 24$	$-35 \le k \le 35$	$-17 \le k \le 17$	$-28 \le k \le 28$
limiting indices, l	$-31 \le l \le 31$	$-23 \le l \le 23$	$-34 \le l \le 34$	$-25 \le l \le 25$
reflections collected	403235	39326	37066	153572
independent data	20472	9510	18761	12505
restraints	0	1	0	36
parameters refined	606	610	985	989
GooF^a	1.081	0.964	0.945	1.023
R1,^{b,c} wR2^{d,c}	0.0239, 0.0594	0.0898, 0.1686	0.0427, 0.0931	0.0505, 0.1241
R1,^{b,e} wR2^{d,e}	0.0314, 0.0641	0.1753, 0.2007	0.0707, 0.1017	0.0794, 0.1403
largest diff. peak, e·Å^{-3}	2.242	0.873	1.226	2.552
largest diff. hole, e·Å^{-3}	–0.781	–0.405	–1.590	–1.094
abs structure parameter	-	-	-	-

^{a}GooF = $\{\Sigma[w(F_o^2 - F_c^2)^2]/(n-p)\}^{½}$, where n = number of reflections and p is the total number of parameters refined; ^{b}R1 = $\Sigma||F_o| - |F_c||/\Sigma|F_o|$; ^{c}R indices for data cut off at I > 2σ(I); ^{d}wR2 = $\{\Sigma[w(F_o^2 - F_c^2)^2]/\Sigma[w(F_o^2)^2]\}^{1/2}$; $w = 1/[\sigma^2(F_o^2) + (xP)^2 + yP]$, where $P = [2F_c^2 + \mathrm{Max}(F_o^2,0)]/3$; ^{e}R indices for all data.

Table S5. Selected bond lengths (Å) and angles (deg.) for the two polymorphs of [(dppbO$_2$)$_3$Ni][I_3]$_2$. Chemically identical values are averaged.^{a}

	Triclinic	Monoclinic
Ni–O	2.061[1]	2.060[2]
P–O	1.495[1]	1.493[2]
P–C^b	1.823[2]	1.828[3]
O–Ni–O$_{trans}$	173.26[7]	173.65[10]
O–Ni–O$_{cis,\mathrm{intraligand}}$	85.86[7]	86.47[10]
O–Ni–O$_{cis,\mathrm{interligand}}$	91.47[4]	91.28[6]
θ^c	58.7	55.6

^{a}Uncertainties are propagated according to Taylor, J. R. *An Introduction to Error Analysis*; 2nd ed.; University Science Books: Sausalito, CA, 1997, pp 73-77. ^{b}Carbon atom of central C_6H_4 arene ring. ^{c}Fold angle between NiO_2 plane and P_2C_2 mean plan of central arene ring.

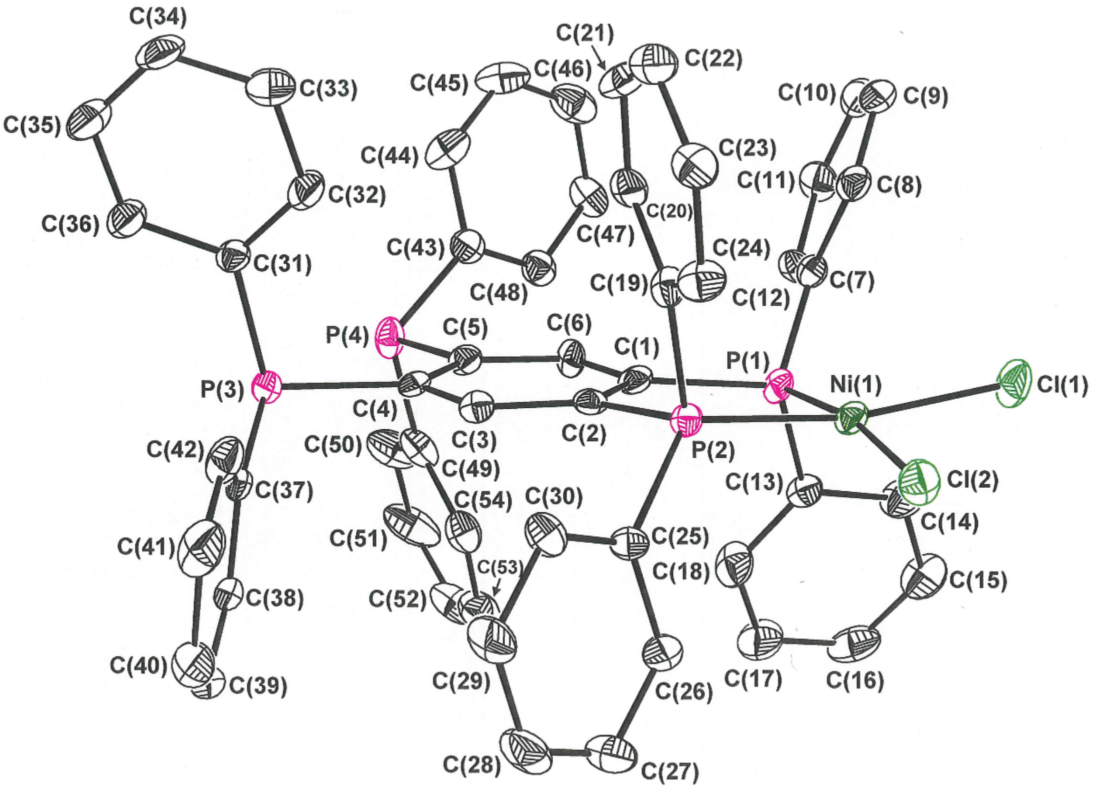

Figure S1. Full atom labeling for [(tpbz)$NiCl_2$]. The thermal ellipsoid plot is drawn at the 50% level, and all H atoms are omitted for clarity.

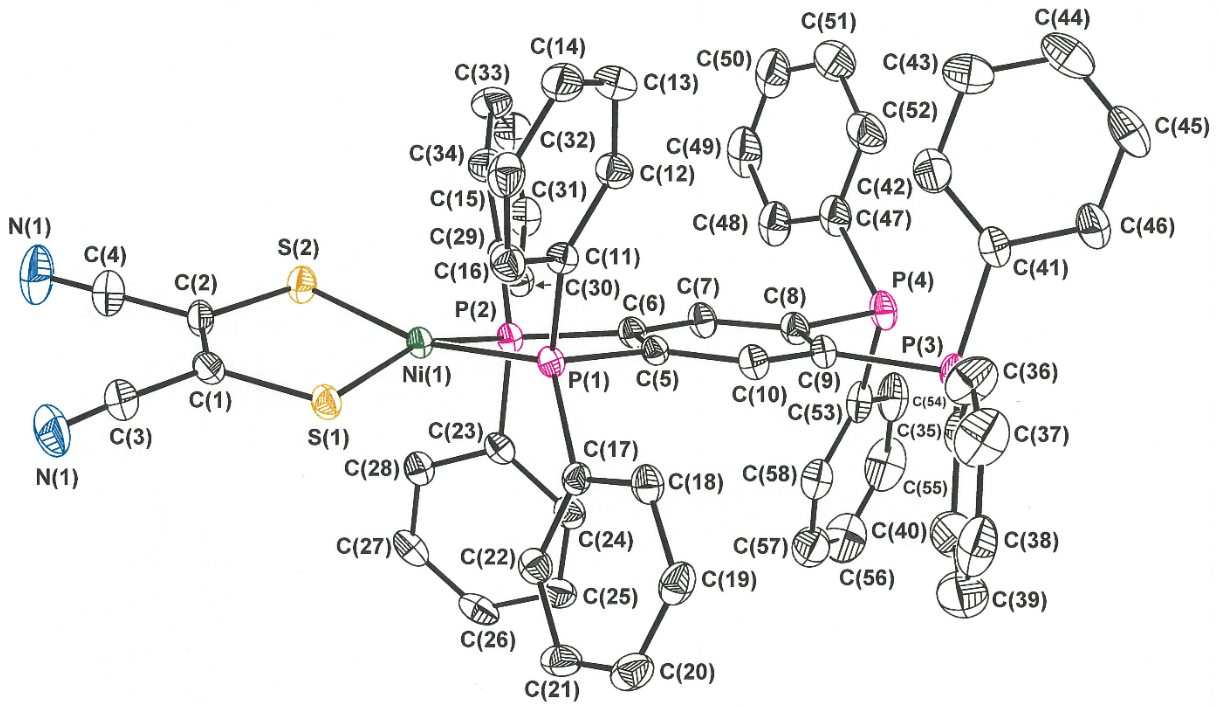

Figure S2. Full atom labeling for [((NC)$_2$C$_2$S$_2$)Ni(η^2-tpbz)]. The thermal ellipsoid plot is drawn at the 50% level, and all H atoms are omitted for clarity.

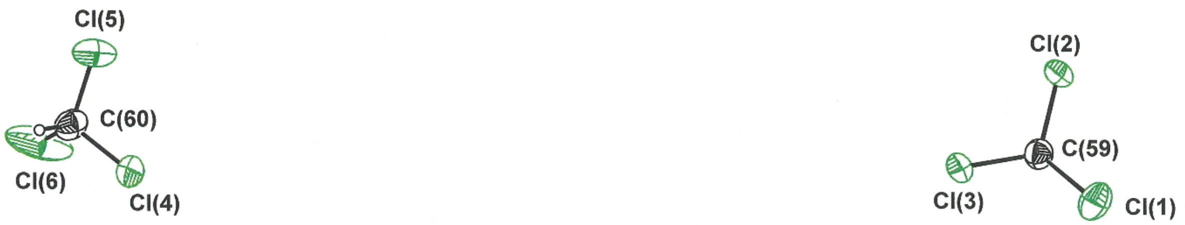

Figure S3. Atom labeling for interstitial CHCl$_3$ in [((NC)$_2$C$_2$S$_2$)Ni(η^2-tpbz)]·2(CHCl$_3$). The thermal ellipsoid plot is drawn at the 50% level.

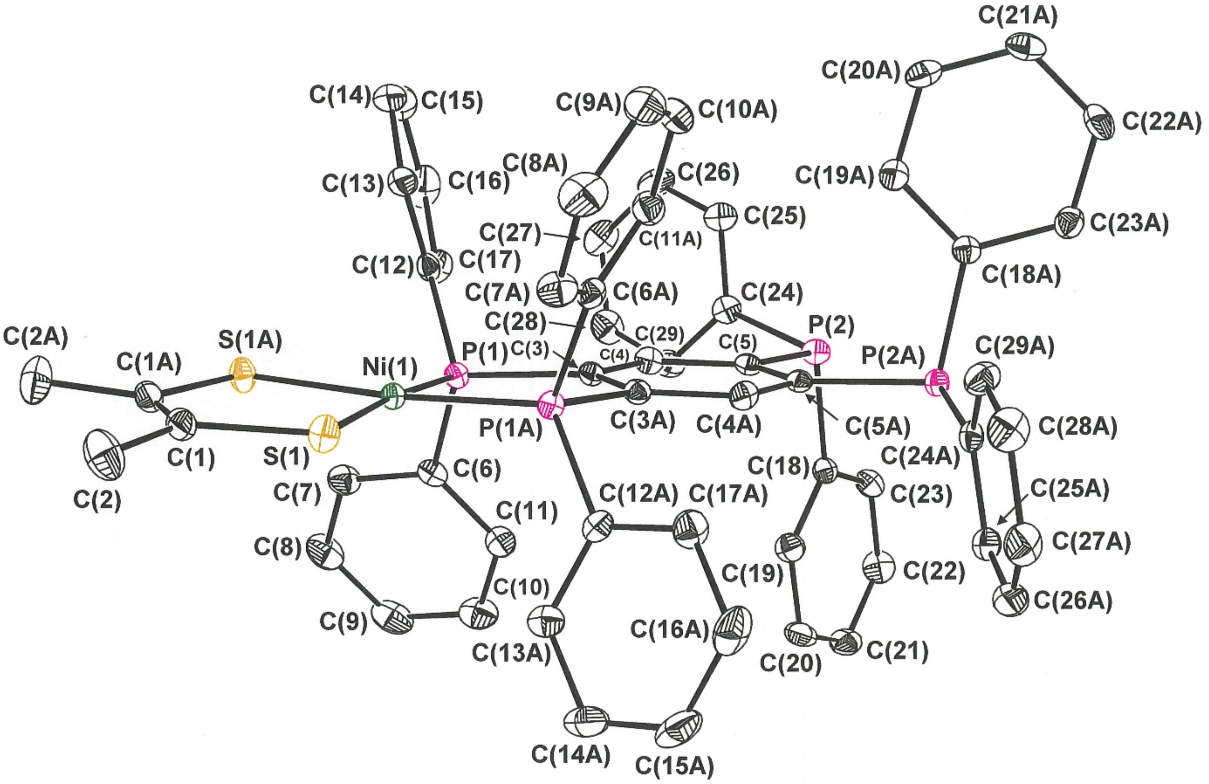

Figure S4. Full atom labeling for [$(Me_2C_2S_2)Ni(\eta^2$-tpbz)]. The thermal ellipsoid plot is drawn at the 50% level, and all H atoms are omitted for clarity.

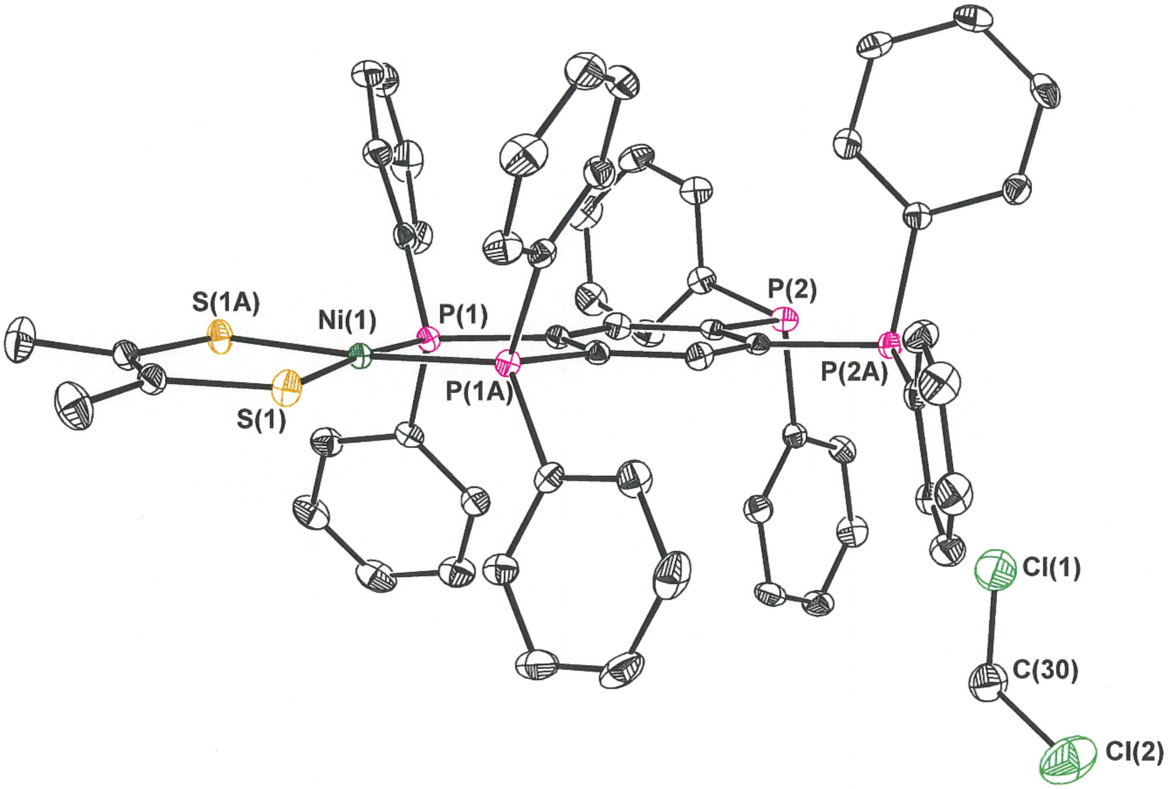

Figure S5. Thermal ellipsoid plot of [$(Me_2C_2S_2)Ni(\eta^2$-tpbz)] at the 50% level with interstitial CH_2Cl_2 molecule shown.

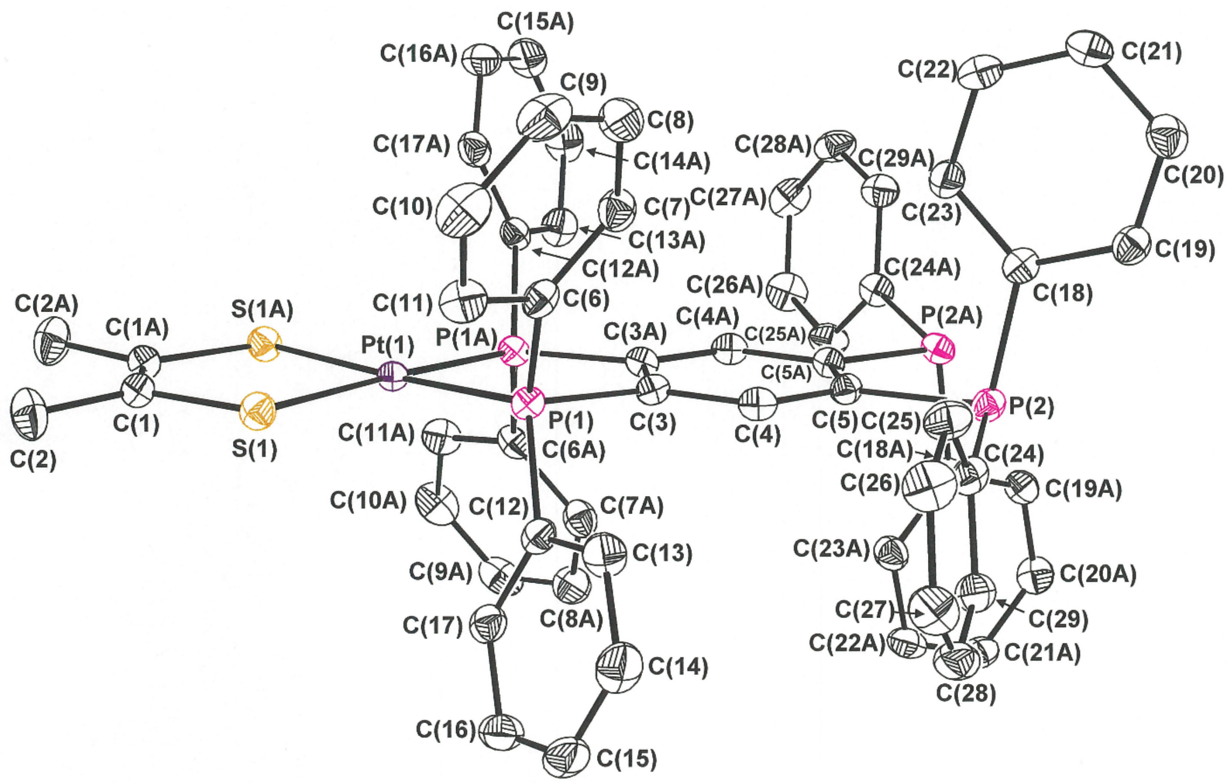

Figure S6. Full atom labeling for [($Me_2C_2S_2$)Pt(η^2-tpbz)]. The thermal ellipsoid plot is drawn at the 50% level, and all H atoms are omitted for clarity.

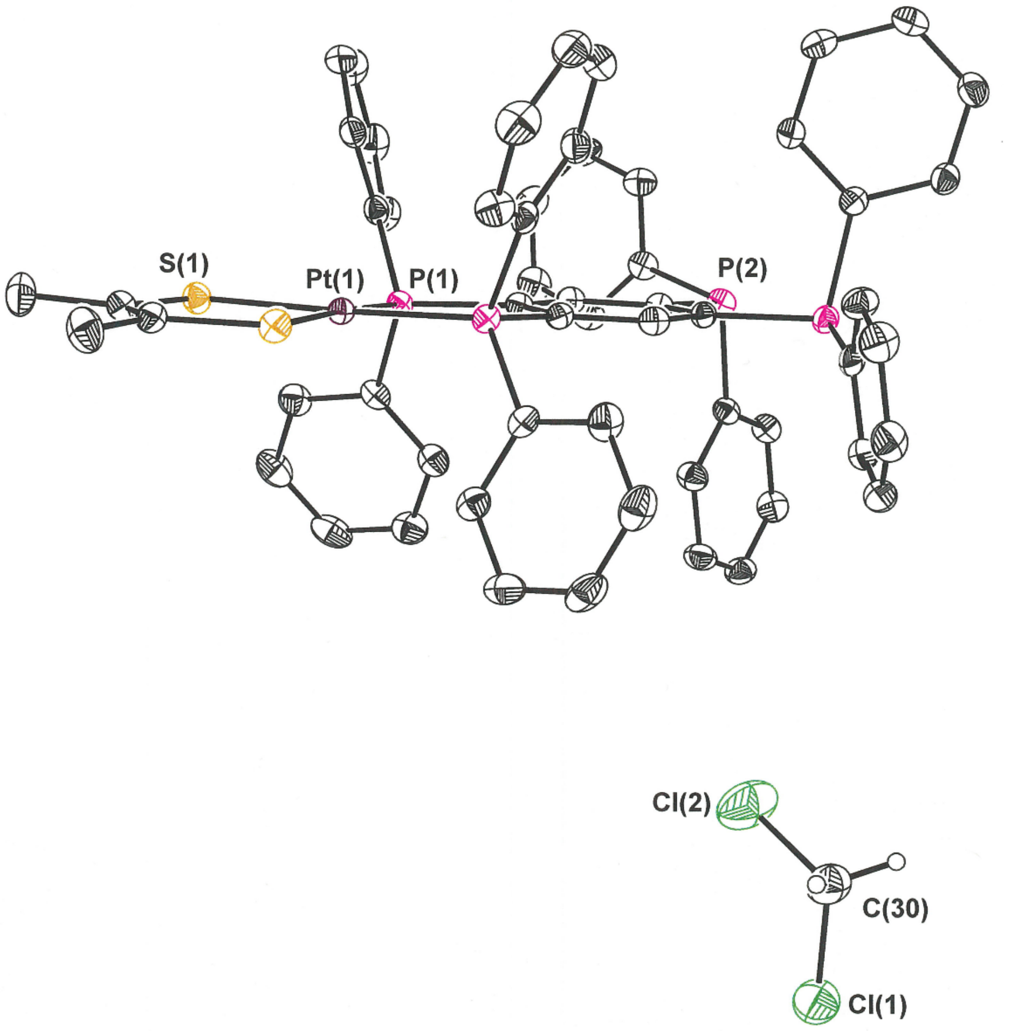

Figure S7. Atom labeling for interstitial CH_2Cl_2 in $[(Me_2C_2S_2)Pt(\eta^2\text{-tpbz})]\cdot 2(CH_2Cl_2)$. The thermal ellipsoid plot is drawn at the 50% level. The second CH_2Cl_2 molecule is symmetry-related to that which is shown.

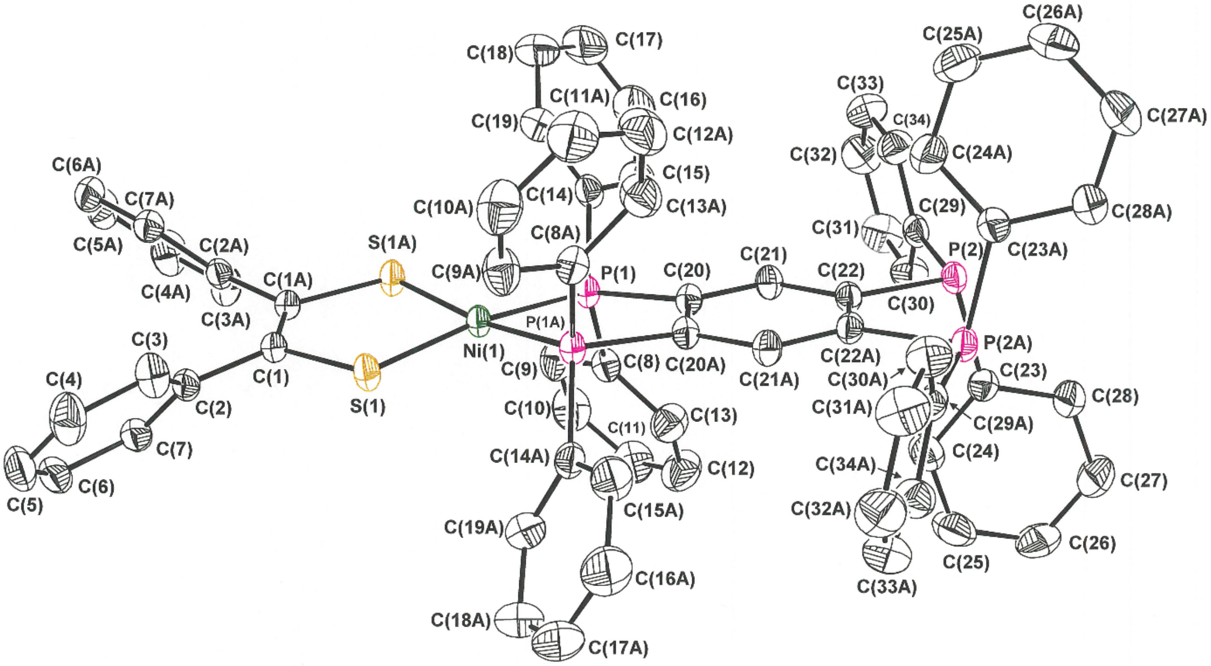

Figure S8. Full atom labeling for $[(Ph_2C_2S_2)Ni(\eta^2\text{-tpbz})]$. The thermal ellipsoid plot is drawn at the 50% level, and all H atoms are omitted for clarity.

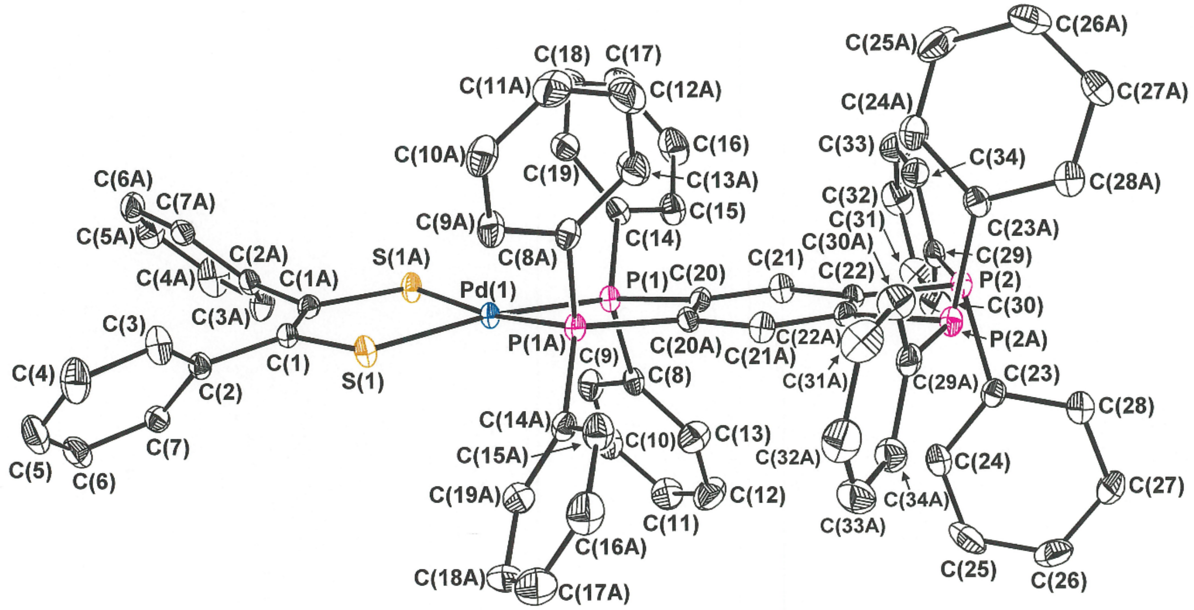

Figure S9. Full atom labeling for $[(Ph_2C_2S_2)Pd(\eta^2\text{-tpbz})]$. The thermal ellipsoid plot is drawn at the 50% level, and all H atoms are omitted for clarity.

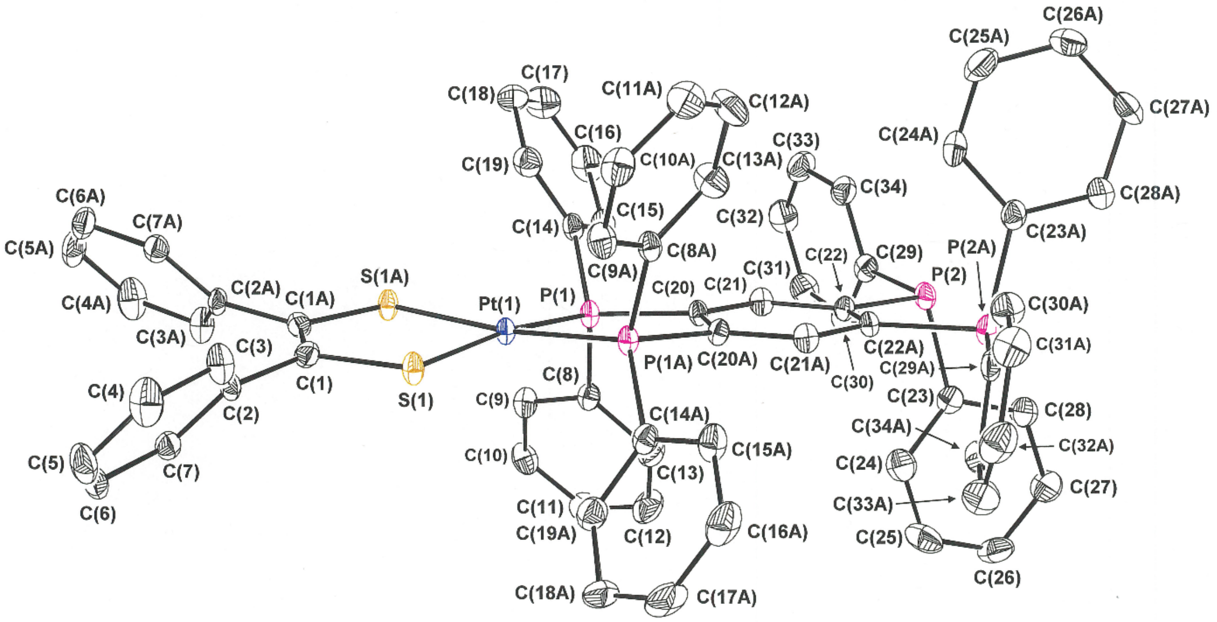

Figure S10. Full atom labeling for [($Ph_2C_2S_2$)Pt(η^2-tpbz)]. The thermal ellipsoid plot is drawn at the 50% level, and all H atoms are omitted for clarity.

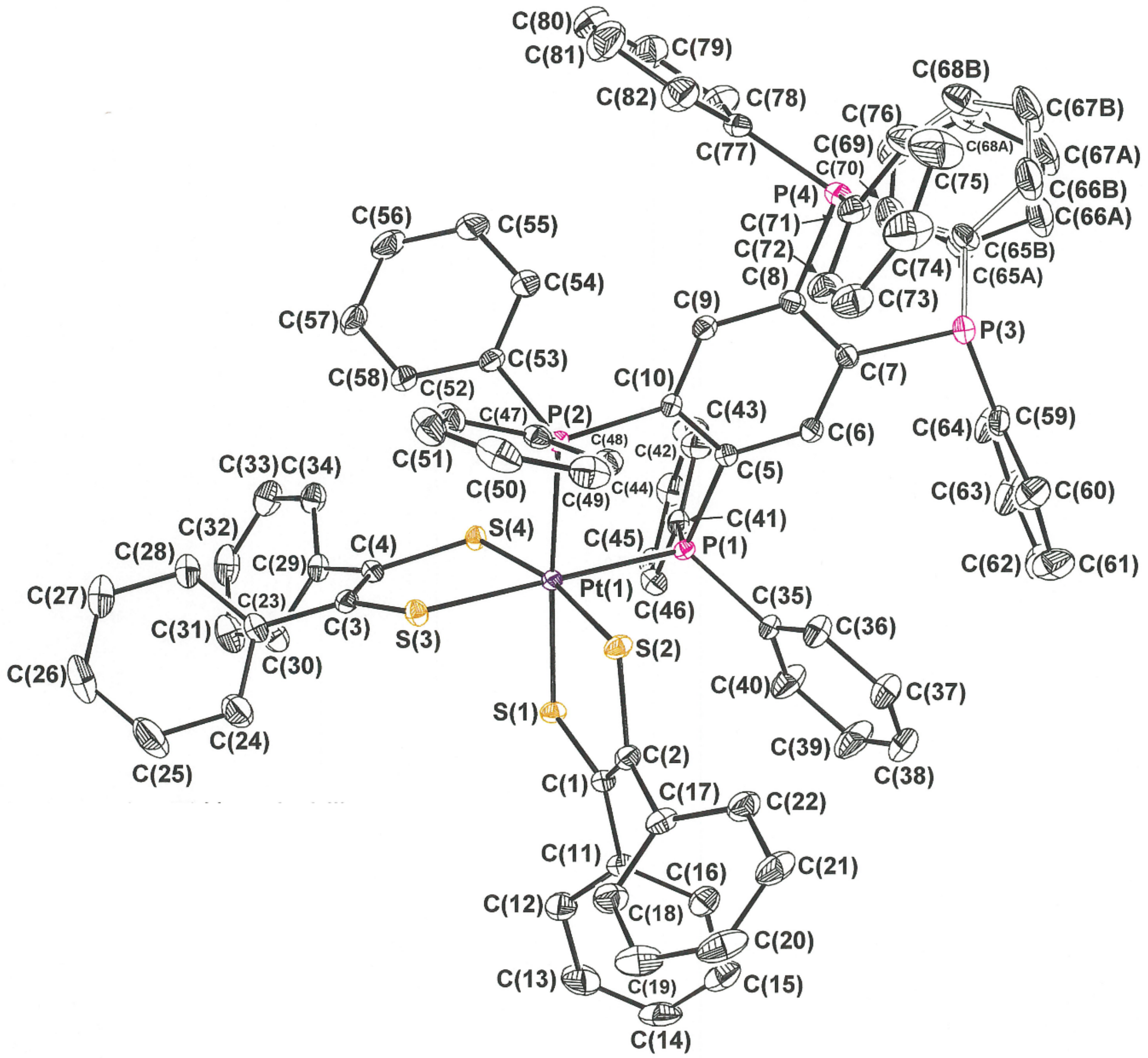

Figure S11. Full atom labeling for $[(Ph_2C_2S_2)_2Pt(\eta^2\text{-tpbz})]$. The thermal ellipsoid plot is drawn at the 50% level, and all H atoms are omitted for clarity.

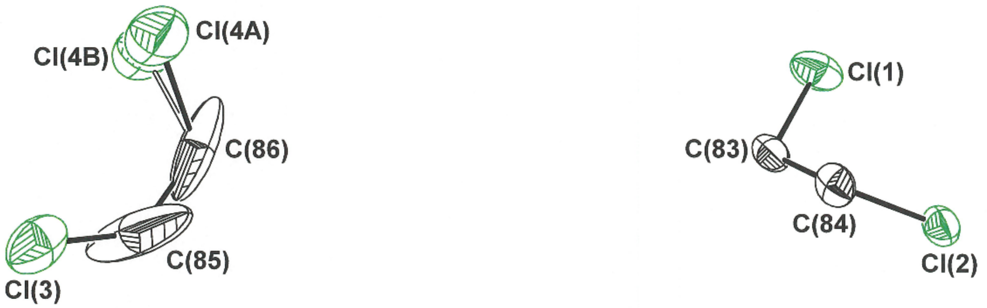

Figure S12. Full atom labeling for the interstitial $ClCH_2CH_2Cl$ molecules in $[(Ph_2C_2S_2)_2Pt(\eta^2\text{-tpbz})]\cdot 2(ClCH_2CH_2Cl)$. The thermal ellipsoid plot is drawn at the 50% level, and all H atoms are omitted for clarity. Chlorine atom Cl4 is disordered over two positions and refined with a split atom model.

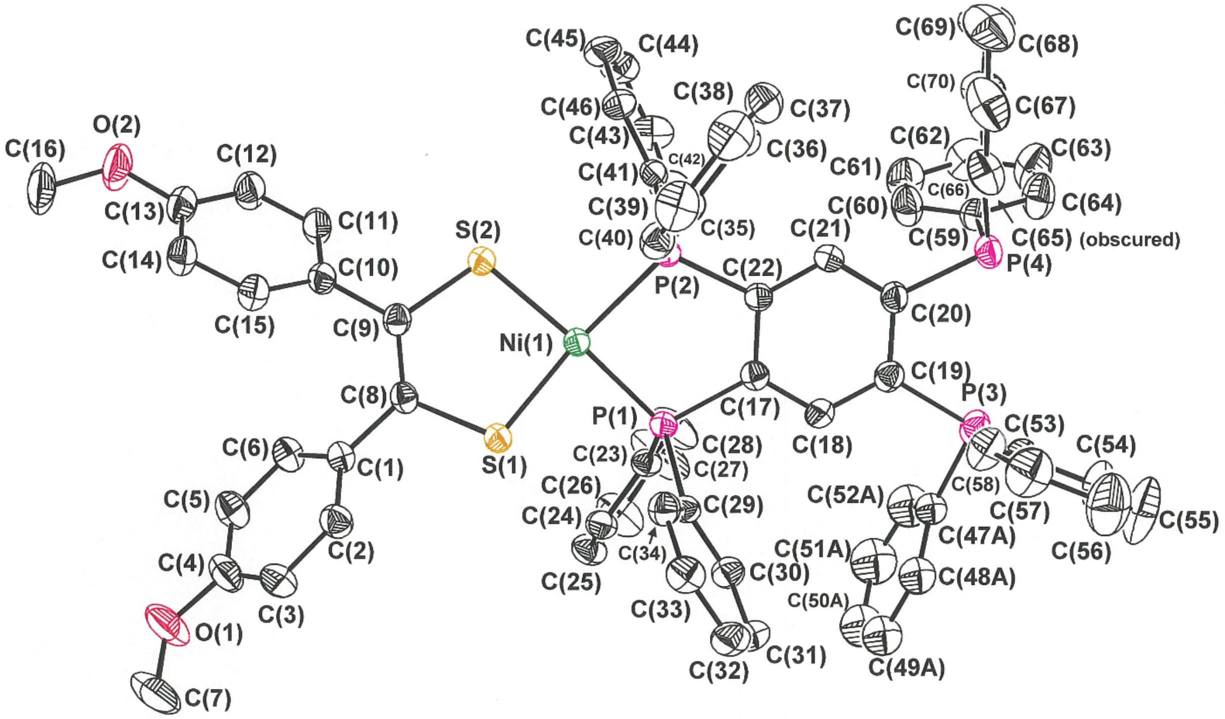

Figure S13. Full atom labeling for $[(CH_3O\text{-}p\text{-}C_6H_4)_2C_2S_2)Ni(\eta^2\text{-tpbz})]$. The thermal ellipsoid plot is drawn at the 50% level, and all H atoms are omitted for clarity. For the phenyl ring defined by C47-C52, one position of two over which it is disordered is shown.

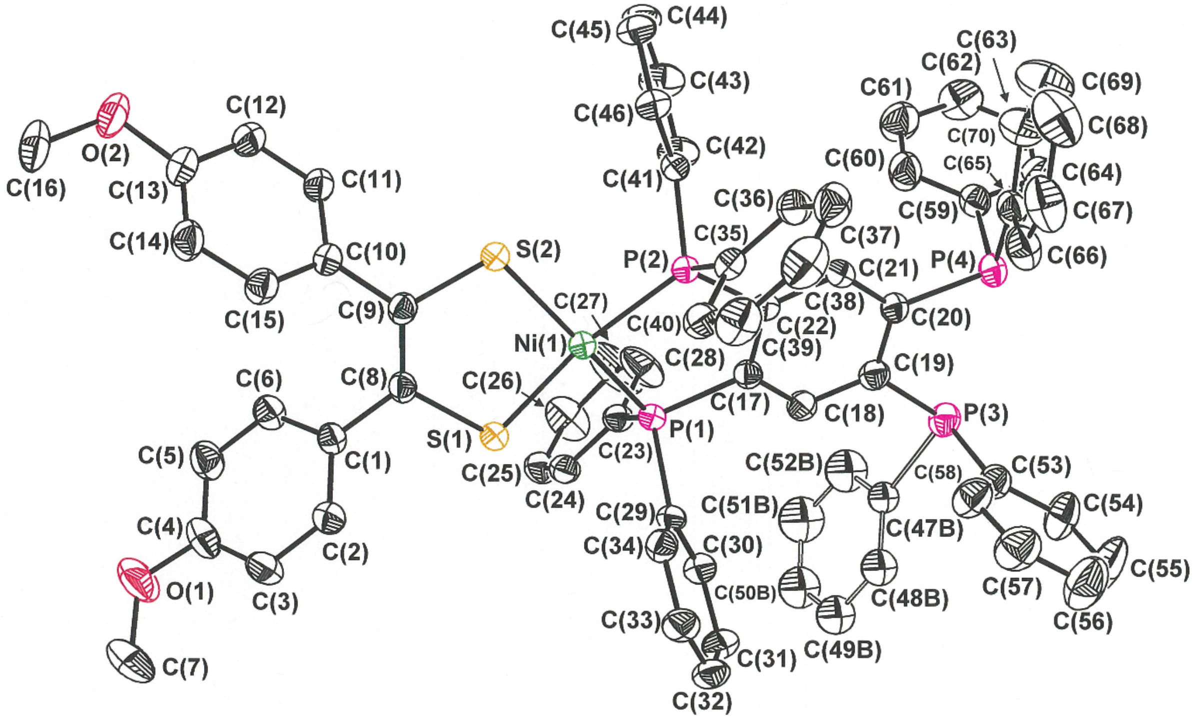

Figure S14. Full atom labeling for $[(CH_3O\text{-}p\text{-}C_6H_4)_2C_2S_2)Ni(\eta^2\text{-tpbz})]$. The thermal ellipsoid plot is drawn at the 50% level, and all H atoms are omitted for clarity. For the phenyl ring defined by C47-C52, the second position of two over which it is disordered is shown.

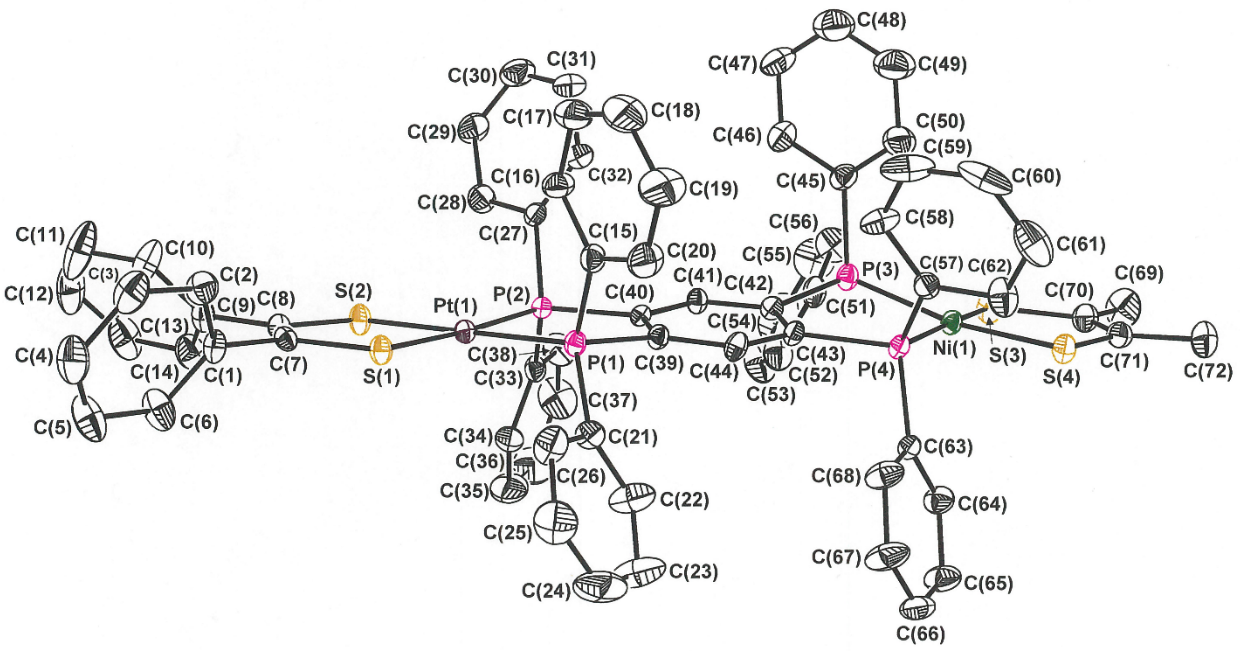

Figure S15. Full atom labeling for [$(Ph_2C_2S_2)Pt(tpbz)Ni(S_2C_2Me_2)$]. The thermal ellipsoid plot is drawn at the 50% level, and all H atoms are omitted for clarity.

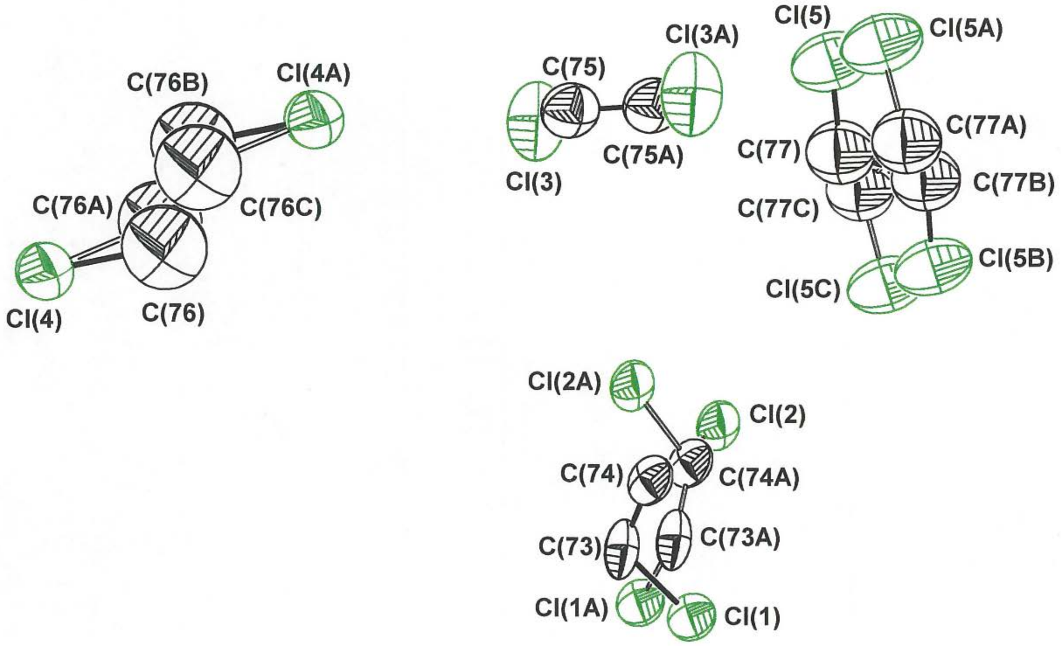

Figure S16. Full atom labeling for the interstitial solvent in [$(Ph_2C_2S_2)Pt(tpbz)Ni(S_2C_2Me_2)$]·2½($ClCH_2CH_2Cl$). The thermal ellipsoid plot is drawn at the 50% level, and all H atoms are omitted for clarity. The atoms of these solvent molecules have been handled with a mixture of anisotropic and isotropic refinement owing to the positional disorder that they show.

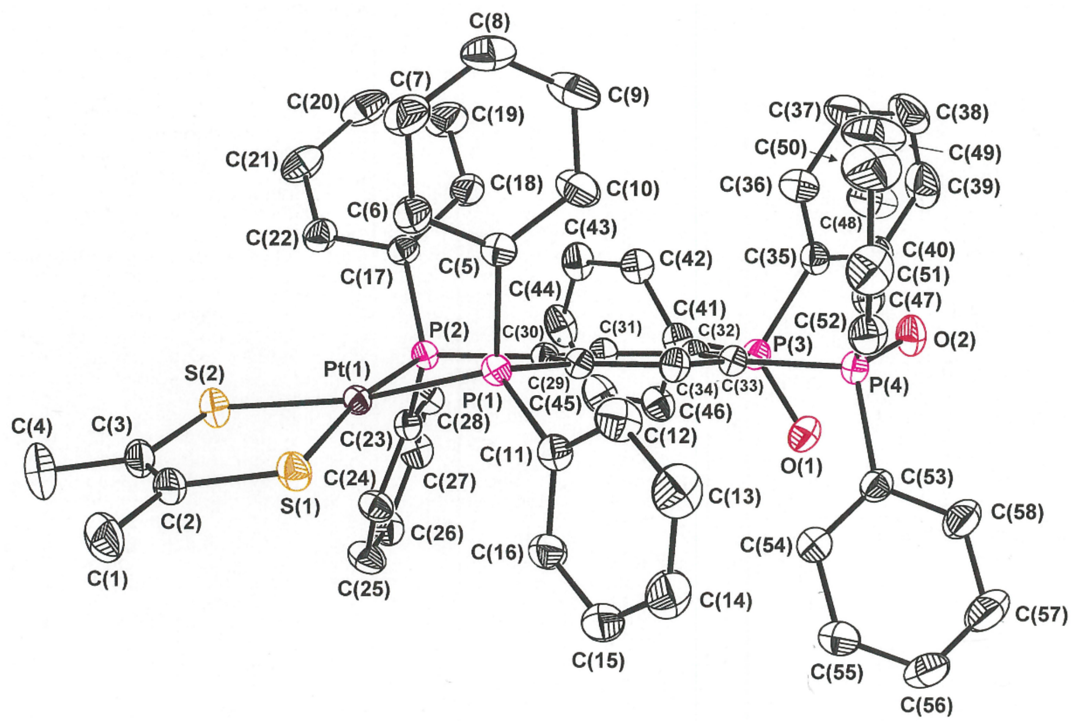

Figure S17. Full atom labeling for [$(Me_2C_2S_2)Pt(\eta^2\text{-}tpbzO_2)$]. The thermal ellipsoid plot is drawn at the 50% level, and all H atoms are omitted for clarity.

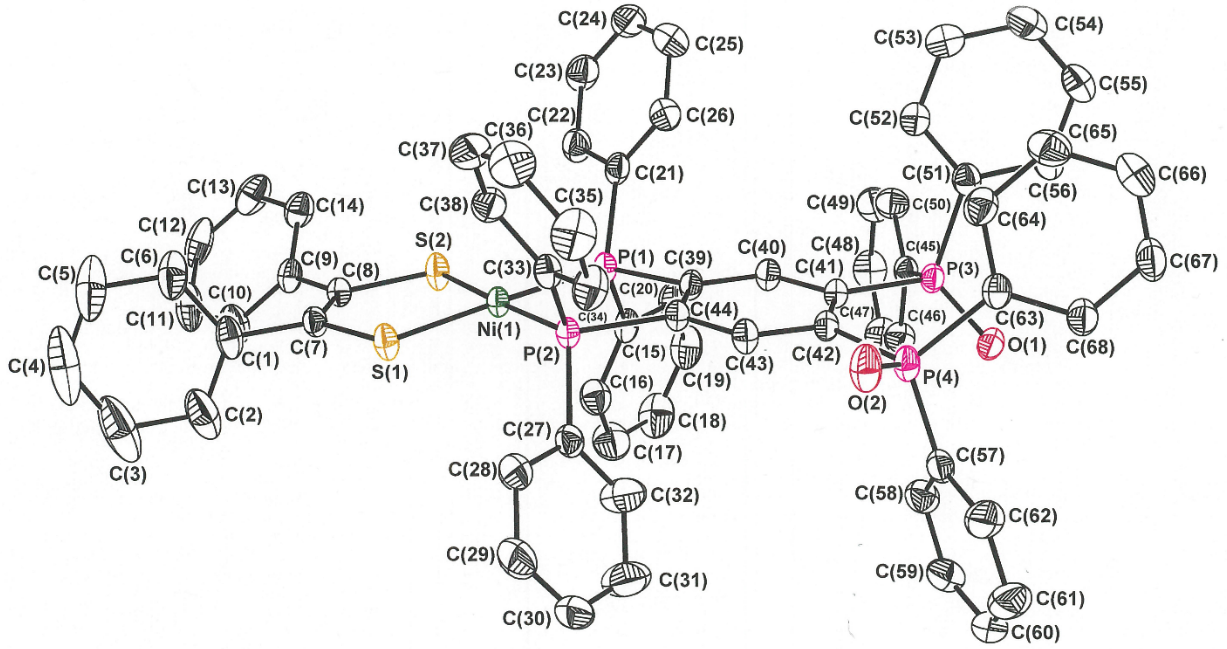

Figure S18. Full atom labeling for $[(Ph_2C_2S_2)Ni(\eta^2\text{-tpbzO}_2)]$. The thermal ellipsoid plot is drawn at the 50% level, and all H atoms are omitted for clarity.

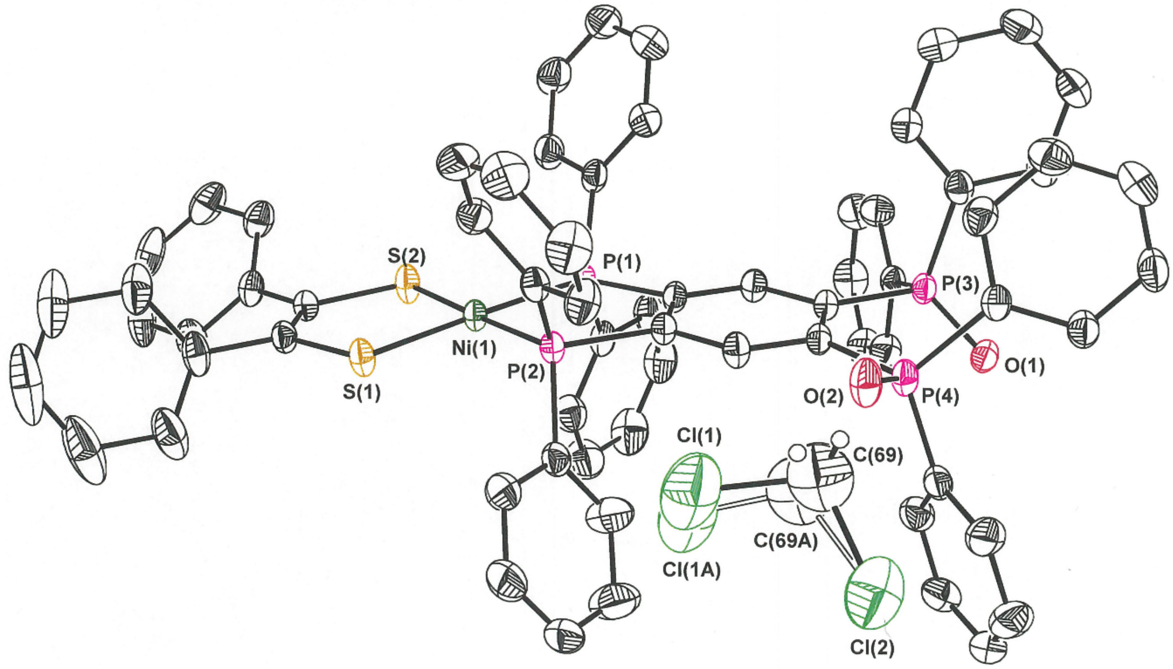

Figure S19. Atom labeling for interstitial CH_2Cl_2 in $[(Ph_2C_2S_2)Ni(\eta^2\text{-tpbzO}_2)]\cdot CH_2Cl_2$). The thermal ellipsoid plot is drawn at the 50% level. Chlorine atom 1 and carbon atom 69 are disordered over two positions and consequently refined using a split atom model.

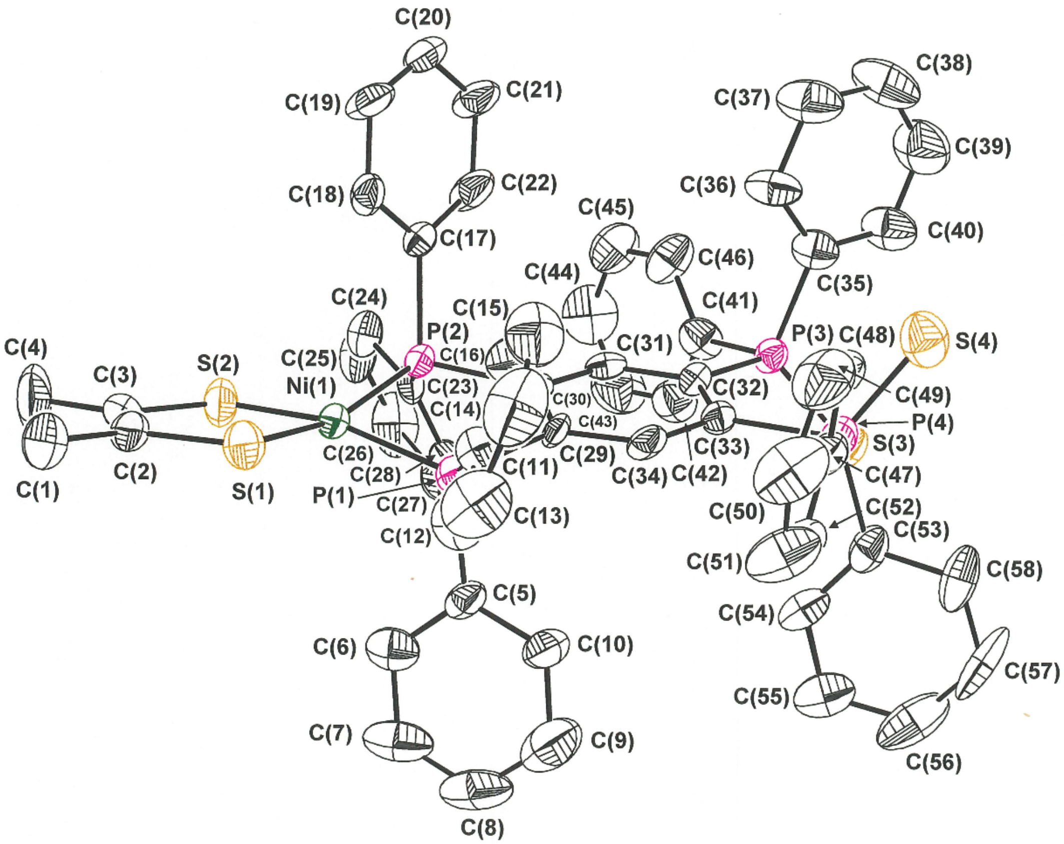

Figure S20. Full atom labeling for [($Me_2C_2S_2$)Ni(η^2-tpbzS_2)]. The thermal ellipsoid plot is drawn at the 50% level, and all H atoms are omitted for clarity.

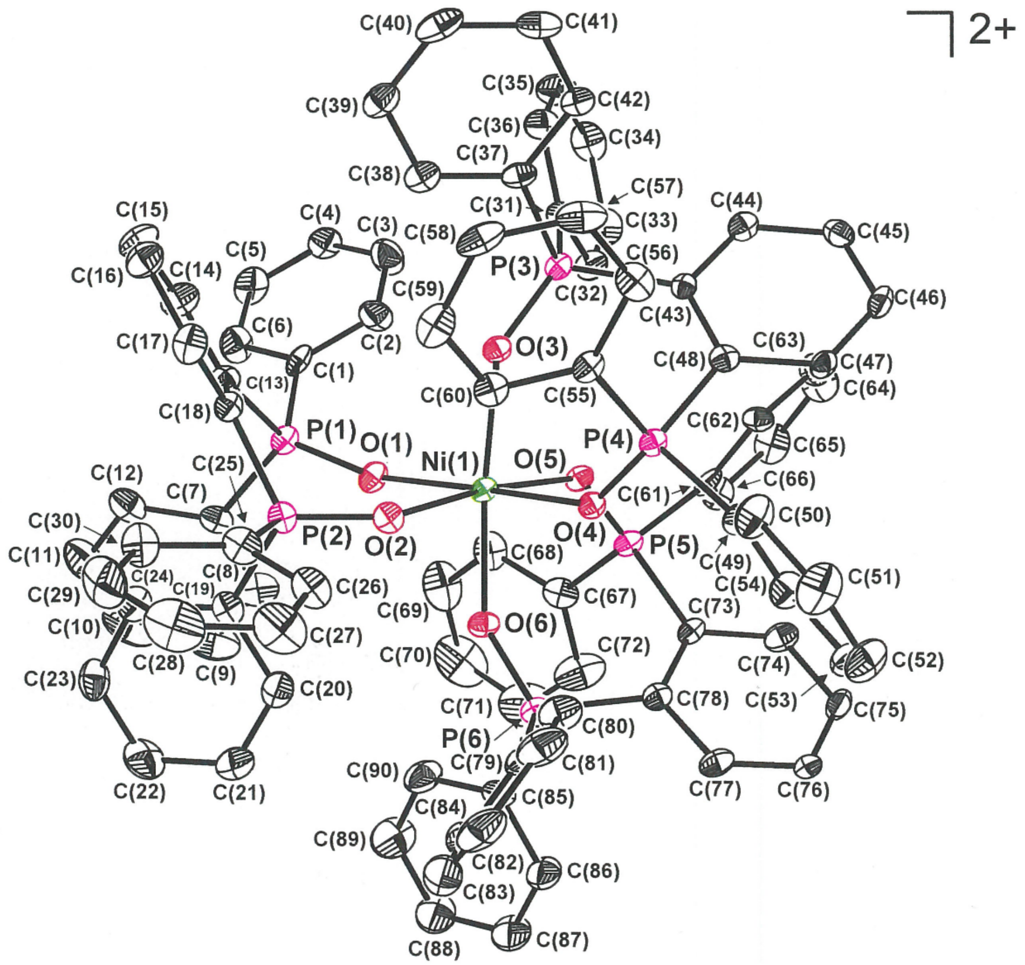

Figure S21. Full atom labeling for $[Ni(dppbO_2)_3]^{2+}$ in $[Ni(dppbO_2)_3][I_3]_2$ (triclinic polymorph). The thermal ellipsoid plot is drawn at the 50% level, and all H atoms are omitted for clarity.

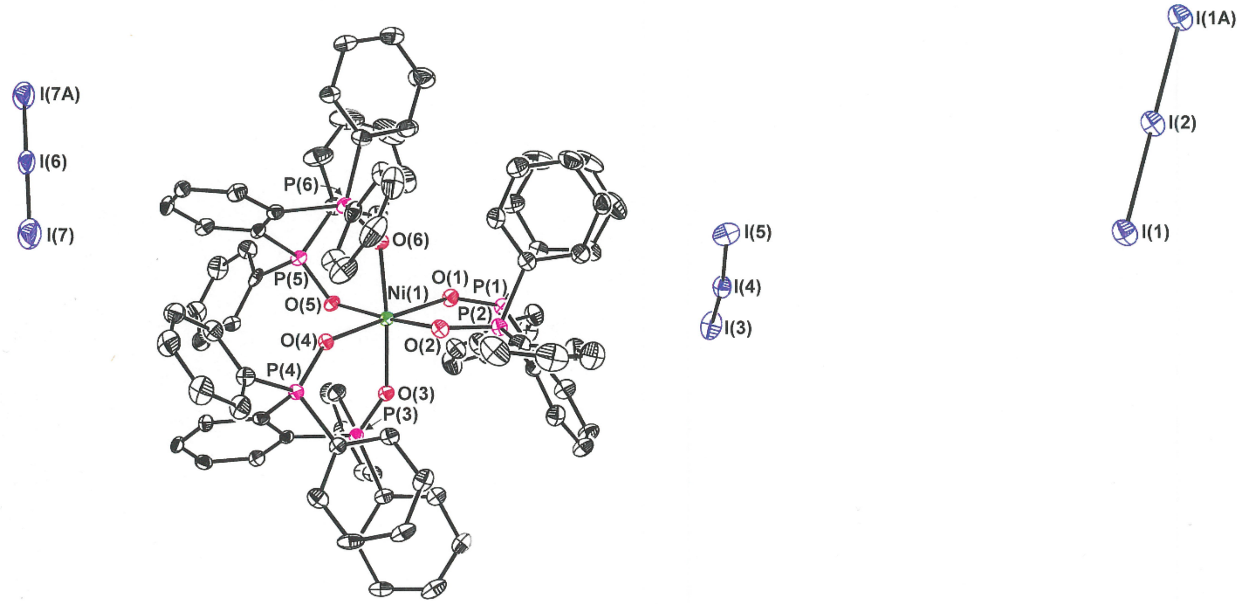

Figure S22. Partial atom labeling for $[Ni(dppbO_2)_3][I_3]_2$ (triclinic polymorph) with counteranions shown. One full I_3^- anion and two half I_3^- anions are paired with the dication. The thermal ellipsoid plot is drawn at the 50% level, and all H atoms are omitted for clarity.

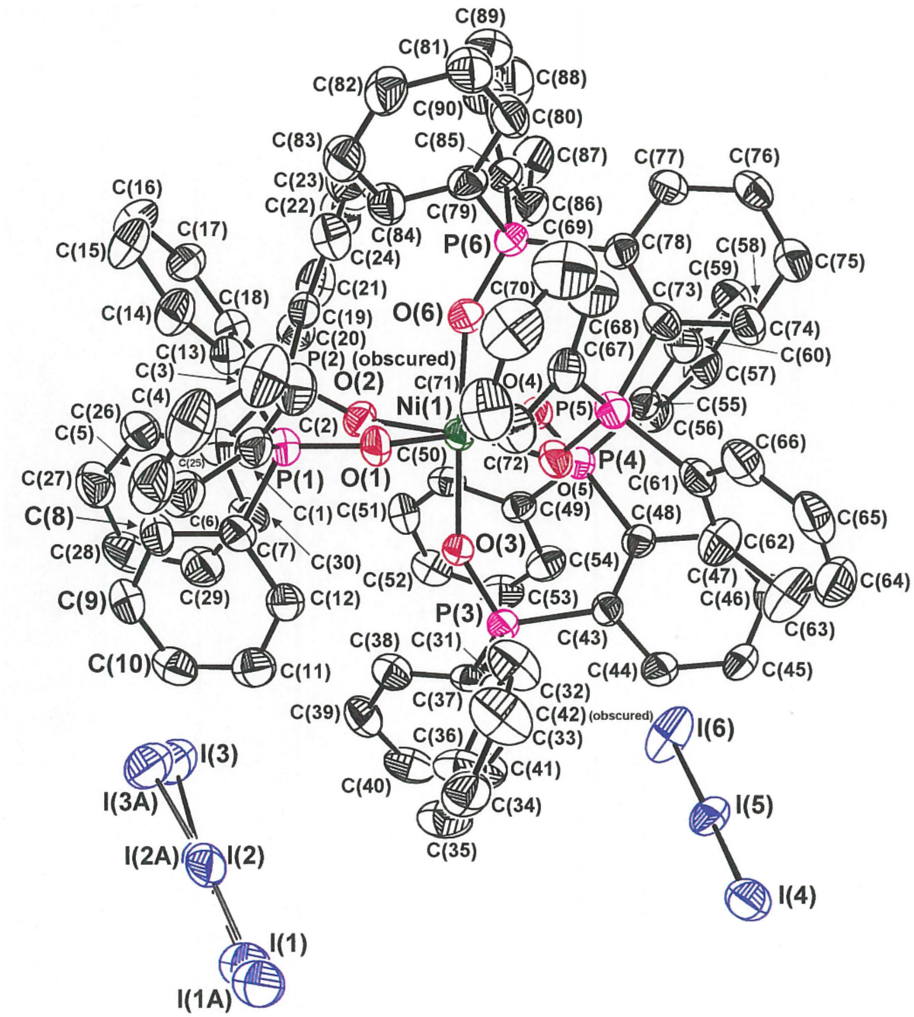

Figure S23. Full atom labeling for $[Ni(dppbO_2)_3]^{2+}$ in $[Ni(dppbO_2)_3][I_3]_2$ (monoclinic polymorph). The thermal ellipsoid plot is drawn at the 50% level, and all H atoms are omitted for clarity.

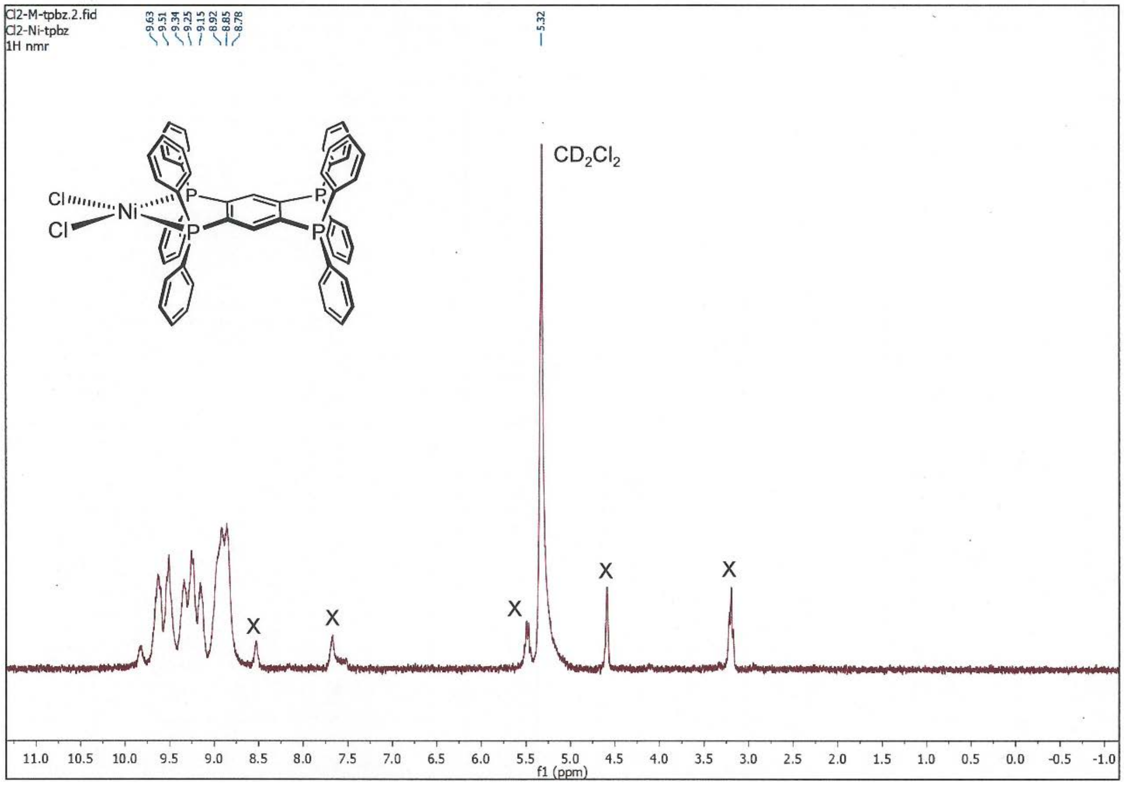

Figure S24. ^{1}H NMR spectrum of [Cl_2Ni(tpbz)] in CD_2Cl_2. Signals not corresponding to [Cl_2Ni(tpbz)] are marked with “x”.

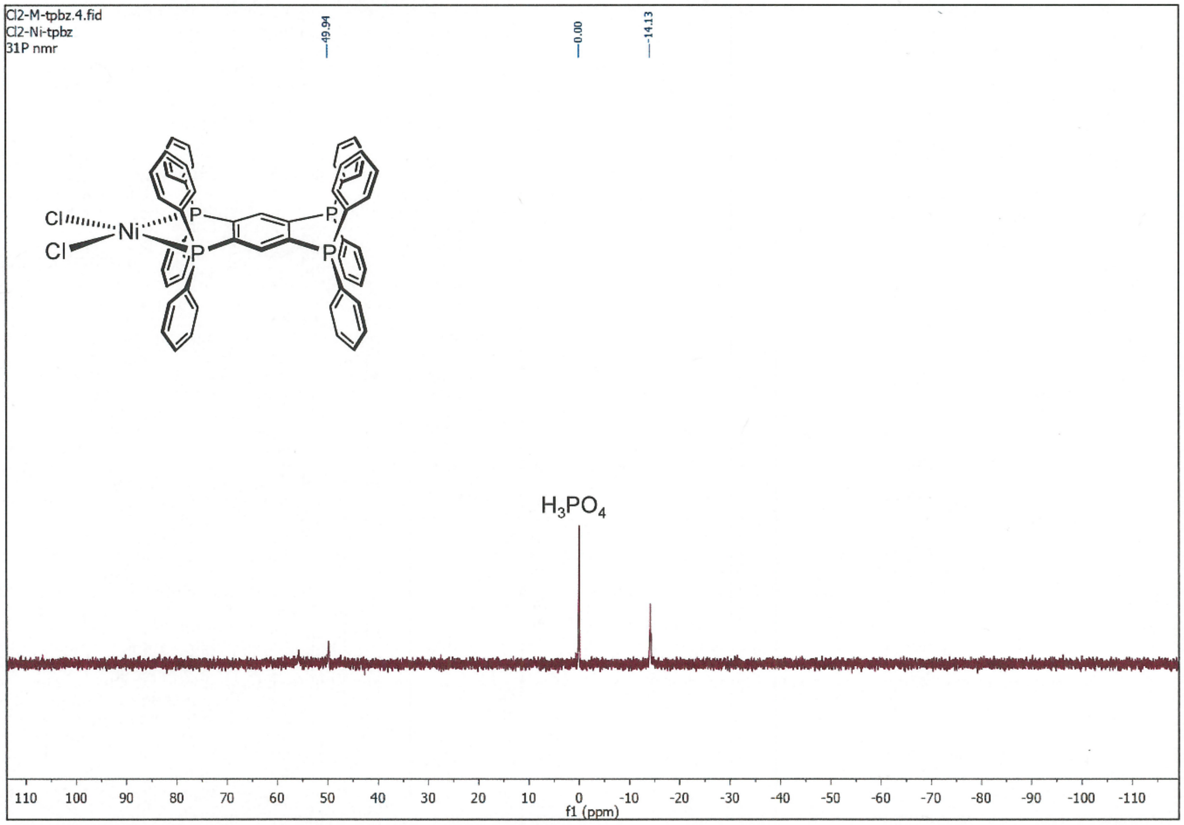

Figure S25. ^{31}P NMR spectrum of [Cl_2Ni(tpbz)] in CD_2Cl_2.

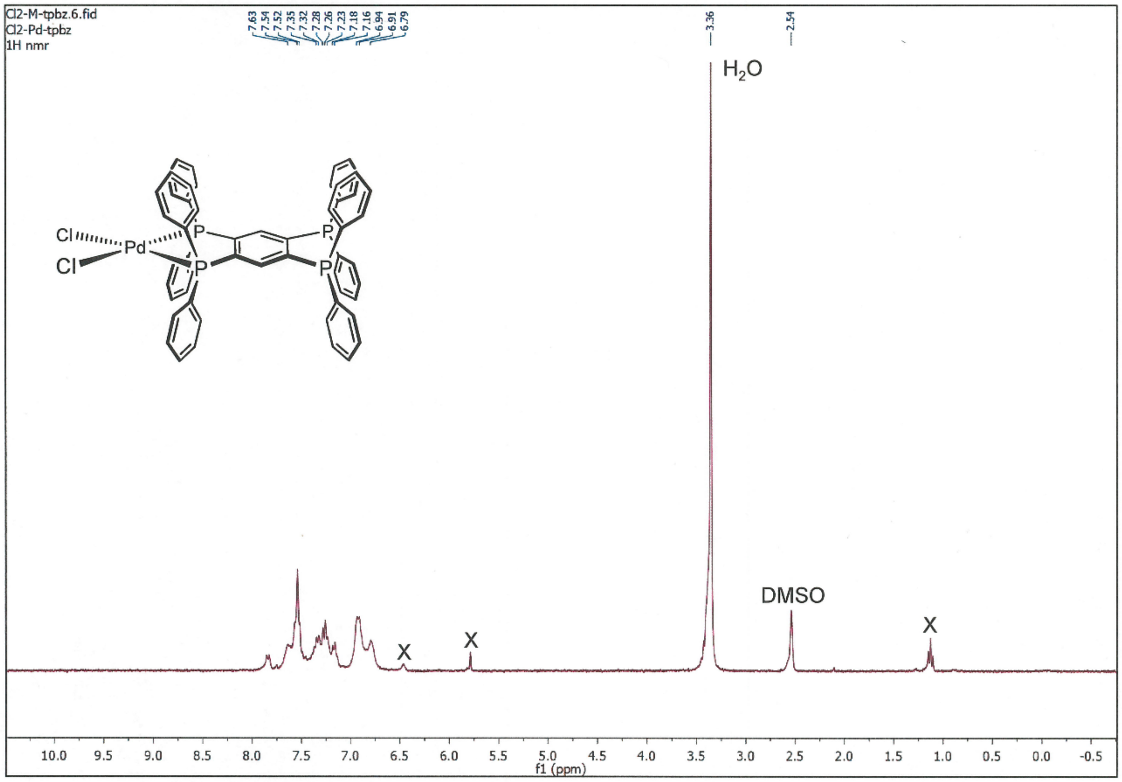

Figure S26. ^{1}H NMR spectrum of $[Cl_2Pd(tpbz)]$ in DMSO-d_6. Signals not corresponding to $[Cl_2Pd(tpbz)]$ are marked with "x".

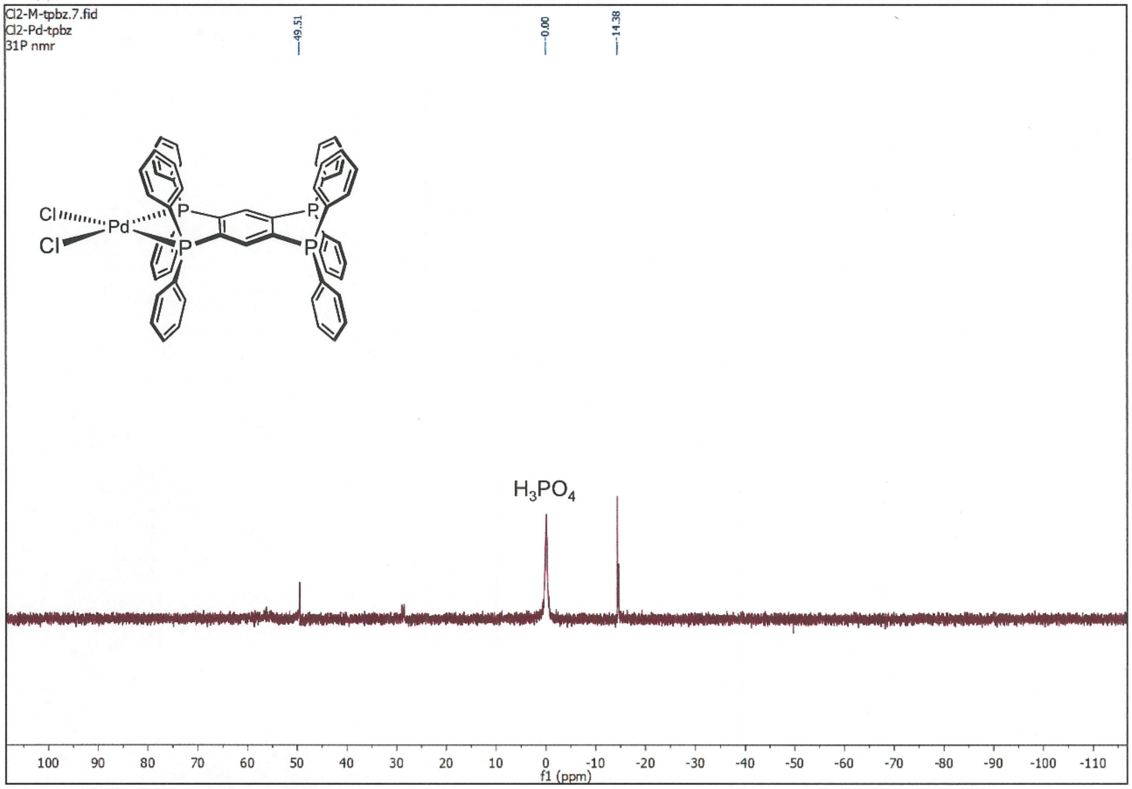

Figure S27. ^{31}P NMR spectrum of $[Cl_2Pd(tpbz)]$ in DMSO-d_6.

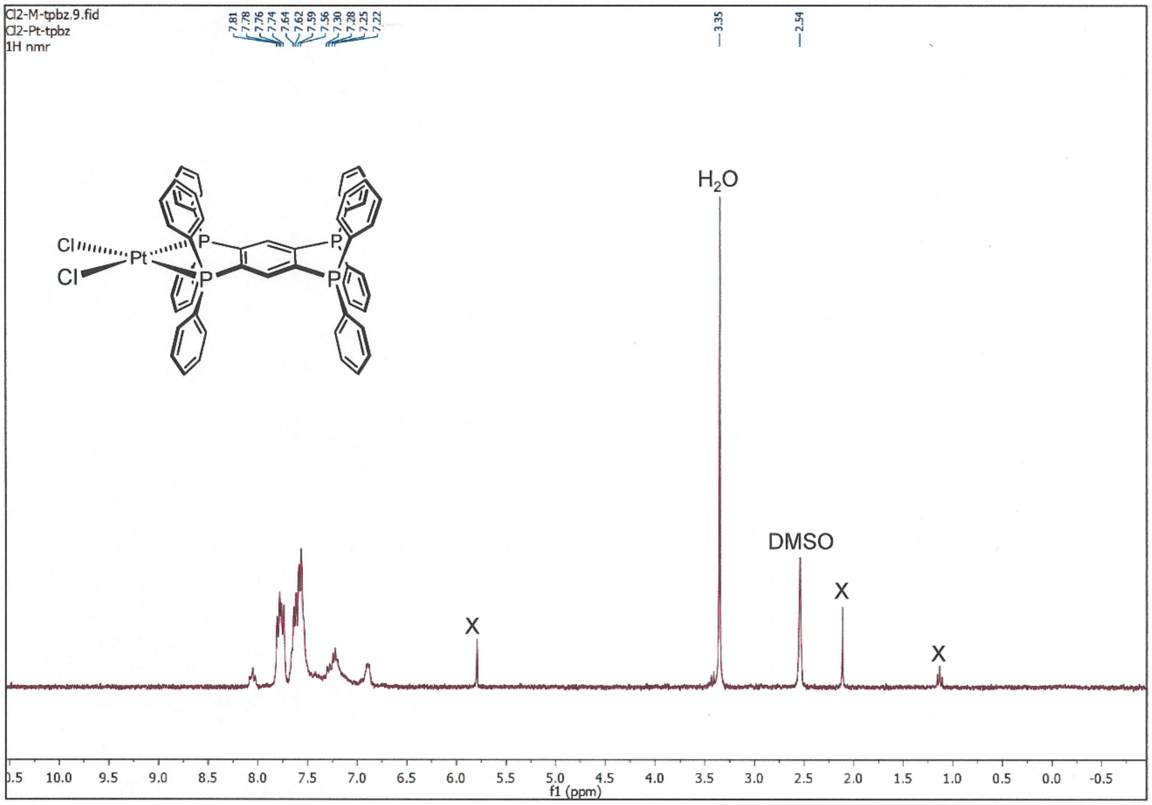

Figure S28. ^{1}H NMR spectrum of [Cl_2Pt(tpbz)] in DMSO-d_6. Signals not corresponding to [Cl_2Pt(tpbz)] are marked with "x".

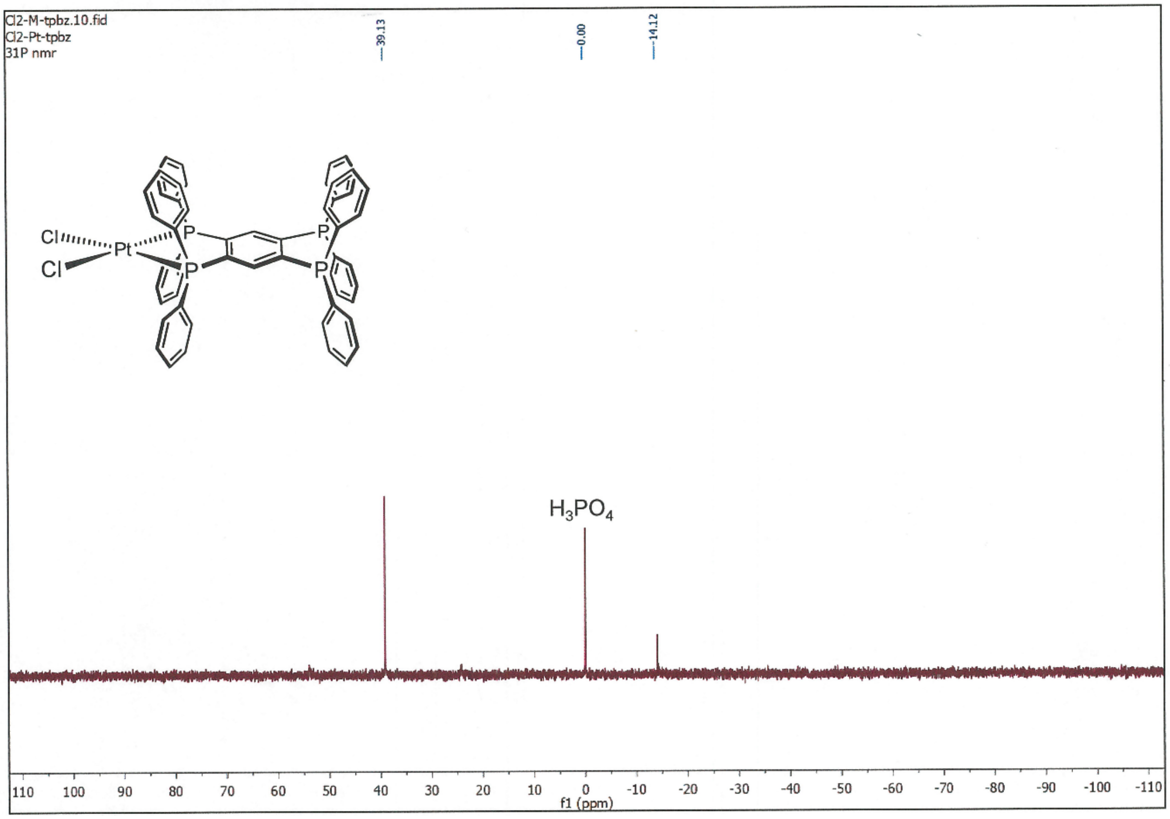

Figure S29. ^{31}P NMR spectrum of [Cl_2Pt(tpbz)] in DMSO-d_6.

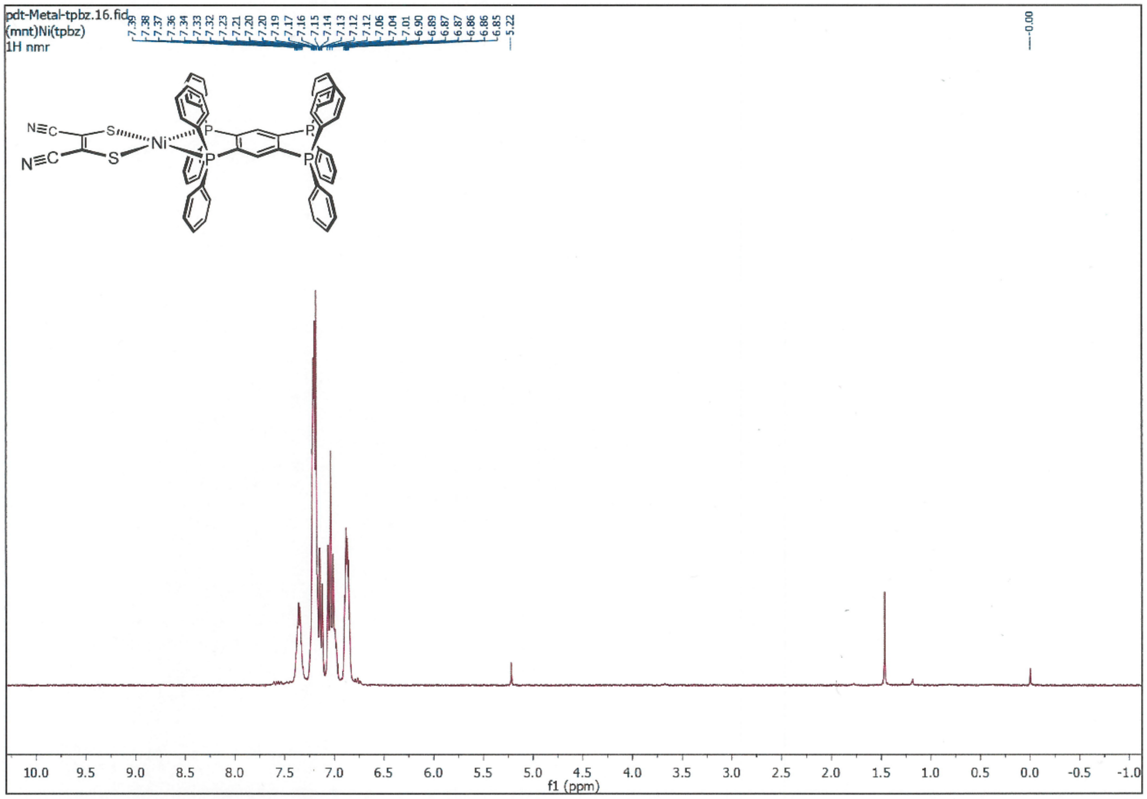

Figure S30. ^{1}H NMR spectrum ($CDCl_3$) of [(mnt)Ni(tpbz)].

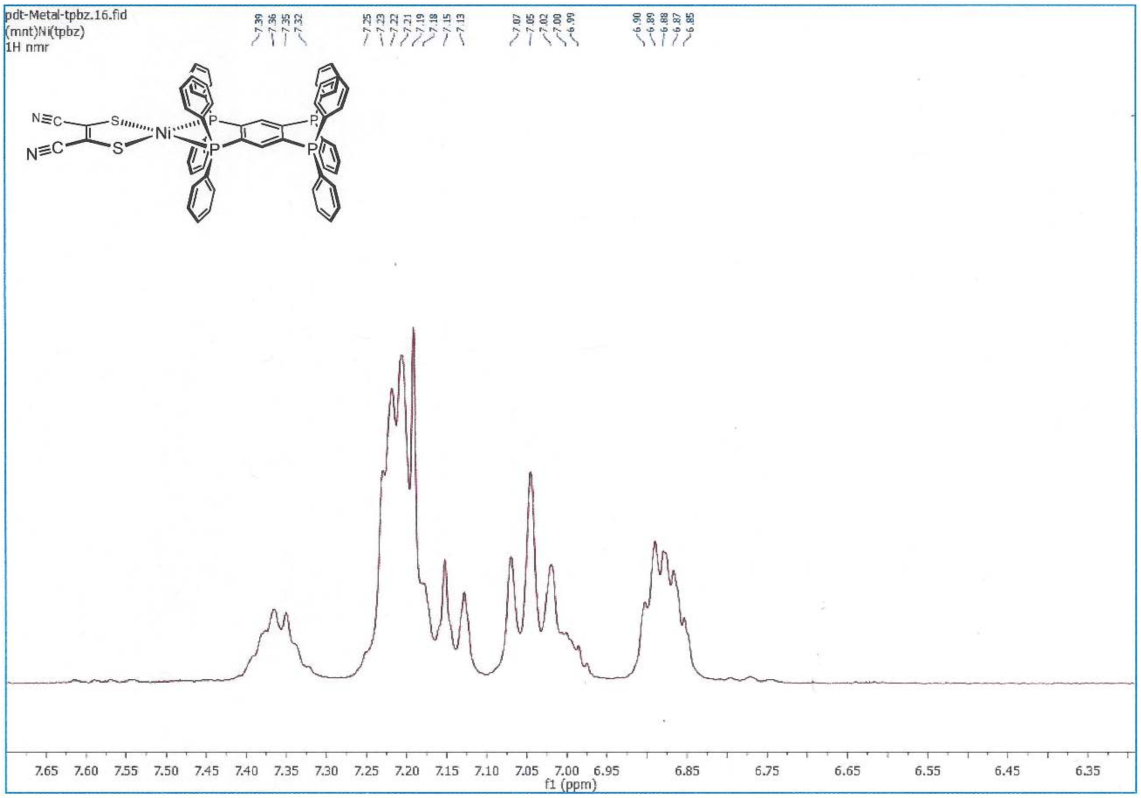

Figure S31. Close-up of the aromatic region of the ^{1}H NMR spectrum ($CDCl_3$) of [(mnt)Ni(tpbz)].

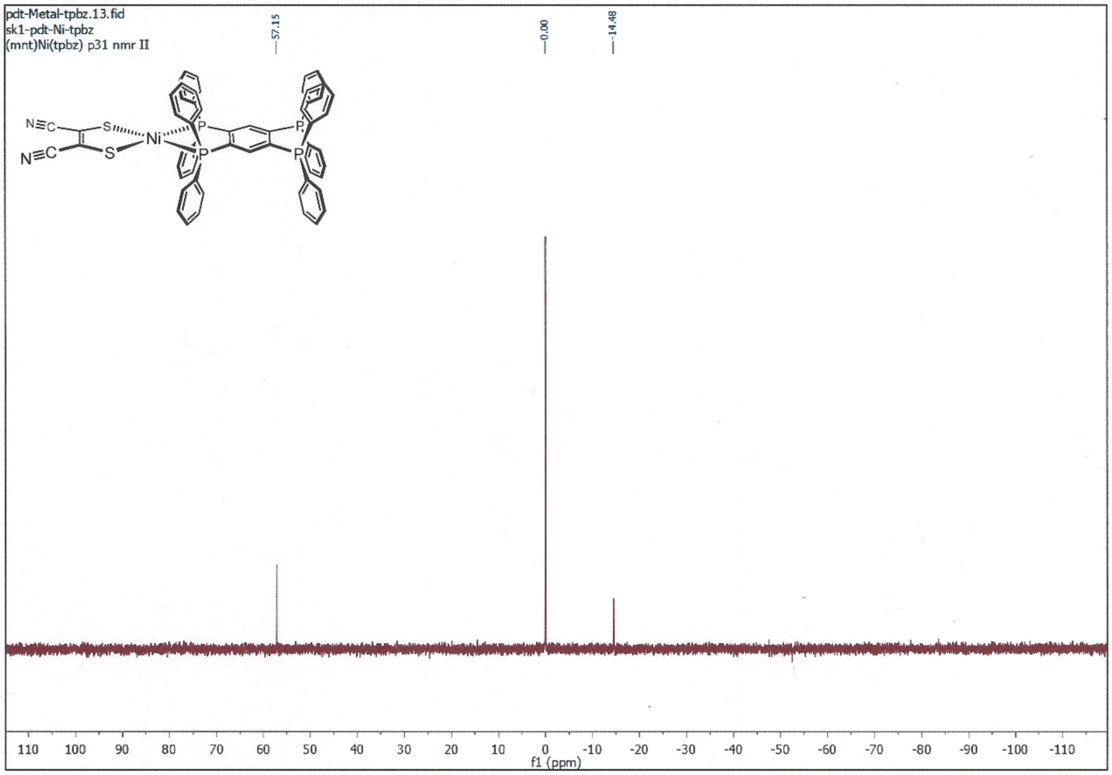

Figure S32. ^{31}P NMR spectrum ($CDCl_3$) of [(mnt)Ni(tpbz)].

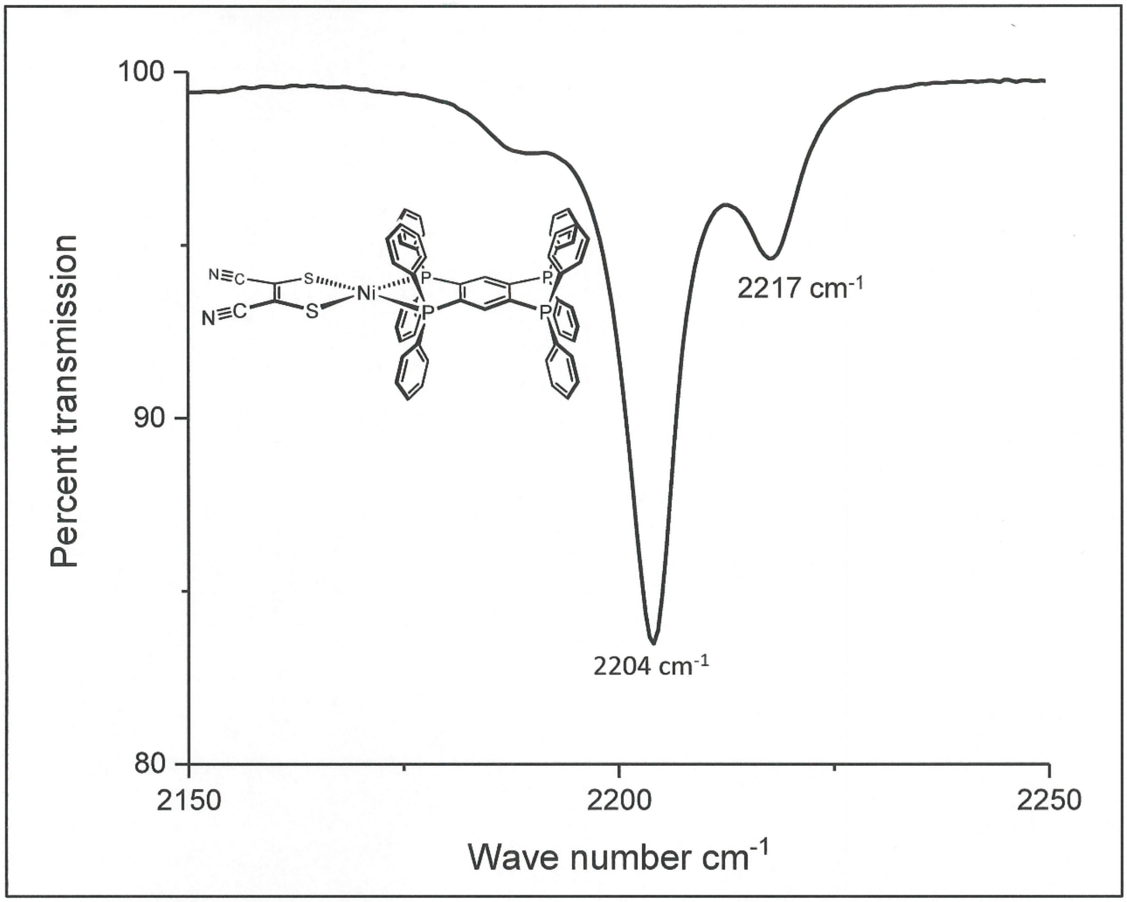

Figure S33. IR spectrum (KBr disk) of [(mnt)Ni(tpbz)].

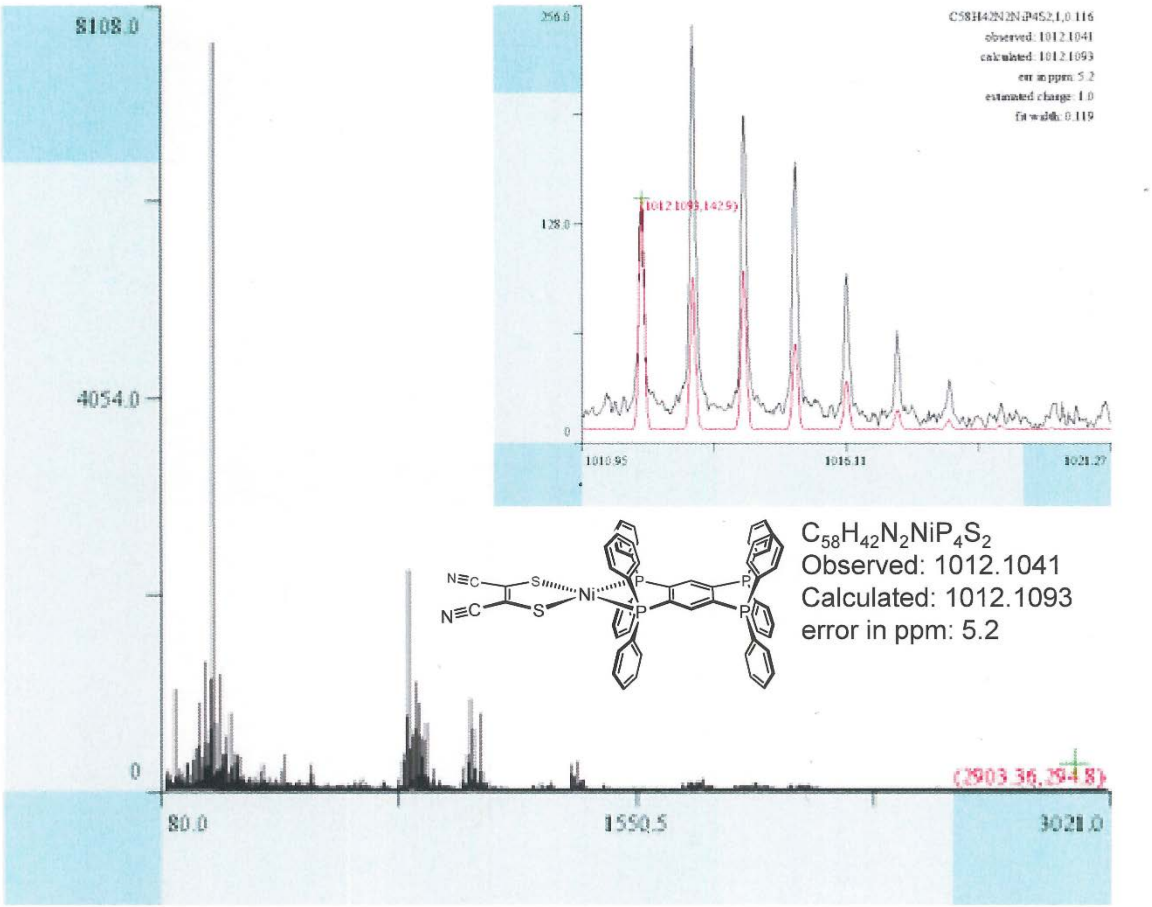

Figure S34. MALDI mass spectrum (positive ion mode) of [(mnt)Ni(tpbz)].

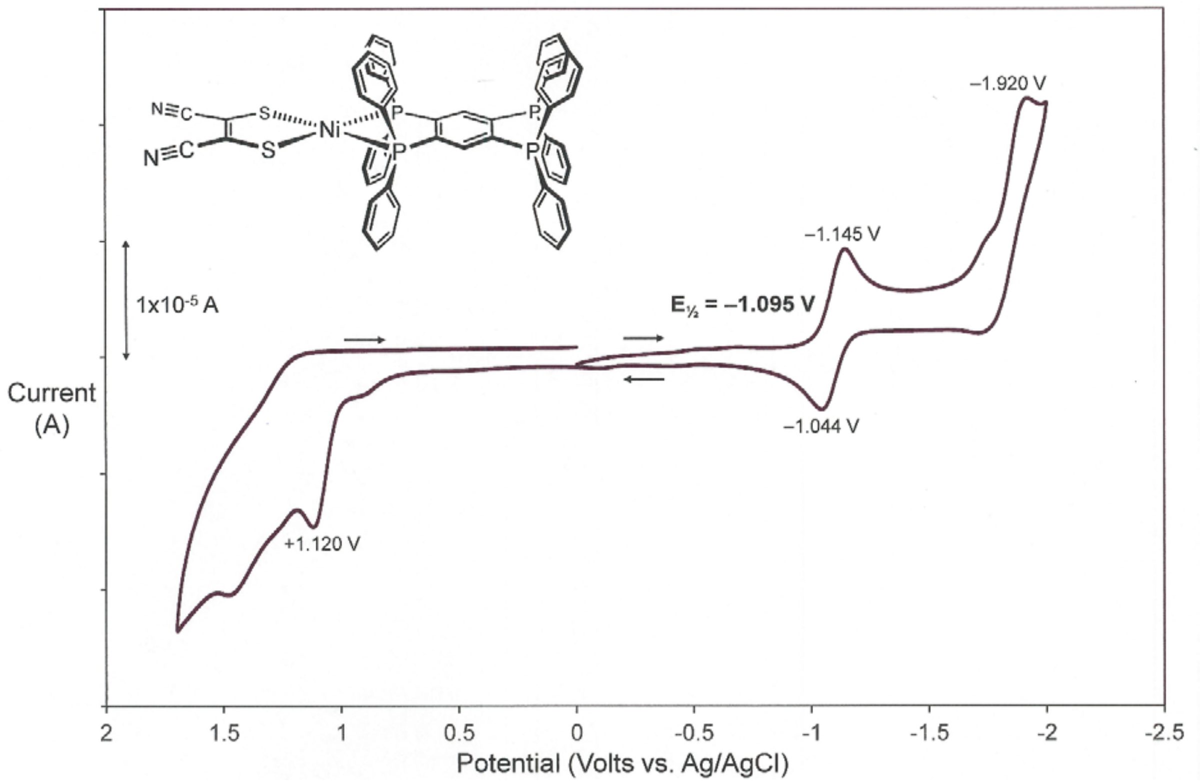

Figure S35. Cyclic voltammogram of [(mnt)Ni(tpbz)] in CH_2Cl_2. The working electrode was Pt disk, the scan speed 100 mV/sec, and the supporting electrolyte [^{n}Bu$_4$][PF_6].

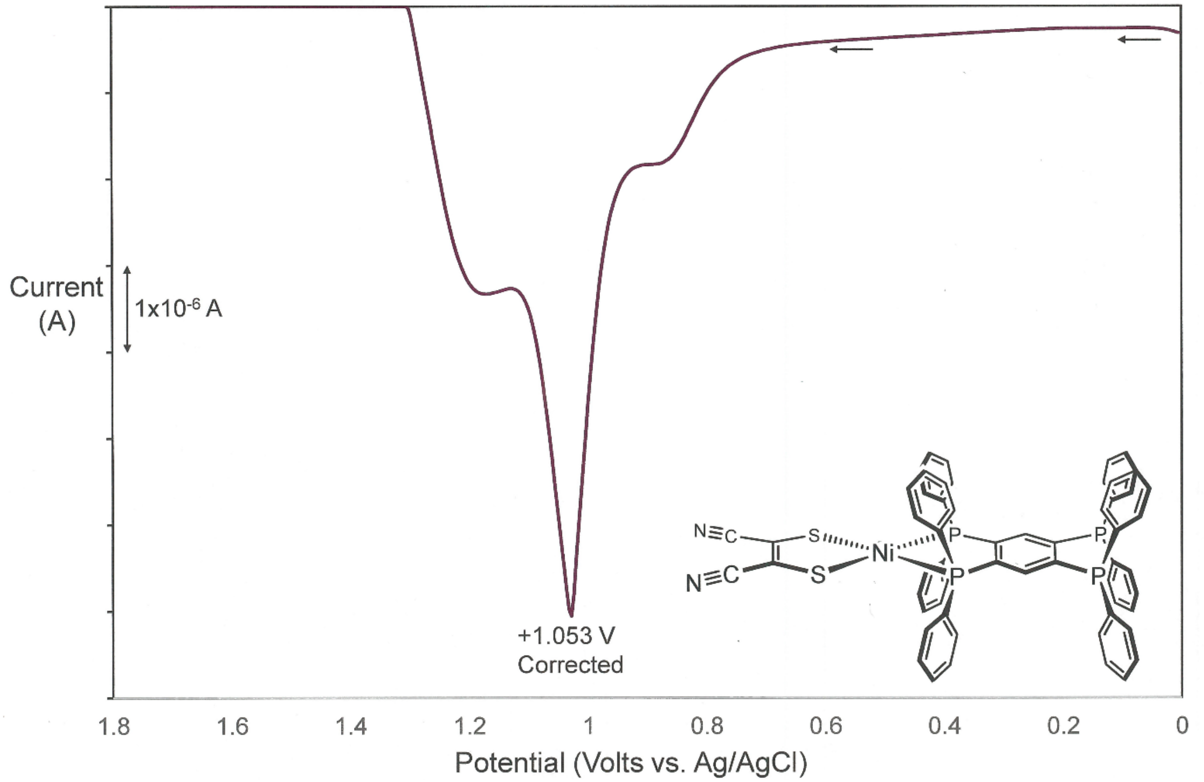

Figure S36. Differential pulse voltammogram of [(mnt)Ni(tpbz)] in CH_2Cl_2. The working electrode was Pt disk, the scan speed 100 mV/sec, and the supporting electrolyte [^{n}Bu$_4$][PF_6].

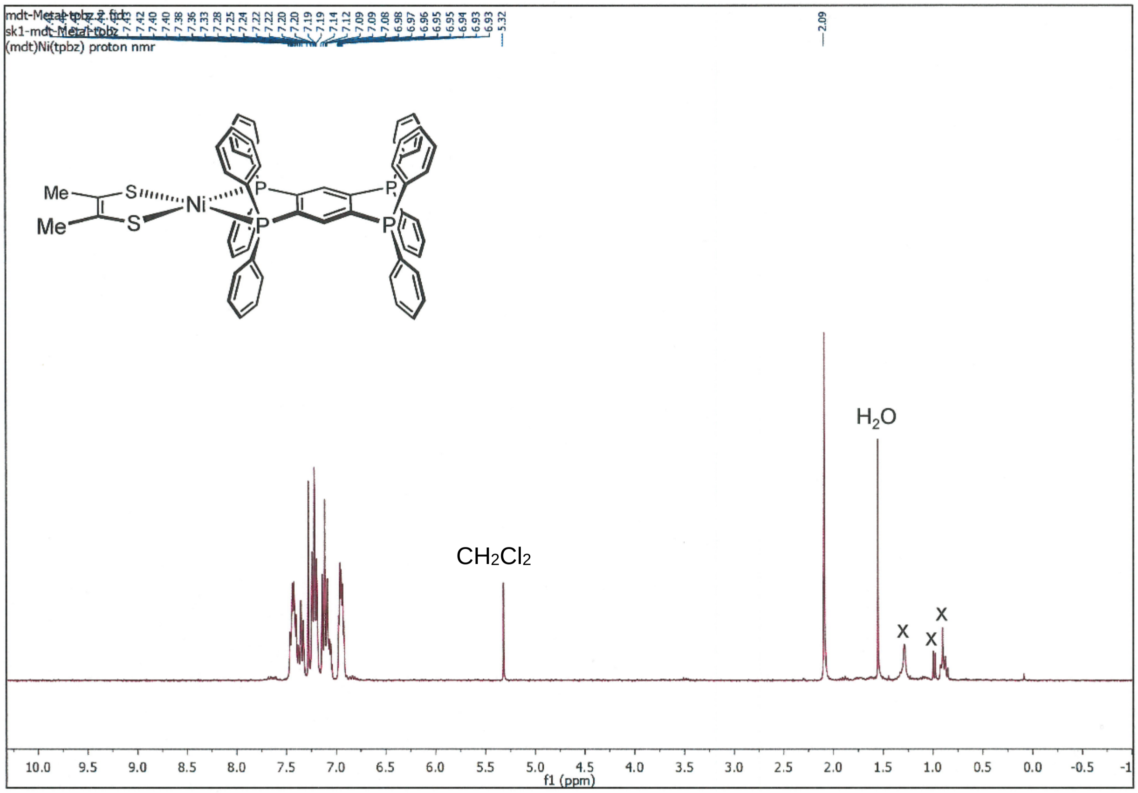

Figure S37. ^{1}H NMR spectrum ($CDCl_3$) of [(mdt)Ni(tpbz)]. Signals not belonging to [(mdt)Ni(tpbz)] are designated with "x".

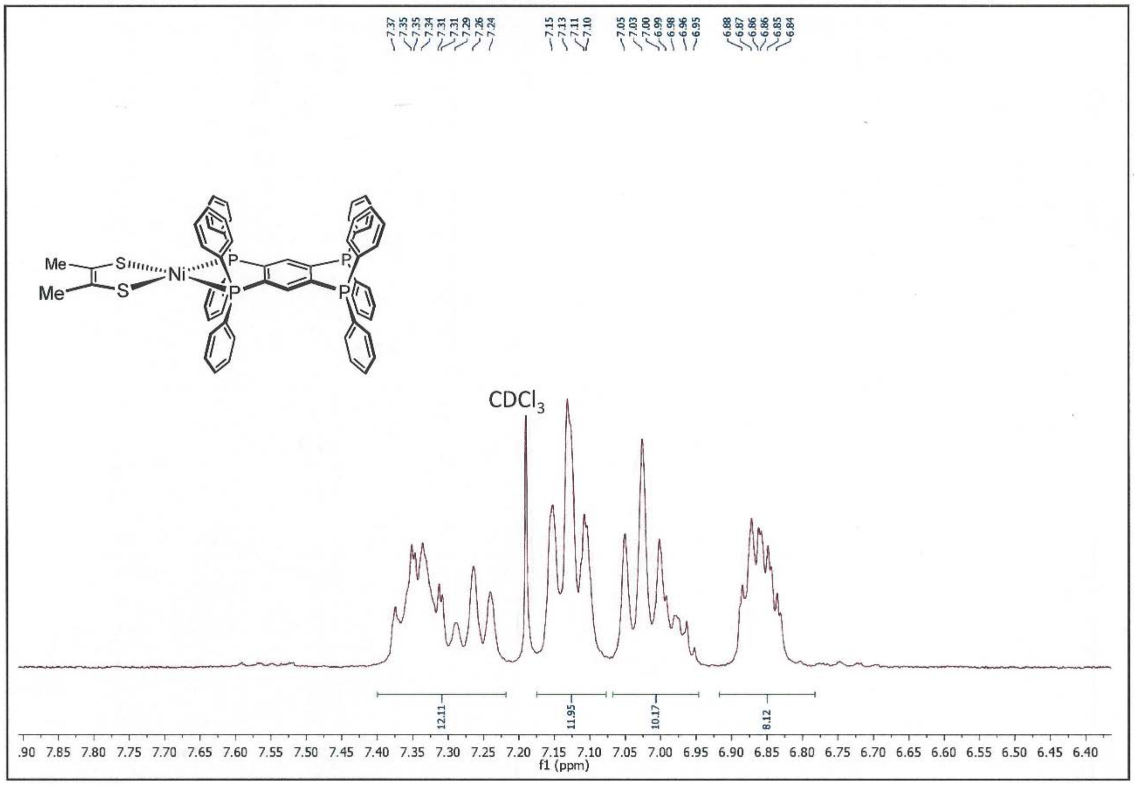

Figure S38. Close-up ^{1}H NMR spectrum ($CDCl_3$) of [(mdt)Ni(tpbz)].

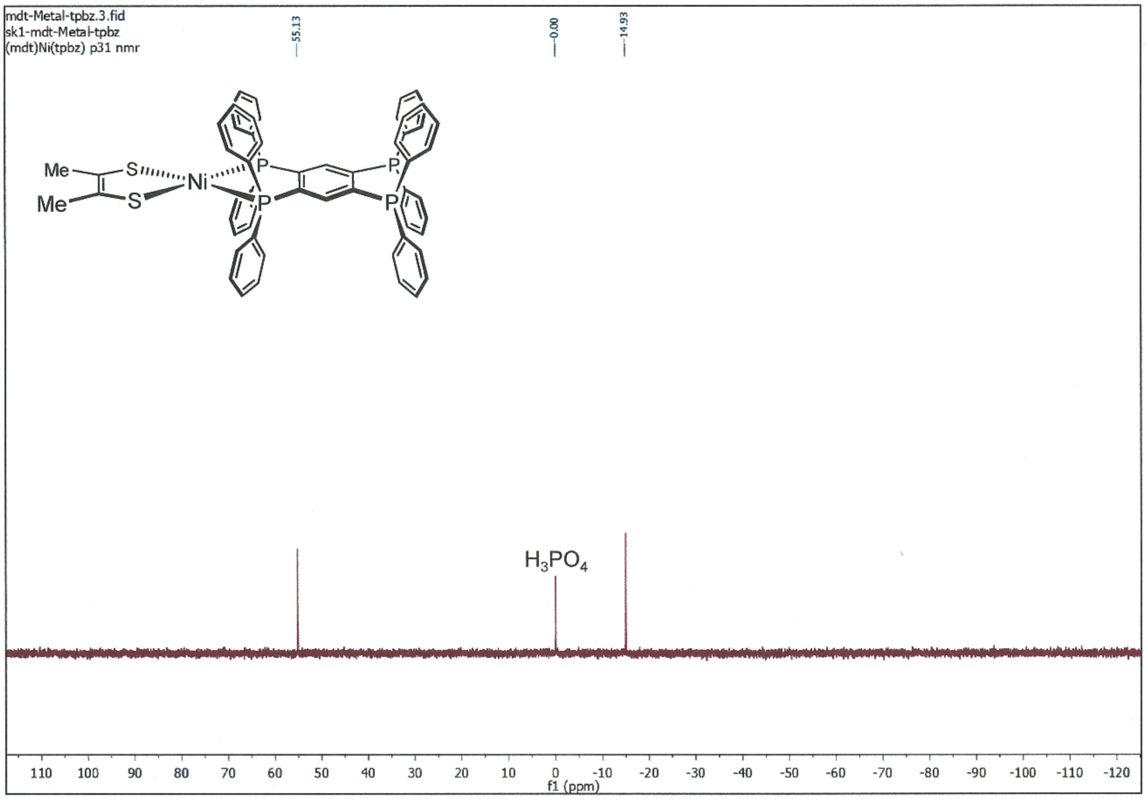

Figure S39. ^{31}P NMR spectrum ($CDCl_3$) of [(mdt)Ni(tpbz)].

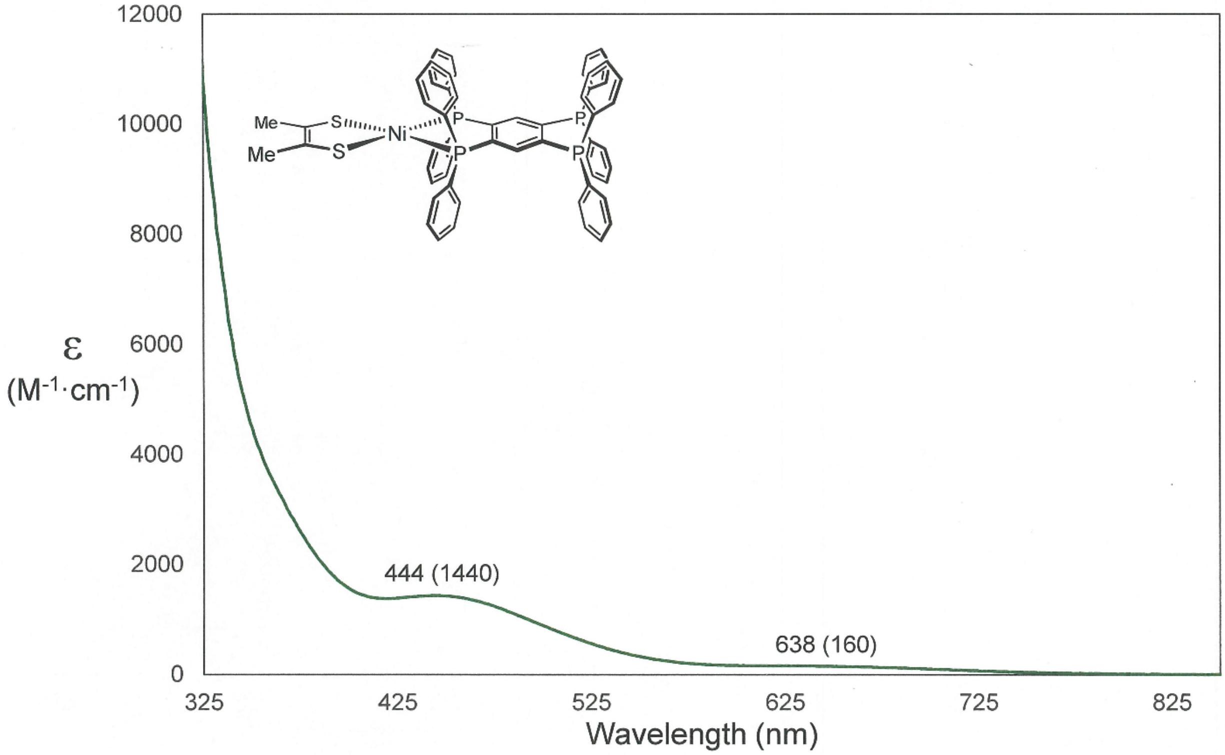

Figure S40. UV-vis absorption spectrum (CH_2Cl_2) of [(mdt)Ni(tpbz)].

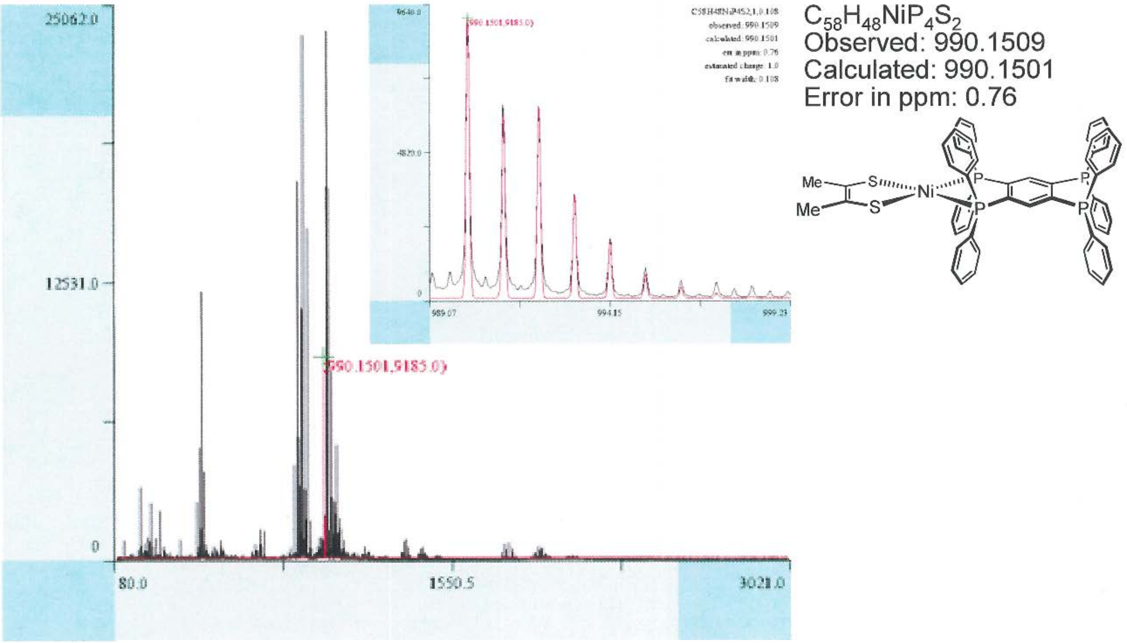

Figure S41. ESI mass spectrum (positive ion mode) of [(mdt)Ni(tpbz)].

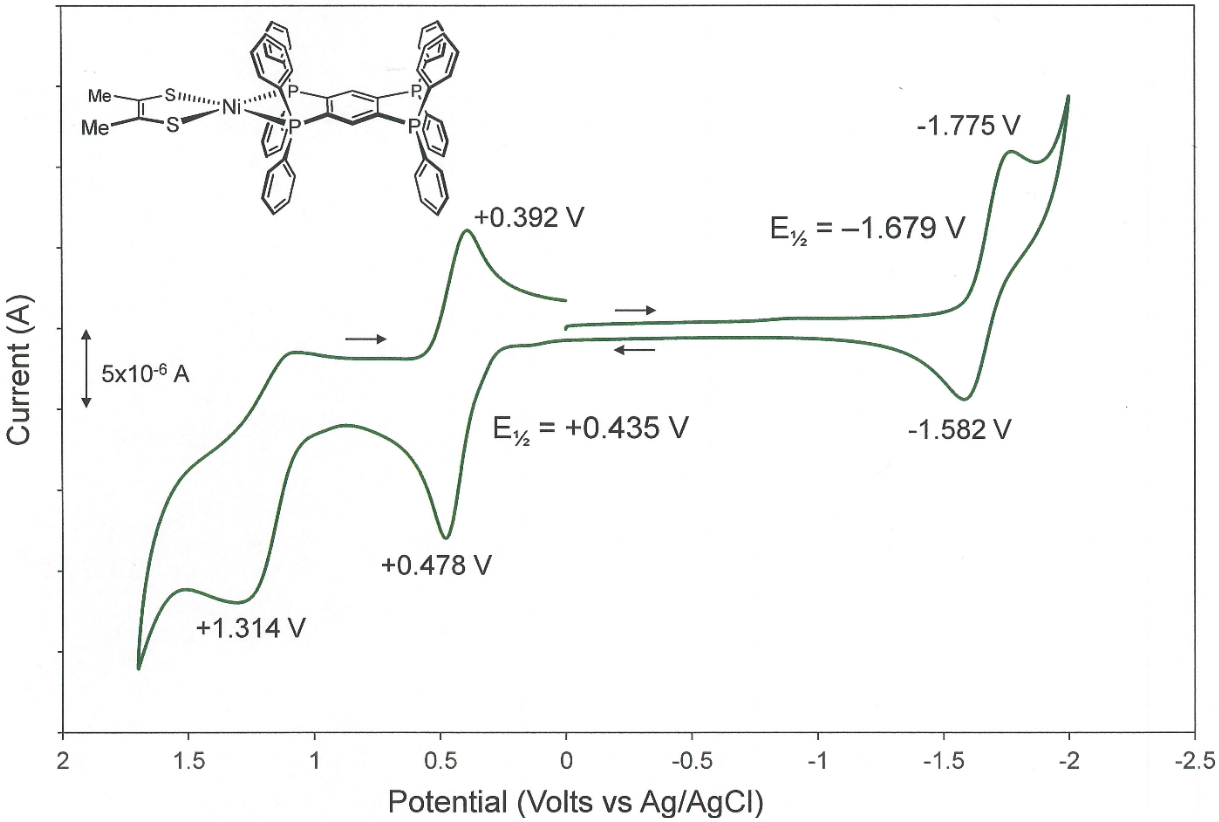

Figure S42. Cyclic voltammogram of [(mdt)Ni(tpbz)] in CH_2Cl_2. The working electrode was glassy carbon, the scan speed 100 mV/sec, and the supporting electrolyte [$^{n}Bu_4$][PF_6].

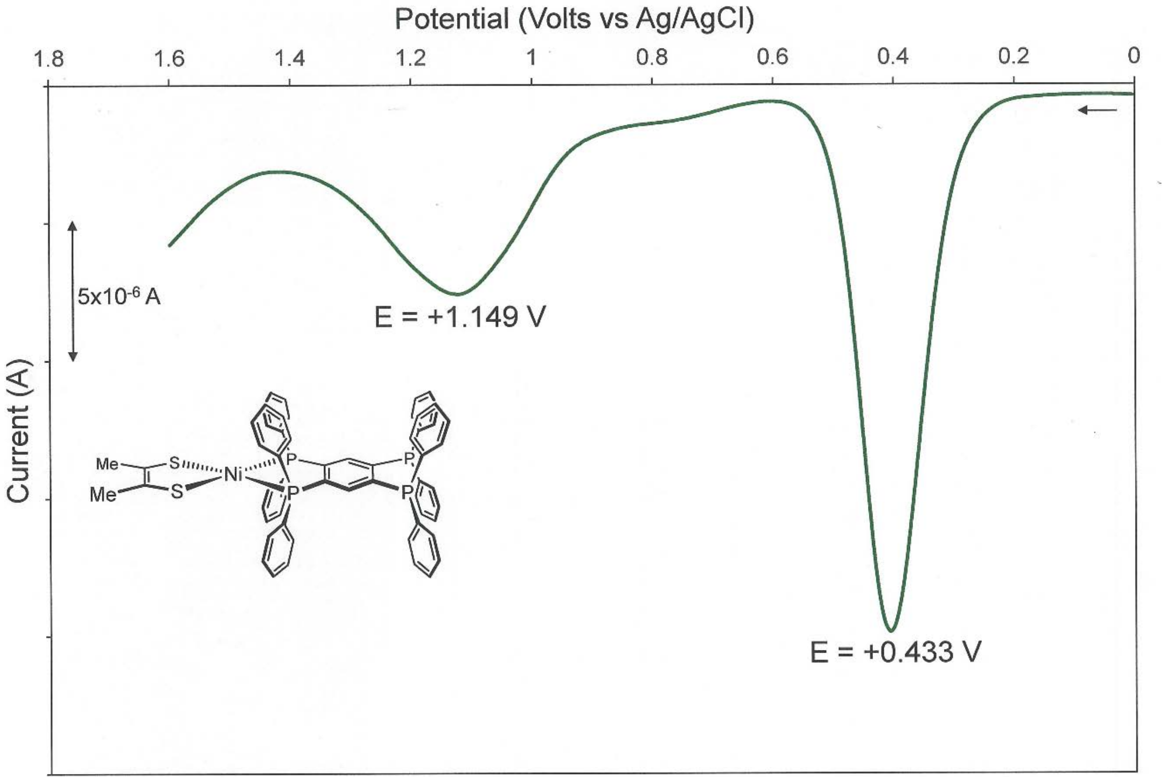

Figure S43. Differential pulse voltammogram (oxidizing direction) of [(mdt)Ni(tpbz)] in CH_2Cl_2. The working electrode was glassy carbon, and the supporting electrolyte was [^{n}Bu$_4$][PF_6].

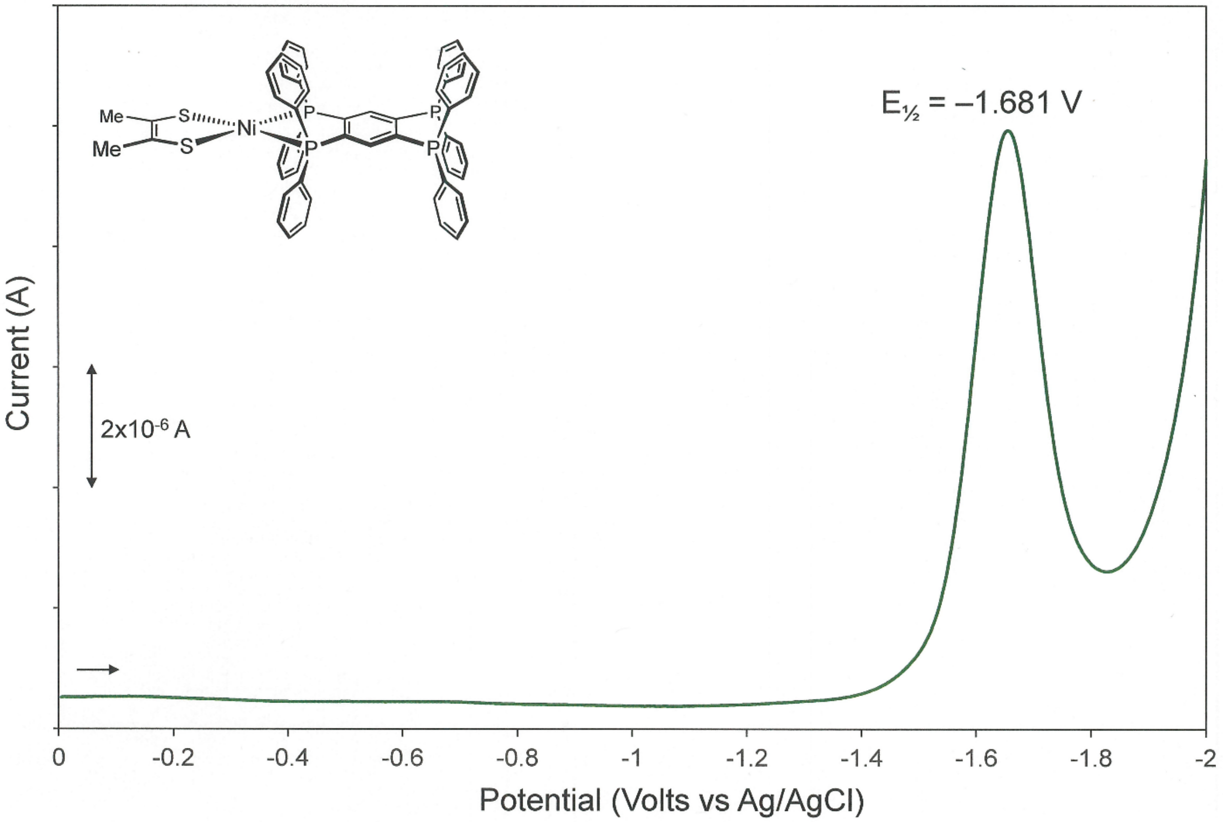

Figure S44. Differential pulse voltammogram (reducing direction) of [(mdt)Ni(tpbz)] in CH_2Cl_2. The working electrode was glassy carbon, and the supporting electrolyte was [^{n}Bu$_4$][PF_6].

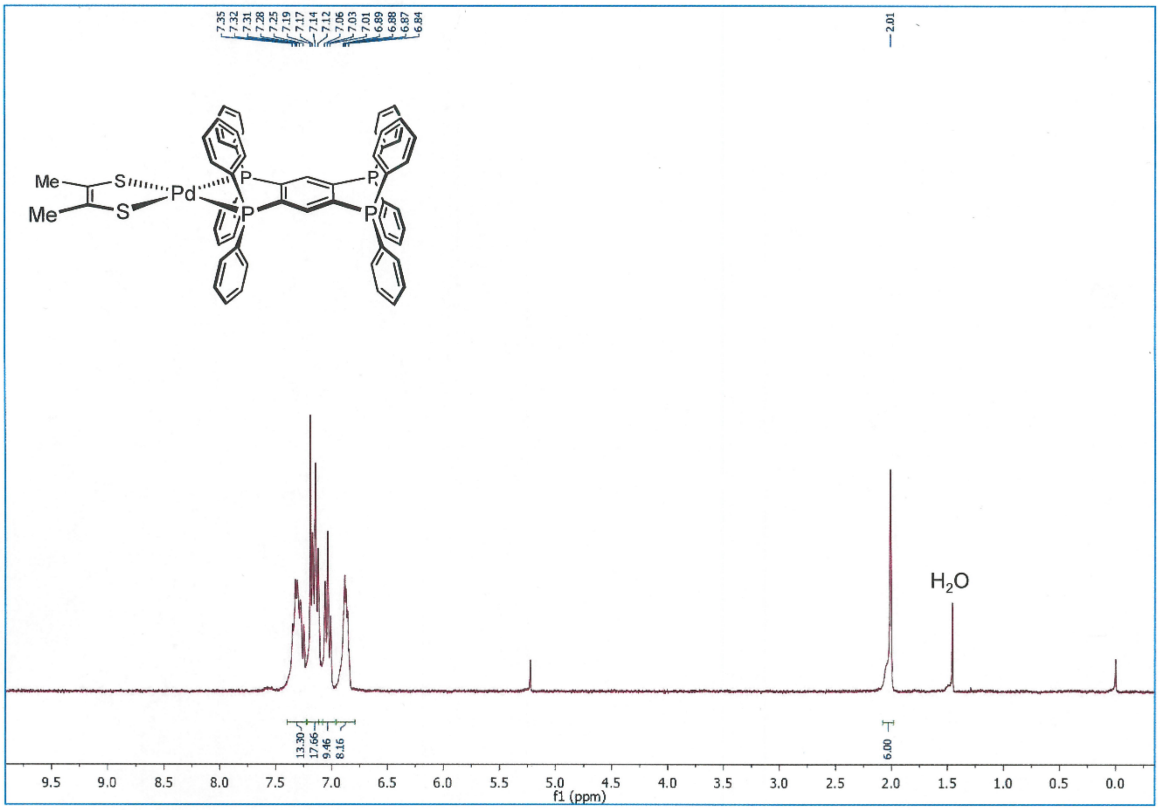

Figure S45. ^{1}H NMR spectrum ($CDCl_3$) of [(mdt)Pd(tpbz)].

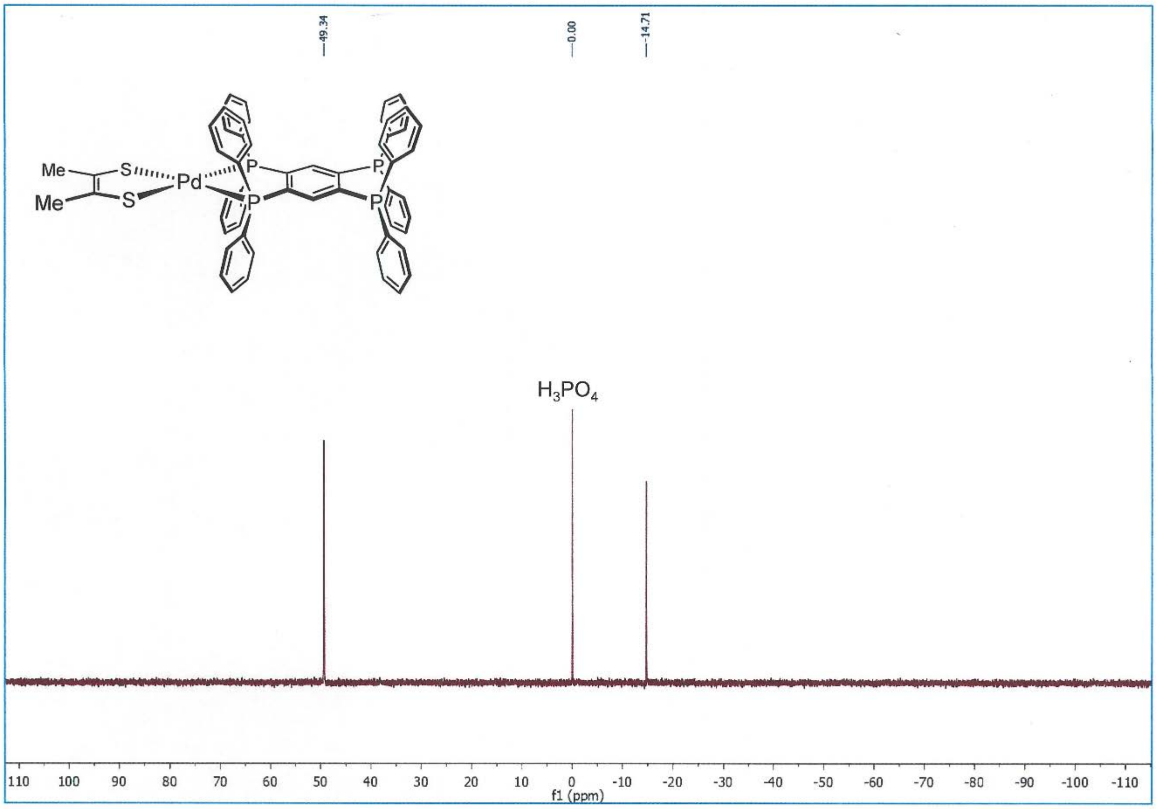

Figure S46. ^{31}P NMR spectrum ($CDCl_3$) of [(mdt)Pd(tpbz)].

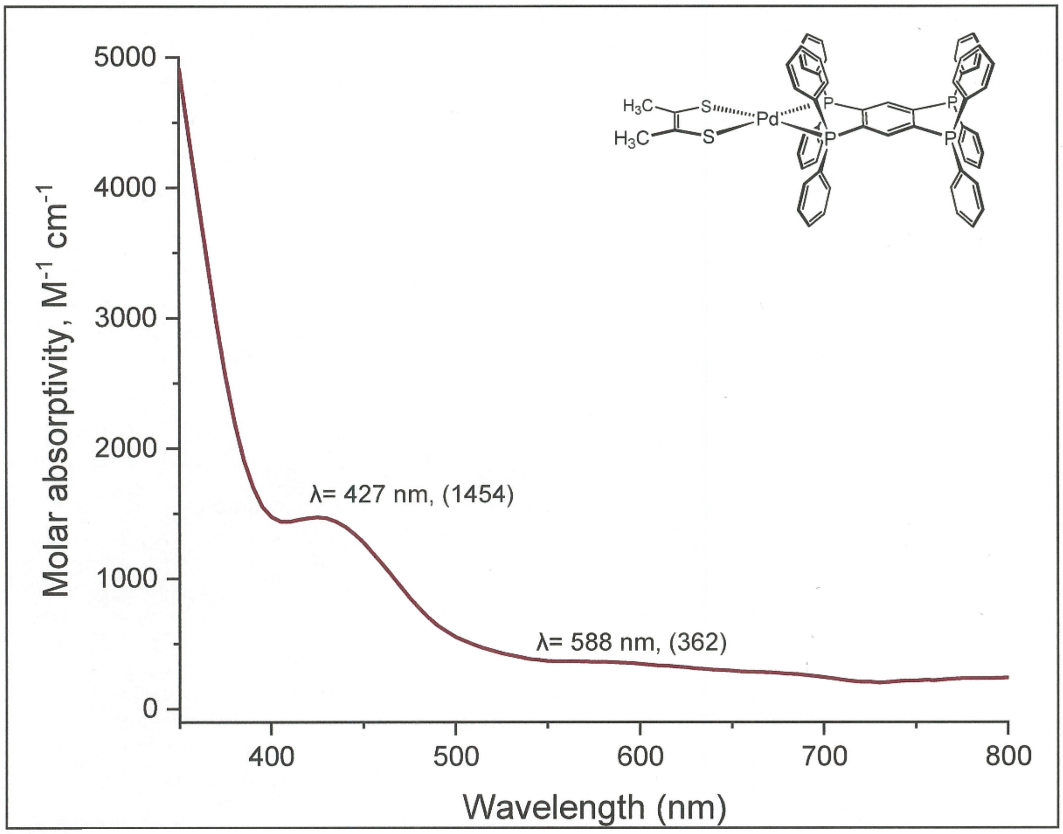

Figure S47. UV-vis spectrum (CH_2Cl_2) of [($Me_2C_2S_2$)Pd(tpbz)].

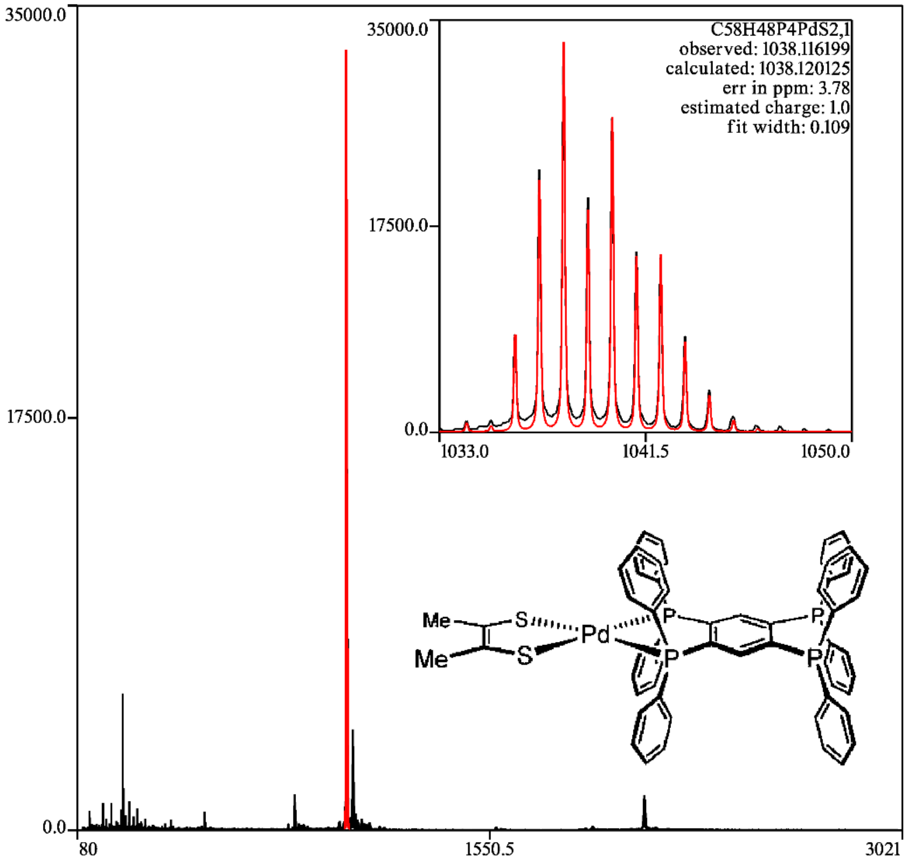

Figure S48. ESI mass spectrum (positive ion mode) of [($Me_2C_2S_2$)Pd(tpbz)].

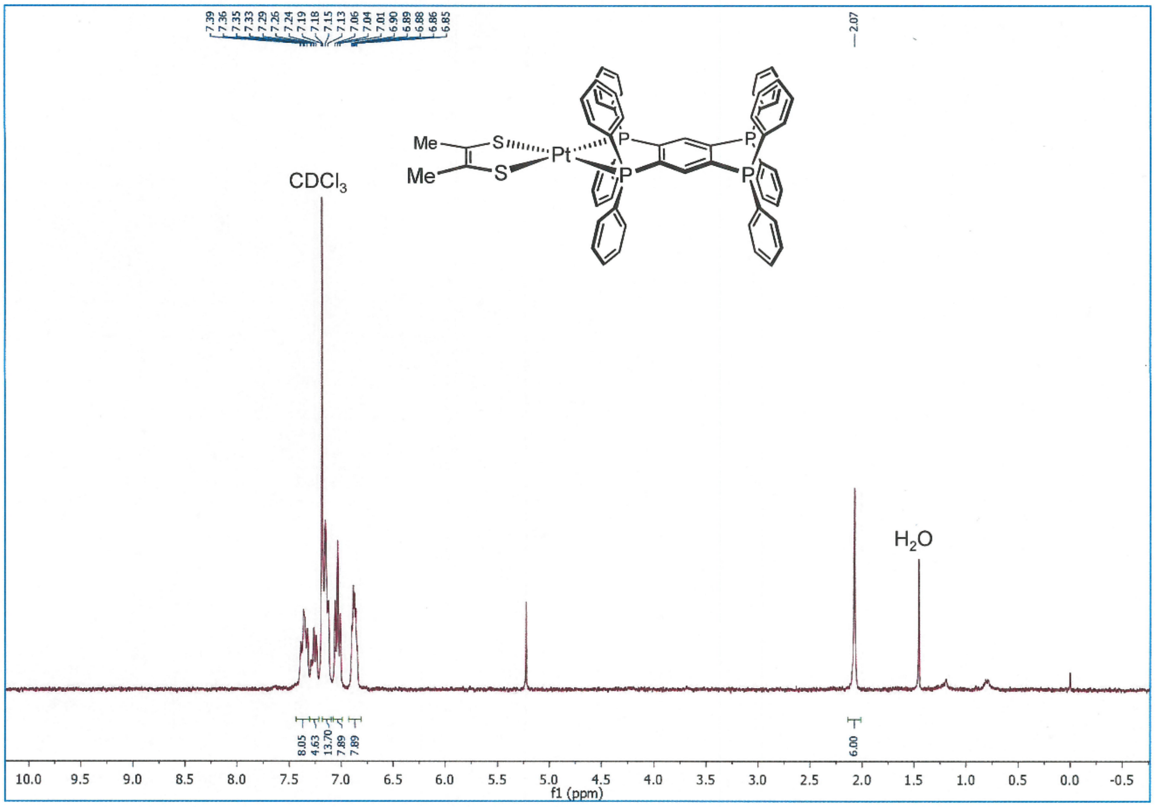

Figure S49. ^{1}H NMR spectrum ($CDCl_3$) of [($Me_2C_2S_2$)Pt(tpbz)].

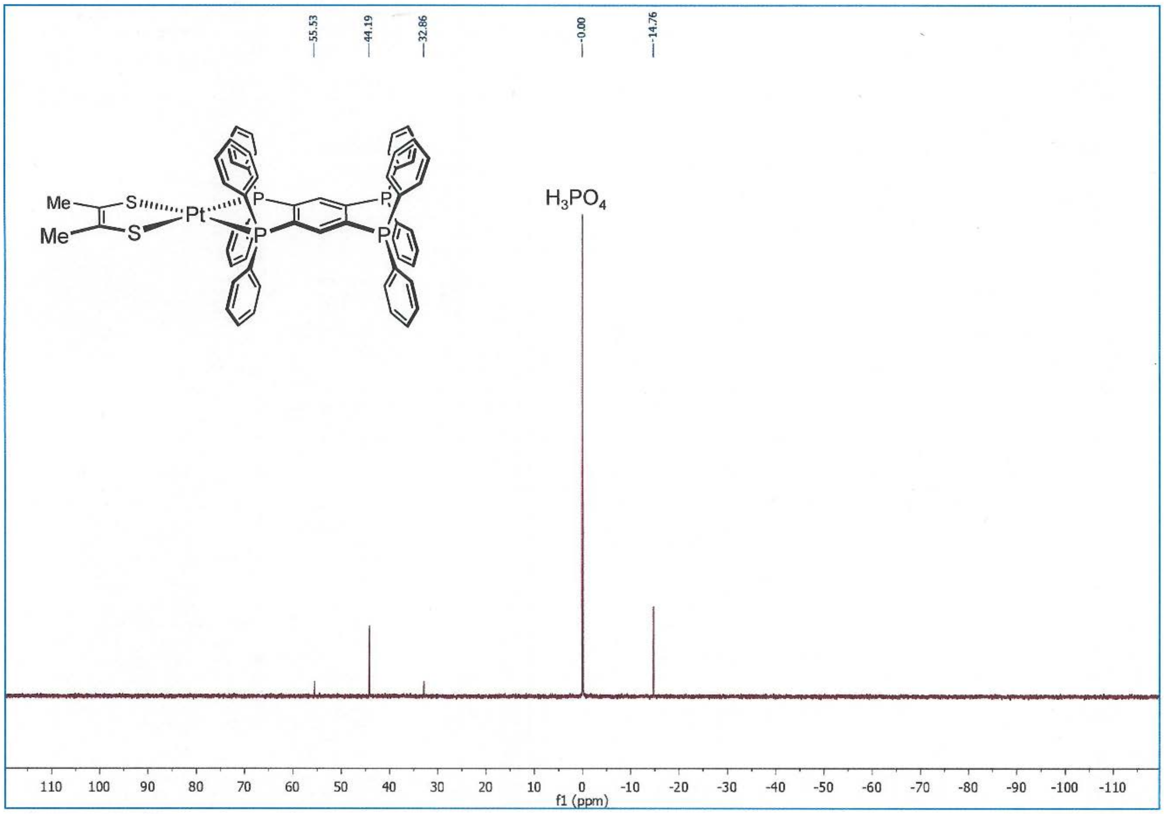

Figure S50. ^{31}P NMR spectrum ($CDCl_3$) of [($Me_2C_2S_2$)Pt(tpbz)].

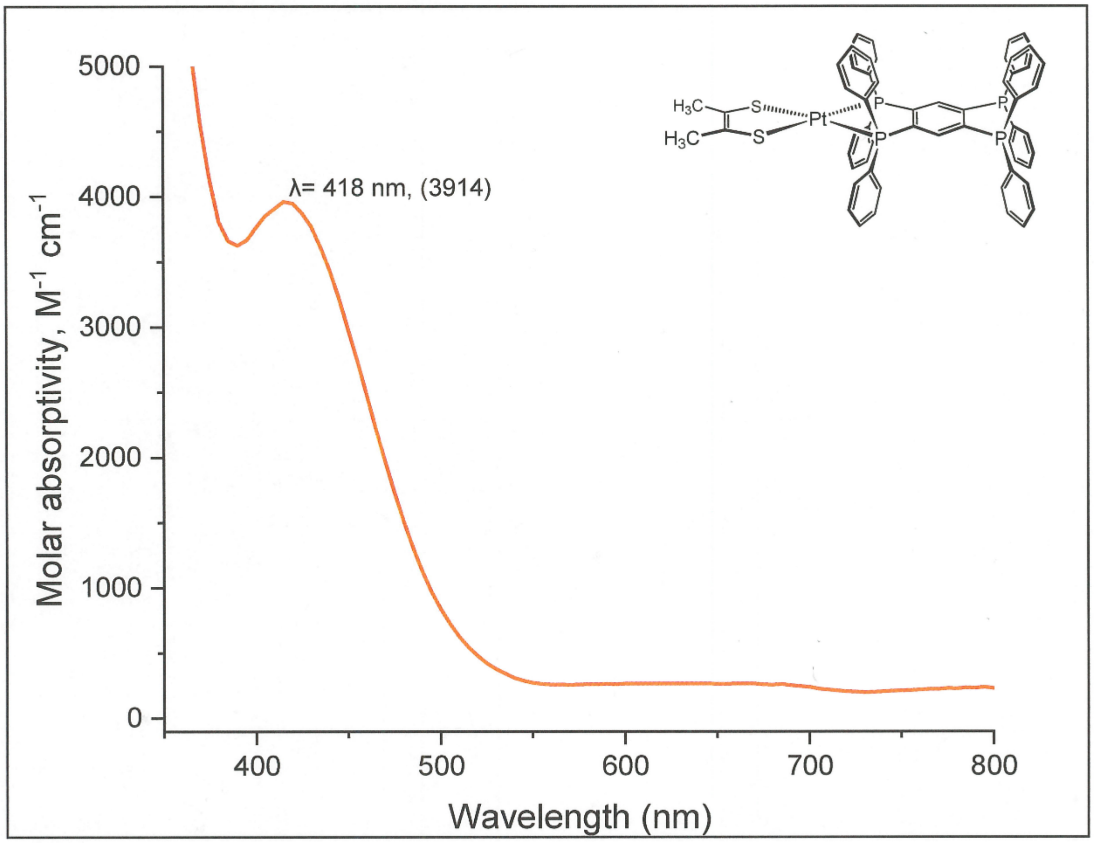

Figure S51. ESI mass spectrum (positive ion mode) of [($Me_2C_2S_2$)Pt(tpbz)].

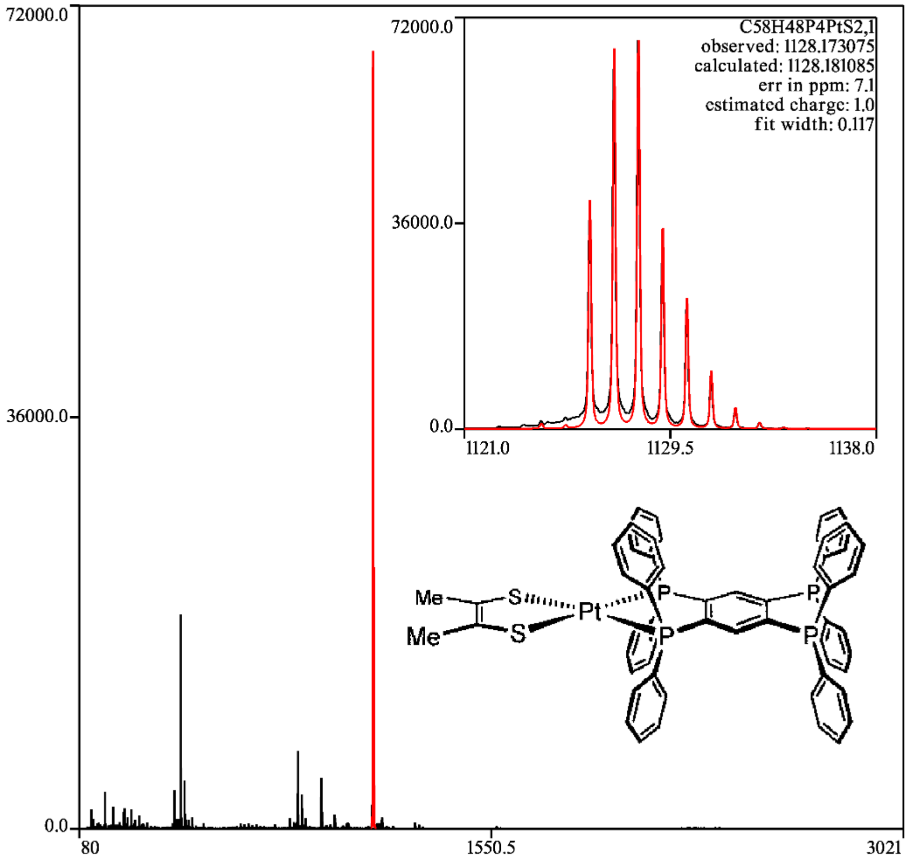

Figure S52. ESI mass spectrum (positive ion mode) of [($Me_2C_2S_2$)Pt(tpbz)].

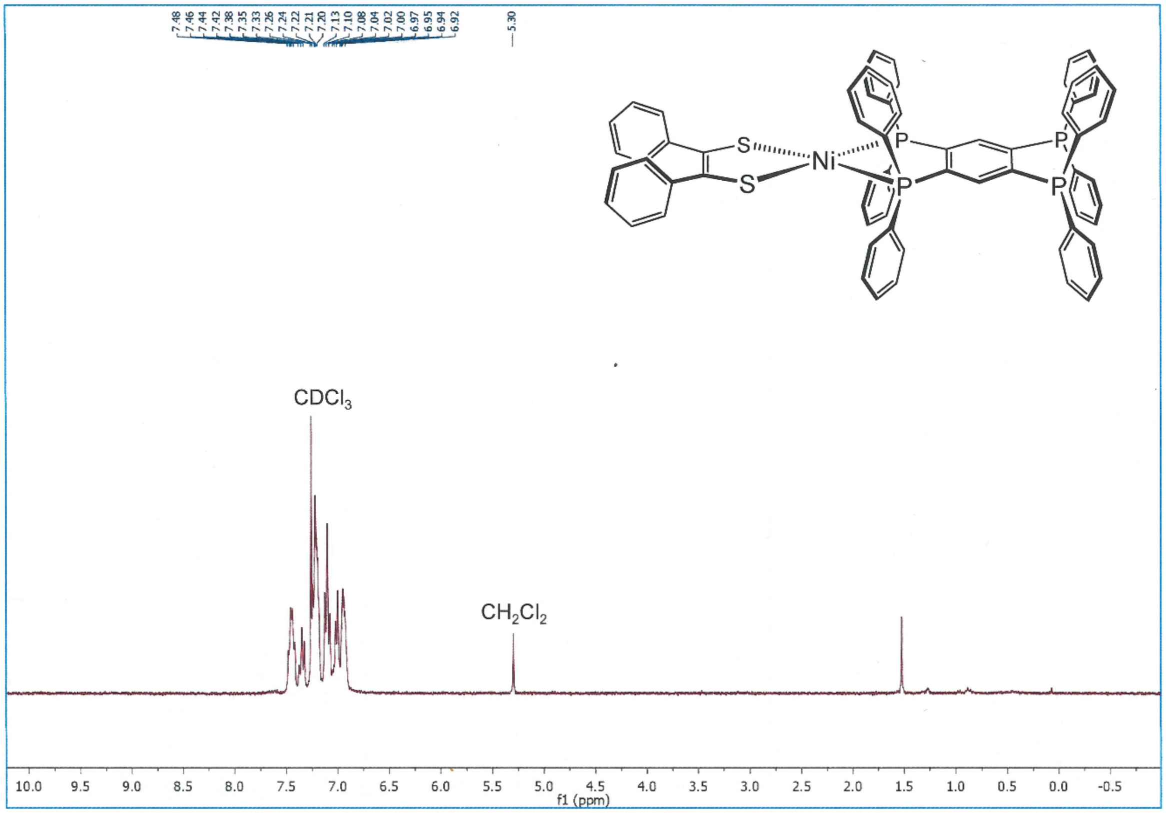

Figure S53. ^{1}H NMR spectrum ($CDCl_3$) of [($Ph_2C_2S_2$)Ni(tpbz)].

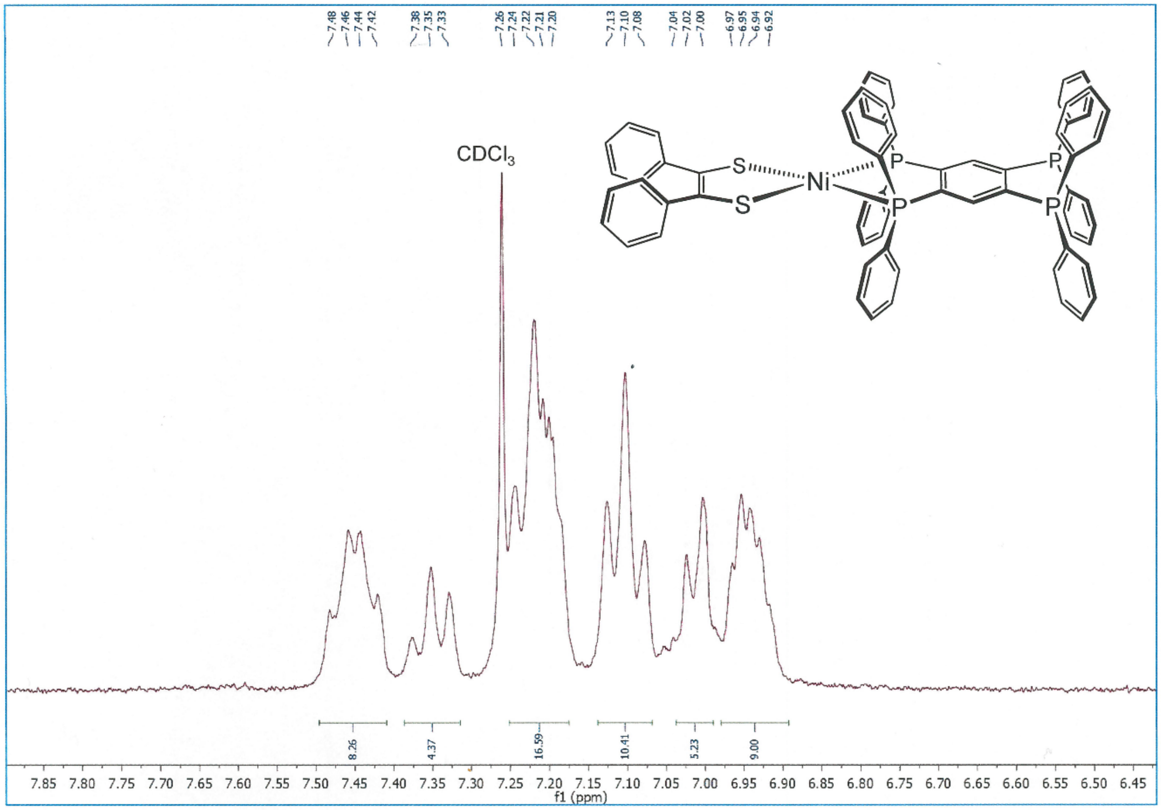

Figure S54. Close-up of the aromatic region of the ^{1}H NMR spectrum ($CDCl_3$) of [($Ph_2C_2S_2$)Ni(tpbz)].

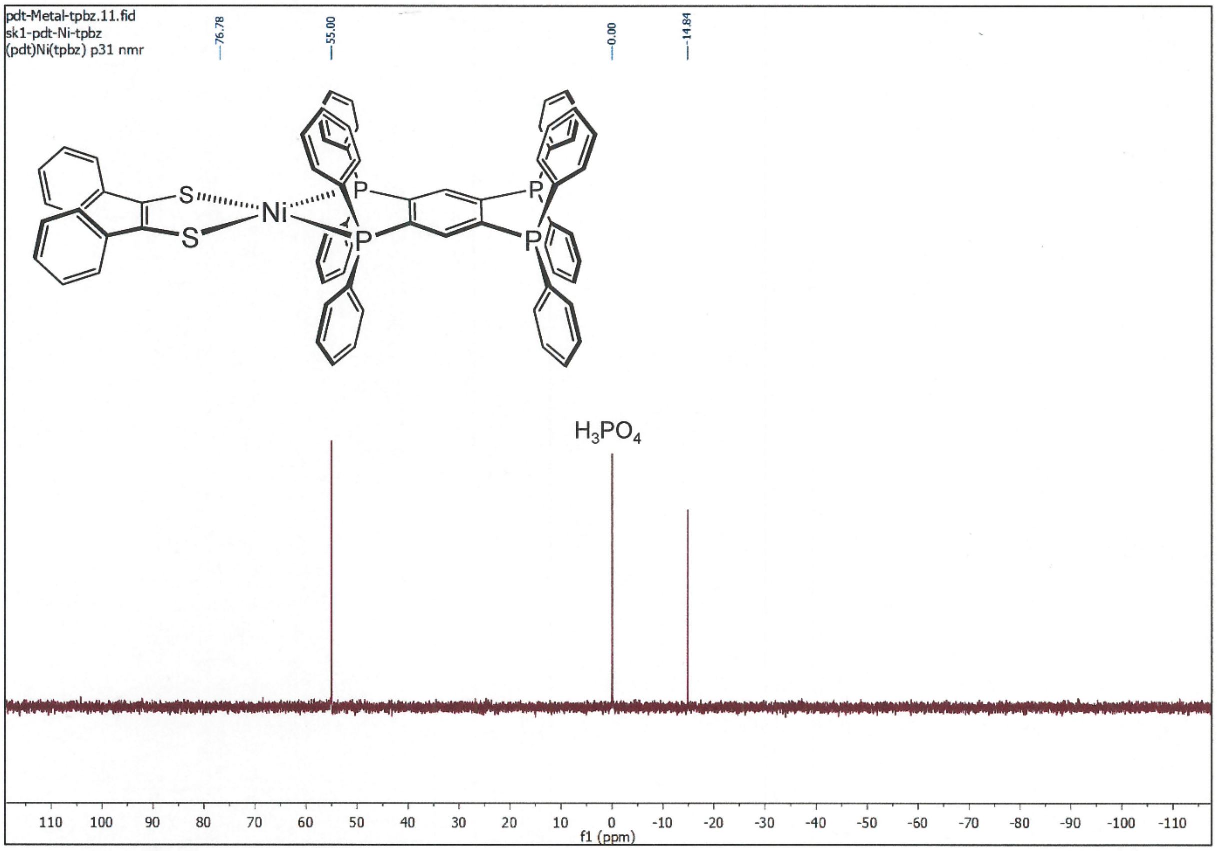

Figure S55. ^{31}P NMR spectrum ($CDCl_3$) of [($Ph_2C_2S_2$)Ni(tpbz)].

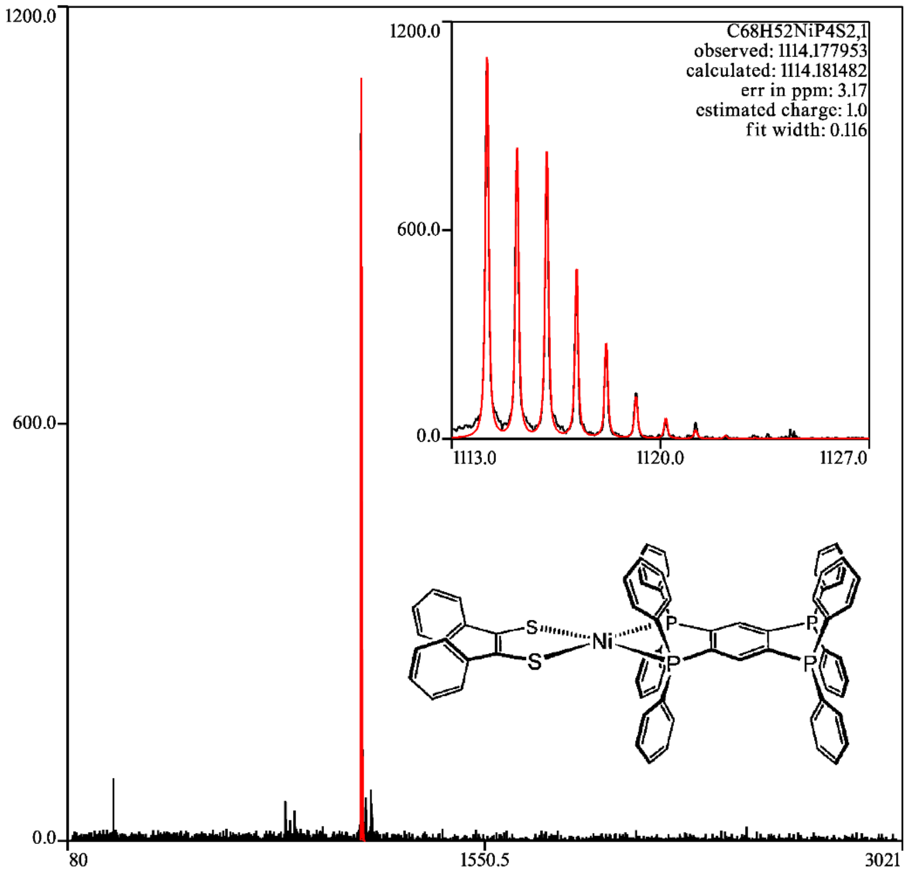

Figure S56. ESI mass spectrum (positive ion mode) of [($Ph_2C_2S_2$)Ni(tpbz)].

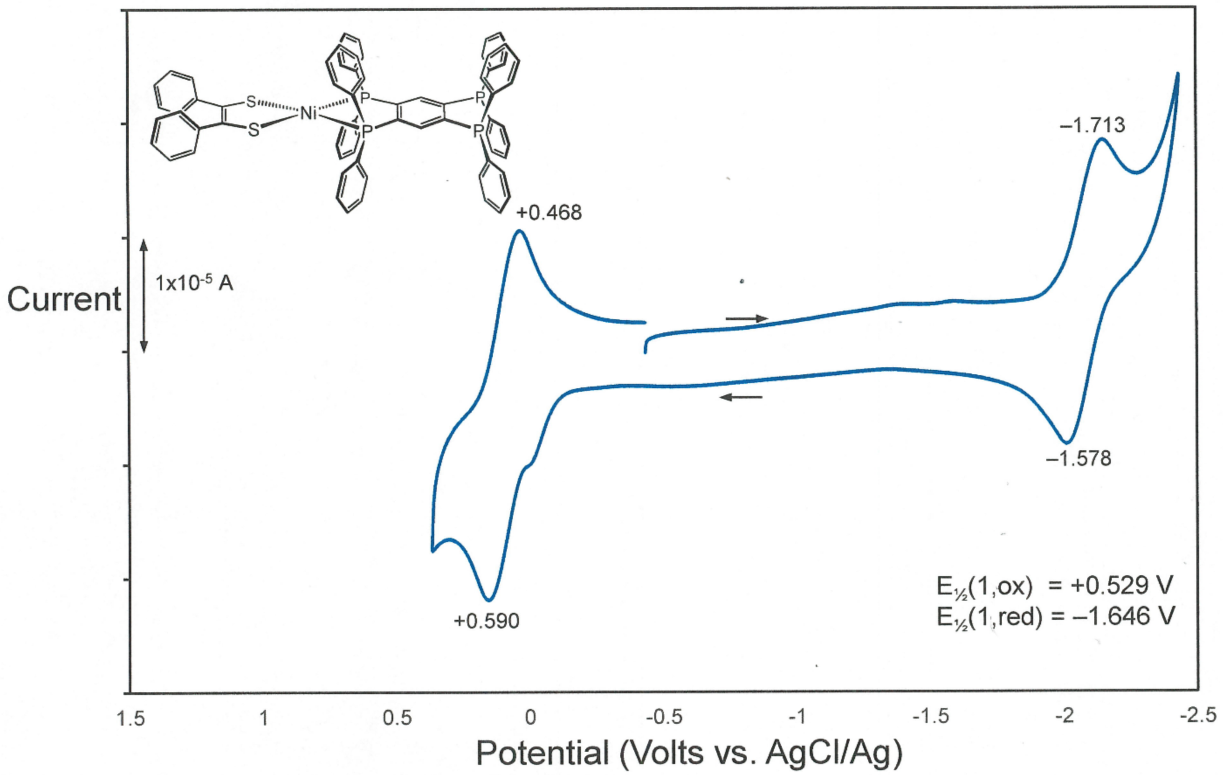

Figure S57. Cyclic voltammogram of [($Ph_2C_2S_2$)Ni(tpbz)] in CH_2Cl_2 with [^{n}Bu$_4$N][PF_6] supporting electrolyte, glassy carbon working electrode, Pt wire counter electrode and AgCl/Ag reference electrode. The scan rate was 100 mV/s.

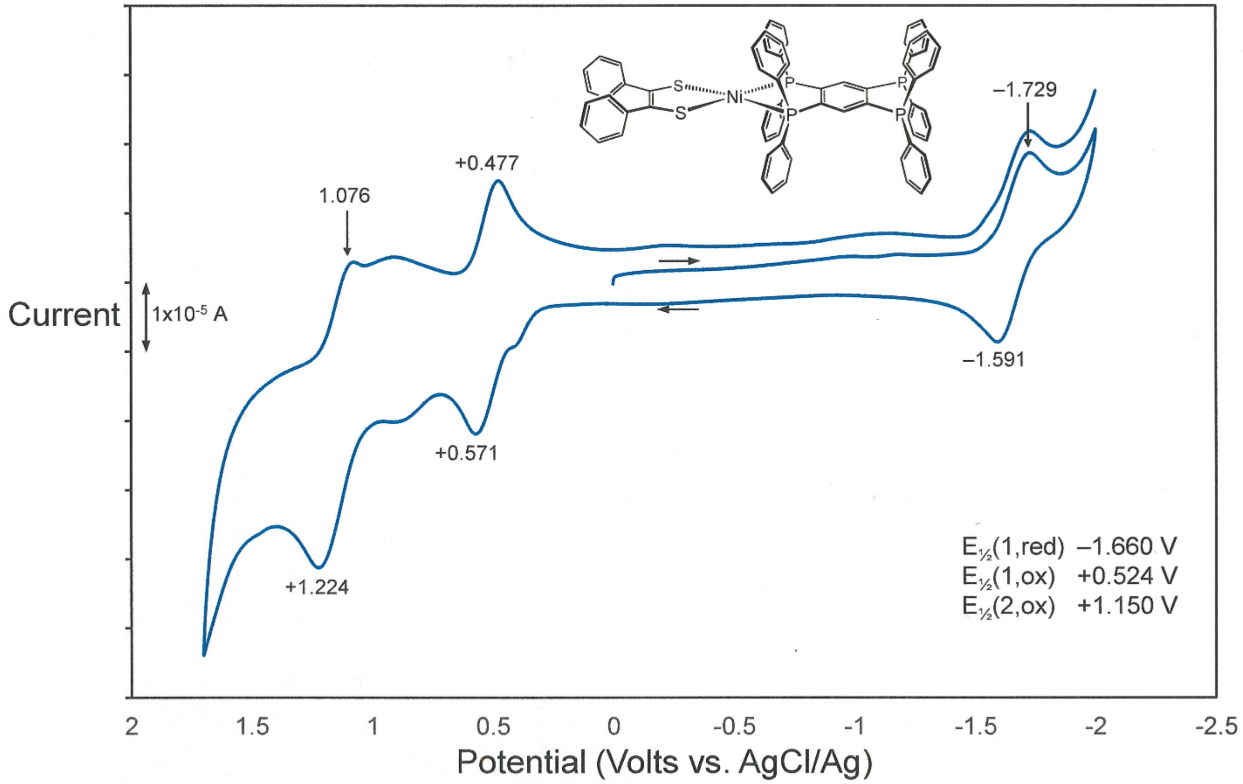

Figure S58. Cyclic voltammogram of [($Ph_2C_2S_2$)Ni(tpbz)] in CH_2Cl_2 with [^{n}Bu$_4$N][PF_6] supporting electrolyte, glassy carbon working electrode, Pt wire counter electrode and AgCl/Ag reference electrode. The scan rate was 100 mV/s.

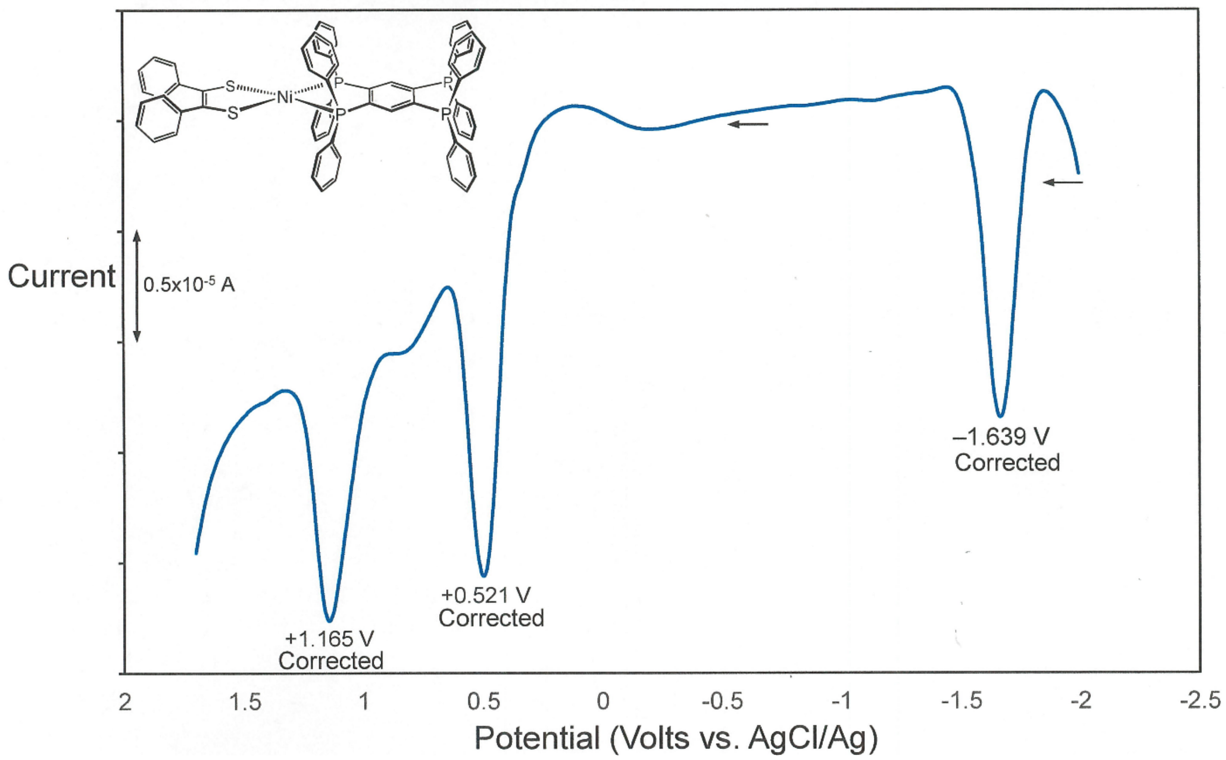

Figure S59. Differential pulse voltammogram of [($Ph_2C_2S_2$)Ni(tpbz)] in CH_2Cl_2 with [^{n}Bu$_4$N][PF_6] supporting electrolyte, glassy carbon working electrode, Pt wire counter electrode and AgCl/Ag reference electrode. The pulse amplitude was 50 mV.

James P. Donahue
Dept. of Chemistry, Tulane University
6400 Freret Street
Name New Orleans, LA 70118-5698
City Zip

JPD-125
SAMPLE IDENTIFICATION

Date April 28, 2016

Analysis	Theory	% Found	
C	73.19%	72.99	
H	4.70%	4.78	
P	11.10%	11.24	
O	0.00%		
S	5.75%		
Ni	5.26%		

M.P./B.P.

Hygroscopic: Explosive:

Molecular Formula: $C_{68}H_{52}NiP_4S_2$

To Be Dried: No X Yes ☐ at °C

ANALYZE for: C, H, P X Single ☐ Duplicate

m/m Lab Established in 1664

MIDWEST MICROLAB, LLC
7212 N. SHADELAND AVE., SUITE 110 INDIANAPOLIS, IN 46250
PHONE (317) 849-6606 FAX (317) 849-8534

Analysis results may be emailed to donahue@tulane.edu .

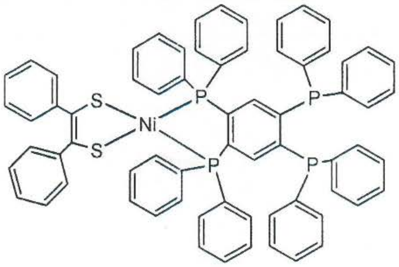

$C_{68}H_{52}NiP_4S_2$
1115.8724 g/mol

Received: MAY - 5 2016 Completed:

It is suggested that at least 5 mg. of sample be supplied per determination. Liquid samples should be suitably protected by metal foil in the cap if submitted in a screw cap vial. Volatile substances would best be sealed in small ampoules. All samples will be returned for postal charges only.

Figure S60. Elemental analysis of [$(Ph_2C_2S_2)Ni(tpbz)$] from Midwest Microlab, LLC. The form is reproduced with the permission of Midwest Microlab.

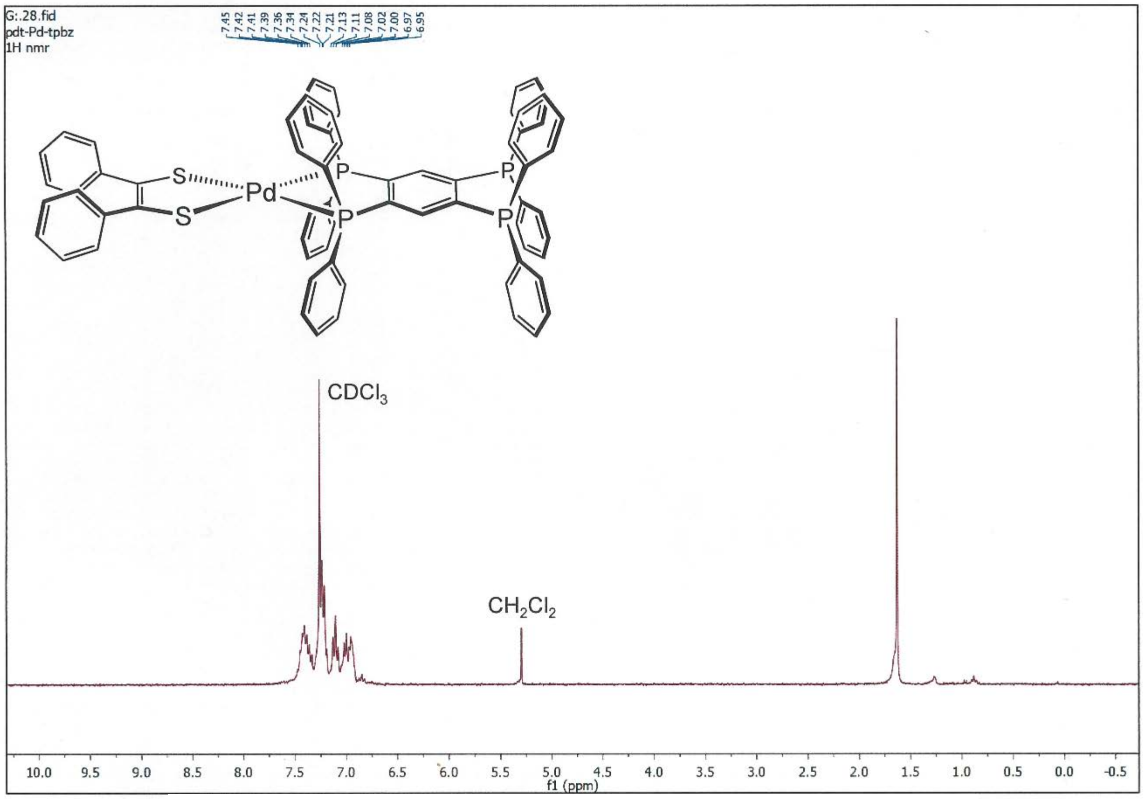

Figure S61. ^{1}H NMR spectrum ($CDCl_3$) of [($Ph_2C_2S_2$)Pd(tpbz)].

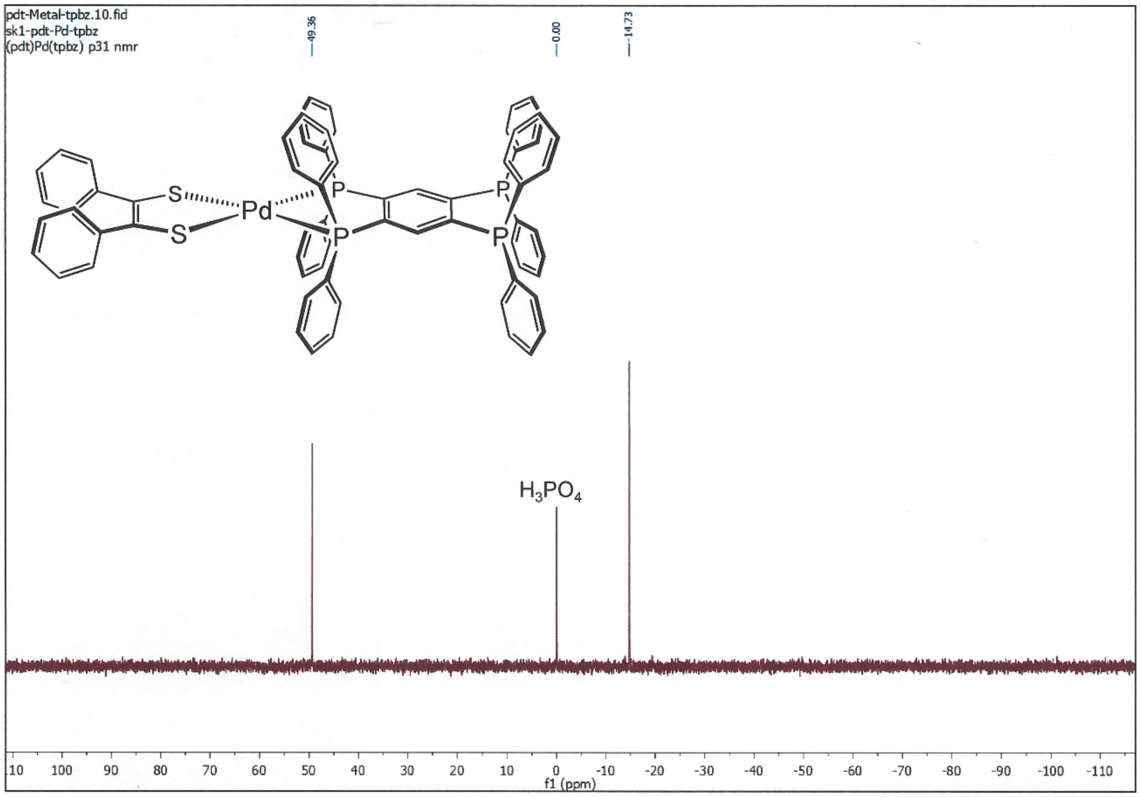

Figure S62. ^{31}P NMR spectrum ($CDCl_3$) of [($Ph_2C_2S_2$)Pd(tpbz)].

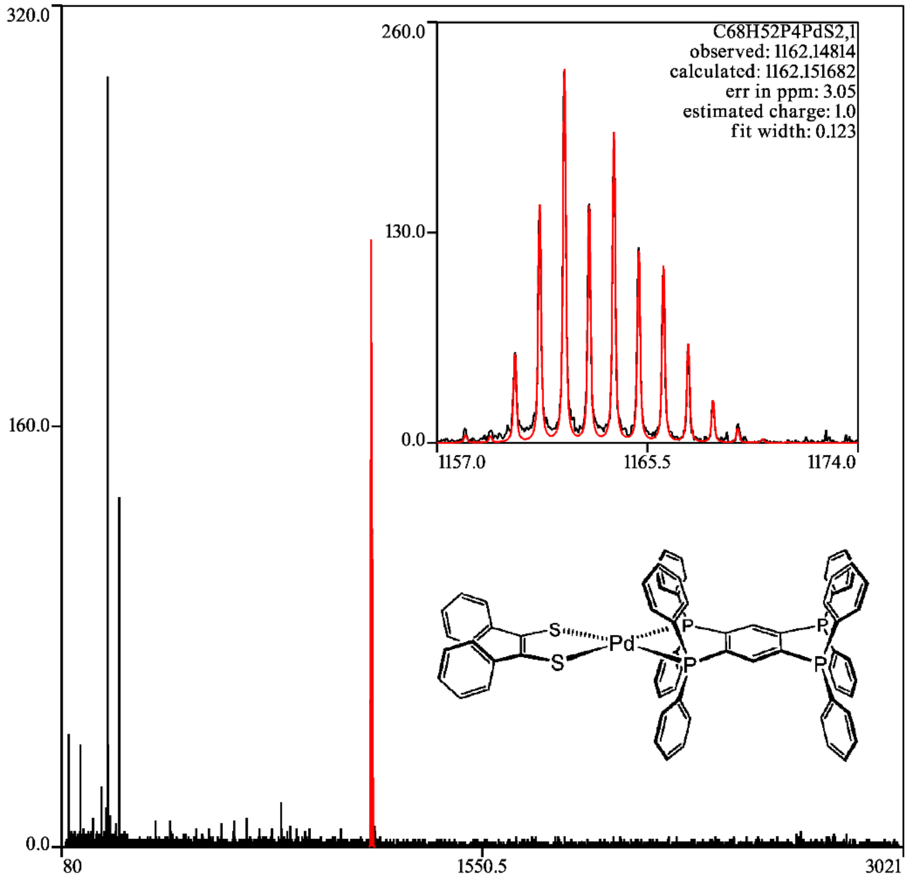

Figure S63. ESI mass spectrum (positive ion mode) of $[(Ph_2C_2S_2)Pd(tpbz)]$.

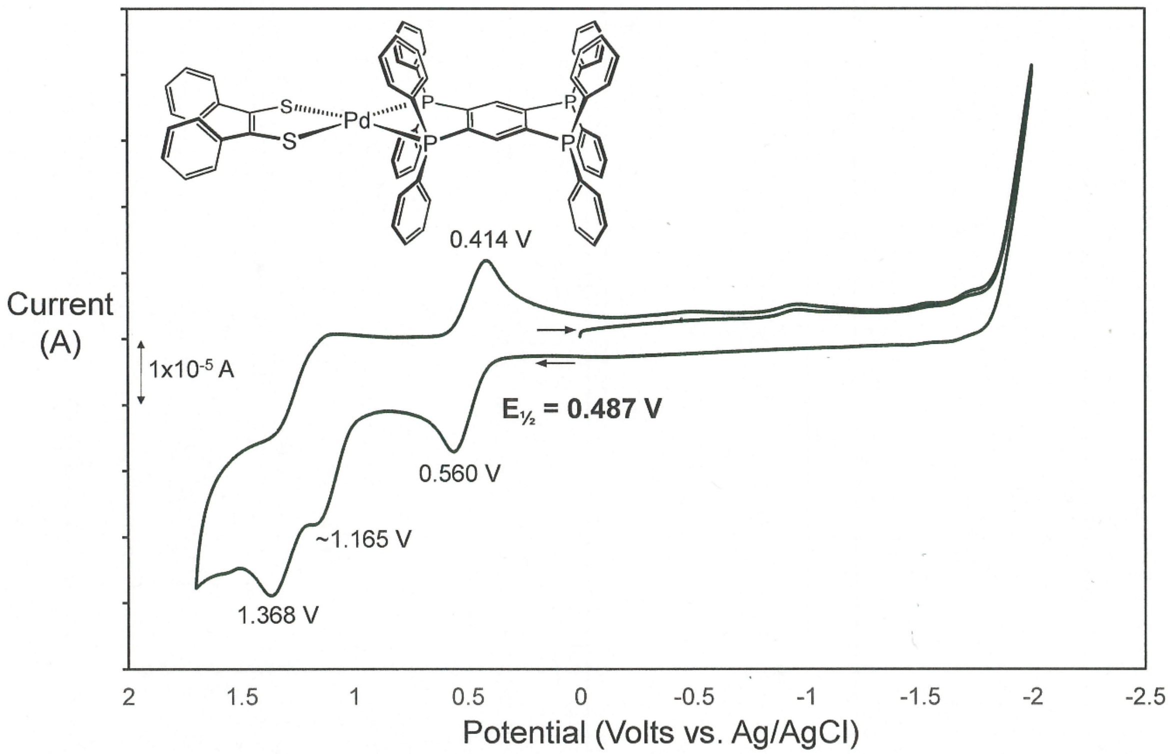

Figure S64. Cyclic voltammogram of [$(Ph_2C_2S_2)Pd(tpbz)$] in CH_2Cl_2 with [$^{n}Bu_4N$][PF_6] supporting electrolyte, glassy carbon working electrode, Pt wire counter electrode and AgCl/Ag reference electrode. The scan rate was 100 mV/s.

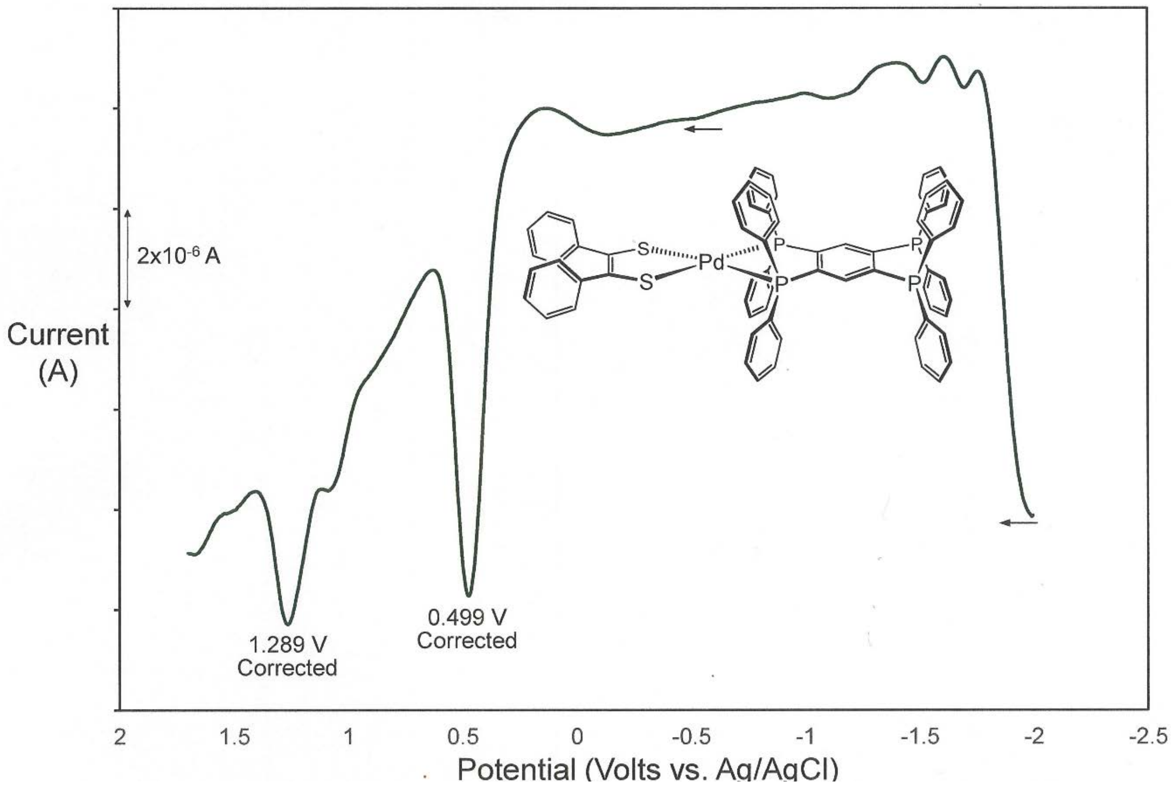

Figure S65. Differential pulse voltammogram of [$(Ph_2C_2S_2)Ni(tpbz)$] in CH_2Cl_2 with [$^{n}Bu_4N$][PF_6] supporting electrolyte, glassy carbon working electrode, Pt wire counter electrode and AgCl/Ag reference electrode. The pulse amplitude was 50 mV.

Report Number: 102062 GALBRAITH LABORATORIES INC. Report Date: 2017-11-07

Laboratory Report

Report prepared for:
James P Donahue
Tulane Univ
Dept of Chem
Stern Hall, Room 2015
6400 Freret St
New Orleans, LA 70118
Phone: 504-862-3562
Email: donahue@tulane.edu

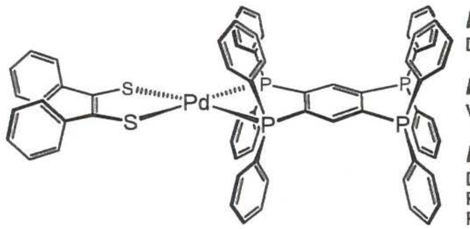

Report prepared by:
Debbie S Robertson

Purchase Order:
VISA,Sanchez,10/23/17

For further assistance, contact:
Debbie S Robertson
Report Production Coordinator
PO Box 51610
Knoxville, TN 37950 -1610
(865) 546-1335
debbierobertson@galbraith.com

Anal. Calcd for [($Ph_2C_2S_2$)Pd(tpbz)], $C_{68}H_{52}PdP_4S_2$: C, 70.19; H, 4.50; P, 10.65.

Sample: JPD148
Lab ID: 2017-F-3186 **Received:** 2017-10-23

Analysis	Method	Result	Basis	Sample Amount Used	Date (Time)
C : Carbon					
	GLI Procedure ME-14	69.57 %	As Received	1.887 mg	2017-10-31
H : Hydrogen					
	GLI Procedure ME-14	4.17 %	As Received	1.887 mg	2017-10-31
P : Phosphorus					
	GLI Procedure ME-70	9.83 %	As Received	4.139 mg	2017-11-03

For all samples on this report:

1. There was insufficient sample provided to perform more than one replicate on the analyses.

Signatures:

Published By:	Debbie.S.Robertson	2017-11-07T15:53:42.223-05:00
Created By:	Debbie.S.Robertson	2017-11-07T15:53:17.387-05:00

- Physical signatures are on file.
- "Published By" signature indicates authorized release of data.

Copyright 2017 Galbraith Laboratories, Inc.
Reported results are only applicable to the item tested.
This report shall not be reproduced, except in full, without the written approval of the laboratory.

Page 1 of 1

Figure S66. Elemental analysis of [($Ph_2C_2S_2$)Pd(tpbz)] from Galbraith Laboratories, Inc. The form is reproduced with the permission of Galbraith Laboratories.

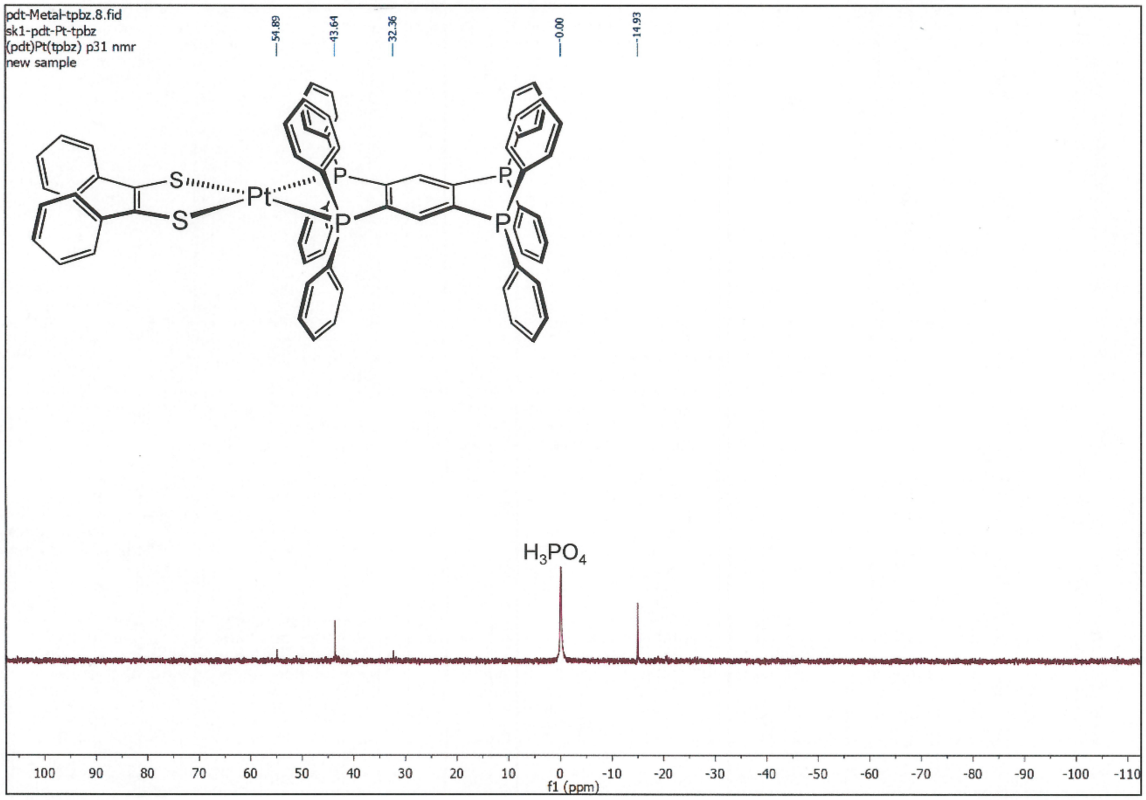

Figure S67. ^{31}P NMR spectrum ($CDCl_3$) of [($Ph_2C_2S_2$)Pd(tpbz)].

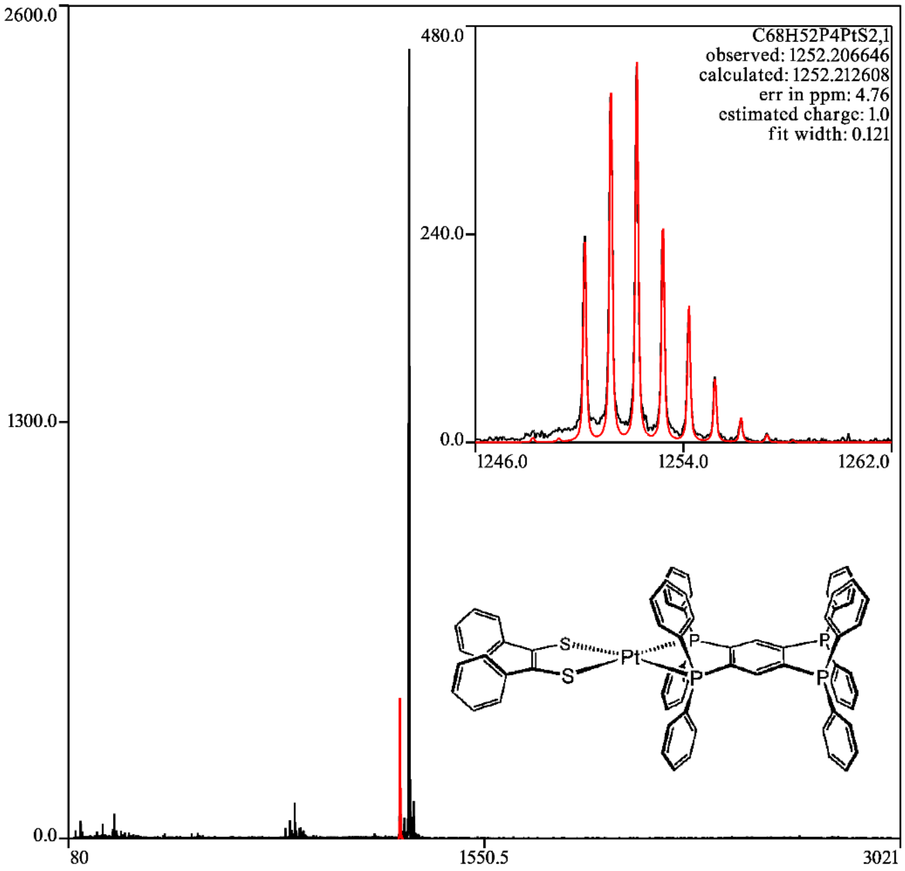

Figure S68. ESI mass spectrum (positive ion mode) of [($Ph_2C_2S_2$)Pt(tpbz)].

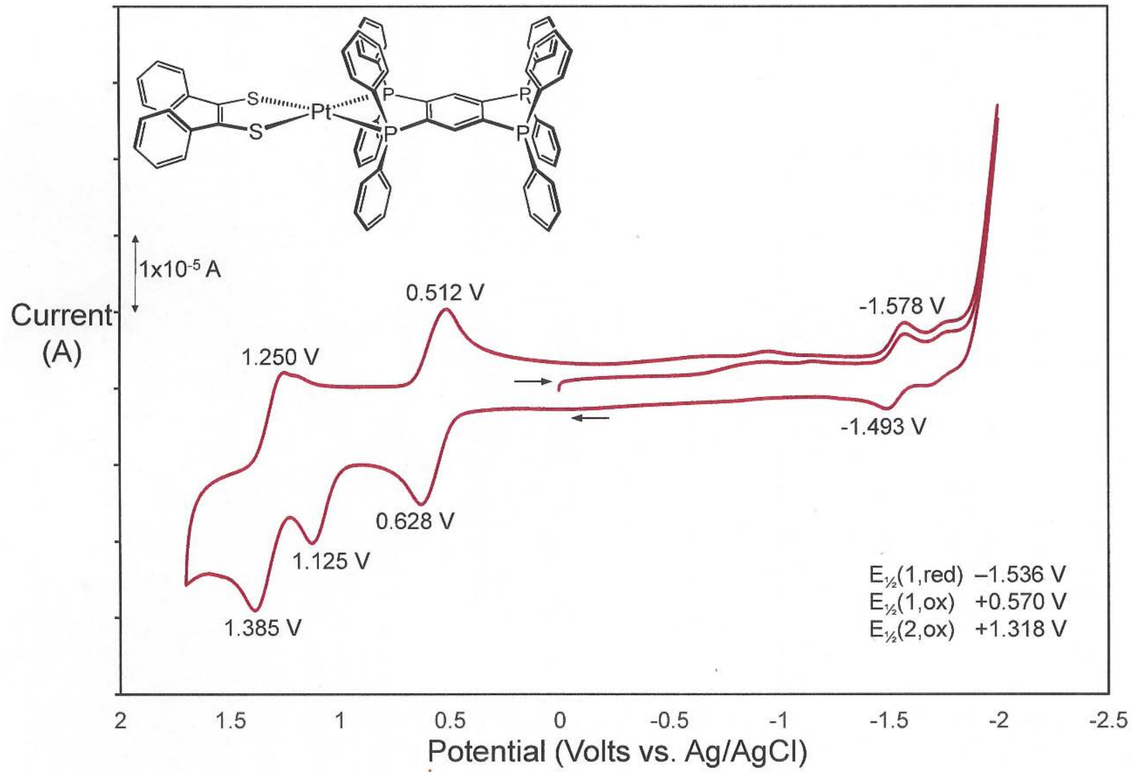

Figure S69. Cyclic voltammogram of [($Ph_2C_2S_2$)Pt(tpbz)] in CH_2Cl_2 with [$^{n}Bu_4N$][PF_6] supporting electrolyte, glassy carbon working electrode, Pt wire counter electrode and AgCl/Ag reference electrode. The scan rate was 100 mV/s.

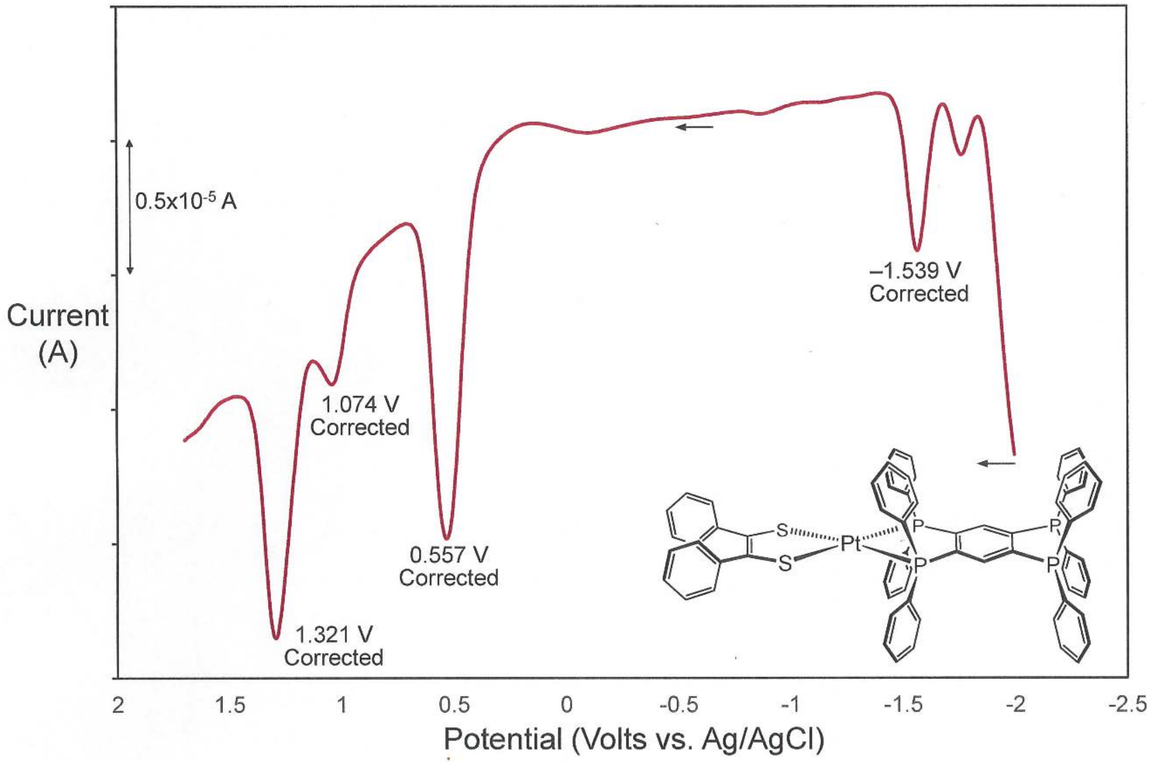

Figure S70. Differential pulse voltammogram of [($Ph_2C_2S_2$)Pt(tpbz)] in CH_2Cl_2 with [$^{n}Bu_4N$][PF_6] supporting electrolyte, glassy carbon working electrode, Pt wire counter electrode and AgCl/Ag reference electrode. The pulse amplitude was 50 mV.

Report Number: 105844 **GALBRAITH** LABORATORIES, INC. Report Date: 2018-04-20

Laboratory Report

Report prepared for:

James P Donahue
Tulane Univ
Dept of Chem
Stern Hall, Room 2015
6400 Freret St
New Orleans, LA 70118
Phone: 504-862-3562
Email: donahue@tulane.edu

Report prepared by:

Debbie S Robertson

Purchase Order:

Visa,Sanchez,4/17/18

For further assistance, contact:

Debbie S Robertson
Report Production Coordinator
PO Box 51610
Knoxville, TN 37950 -1610
(865) 546-1335
debbierobertson@galbraith.com

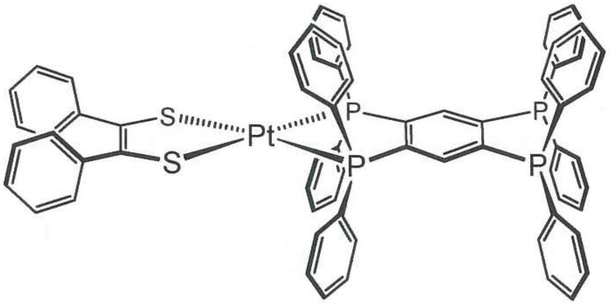

Anal. Calcd for [($Ph_2C_2S_2$)Pt(tpbz)], $C_{68}H_{52}PtP_4S_2$: C, 65.22; H, 4.19.

Sample: JPD156
Lab ID: 2018-G-3862 **Received:** 2018-04-17

Analysis	Method	Result	Basis	Sample Amount Used	Date (Time)
C : Carbon					
	GLI Procedure ME-14 [2]	65.22 %	As Received	1.492 mg	2018-04-18
	GLI Procedure ME-14	64.53 %	As Received	2.852 mg	2018-04-18
H : Hydrogen					
	GLI Procedure ME-14	4.13 %	As Received	2.852 mg	2018-04-18

2. Additional carbon test results were generated because the nitrogen test had to be repeated. There is no additional charge for the additional carbon data point.

Copyright 2018 Galbraith Laboratories, Inc.
Reported results are only applicable to the item tested.
This report shall not be reproduced, except in full, without the written approval of the laboratory.

Page 1 of 2

Figure S71. Elemental analysis of [($Ph_2C_2S_2$)Pt(tpbz)] from Galbraith Laboratories, Inc. The form is reproduced with the permission of Galbraith Laboratories.

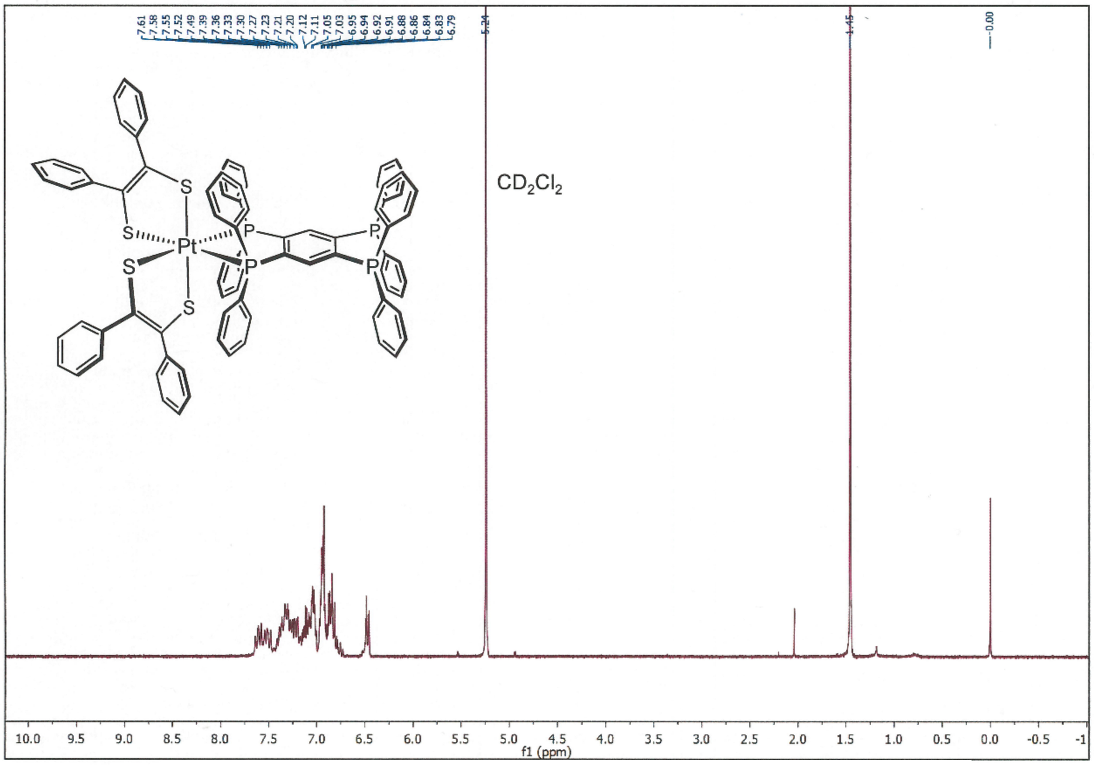

Figure S72. ^{1}H NMR spectrum (CD_2Cl_2) of [$(Ph_2C_2S_2)_2Pd$(tpbz)].

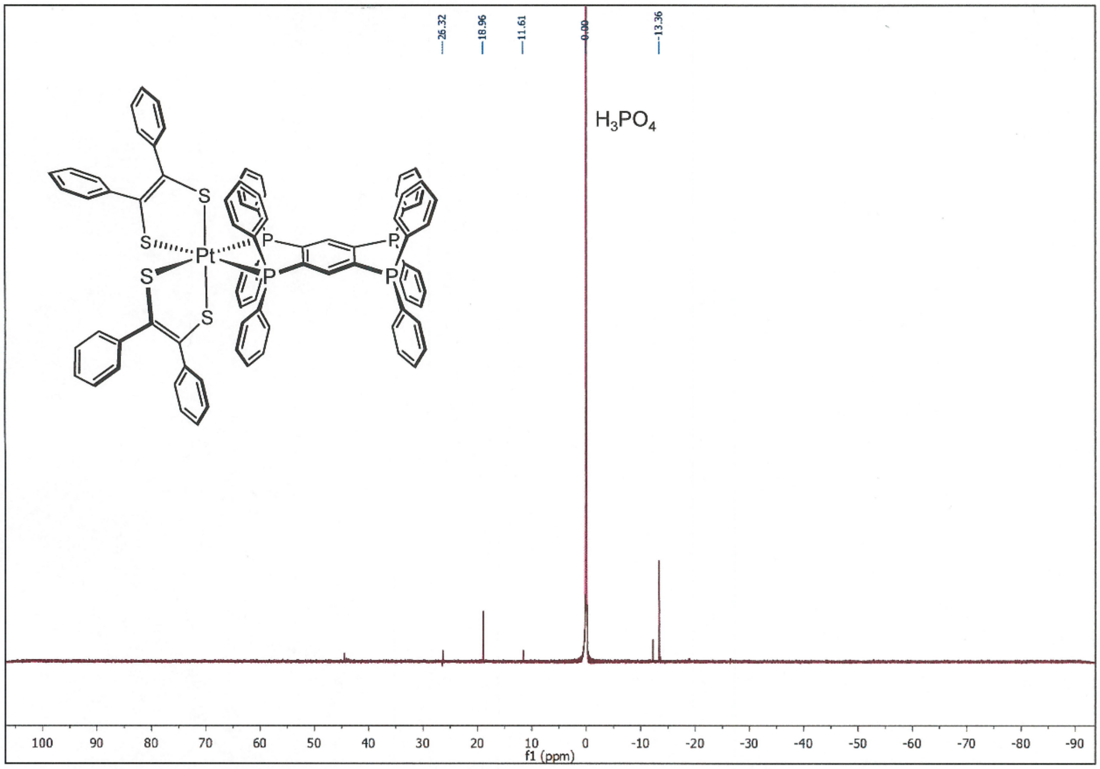

Figure S73. ^{31}P NMR spectrum (CD_2Cl_2) of [$(Ph_2C_2S_2)_2Pd$(tpbz)].

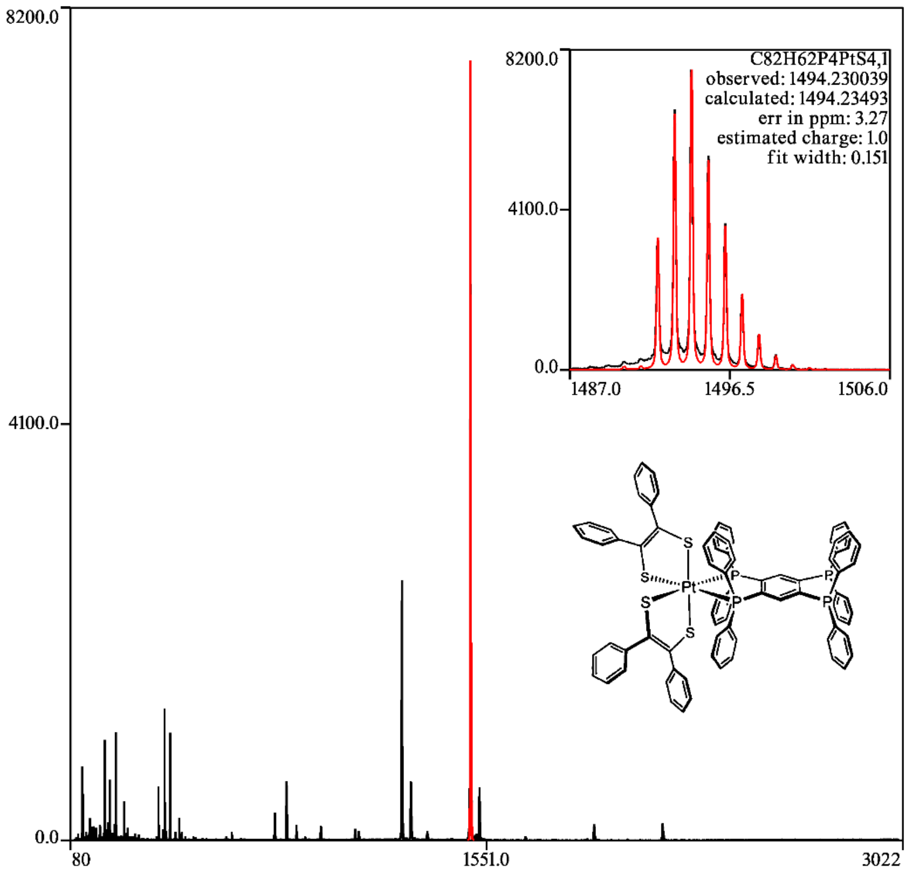

Figure S74. ESI mass spectrum (positive ion mode) of $[(Ph_2C_2S_2)_2Pt(tpbz)]$.

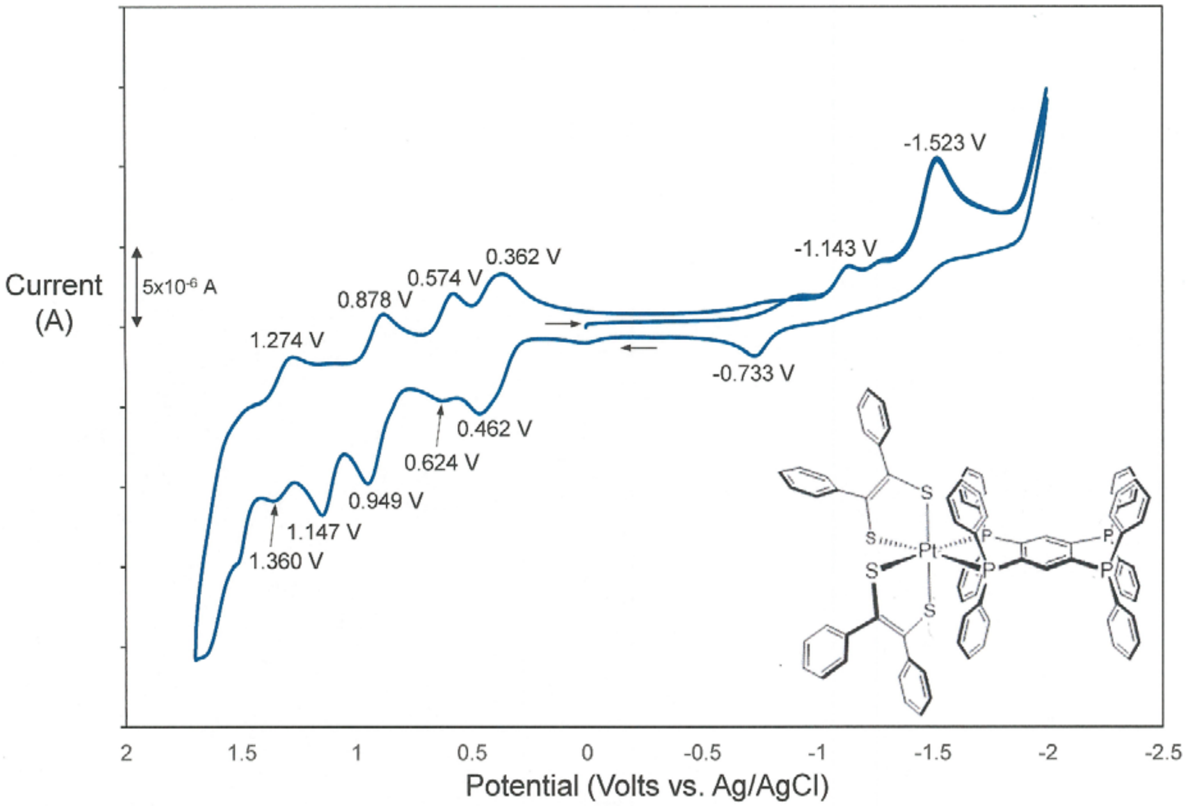

Figure S75. CV of $[(Ph_2C_2S_2)_2Pt(tpbz)]$ in CH_2Cl_2 with $[^nBu_4N][PF_6]$ supporting electrolyte, Pt disk working electrode, Pt wire counter electrode and AgCl/Ag reference electrode. The scan rate was 100 mV/s.

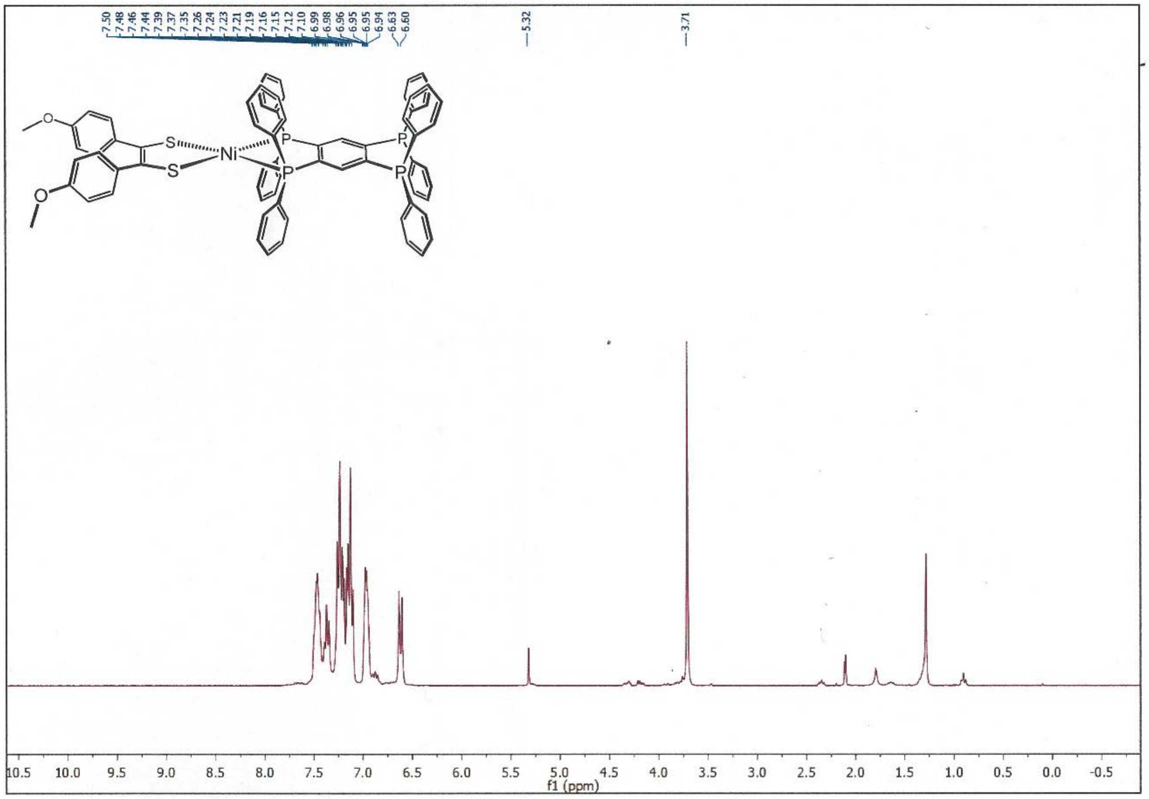

Figure S76. ^{1}H NMR spectrum of [((MeO-*p*-$C_6H_4)_2C_2S_2$)Ni(tpbz)] in $CDCl_3$.

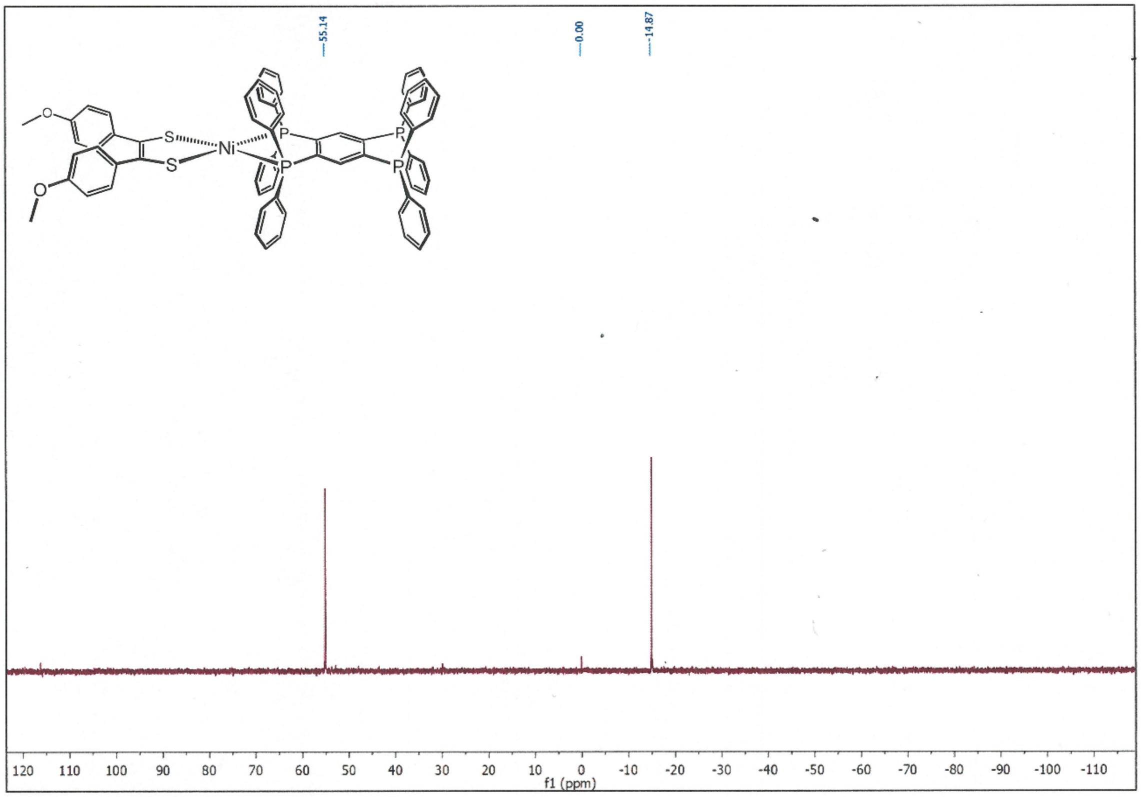

Figure S77. ^{31}P NMR spectrum of [((MeO-*p*-$C_6H_4)_2C_2S_2$)Ni(tpbz)] in $CDCl_3$.

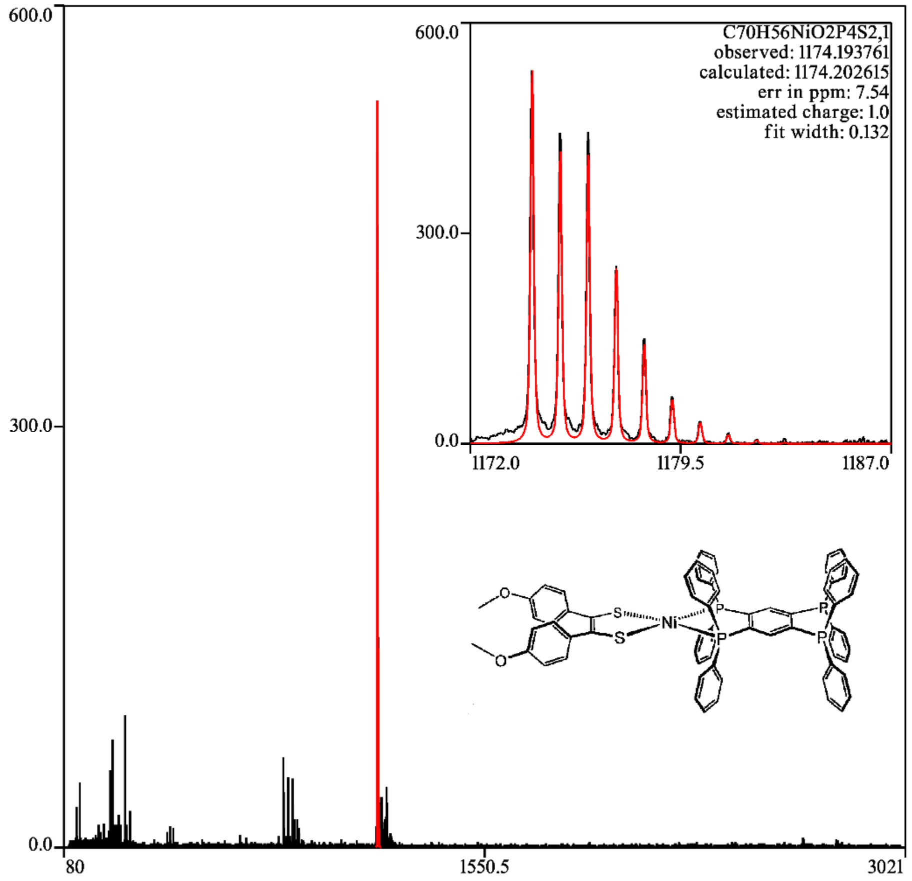

Figure S78. ESI mass spectrum (positive ion mode) of [((MeO-*p*-$C_6H_4)_2C_2S_2$)Ni(tpbz)].

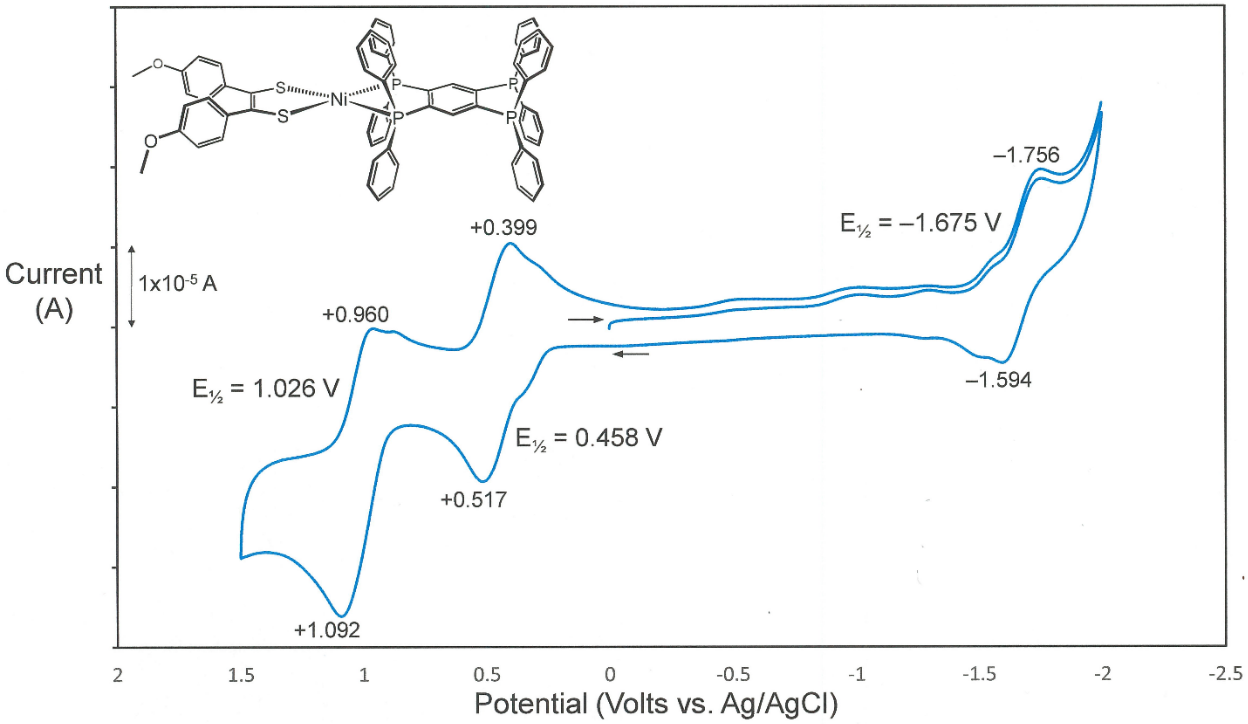

Figure S79. Cyclic voltammogram of [((CH_3O-*p*-$C_6H_4)_2C_2S_2$)Ni(tpbz)] in CH_2Cl_2 with [^{n}Bu_4N][PF_6] supporting electrolyte, glassy carbon working electrode, Pt wire counter electrode and AgCl/Ag reference electrode. The scan rate was 100 mV/s.

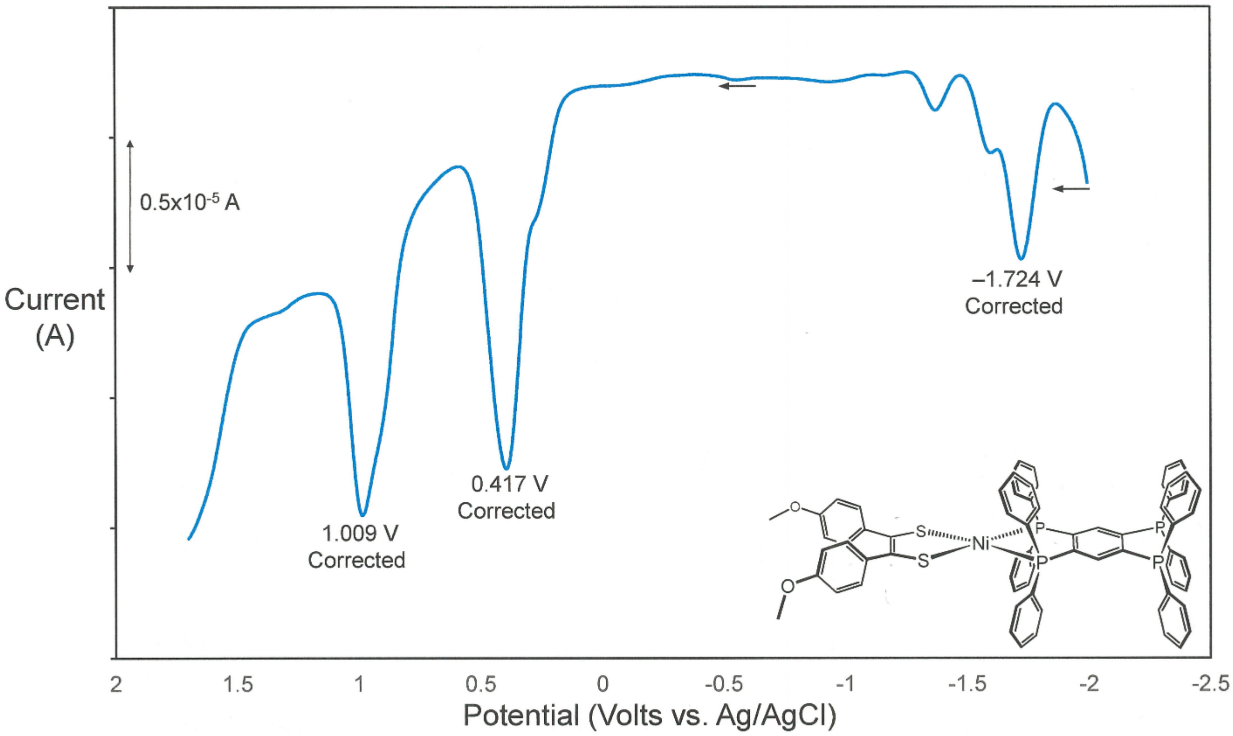

Figure S80. Differential pulse voltammogram, moving in the direction of positive potential, of [((CH_3O-*p*-C_6H_4)$_2C_2S_2$)Ni(tpbz)] in CH_2Cl_2 with [^{n}Bu_4N][PF_6] supporting electrolyte, glassy carbon working electrode, Pt wire counter electrode and AgCl/Ag reference electrode. The pulse amplitude was 50 mV.

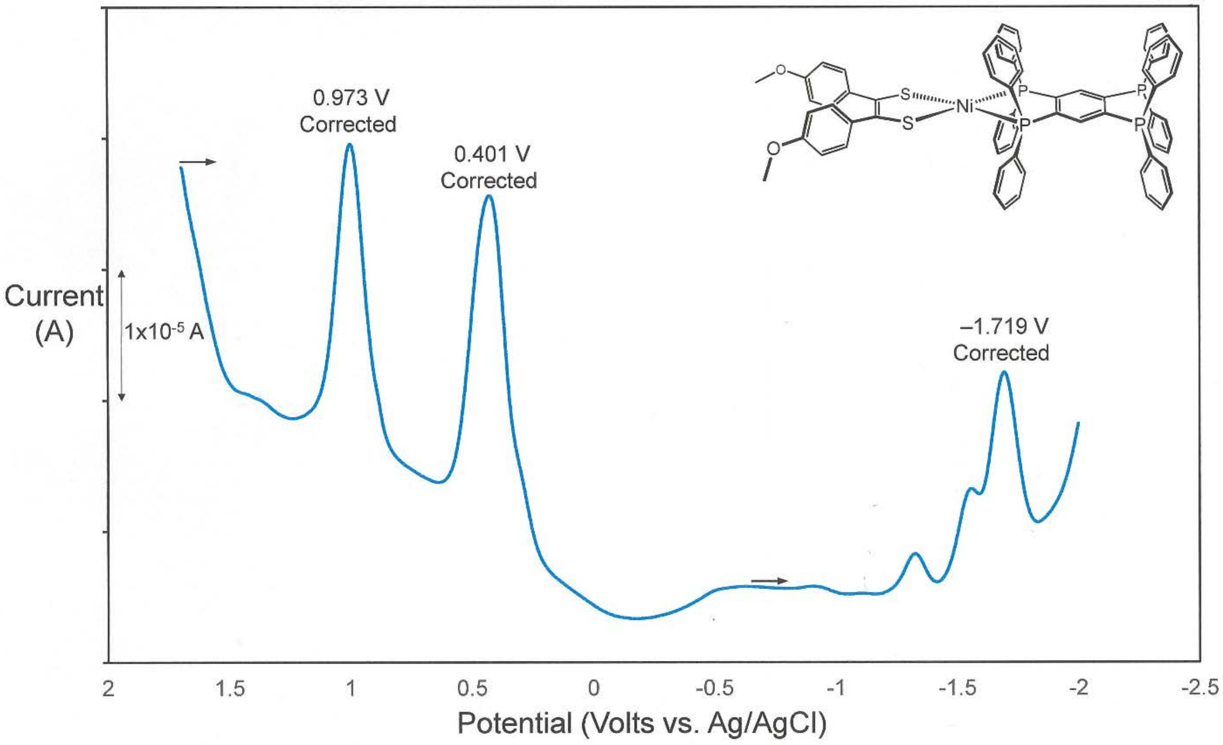

Figure S81. Differential pulse voltammogram, moving in the direction of negative potential, of [((CH_3O-*p*-C_6H_4)$_2C_2S_2$)Ni(tpbz)] in CH_2Cl_2 with [^{n}Bu_4N][PF_6] supporting electrolyte, glassy carbon working electrode, Pt wire counter electrode and AgCl/Ag reference electrode. The pulse amplitude was 50 mV.

MIKROLAB
Mikroanalytisches Laboratorium Kolbe

Professor James P. Donahue
Department of Chemistry
Tulane University
6400 Freret St.
New Orleans, Louisiana 70118-5698, USA

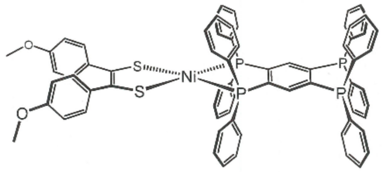

Address : Osterfelder Str. 3
D-46047 Oberhausen
Phone : +49 - (0)208 - 32502
Fax : +49 - (0)208 - 382314
Email : info@mikro-lab.de
Website : www.mikro-lab.de

Date : 03.05.2019

Sample Name	% C	% H	% N	% P	% S						Argon	V2
JPD 175 - b	71,29	4,86		10,41	5,51						x	[illegible]
											x	[illegible]

Kind regards
Anal. Calcd for [((MeO-*p*-C_6H_4)$_2$$C_2S_2$)Ni(tpbz)], [$C_{70}H_{56}NiO_2P_4S_2$]: C, 71.50; H, 4.80; P, 10.54; S, 5.45.

Patrick Springer

P.S.

Figure S82. Elemental analysis of [((MeO-*p*-C_6H_4)$_2$$C_2S_2$)Ni(tpbz)] from Kolbe Microanalytical Laboratory. The form is reproduced with the permission of Kolbe Microanalytical Laboratory.

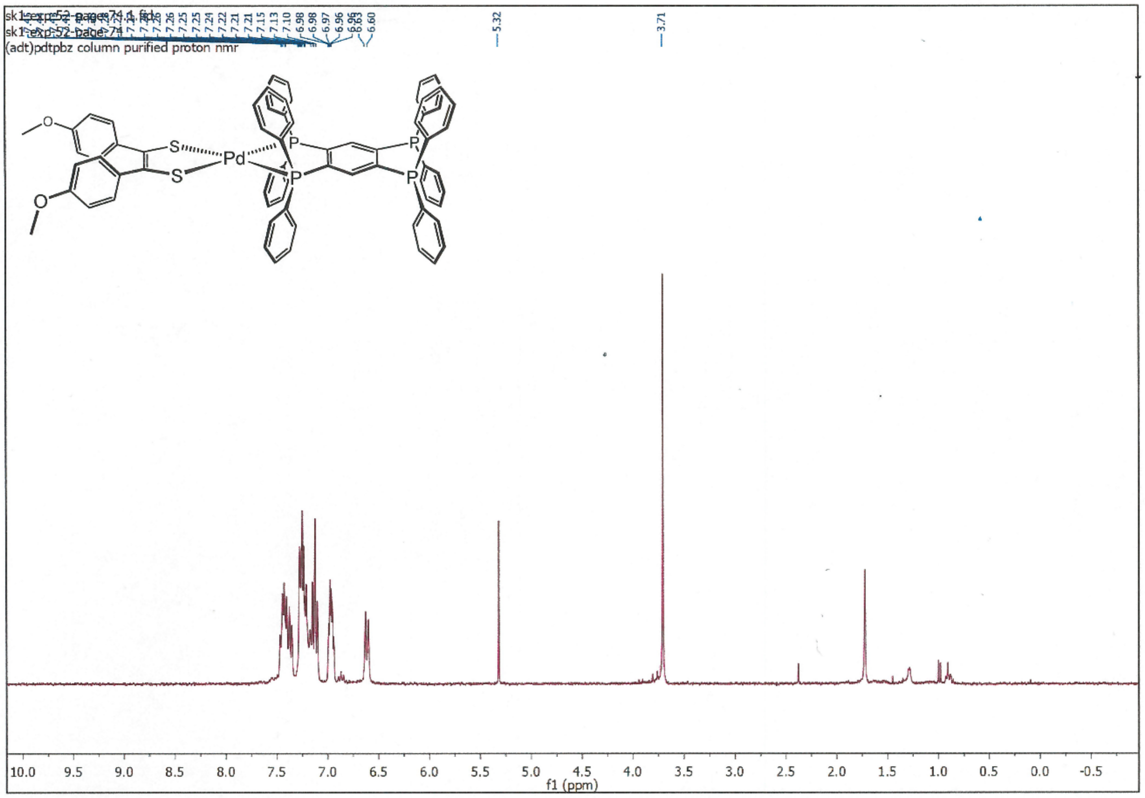

Figure S83. ^{1}H NMR spectrum of [((MeO-*p*-C_6H_4)$_2$$C_2S_2$)Pd(tpbz)] in $CDCl_3$.

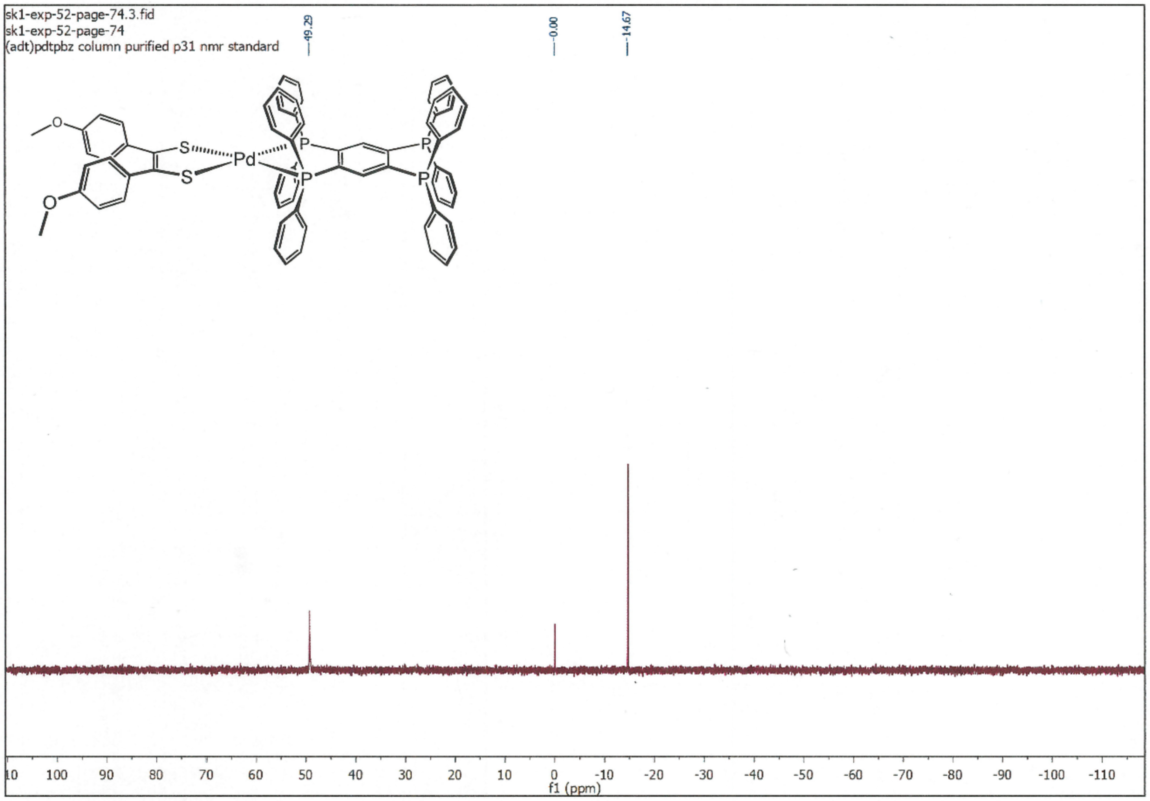

Figure S84. ^{31}P NMR spectrum of [((MeO-*p*-C_6H_4)$_2$$C_2S_2$)Pd(tpbz)] in $CDCl_3$.

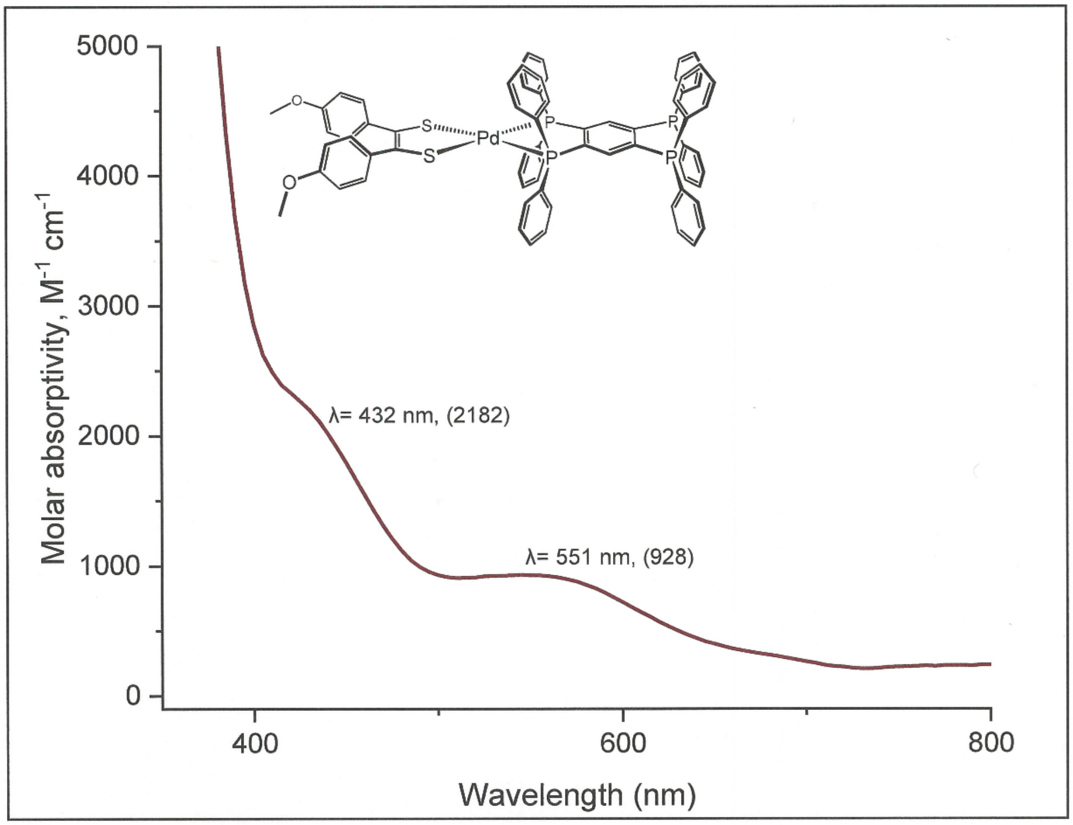

Figure S85. UV-vis spectrum (CH_2Cl_2) of [((MeO-*p*-C_6H_4)$_2$$C_2S_2$)Pd(tpbz)].

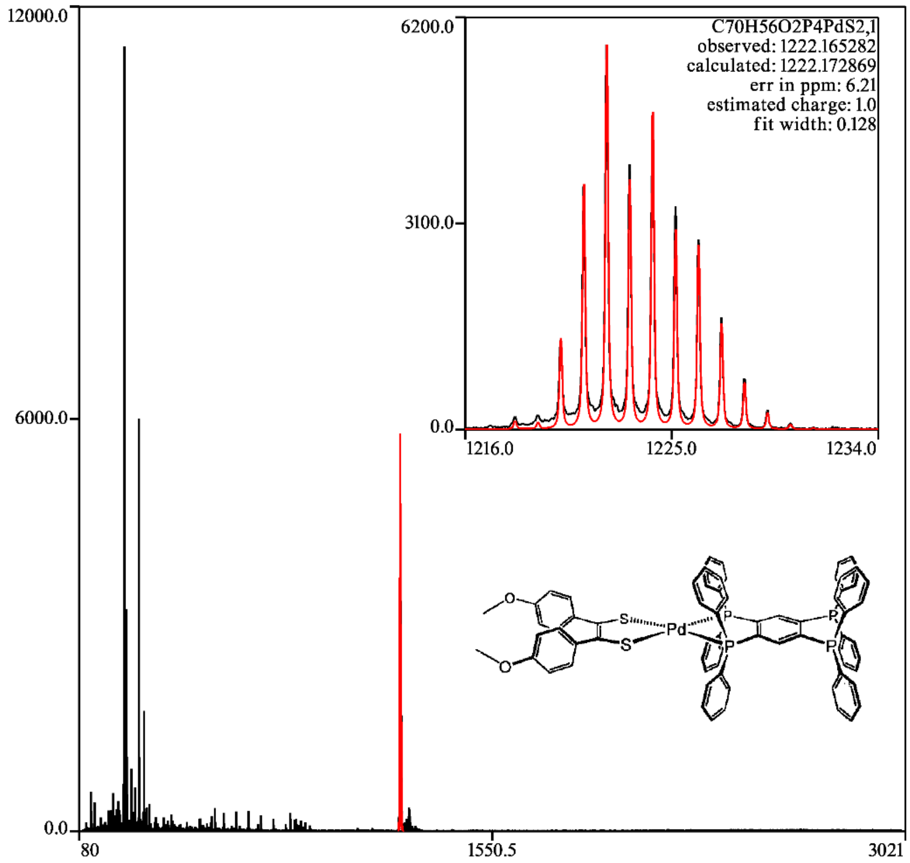

Figure S86. ESI mass spectrum (positive ion mode) of [((MeO-*p*-C_6H_4)$_2$$C_2S_2$)Pd(tpbz)].

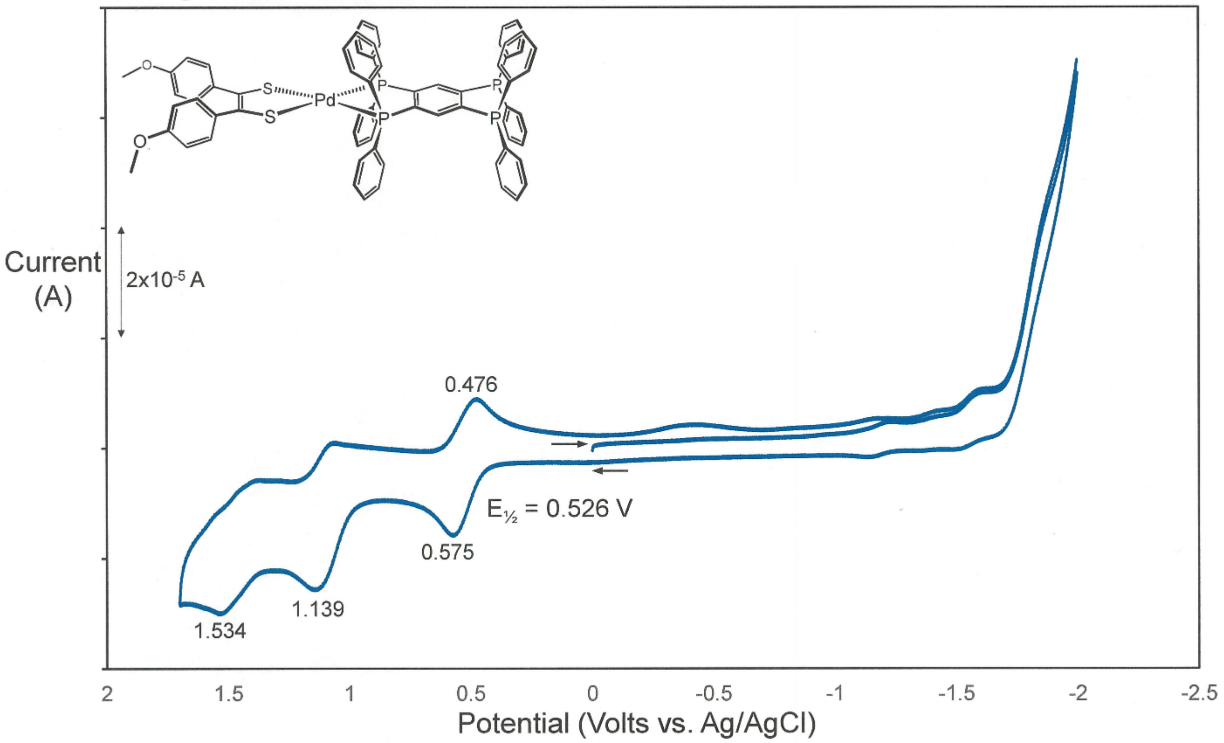

Figure S87. Cyclic voltammogram of $[((CH_3O\text{-}p\text{-}C_6H_4)_2C_2S_2)Pd(tpbz)]$ in CH_2Cl_2 with $[^nBu_4N][PF_6]$ supporting electrolyte, glassy carbon working electrode, Pt wire counter electrode and AgCl/Ag reference electrode. The scan rate was 100 mV/s.

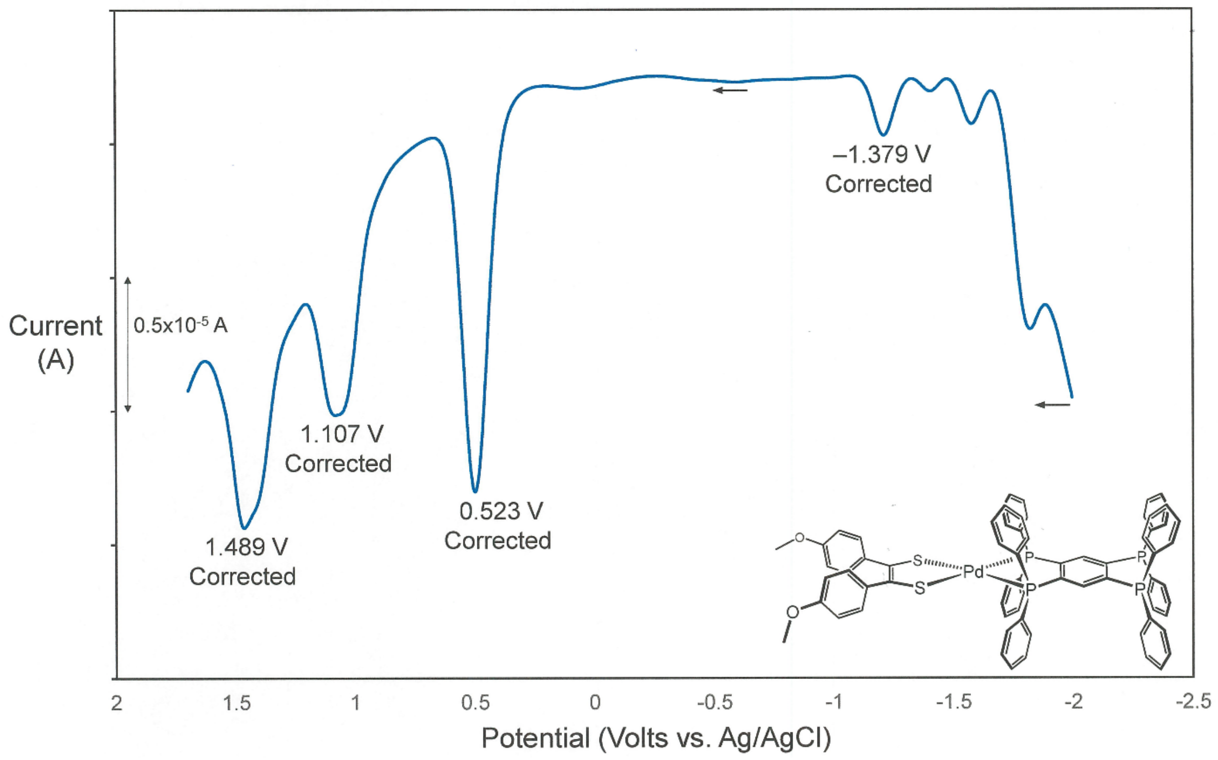

Figure S88. Differential pulse voltammogram, moving in the direction of positive potential, of [((CH$_3$O-*p*-C$_6$H$_4$)$_2$C$_2$S$_2$)Pd(tpbz)] in CH$_2$Cl$_2$ with [^{n}Bu$_4$N][PF$_6$] supporting electrolyte, glassy carbon working electrode, Pt wire counter electrode and AgCl/Ag reference electrode. The pulse amplitude was 50 mV.

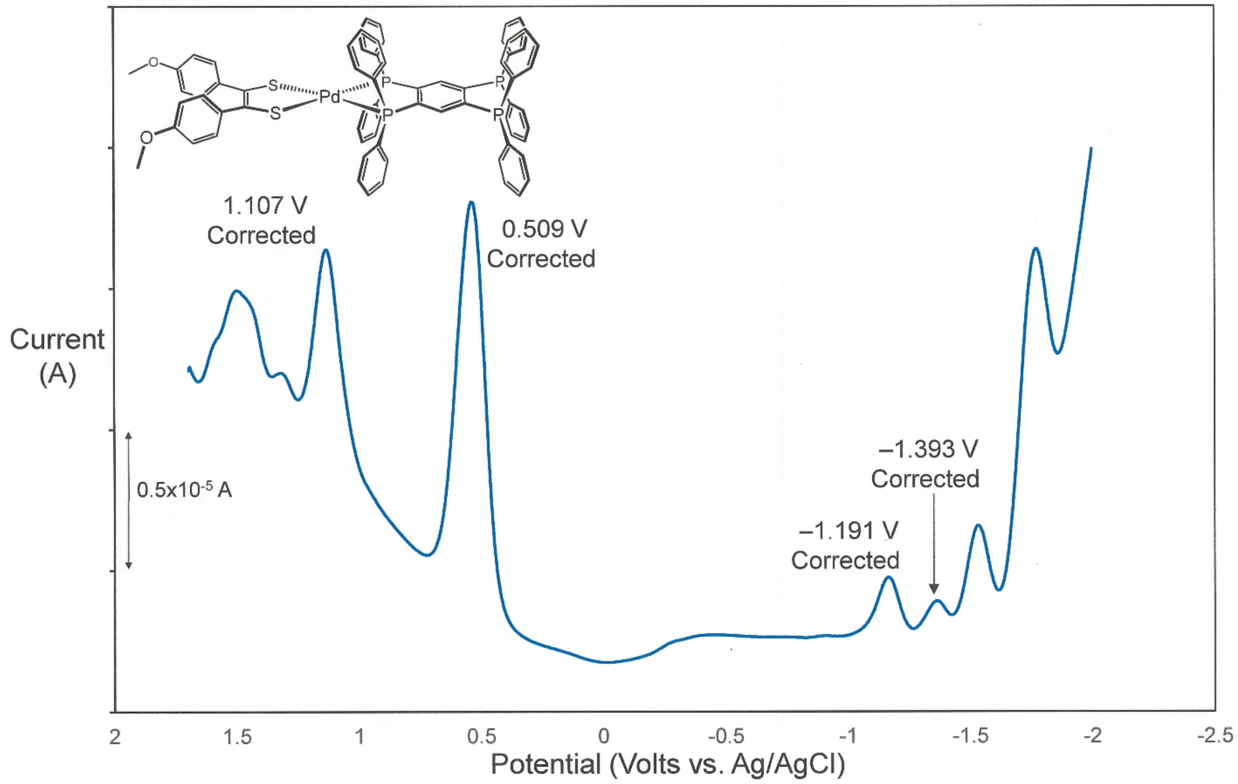

Figure S89. Differential pulse voltammogram, moving in the direction of negative potential, of [((CH$_3$O-*p*-C$_6$H$_4$)$_2$C$_2$S$_2$)Pd(tpbz)] in CH$_2$Cl$_2$ with [^{n}Bu$_4$N][PF$_6$] supporting electrolyte, glassy carbon working electrode, Pt wire counter electrode and AgCl/Ag reference electrode. The pulse amplitude was 50 mV.

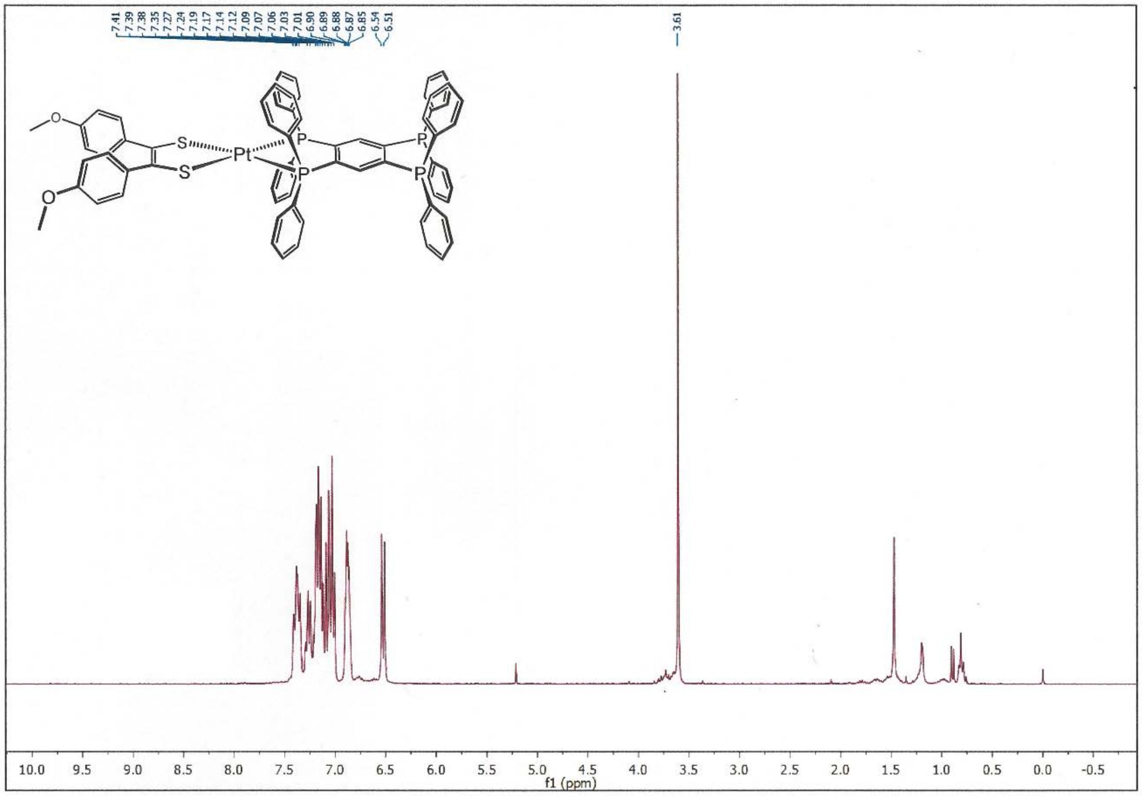

Figure S90. ^{1}H NMR spectrum of [((MeO-*p*-C_6H_4)$_2C_2S_2$)Pt(tpbz)] in $CDCl_3$.

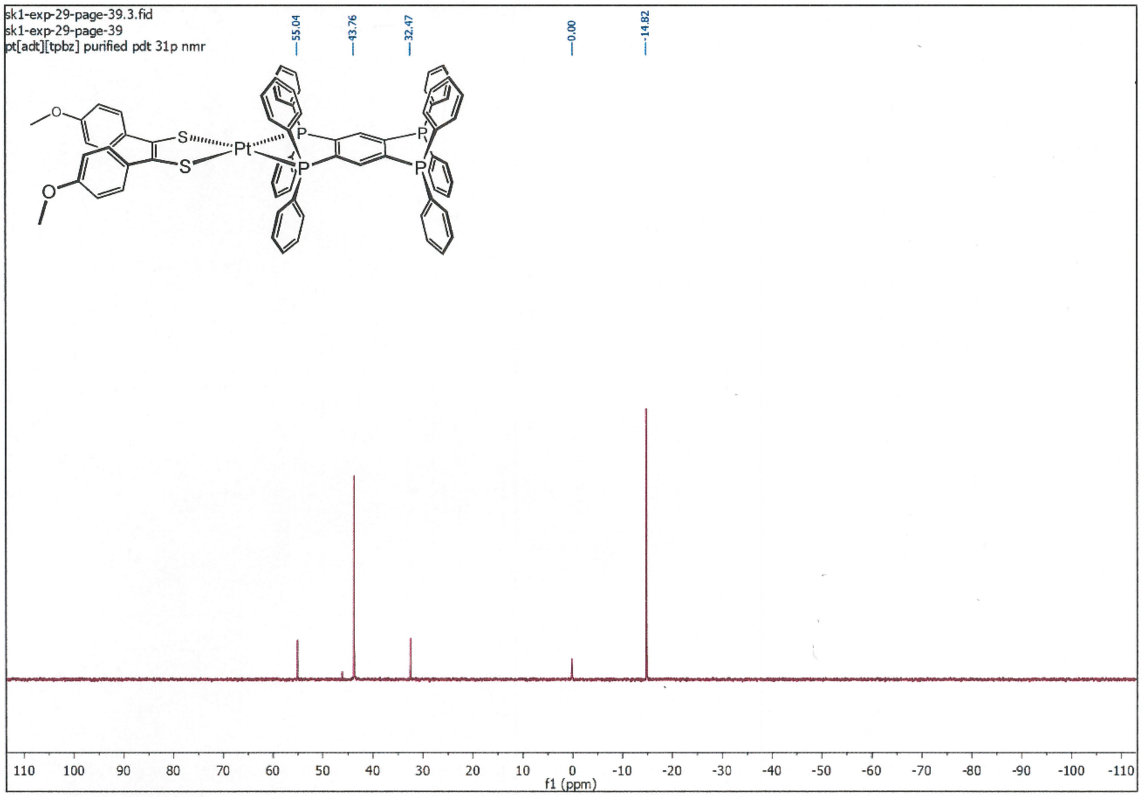

Figure S91. ^{31}P NMR spectrum of [((MeO-*p*-C_6H_4)$_2C_2S_2$)Pt(tpbz)] in $CDCl_3$.

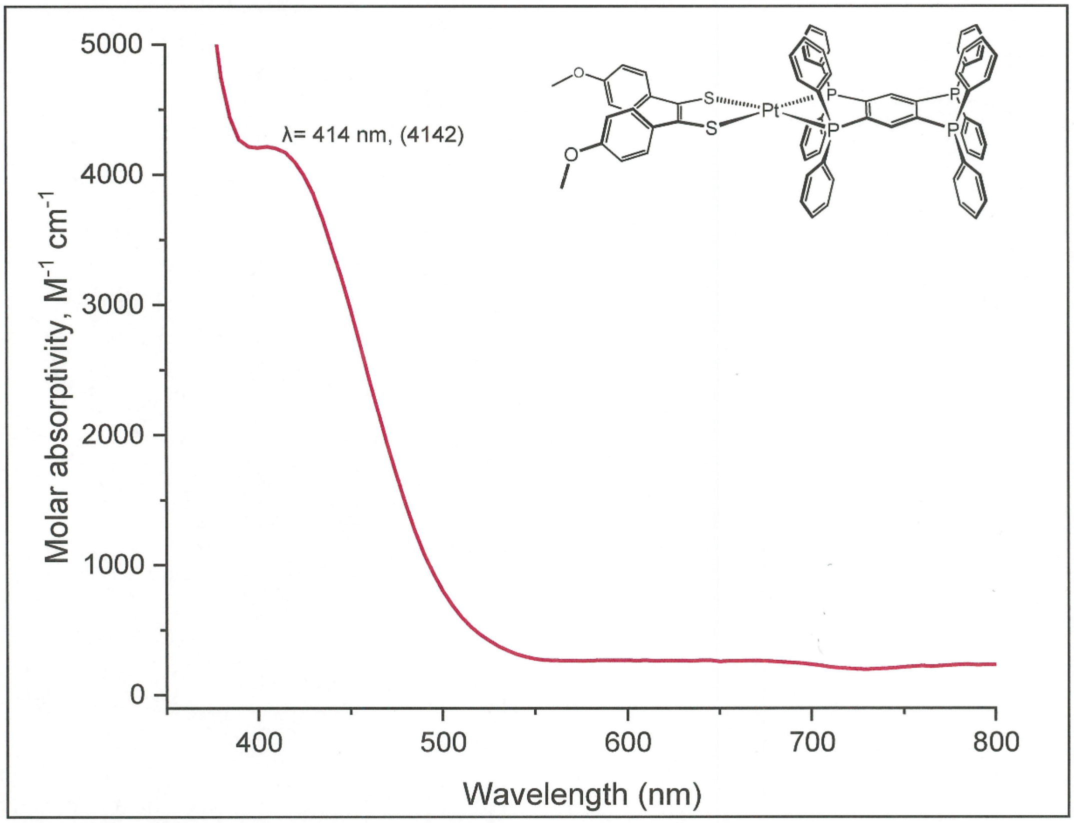

Figure S92. UV-vis spectrum (CH_2Cl_2) of [((MeO-*p*-C_6H_4)$_2C_2S_2$)Pt(tpbz)].

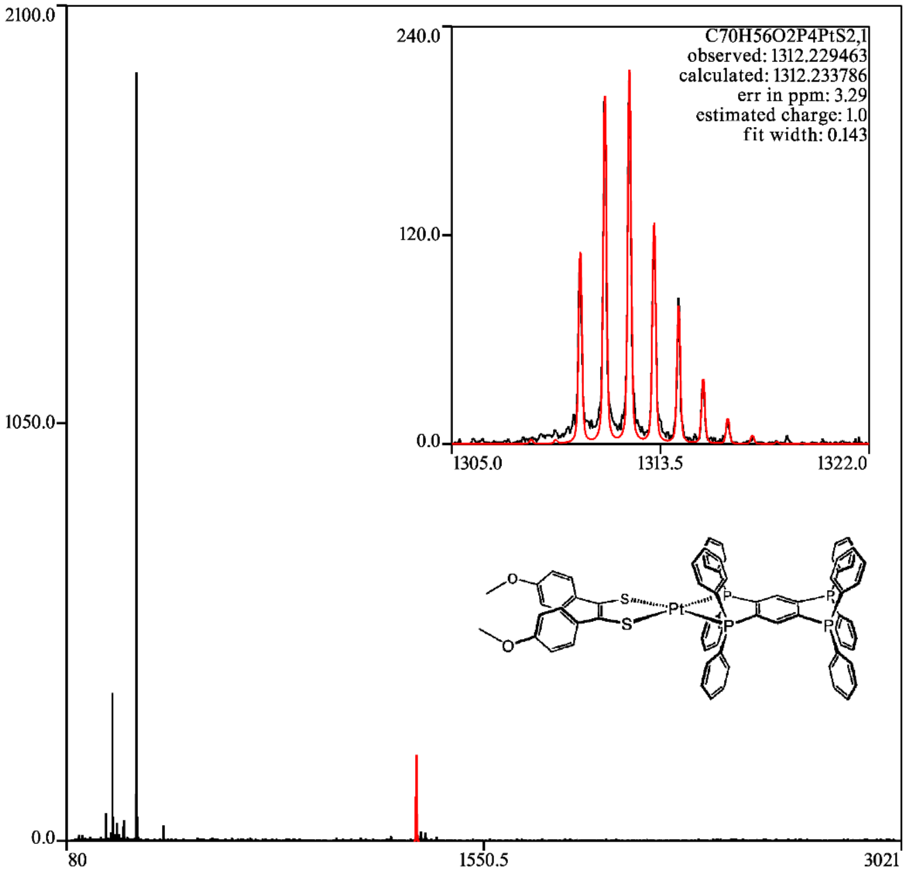

Figure S93. ESI mass spectrum (positive ion mode) of [((MeO-*p*-C_6H_4)$_2C_2S_2$)Pt(tpbz)].

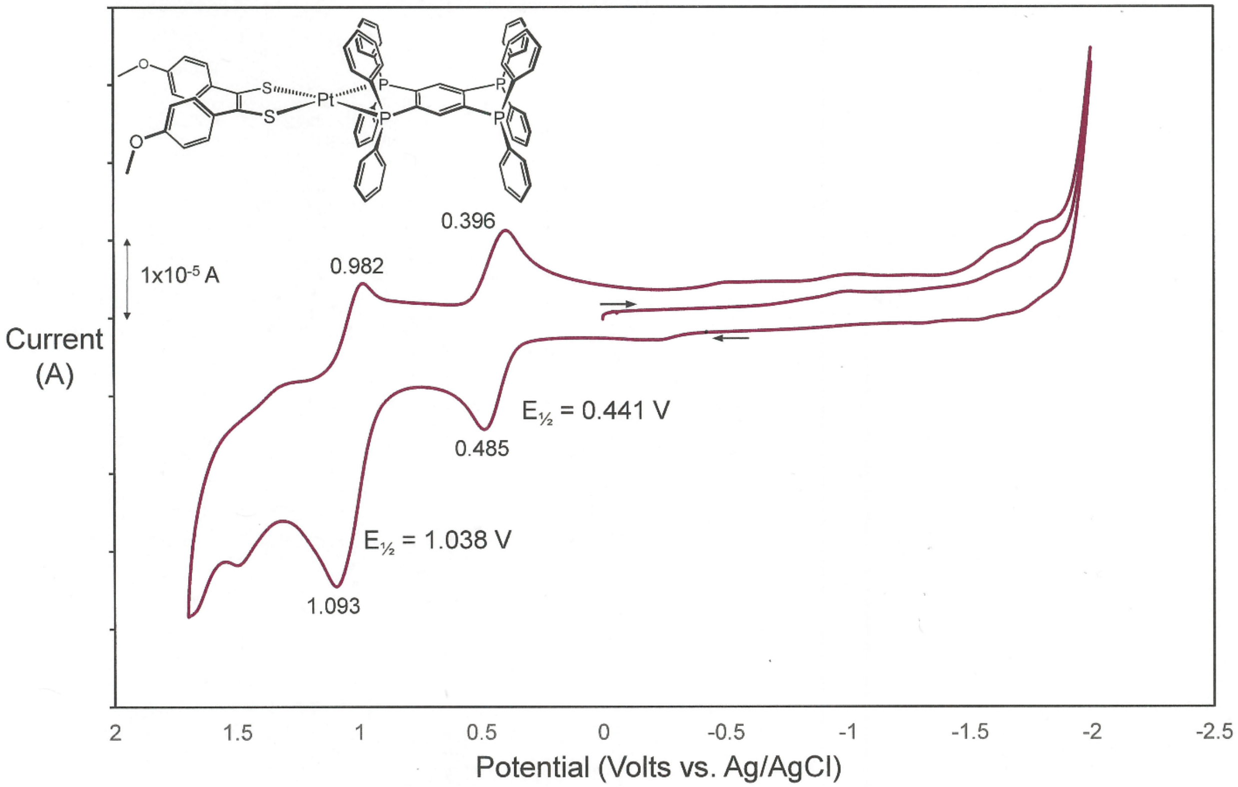

Figure S94. Cyclic voltammogram of [((CH$_3$O-*p*-C$_6$H$_4$)$_2$C$_2$S$_2$)Pt(tpbz)] in CH$_2$Cl$_2$ with [^{n}Bu$_4$N][PF$_6$] supporting electrolyte, glassy carbon working electrode, Pt wire counter electrode and AgCl/Ag reference electrode. The scan rate was 100 mV/s.

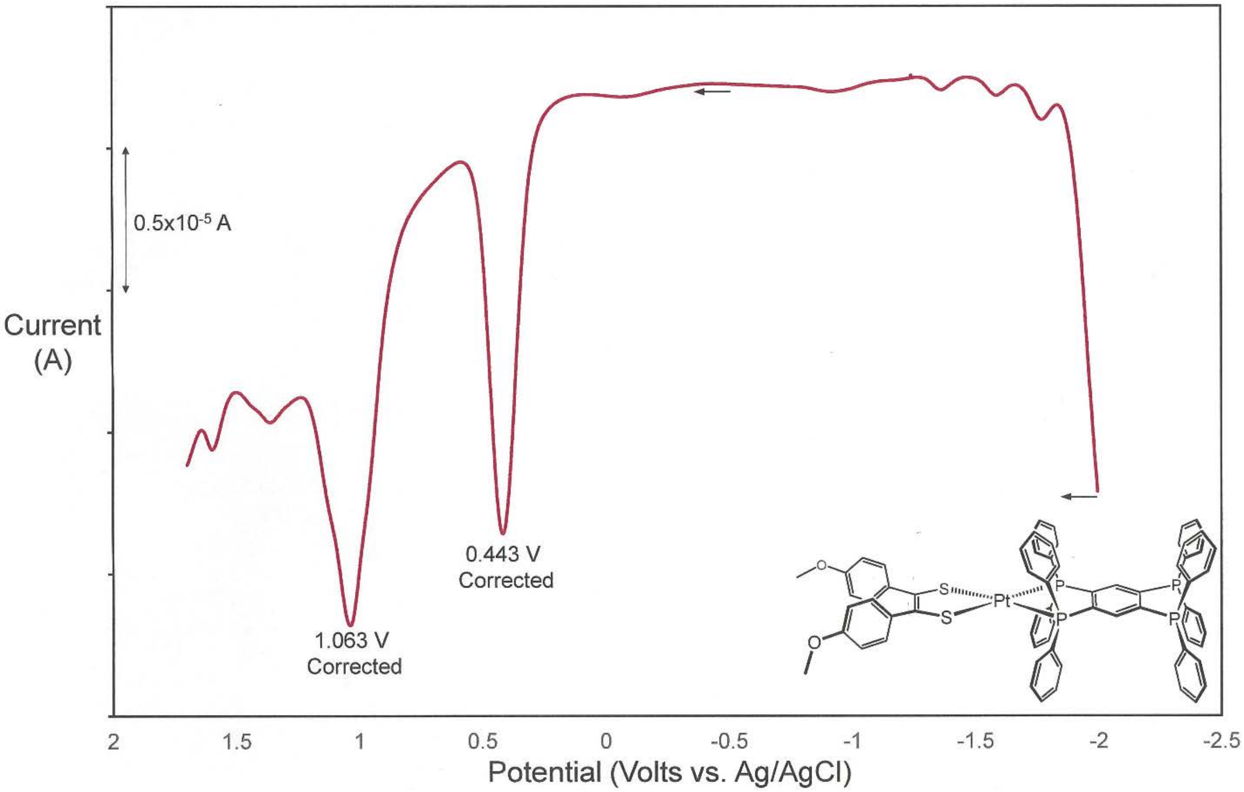

Figure S95. Differential pulse voltammogram, moving in the direction of positive potential, of [((CH$_3$O-*p*-C$_6$H$_4$)$_2$C$_2$S$_2$)Pt(tpbz)] in CH$_2$Cl$_2$ with [^{n}Bu$_4$N][PF$_6$] supporting electrolyte, glassy carbon working electrode, Pt wire counter electrode and AgCl/Ag reference electrode. The pulse amplitude was 50 mV.

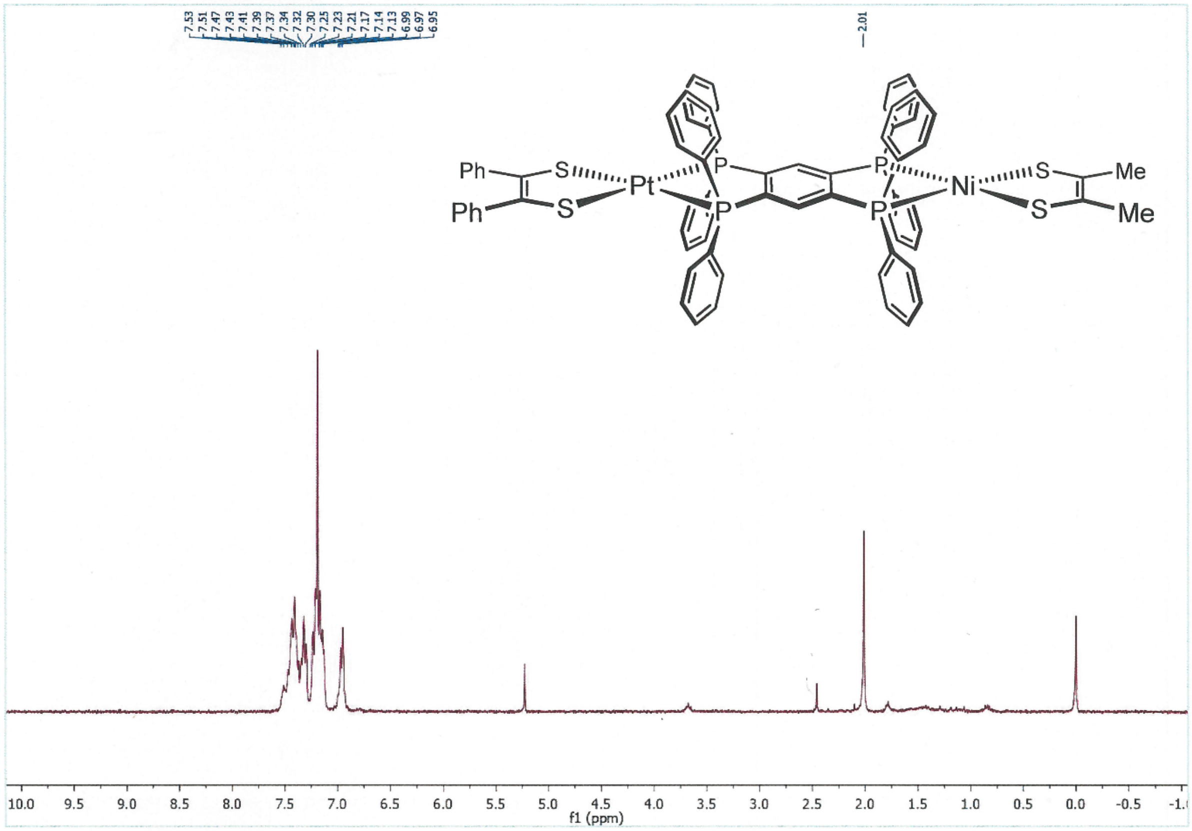

Figure S96. ^{1}H NMR spectrum of [(pdt)Pt(tpbz)Ni(mdt)] in $CDCl_3$.

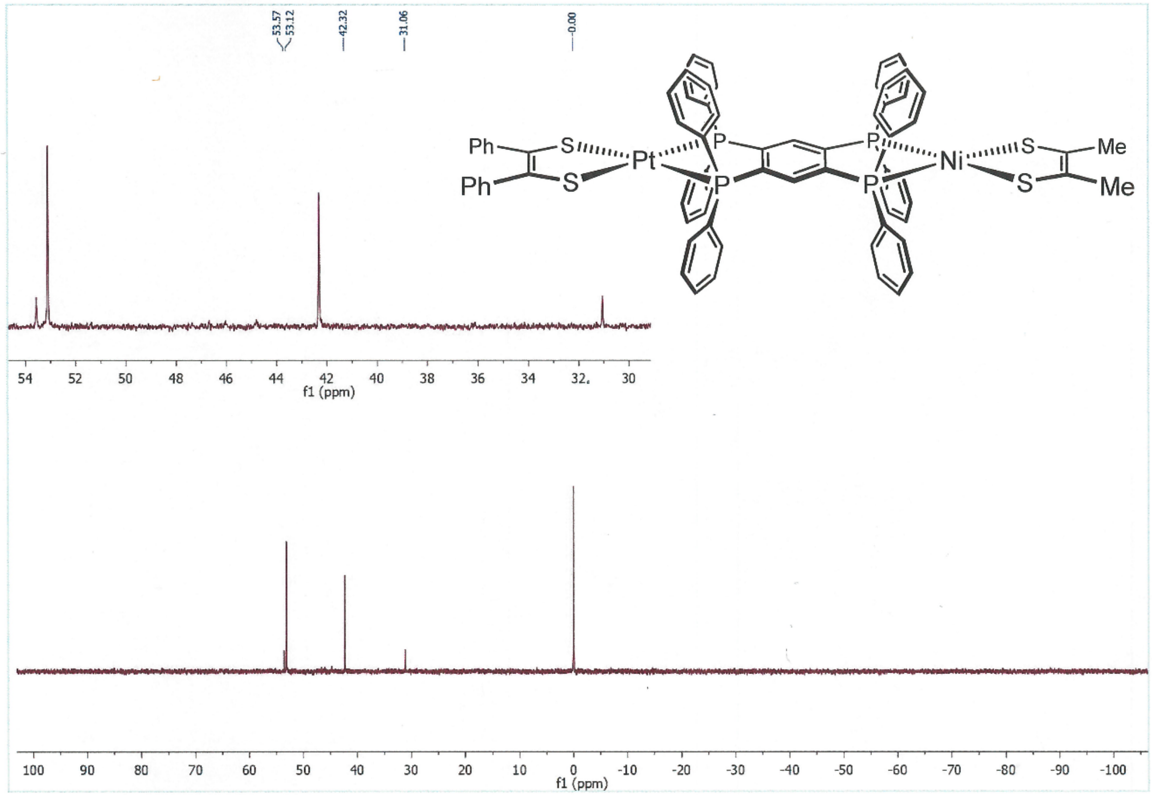

Figure S97. ^{31}P NMR spectrum of [(pdt)Pt(tpbz)Ni(mdt)] in $CDCl_3$.

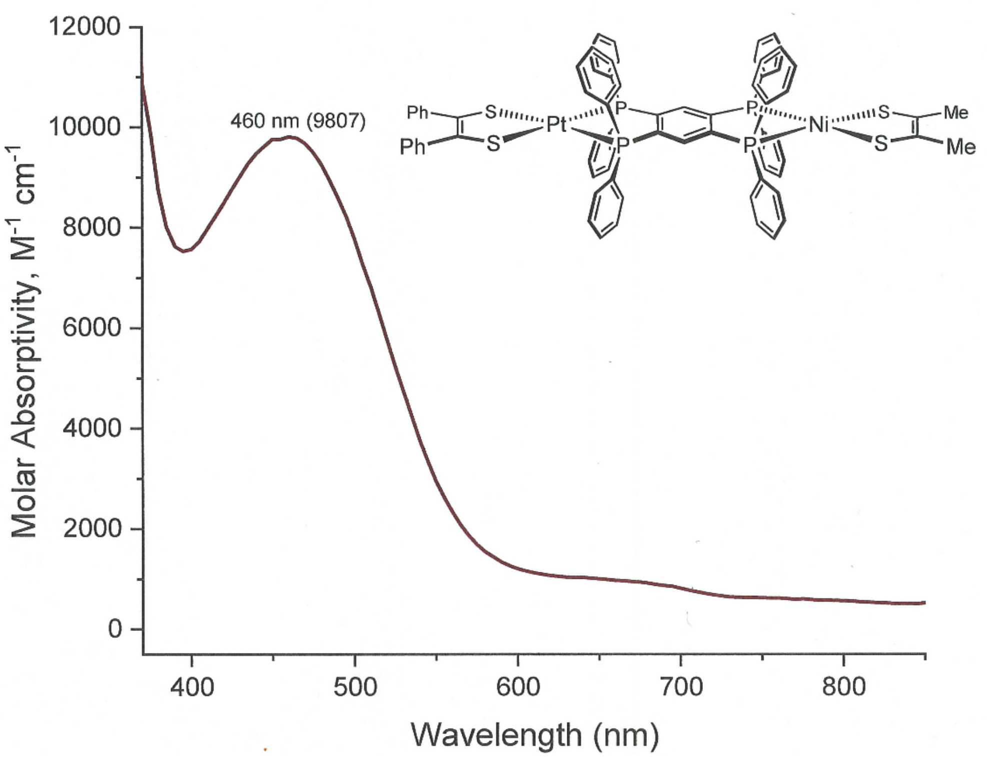

Figure S98. UV-vis spectrum in CH_2Cl_2 of [($Ph_2C_2S_2$)Pt(tpbz)Ni($S_2C_2Me_2$)].

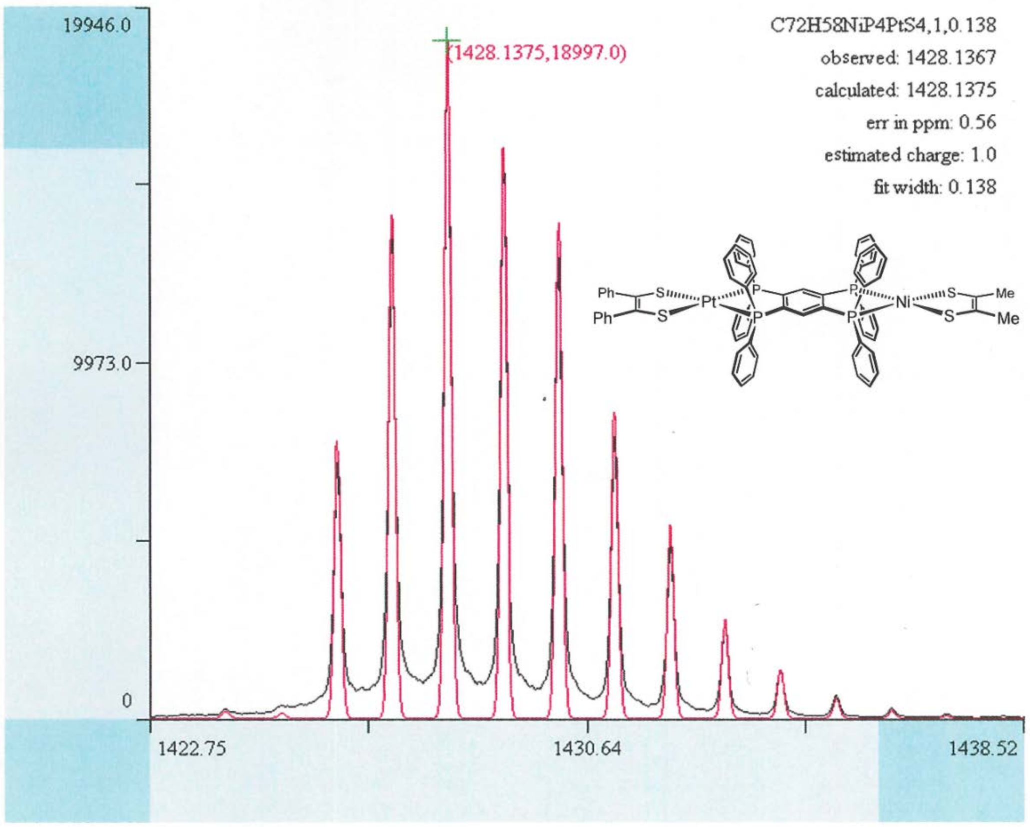

Figure S99. Mass spectrum (ESI, positive ion mode) of [($Ph_2C_2S_2$)Pt(tpbz)Ni($S_2C_2Me_2$)].

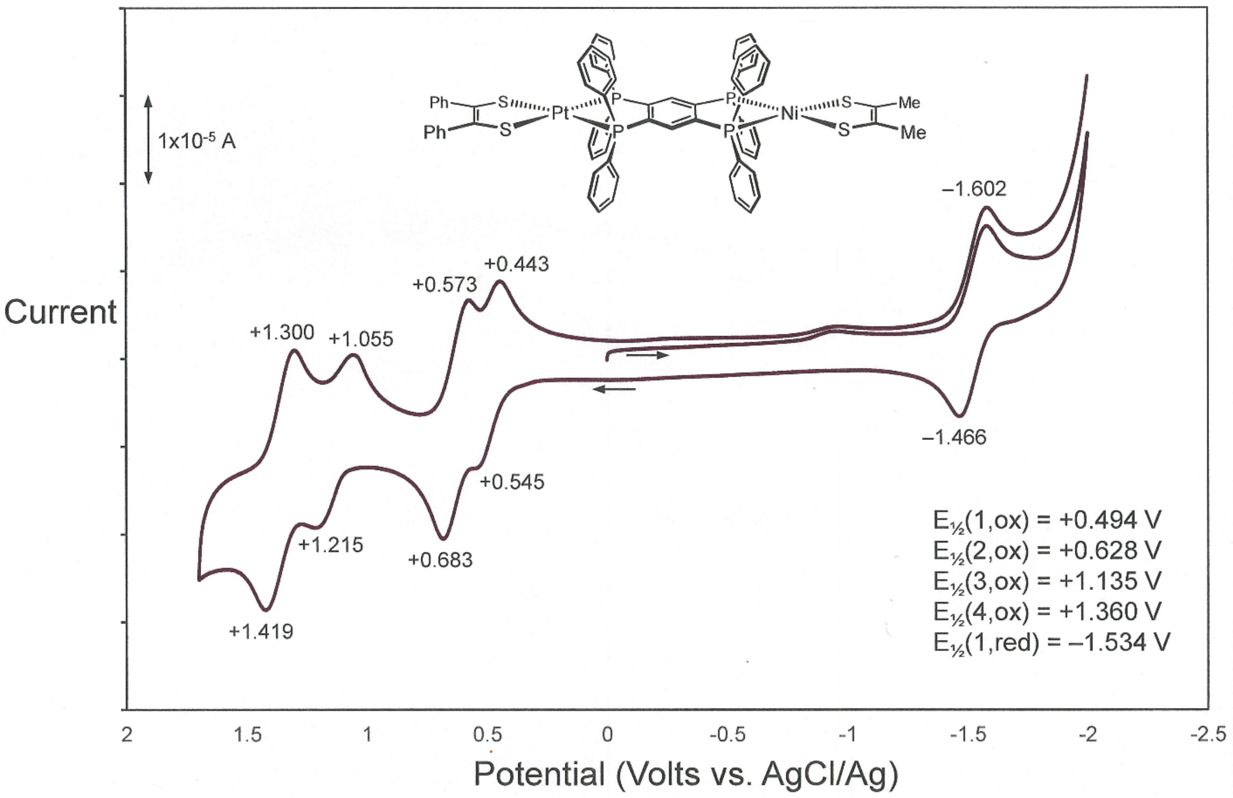

Figure S100. Cyclic voltammogram of [($Ph_2C_2S_2$)Pt(tpbz)Ni($S_2C_2Me_2$)] in CH_2Cl_2 with [^{n}Bu_4N][PF_6] supporting electrolyte, glassy carbon working electrode, Pt wire counter electrode and AgCl/Ag reference electrode. The scan rate was 100 mV/s.

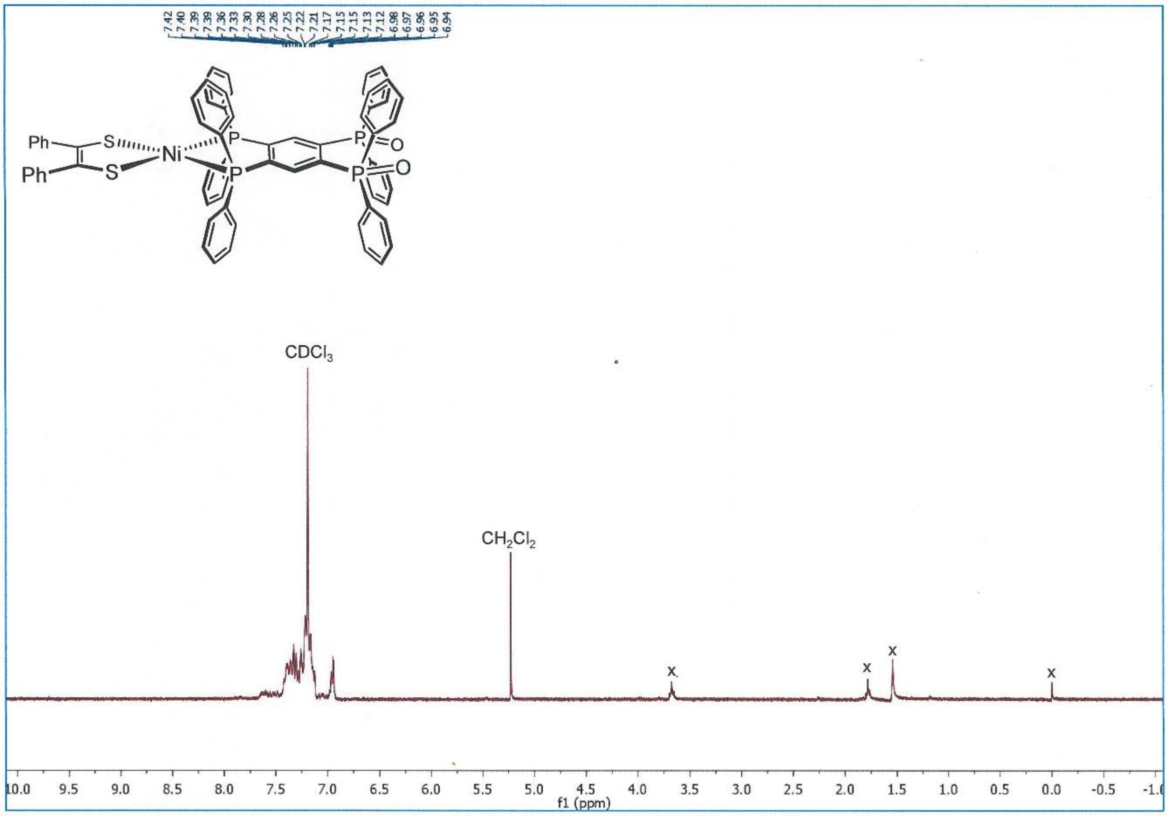

Figure S101. ^{1}H NMR spectrum ($CDCl_3$) of [($Ph_2C_2S_2$)Ni(tpbzO_2)].

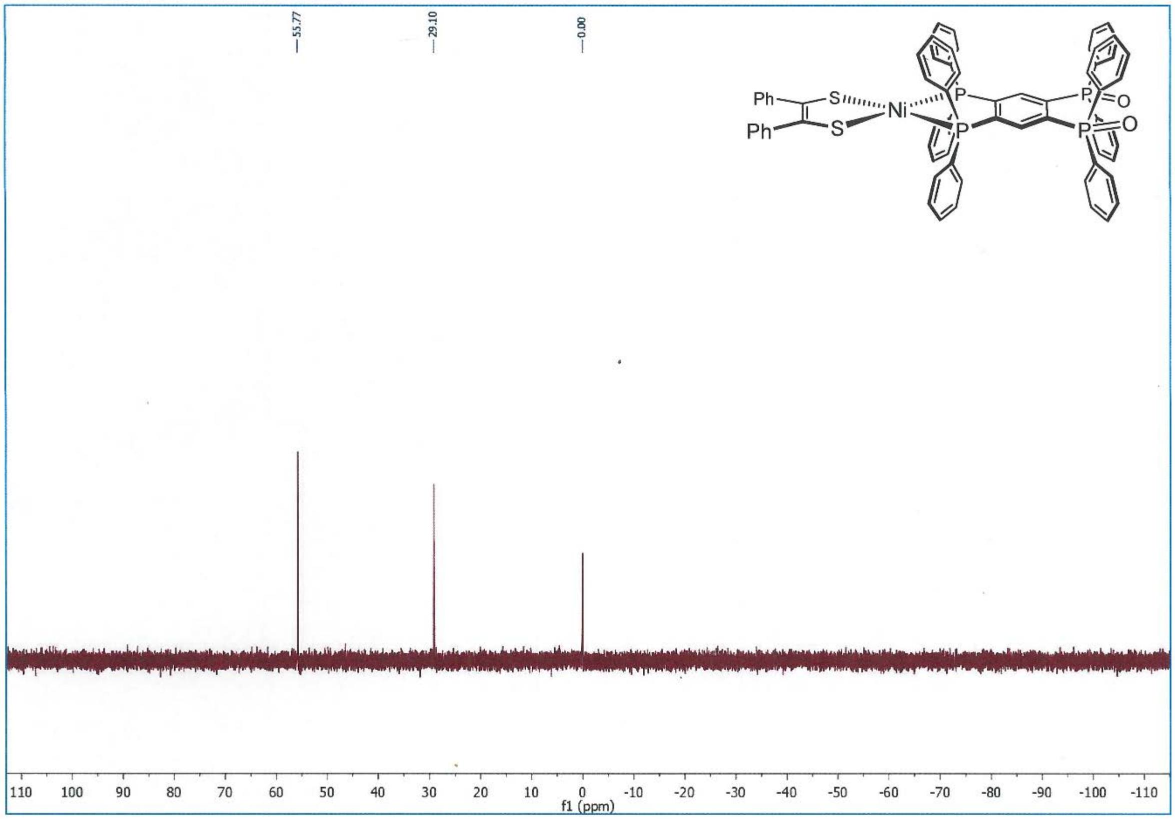

Figure S102. ^{31}P NMR spectrum ($CDCl_3$) of [($Ph_2C_2S_2$)Ni(tpbzO_2)].

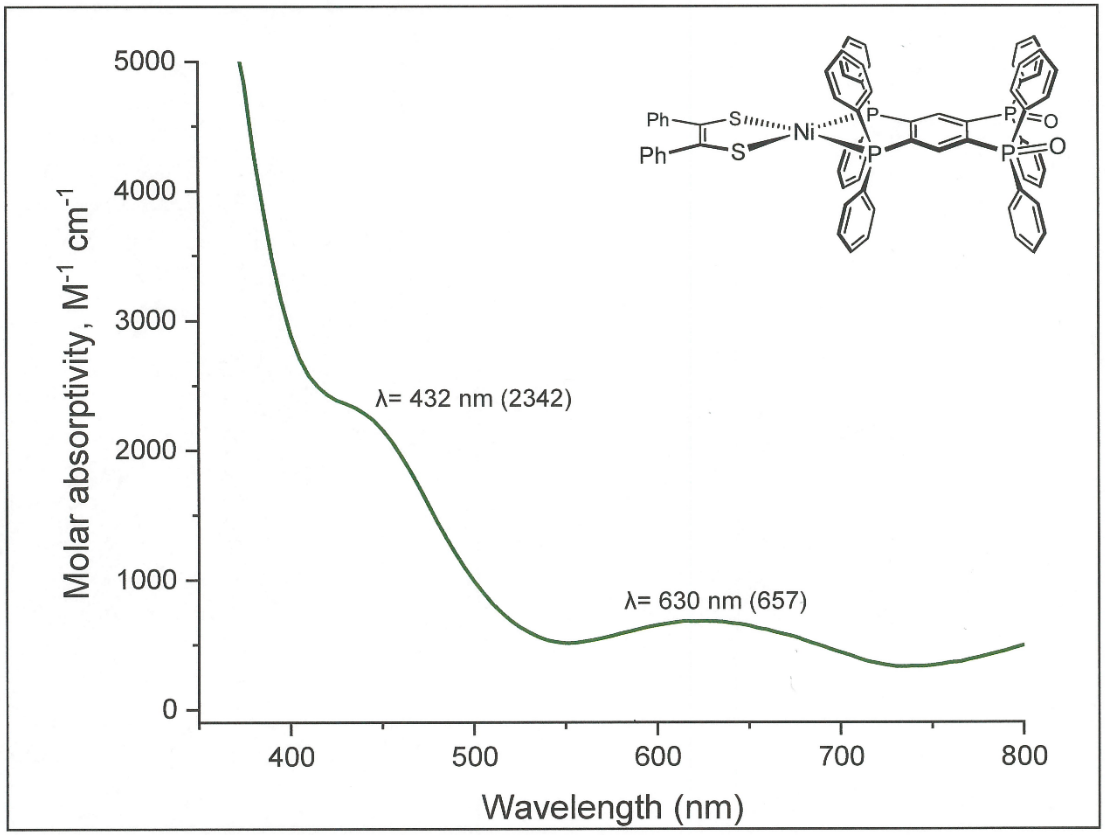

Figure S103. UV-vis spectrum (CH_2Cl_2) of [($Ph_2C_2S_2$)Ni(tpbzO_2)].

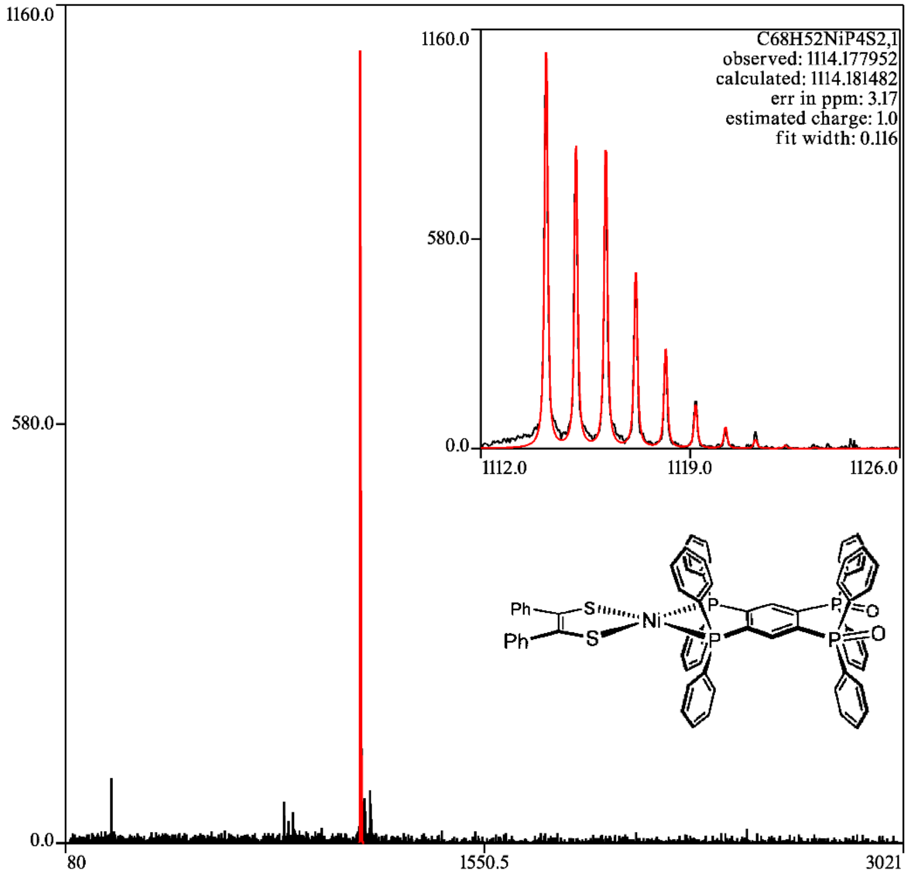

Figure S104. Mass spectrum (ESI, positive ion mode) of [($Ph_2C_2S_2$)Ni(tpbzO_2)].

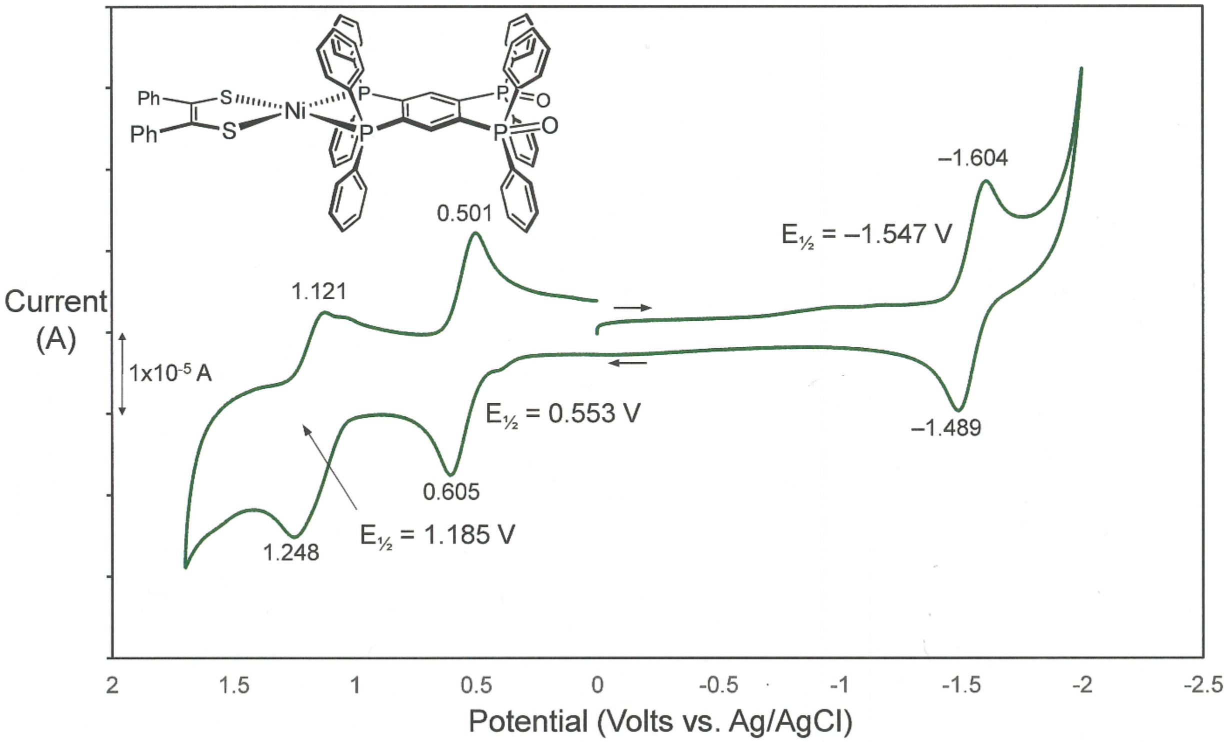

Figure S105. Cyclic voltammogram of [($Ph_2C_2S_2$)Ni(tpbzO_2)] in CH_2Cl_2 with [^{n}Bu_4N][PF_6] supporting electrolyte, glassy carbon working electrode, Pt wire counter electrode and AgCl/Ag reference electrode. The scan rate was 100 mV/s.

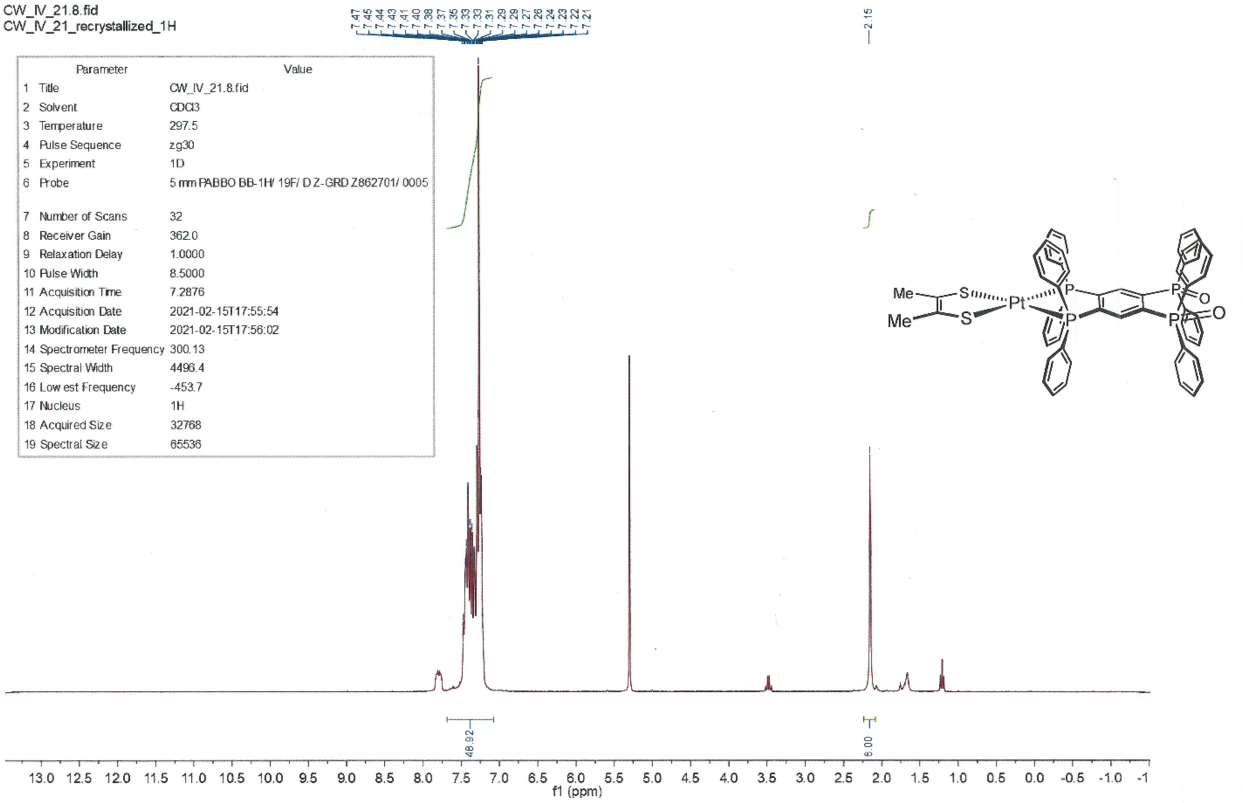

Figure S106. ^{1}H NMR spectrum ($CDCl_3$) of [$((CH_3)_2C_2S_2)Pt(tpbzO_2)$].

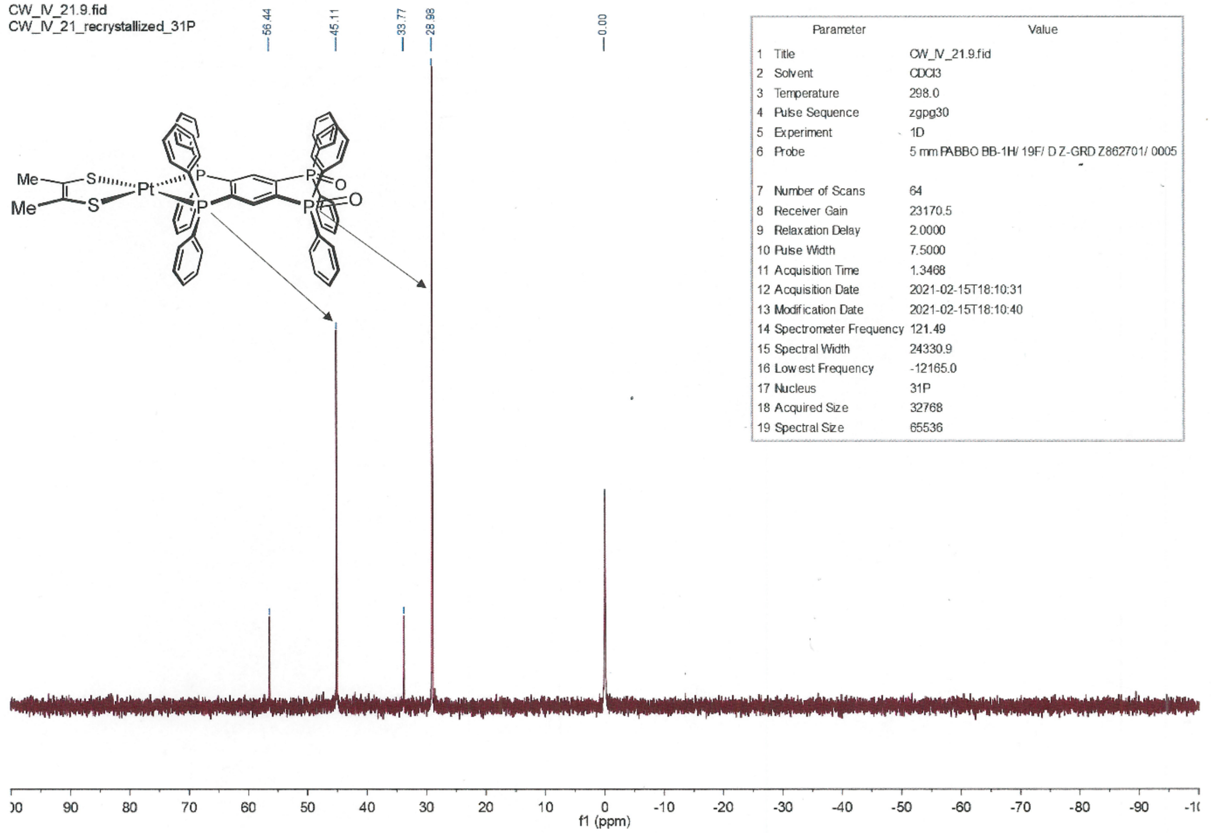

Figure S107. ^{31}P NMR spectrum ($CDCl_3$) of [$((CH_3)_2C_2S_2)Pt(tpbzO_2)$].

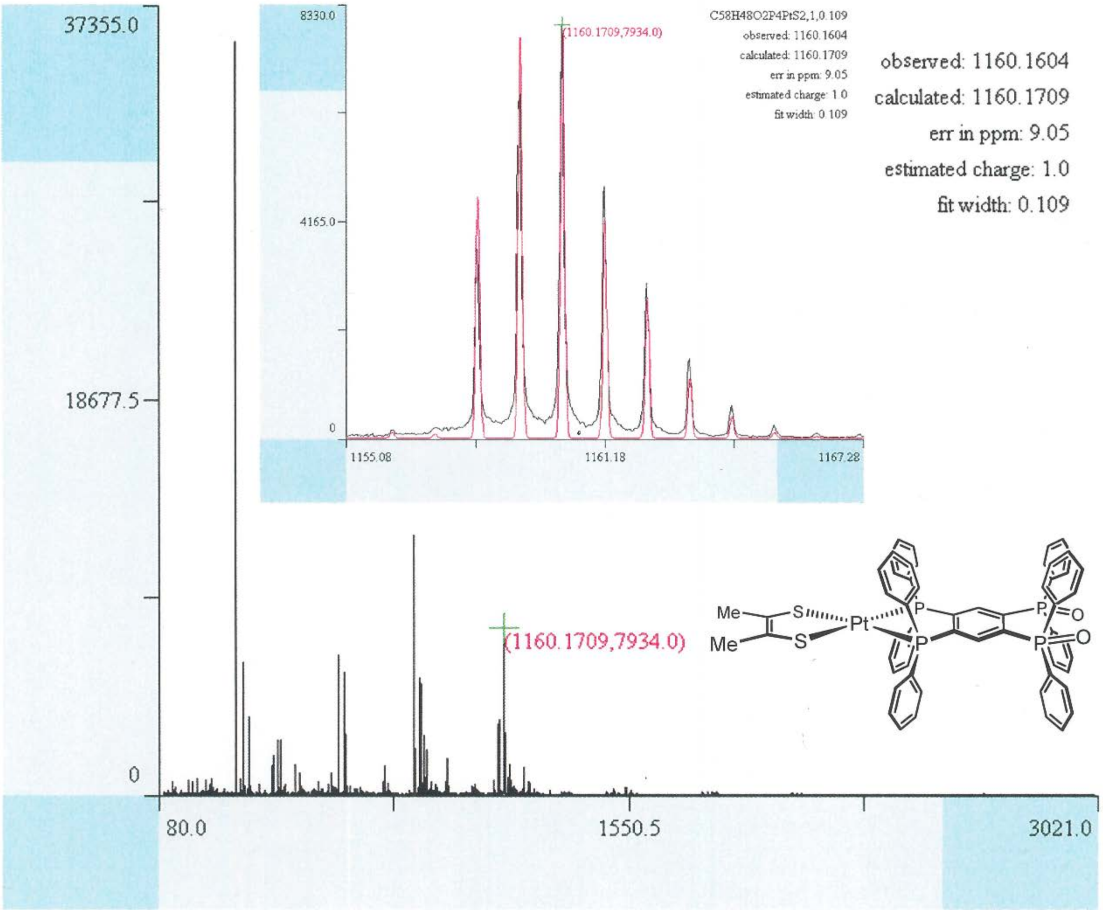

Figure S108. Mass spectrum (ESI, positive ion mode) of [($Me_2C_2S_2$)Pt(tpbzO_2)].

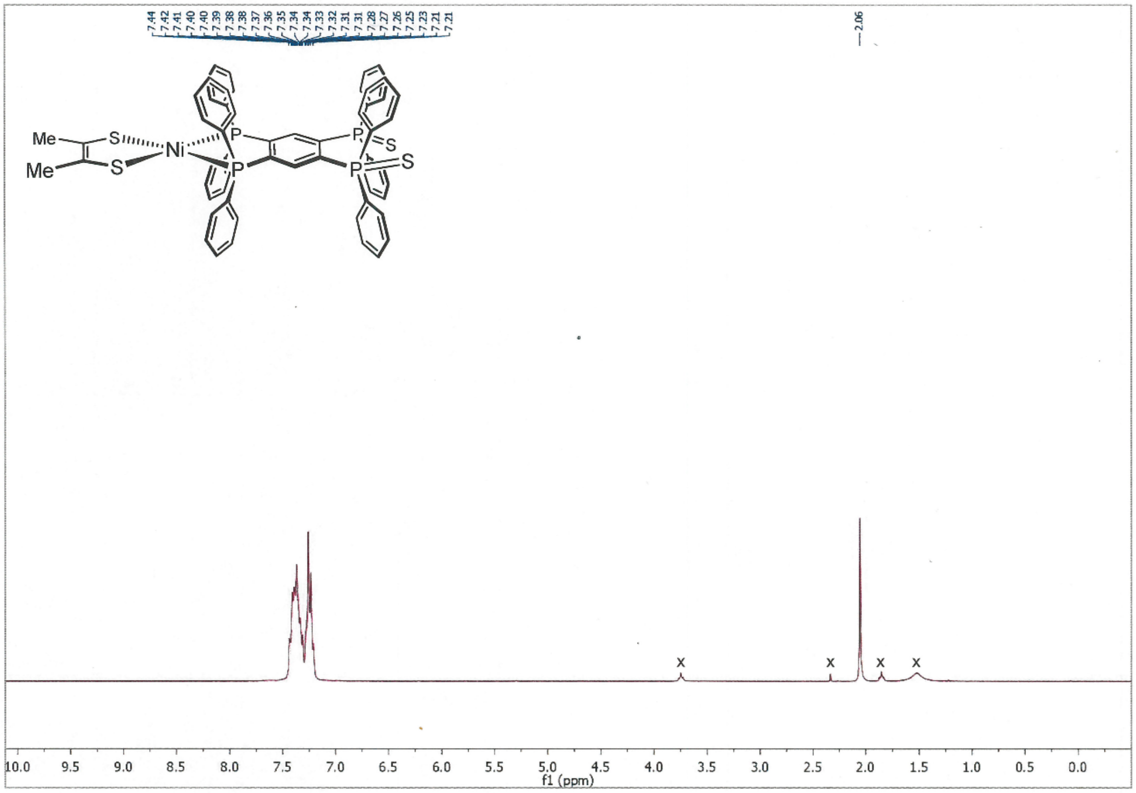

Figure S109. ^{1}H NMR spectrum ($CDCl_3$) of [(($CH_3)_2C_2S_2$)Ni(tpbzS_2)].

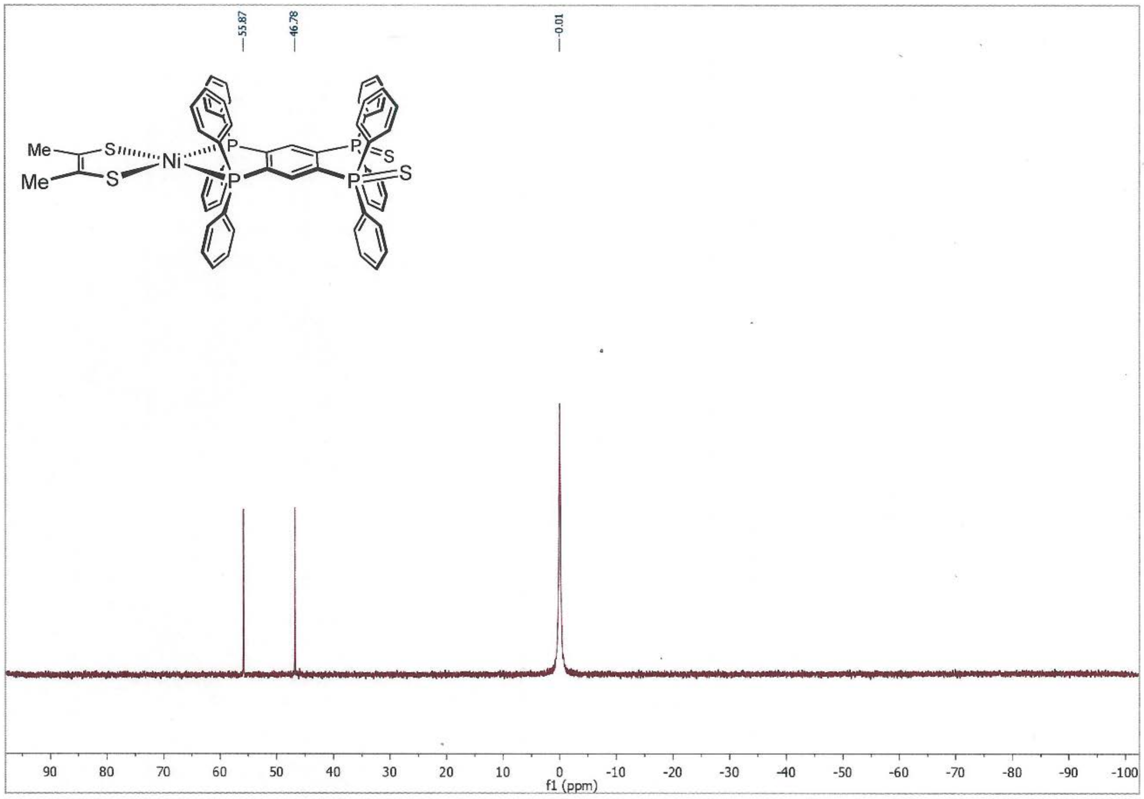

Figure S110. ^{31}P NMR spectrum ($CDCl_3$) of [(($CH_3)_2C_2S_2$)Ni(tpbzS_2)].

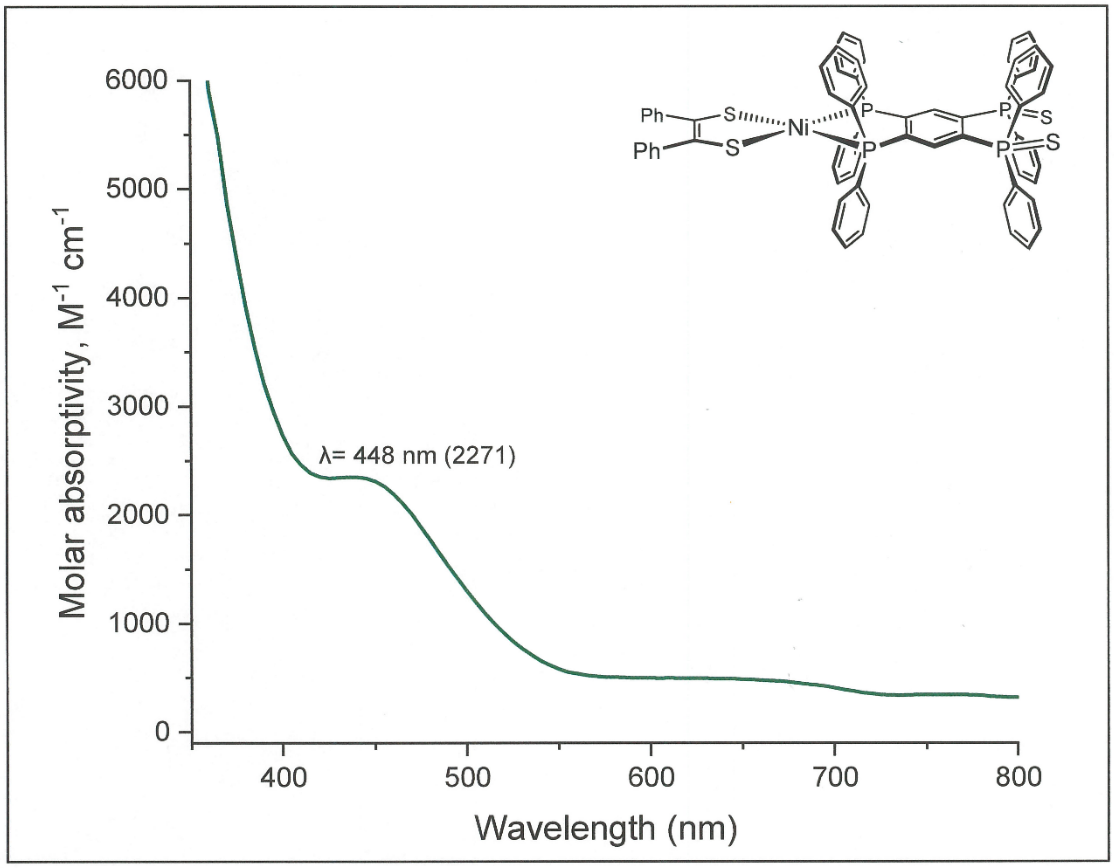

Figure S111. UV-vis spectrum (CH_2Cl_2) of [($Me_2C_2S_2$)Ni(tpbzS_2)].

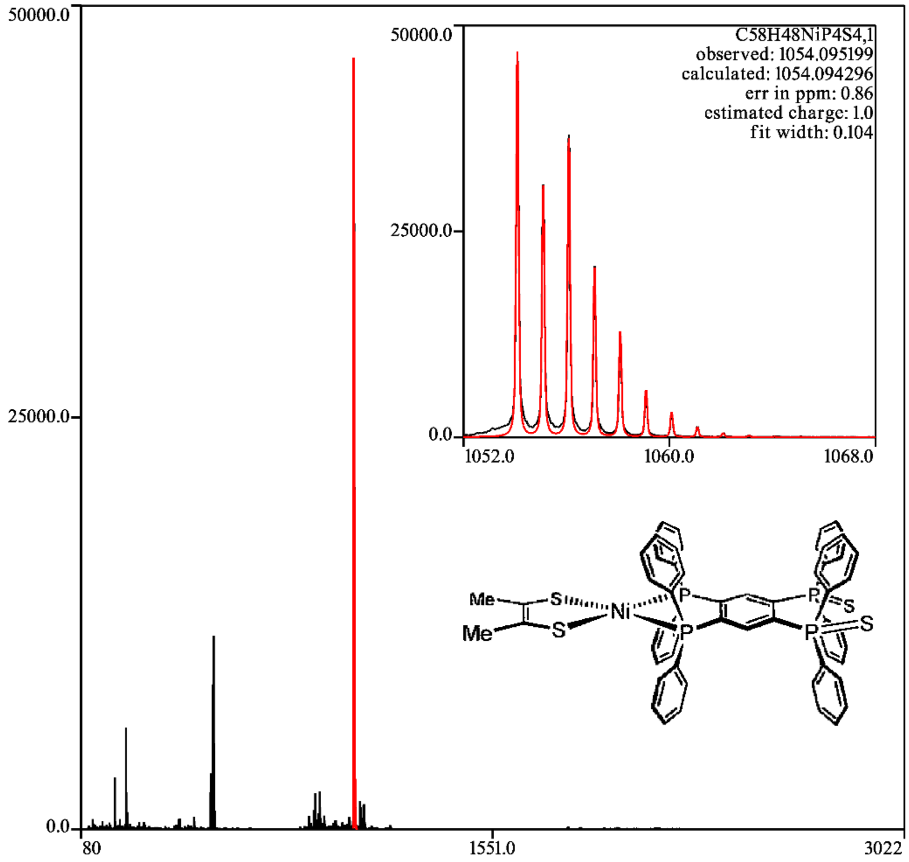

Figure S112. Mass spectrum (ESI, positive ion mode) of [($Me_2C_2S_2$)Ni(tpbzS_2)].

Report Number: 126784 GALBRAITH LABORATORIES, INC. Report Date: 2020-11-10

Laboratory Report

Report prepared for:
James P Donahue
Tulane Univ
Dept of Chem
Stern Hall, Room 2015
6400 Freret St
New Orleans, LA 70118
Phone: 504-862-3562
Email: donahue@tulane.edu

Report prepared by:
Debbie S Robertson

Purchase Order:
Visa,Bryant, 10/29/20

For further assistance, contact:
Debbie S Robertson
Report Production Coordinator
PO Box 51610
Knoxville, TN 37950 -1610
(865) 546-1335
debbierobertson@galbraith.com

Anal. Calcd for [$(Me_2C_2S_2)$Ni(tpbzS_2)], [$C_{58}H_{48}NiP_4S_4$]: C, 65.98; H, 4.58; P, 11.73.

Sample: JPD191
Lab ID: 2020-M-2234 **Received:** 2020-10-29

Analysis	Method	Result	Basis	Sample Amount Used	Date (Time)
C : Carbon					
	GLI Procedure ME-14	65.46 %	As Received	1.523 mg	2020-11-05
H : Hydrogen					
	GLI Procedure ME-14	4.44 %	As Received	1.523 mg	2020-11-05
P : Phosphorus					
	GLI Procedure ME-70	10.7 %	As Received	6.814 mg	2020-11-09

For all samples on this report:

1. There was insufficient sample to perform duplicates on elements that did not match the theory range.

Signatures:

Created By: Debbie.S.Robertson 2020-11-10T20:28:51.25-05:00
Published By: Debbie.S.Robertson 2020-11-10T20:29:14.9-05:00

- Physical signatures are on file.
- "Published By" signature indicates authorized release of data.

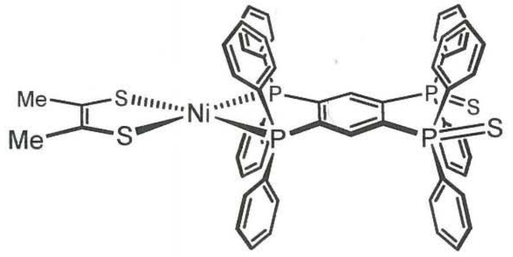

Copyright 2020 Galbraith Laboratories, Inc.
Reported results are only applicable to the item tested.
This report shall not be reproduced, except in full, without the written approval of the laboratory.

Page 1 of 1

Figure S113. Elemental analysis of [$(Me_2C_2S_2)$Ni(tpbzS_2)] from Galbraith Laboratories, Inc. The form is reproduced with the permission of Galbraith Laboratories.

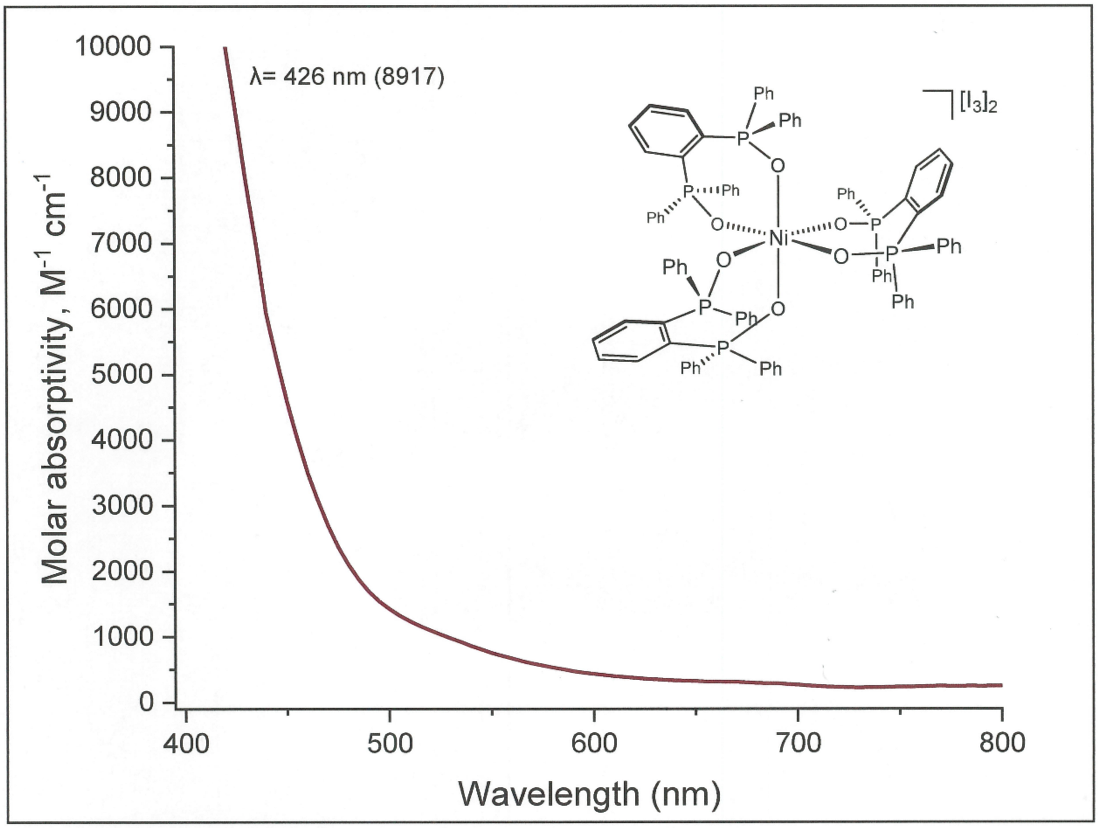

Figure S114. UV-vis spectrum (CH_2Cl_2) of [Ni(dppbO$_2$)$_3$][I$_3$]$_2$.

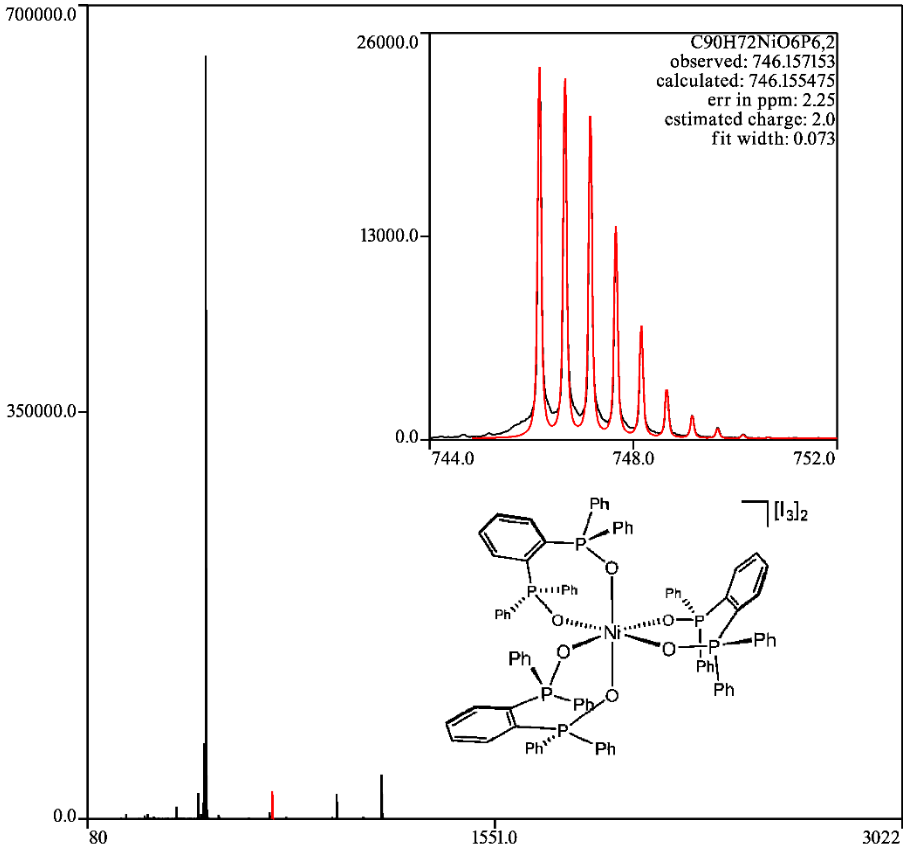

Figure S115. Mass spectrum (ESI, positive ion mode) of [Ni(dppbO$_2$)$_3$]$^{2+}$.

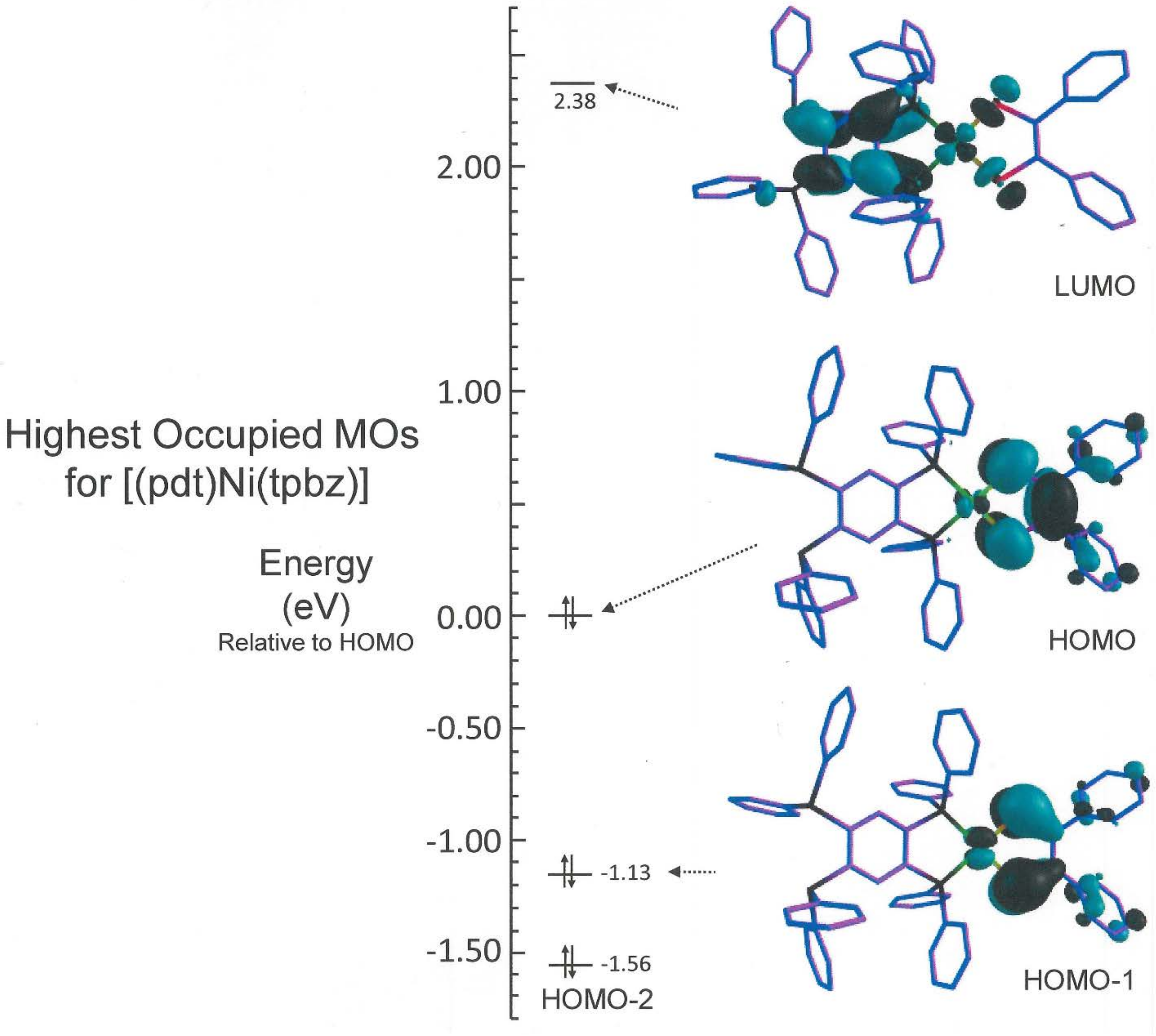

Figure S116. Molecular orbital energy level diagram illustrating the frontier MOs for [(pdt)Ni(tpbz)]. Images are presented at the 0.03 contour level.

Table S6. Atomic Coordinates for Optimized [(pdt)Pt(tpbz)Ni(mdt)].

Center Number	Atomic Number	Atomic Type	Coordinates (Angstroms) X	Y	Z
1	78	0	3.232403	0.004368	0.196555
2	28	0	-5.639882	-0.002076	0.084196
3	16	0	4.890738	-1.620118	-0.202738
4	16	0	4.901464	1.624287	-0.181603
5	16	0	-7.143396	1.555500	0.486837
6	16	0	-7.150263	-1.555337	0.477940
7	15	0	1.573089	-1.566811	0.622692
8	15	0	1.570967	1.575478	0.617154
9	15	0	-4.070032	1.536363	-0.401172
10	15	0	-4.067756	-1.541147	-0.392166
11	6	0	7.589531	-1.530392	-0.643226
12	6	0	7.613052	-2.581740	-1.576699
13	1	0	6.736779	-2.755777	-2.202317
14	6	0	8.740351	-3.390254	-1.716559
15	1	0	8.738215	-4.196285	-2.453868
16	6	0	9.867246	-3.171365	-0.922964
17	1	0	10.749742	-3.805367	-1.031925
18	6	0	9.854234	-2.137340	0.014923
19	1	0	10.727037	-1.961406	0.647712
20	6	0	8.728000	-1.329089	0.156726
21	1	0	8.721201	-0.529688	0.897626
22	6	0	6.379189	-0.679820	-0.475239
23	6	0	6.380549	0.677736	-0.483504
24	6	0	7.558513	1.522630	-0.823215
25	6	0	8.282393	1.305445	-2.008845
26	1	0	7.979572	0.497278	-2.674411
27	6	0	9.371203	2.109202	-2.340232
28	1	0	9.918172	1.920983	-3.266817
29	6	0	9.757796	3.154814	-1.499761
30	1	0	10.610187	3.785495	-1.761183
31	6	0	9.042347	3.389552	-0.324866
32	1	0	9.335151	4.204687	0.340786
33	6	0	7.953081	2.585330	0.008500
34	1	0	7.400339	2.771836	0.929972
35	6	0	1.462487	-3.055592	-0.451374
36	6	0	1.840367	-2.930034	-1.797820
37	1	0	2.242056	-1.982672	-2.159040
38	6	0	1.729096	-4.020032	-2.660205
39	1	0	2.031633	-3.915146	-3.703623
40	6	0	1.254407	-5.244854	-2.186590
41	1	0	1.177574	-6.099533	-2.861434
42	6	0	0.891713	-5.378925	-0.845951
43	1	0	0.531361	-6.337461	-0.467867
44	6	0	0.994624	-4.290390	0.021539
45	1	0	0.715496	-4.408027	1.068610
46	6	0	1.521517	-2.192831	2.354311

Table S6 Continued. Atomic Coordinates for Optimized [(pdt)Pt(tpbz)Ni(mdt)].

Center Number	Atomic Number	Atomic Type	Coordinates (Angstroms) X	Y	Z
47	6	0	0.327144	-2.444176	3.046910
48	1	0	-0.638841	-2.255163	2.578958
49	6	0	0.363458	-2.936748	4.352051
50	1	0	-0.572273	-3.126230	4.881328
51	6	0	1.587087	-3.184347	4.975230
52	1	0	1.612090	-3.567738	5.997120
53	6	0	2.778064	-2.934136	4.291597
54	1	0	3.738711	-3.120160	4.774991
55	6	0	2.750515	-2.434752	2.989987
56	1	0	3.682955	-2.224317	2.463170
57	6	0	1.461018	3.066379	-0.454148
58	6	0	0.967355	4.293034	0.014396
59	1	0	0.665917	4.403062	1.056137
60	6	0	0.868332	5.384017	-0.850271
61	1	0	0.487620	6.336035	-0.475642
62	6	0	1.261605	5.261012	-2.183425
63	1	0	1.188776	6.118022	-2.855746
64	6	0	1.762244	4.044865	-2.652209
65	1	0	2.089608	3.948992	-3.688893
66	6	0	1.868863	2.952121	-1.792649
67	1	0	2.291309	2.012166	-2.149259
68	6	0	1.515533	2.199620	2.349889
69	6	0	2.742844	2.437777	2.990026
70	1	0	3.676192	2.224820	2.465744
71	6	0	2.767471	2.935210	4.292411
72	1	0	3.726852	3.117784	4.779707
73	6	0	1.574965	3.187658	4.972541
74	1	0	1.597618	3.569596	5.995022
75	6	0	0.352857	2.943755	4.345049
76	1	0	-0.584132	3.134651	4.871576
77	6	0	0.319677	2.452723	3.039213
78	1	0	-0.645249	2.265976	2.568155
79	6	0	-0.046184	-0.700864	0.352107
80	6	0	-0.046984	0.706609	0.347664
81	6	0	-1.240375	1.395068	0.100881
82	1	0	-1.241754	2.485251	0.093888
83	6	0	-2.433334	0.703120	-0.136555
84	6	0	-2.432530	-0.703775	-0.131810
85	6	0	-1.238622	-1.392509	0.109998
86	1	0	-1.238470	-2.482730	0.109920
87	6	0	-4.065650	2.059426	-2.168518
88	6	0	-2.891673	2.315214	-2.894297
89	1	0	-1.913060	2.199338	-2.427760
90	6	0	-2.965681	2.716662	-4.228513
91	1	0	-2.046370	2.911451	-4.784071
92	6	0	-4.206979	2.865913	-4.848987

Table S6 Continued. Atomic Coordinates for Optimized [(pdt)Pt(tpbz)Ni(mdt)].

Center Number	Atomic Number	Atomic Type	Coordinates (Angstroms) X	Y	Z
93	1	0	-4.260963	3.178150	-5.893757
94	6	0	-5.377055	2.608666	-4.133242
95	1	0	-6.350557	2.717704	-4.614634
96	6	0	-5.311553	2.201460	-2.800883
97	1	0	-6.225209	1.989096	-2.242424
98	6	0	-3.924515	3.082865	0.585636
99	6	0	-4.235269	3.021214	1.953733
100	1	0	-4.614652	2.092210	2.380551
101	6	0	-4.089408	4.151605	2.756863
102	1	0	-4.340031	4.095497	3.817695
103	6	0	-3.648682	5.355087	2.202179
104	1	0	-3.544642	6.241442	2.830948
105	6	0	-3.355572	5.426521	0.839997
106	1	0	-3.022777	6.367870	0.398633
107	6	0	-3.492556	4.296413	0.031784
108	1	0	-3.266237	4.364799	-1.032262
109	6	0	-4.059792	-2.076727	-2.155911
110	6	0	-2.884167	-2.333540	-2.878643
111	1	0	-1.906403	-2.210458	-2.412249
112	6	0	-2.955274	-2.745436	-4.209828
113	1	0	-2.034584	-2.940935	-4.762856
114	6	0	-4.195304	-2.904253	-4.830409
115	1	0	-4.247050	-3.224655	-5.872819
116	6	0	-5.367044	-2.646014	-4.117742
117	1	0	-6.339656	-2.762440	-4.599210
118	6	0	-5.304474	-2.228332	-2.788491
119	1	0	-6.219633	-2.015128	-2.232777
120	6	0	-3.921805	-3.080653	0.605440
121	6	0	-4.241957	-3.011561	1.970971
122	1	0	-4.628616	-2.081576	2.389038
123	6	0	-4.096142	-4.136013	2.782427
124	1	0	-4.354026	-4.074168	3.841194
125	6	0	-3.646132	-5.340964	2.238553
126	1	0	-3.542120	-6.222720	2.873759
127	6	0	-3.343630	-5.419901	0.878831
128	1	0	-3.003428	-6.362521	0.445906
129	6	0	-3.480522	-4.295770	0.062366
130	1	0	-3.247017	-4.370129	-0.999748
131	6	0	-9.844807	1.579070	1.035042
132	1	0	-10.762792	1.011245	1.249381
133	1	0	-10.044932	2.227085	0.162661
134	1	0	-9.654780	2.249469	1.892491
135	6	0	-8.664654	0.676346	0.781978
136	6	0	-8.667486	-0.671186	0.778674
137	6	0	-9.851316	-1.570101	1.028104
138	1	0	-9.663036	-2.245991	1.881615

Table S6 Continued. Atomic Coordinates for Optimized [(pdt)Pt(tpbz)Ni(mdt)].

Center Number	Atomic Number	Atomic Type	Coordinates (Angstroms) X	Y	Z
139	1	0	-10.055389	-2.212472	0.152471
140	1	0	-10.766534	-0.999470	1.246780

Table S7. Atomic Coordinates for Optimized [(pdt)Ni(tpbz)].

Center Number	Atomic Number	Atomic Type	Coordinates (Angstroms) X	Y	Z
1	28	0	-2.350486	0.045313	0.144352
2	16	0	-3.850573	-1.546188	-0.102497
3	15	0	-0.738110	1.613401	0.386474
4	15	0	4.854019	1.578207	-0.905455
5	6	0	-5.404174	-0.707782	-0.325439
6	6	0	-0.628609	2.305665	2.091245
7	6	0	-1.832671	2.576314	2.762263
8	1	0	-2.783228	2.356920	2.272132
9	6	0	-1.813400	3.116865	4.047671
10	1	0	-2.756005	3.323953	4.557673
11	6	0	-0.599075	3.382122	4.682404
12	1	0	-0.587137	3.798324	5.691744
13	6	0	0.600875	3.107354	4.025465
14	1	0	1.554710	3.307953	4.517546
15	6	0	0.588631	2.571920	2.736764
16	1	0	1.534796	2.360774	2.237550
17	6	0	0.876341	0.757073	0.102139
18	6	0	2.066269	1.419305	-0.227926
19	1	0	2.062330	2.505088	-0.323560
20	6	0	3.261084	0.718965	-0.445702
21	6	0	5.719196	1.676621	0.729042
22	6	0	5.104713	1.473521	1.974228
23	1	0	4.038512	1.247918	2.025705
24	6	0	5.846684	1.547894	3.155044
25	1	0	5.352465	1.384658	4.115283
26	6	0	7.211982	1.832756	3.110869
27	1	0	7.790673	1.890710	4.034865
28	6	0	7.834908	2.036928	1.878486
29	1	0	8.904171	2.253431	1.833929
30	6	0	7.096478	1.950248	0.698852
31	1	0	7.596386	2.091066	-0.262110
32	6	0	4.307519	3.326535	-1.181229
33	6	0	4.342448	4.336246	-0.206800
34	1	0	4.663267	4.102043	0.808822
35	6	0	3.979406	5.645817	-0.527136
36	1	0	4.019257	6.420461	0.241731
37	6	0	3.568595	5.965610	-1.821597
38	1	0	3.286987	6.990688	-2.070499
39	6	0	3.527299	4.970072	-2.799719
40	1	0	3.210326	5.212454	-3.815848
41	6	0	3.905507	3.666027	-2.484837
42	1	0	3.894261	2.898734	-3.262708
43	16	0	-3.912671	1.561800	-0.195916
44	15	0	-0.734974	-1.460883	0.602816
45	15	0	4.834299	-1.633991	-0.694162
46	6	0	-5.426381	0.648456	-0.396353

Table S7 Continued. Atomic Coordinates for Optimized [(pdt)Ni(tpbz)].

Center Number	Atomic Number	Atomic Type	Coordinates (Angstroms) X	Y	Z
47	6	0	-0.640668	-1.920097	2.383087
48	6	0	0.569491	-2.159483	3.053131
49	1	0	1.520769	-2.070948	2.527471
50	6	0	0.567058	-2.513401	4.402738
51	1	0	1.514524	-2.696900	4.913396
52	6	0	-0.638630	-2.631675	5.095902
53	1	0	-0.636834	-2.907156	6.152468
54	6	0	-1.844767	-2.391730	4.436404
55	1	0	-2.791258	-2.477749	4.973161
56	6	0	-1.850089	-2.032659	3.088600
57	1	0	-2.792745	-1.836446	2.574157
58	6	0	0.875413	-0.644294	0.210703
59	6	0	2.059928	-1.350459	-0.030377
60	1	0	2.049858	-2.439109	0.025660
61	6	0	3.250851	-0.696760	-0.370420
62	6	0	4.497077	-3.230766	0.182225
63	6	0	4.066801	-4.411930	-0.439407
64	1	0	3.866196	-4.422373	-1.511088
65	6	0	3.889002	-5.582018	0.303245
66	1	0	3.554451	-6.492246	-0.198736
67	6	0	4.133332	-5.589836	1.676233
68	1	0	3.994054	-6.505514	2.254252
69	6	0	4.567335	-4.420897	2.306567
70	1	0	4.772567	-4.420569	3.379178
71	6	0	4.758477	-3.256529	1.564415
72	1	0	5.122549	-2.353626	2.061354
73	6	0	4.667863	-2.071654	-2.484976
74	6	0	3.451363	-2.197988	-3.174369
75	6	0	5.864438	-2.270969	-3.193073
76	6	0	3.434821	-2.522948	-4.531500
77	6	0	5.847147	-2.604115	-4.547488
78	6	0	4.631196	-2.728828	-5.220473
79	1	0	2.505955	-2.042625	-2.653035
80	1	0	6.819915	-2.156975	-2.676473
81	1	0	2.479619	-2.615867	-5.052417
82	1	0	6.787678	-2.756875	-5.080639
83	1	0	4.615443	-2.981291	-6.282635
84	6	0	-0.663603	-3.041954	-0.330650
85	6	0	-0.232825	-4.244888	0.245751
86	6	0	-1.033983	-3.022843	-1.685425
87	6	0	-0.158023	-5.405995	-0.525785
88	6	0	-0.951218	-4.183673	-2.452428
89	6	0	-0.513052	-5.377000	-1.874382
90	1	0	0.045196	-4.280618	1.299239
91	1	0	-1.408672	-2.100011	-2.129996
92	1	0	0.177718	-6.337206	-0.065802

Table S7 Continued. Atomic Coordinates for Optimized [(pdt)Ni(tpbz)].

Center Number	Atomic Number	Atomic Type	Coordinates (Angstroms) X	Y	Z
93	1	0	-1.246616	-4.158640	-3.502932
94	1	0	-0.457387	-6.287361	-2.474554
95	6	0	-0.709965	3.064978	-0.737338
96	6	0	-1.186100	2.894810	-2.047110
97	6	0	-0.234589	4.322023	-0.336935
98	6	0	-1.166630	3.960752	-2.944714
99	6	0	-0.223375	5.387297	-1.238064
100	6	0	-0.687087	5.208159	-2.541583
101	1	0	-1.596698	1.931705	-2.351702
102	1	0	0.122387	4.475330	0.681849
103	1	0	-1.545355	3.819083	-3.958422
104	1	0	0.147775	6.361728	-0.916102
105	1	0	-0.683602	6.045221	-3.242630
106	6	0	-6.614363	1.478982	-0.722815
107	6	0	-7.421422	1.178581	-1.835142
108	6	0	-6.940343	2.612492	0.043200
109	6	0	-8.521758	1.970397	-2.156645
110	6	0	-8.041513	3.404245	-0.279967
111	6	0	-8.838961	3.086823	-1.380343
112	1	0	-7.173315	0.315520	-2.452820
113	1	0	-6.322344	2.864614	0.905639
114	1	0	-9.132620	1.717009	-3.026110
115	1	0	-8.278588	4.275739	0.334638
116	1	0	-9.700506	3.708105	-1.634392
117	6	0	-6.591731	-1.598539	-0.377544
118	6	0	-7.720459	-1.345445	0.422999
119	6	0	-6.605023	-2.742950	-1.195454
120	6	0	-8.825411	-2.193566	0.392451
121	6	0	-7.710892	-3.591348	-1.223520
122	6	0	-8.827723	-3.320748	-0.431538
123	1	0	-7.721523	-0.474030	1.077649
124	1	0	-5.736646	-2.958736	-1.818953
125	1	0	-9.689574	-1.975658	1.024187
126	1	0	-7.699916	-4.470453	-1.872020
127	1	0	-9.693340	-3.986349	-0.452917
